# Spin state and orbital engineering of cuprous oxide for efficient nitrate reduction

**DOI:** 10.1038/s41467-026-76420-8

**Published:** 2026-08-15

**Authors:** Huicong Xia, Haihui Lan, Yue Yu, Yifan Wei, Zixin Li, Renqin Yu, Siran Xu, Yunchuan Tu, Mingkai Liu, Jia-Nan Zhang

**Affiliations:** 1 College of Materials Science and Engineering, Zhengzhou University, Zhengzhou, P. R. China; 2 College of Chemistry, Zhengzhou University, Laboratory of Pingyuan, Zhengzhou, P. R. China; 3 Department of Chemistry, Massachusetts Institute of Technology, Cambridge, USA; 4 School of Chemistry and Chemical Engineering, Chongqing University, Chongqing, P. R. China; 5 School of Chemistry and Chemical Engineering, Anhui University of Technology, Ma’anshan, P. R. China

**Keywords:** Electrocatalysis, Energy, Electrocatalysis, Electrocatalysis

## Abstract

Electrocatalytic nitrate reduction provides a sustainable pathway for wastewater treatment and ammonia production, yet the complex multi‑electron transfer demands efficient catalysts to achieve both high selectivity and fast kinetics. Here we show that atomic substitution of magnetic transition metals (Cr, Mn, Fe, Co, Ni) into cuprous oxide enables systematic tuning of catalytic performance. Density functional theory calculations combined with experiments identify that catalytic activity is governed by nitrate adsorption strength, ammonia desorption energy, and *d–p* orbital hybridization. Cobalt‑substituted cuprous oxide delivers a near‑unity Faradaic efficiency of 96.5% and an ammonia yield rate of 2.8 mM h^−1^ mg_cat_^−1^, showing favorable performance compared with the other metal‑doped counterparts. Mechanistic studies reveal that cobalt substitution alters the spin configuration at the active sites, which optimizes orbital hybridization and balances the adsorption and desorption of key intermediates. This work establishes a design principle linking atomic‑level spin and orbital engineering to catalytic performance, providing a framework for developing high‑performance nitrate reduction electrocatalysts.

## Abstract (web summary)

Electrocatalytic nitrate reduction offers a sustainable route for ammonia production and wastewater treatment. Here, the authors report cobalt-substituted cuprous oxide that optimizes spin states and orbital hybridization, achieving high ammonia selectivity and stability under ambient conditions.

## Introduction

Ammonia (NH_3_) is a cornerstone of modern agriculture and a promising carbon-free energy carrier. The traditional Haber-Bosch process, operating under harsh conditions (400–500 °C, 15–25 MPa) to cleave the inert N≡N triple bond (941 kJ mol^−^^1^), is energy-intensive and accounts for ~1.2% of global CO_2_ emissions^1–6^. While electrochemical nitrogen reduction (NRR) offers a sustainable alternative, it suffers from the low solubility of N_2_ and overwhelming hydrogen evolution, leading to poor Faradaic efficiency (FE < 10%) and impractical NH_3_ yield rates^7–9^. In contrast, the electrocatalytic nitrate reduction reaction (NO_3_RR) utilizes highly soluble nitrate (NO_3_^–^), an abundant aqueous pollutant, as a nitrogen source, thereby enabling simultaneous NH_3_ synthesis and water remediation with a considerably lower thermodynamic barrier^10–12^. This dual benefit positions NO_3_RR as a viable and scalable route for green ammonia production (Fig. S1).

However, efficient NO_3_RR requires precise orchestration of multi-step hydrogenation and deoxygenation^13–18^. A critical bottleneck is the dynamic supply and consumption of active hydrogen (*H) at the catalyst-electrolyte interface^19–21^. Catalysts with single-component active sites often exhibit mismatched kinetics for *H generation and NO_3_^−^ intermediate adsorption, leading to insufficient *H coverage or its wasteful recombination into H_2_^22–27^. To address this, recent strategies focus on constructing integrated catalytic systems with spatially adjacent but functionally complementary sites^28^. For instance, cascade designs where one site (e.g., Ru nanoparticles^29^) efficiently splits water to generate *H, while a neighboring site (e.g., Co single atoms^30^) strongly adsorbs and activates nitrate species, have achieved effective performance by establishing a seamless *H transfer channel^31–33^. This underscores the importance of atomic-level interface engineering in regulating the *H cycle, which is pivotal for enhancing both activity and selectivity toward NH_3_.

Building on this principle, herein we propose a single-atom Co doping strategy on Cu_2_O to construct a synergistic interface for NO_3_RR. The atomically dispersed Co sites are designed to serve as the primary centers for *H generation via optimized water dissociation, while the adjacent Cu sites facilitate the adsorption and stepwise reduction of nitrate intermediates. This intimate coupling at the atomic scale ensures efficient *H spillover and utilization, minimizing competitive HER. More importantly, beyond the geometric arrangement, the electronic structure of the Co sites, particularly their tunable spin state, plays a decisive role in fine-tuning the adsorption strength of key intermediates (e.g., *NO, *NOH). Stabilizing a specific Co^III^ spin configuration via doping optimizes the *d*-orbital hybridization with reaction intermediates, thereby lowering the energy barrier of the potential-determining step. Through a combination of catalyst synthesis, electrochemical evaluation, and density functional theory (DFT) calculations, we demonstrate that this single-atom interface engineering not only accelerates *H-involved kinetics but also regulates the reaction pathway at an orbital level. The optimized catalyst delivers a near-unity NH_3_ FE of 96.5% and a competitive yield rate compared with reported Cu-based catalysts. This work highlights atomic interface design with electronic spin modulation as a powerful strategy for advancing electrocatalytic NO_3_RR.

## Results

### Catalyst design of Co/Cu_2_O

To identify the optimal active phase for NO_3_RR, we employed DFT calculations to systematically investigate transition-metal catalysts that embedded in the Cu_2_O lattice. The key aim of this study is to elucidate the operative electron spin-state of the catalyst under electrochemical operating conditions. Prior researches have demonstrated that the catalytic performance is governed by three critical factors: (i) the atomic coordination environment of the active site^34^, (ii) the spatial distribution of electron density^35^, and (iii) the strength of reactant-surface interactions^36^. Furthermore, the dynamic evolution of the active phase (e.g., metallic, oxide, or mixed states) during electrochemical operation can significantly modulate the spin configuration. Understanding these structural and electronic transformations is essential for deciphering the interplay between spin states and catalytic activity, a relationship that remains elusive in NO_3_RR despite its established significance in related electrocatalytic processes.

**Fig. 1:**
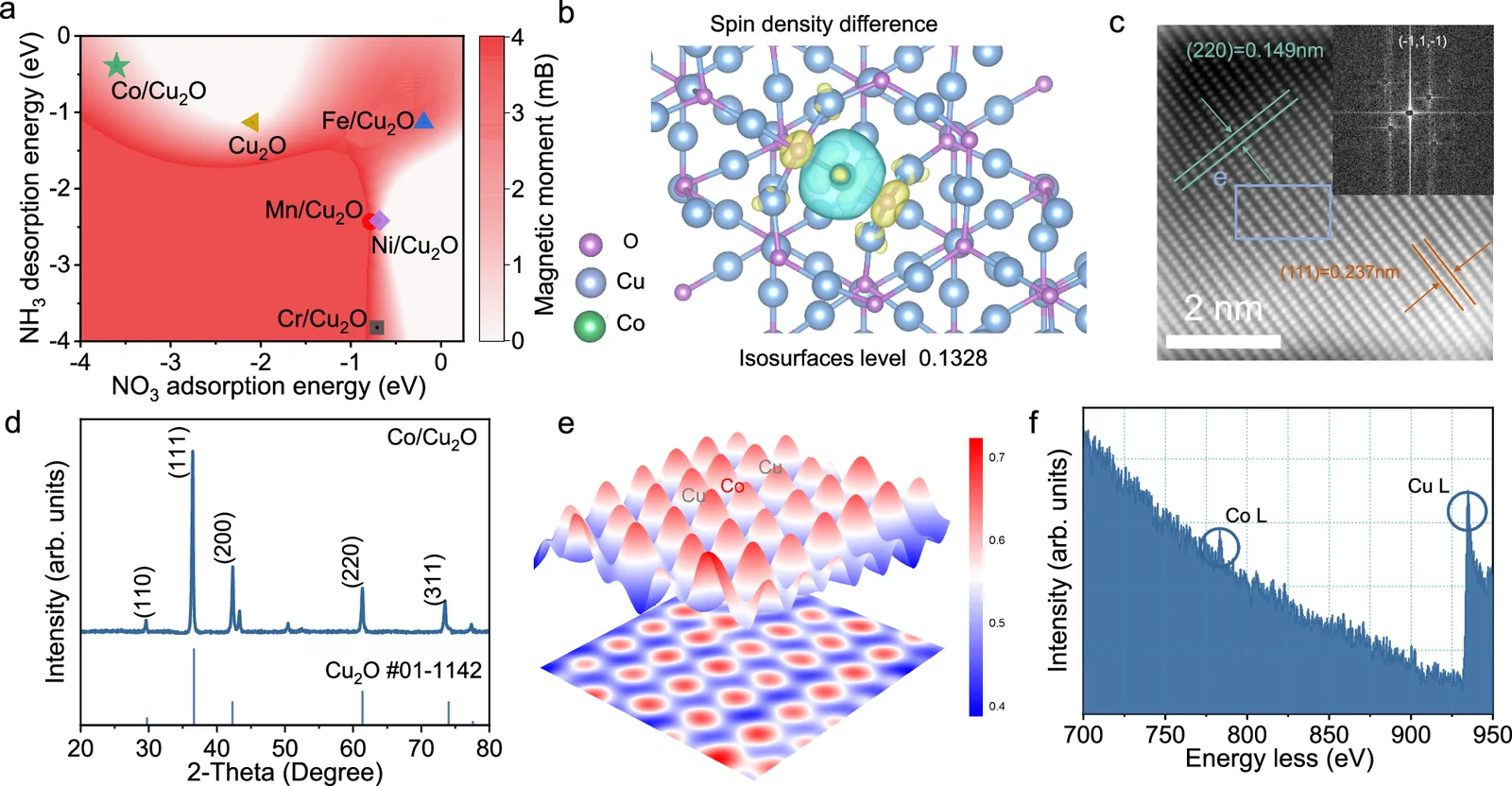
Theoretically prediction and structure confirmation of Co/Cu_2_O. **a** Two-dimensional activity heatmap of transition metal-doped Cu_2_O systems derived from density functional theory calculations. The plot correlates NO_3_ adsorption energy (horizontal axis) and NH_3_ desorption energy (vertical axis), with the color scale representing the calculated magnetic moment of each catalyst in units of Bohr magneton (μ_B_). **b** Spin density difference distribution of the Co/Cu_2_O lattice. Purple, light blue, and green spheres denote oxygen, copper, and cobalt atoms, respectively. The isosurface is set at a level of 0.1328 μ_B_, where yellow and cyan regions correspond to spin density accumulation and depletion induced by cobalt incorporation. **c** HAADF-STEM image of Co/Cu_2_O. The inset shows the corresponding fast Fourier transform (FFT) pattern. High-angle annular dark-field scanning transmission electron microscopy (HAADF-STEM) image of Co/Cu_2_O. The top-right inset presents the corresponding fast Fourier transform (FFT) pattern indexed to the (−1,1,−1) zone axis. **d** X-ray diffraction (XRD) pattern of the as-prepared Co/Cu_2_O sample. Standard diffraction positions of cubic Cu_2_O (PDF #01-1142) are displayed at the bottom as reference, with characteristic crystal planes labeled. **e** Three-dimensional atom-resolved surface profile of Co/Cu_2_O, illustrating the atomic arrangement on the exposed crystal plane. Cobalt and copper atomic columns are marked, and the color scale corresponds to the relative intensity of the atomic columns. **f** Electron energy loss spectroscopy (EELS) spectrum acquired from the Co/Cu_2_O sample. The characteristic L-edge signals of copper and cobalt are indicated, confirming the presence of cobalt species in the Cu_2_O matrix. Source data are provided as a Source data file.

To systematically evaluate the effects of transition-metal dopant on electrocatalytic performance, we conducted DFT calculations on Cu_2_O lattice models incorporating virous metals (e.g., Cr, Mn, Fe, Co, and Ni). The dopants exhibited distinct magnetic moments (Cr/Cu_2_O = 2.96 *μ*_**B**_, Mn/Cu_2_O = 3.97 *μ*_**B**_, Fe/Cu_2_O = 2.98 *μ*_**B**_, Co/Cu_2_O = 1.15 *μ*_**B**_, Ni/Cu_2_O = 0.74 *μ*_**B**_, and undoped Cu_2_O = 0 *μ*_**B**_; Fig. S2 and Table S1), consistent with their respective electronic configurations. Notably, Co/Cu_2_O exhibited competitive catalytic properties, showing the strongest NO_3_^−^ adsorption energy (Δ*E*_ad_ = −3.6 eV; Fig. S3) and the most favorable NH_3_ desorption energy (Δ*E*_de_ = −0.39 eV; Fig. S4). These values substantially exceeded those of other 3*d* metal-doped Cu_2_O, including Cr/Cu_2_O (Δ*E*_ad_ = −0.71 eV, Δ*E*_de_ = −3.82 eV), Mn/Cu_2_O (Δ*E*_ad_ = −0.78 eV, Δ*E*_de_ = −2.43 eV), Fe/Cu_2_O (Δ*E*_ad_ = −0.19 eV, Δ*E*_de_ = −1.13 eV), Ni/Cu_2_O (Δ*E*_ad_ = −0.68 eV, Δ*E*_de_ = −2.42 eV), and undoped Cu_2_O (Δ*E*_ad_ = −2.09 eV, Δ*E*_de_ = −1.13 eV). These findings underscore the critical role of spin-altered Co^III^ sites in promoting *d*–*p* orbital hybridization, which facilitates efficient charge transfer and lowers the kinetic barriers for NO_3_RR (Fig. 1a).

The catalytic performance of Co-substituted Cu_2_O is attributed to strong NO_3_^−^ adsorption at spin-altered Co^III^ sites within the Cu_2_O lattice, an effect mediated by interfacial electron transfer from Cu to Co that extends beyond purely electrostatic interactions^37,38^. Specifically, Co substitution stabilizes NO_3_^−^ adsorption by −3.6 eV and lowers the NH_3_ desorption energy barrier by −0.39 eV, effects attributed to synergistic electrostatic and spin-polarization effects (Fig. 1a). The catalysis enhancement arises from spin effects via two interconnected pathways: (i) spin-dependent charge transfer, which promotes preferential NO_3_^−^ adsorption on magnetic surfaces, and (ii) localized spin quenching at Co^III^ sites (Fig. S5), which optimizes *d*–*p* orbital hybridization and stabilizes transition states during *NO/*NOH conversion. This electronic restructuring disrupts the conventional scaling relationship between *NO adsorption and *NH_3_ desorption energies. Consequently, Co/Cu_2_O emerges as a transformative catalyst for NO_3_^−^ electroreduction, which combines strong reactant adsorption, facile product release, and spin-accelerated kinetics enabled by orbital engineering.

### Synthesis and characterization of metal-doped Cu_2_O catalyst

Building on the theoretical insights, metal-doped Cu_2_O catalysts were synthesized via a chemical-reduction method. Briefly, divalent Cu^II^ was reduced to monovalent Cu^I^ in the presence of varying concentrations of metal salts (e.g., Cr, Mn, Fe, Co, and Ni) using sodium borohydride, followed by freeze-drying to preserve an atomically ordered structure. The stabilization of Co^III^ within the Cu_2_O lattice, despite the use of NaBH_4_, is attributed to lattice confinement and the oxidizing ambient environment. Co^II^ is readily oxidized to Co^III^ during the synthesis, and the Cu_2_O matrix thermodynamically favors the incorporation of Co^III^ due to ionic radius matching and strong Co–O bonding. In contrast, without Cu^II^, Co^II^ precipitates as Co(OH)_2_ and remains as CoO. Characteristic peaks at 36.6° and 42.6° in X-ray diffraction (XRD) spectrum confirmed the existence of (111) and (200) planes of Cu_2_O in Co/Cu_2_O nanomaterial (Fig. 1d). By comparing with the standard XRD pattern of crystalline CoO (Fig. S6), we rule out the presence of crystalline CoO impurity, as none of the characteristic diffraction peaks of CoO are observed in the pattern. The weak shoulder on the higher 2*θ* side of the (200) peak is assigned to the Cu Kα_2_ satellite component, an inherent phenomenon of Cu-target XRD measurements, rather than an impurity phase. A similar XRD pattern was observed for undoped Cu_2_O, with no evidence of a secondary phase (Fig. S6). Transmission electron microscopy (TEM) revealed a well-defined rod-like morphology for Co/Cu_2_O, with diameters of approximately 40 nm and lengths exceeding 100 nm (Fig. S7). The one-dimension (1D) morphology is advantageous for solid-liquid interface reactions, as it provides an elongated reaction interface that mitigates mass-transport limitations caused by intermediate accumulation^39^. Energy-dispersive spectroscopy (EDS) mapping (Fig. S8) demonstrated a uniform distribution of Cu and O elements, indicating a homogeneous composition, while the Co signal remains relatively weak. Inductively coupled plasma-optical emission spectrometry (ICP-OES) quantified the Co content at 0.3 wt% (Table S2), further confirming the low-concentration doping. The aberration-corrected high-angle annular dark-field scanning TEM (AC-HAADF-STEM) (Fig. 1c) shows bright atomic-scale features. Note that lattice fringes with different orientations in the real-space image are projections of crystal planes from different specimen depths, and do not correspond to two sets of planes under the same crystallographic zone axis. Accurate crystal plane indexing and interplanar angle verification are based on the corresponding FFT pattern (inset of Fig. 1c). The measured angle between (111) and (220) diffraction spots is ~35.2°, which agrees well with the theoretical value of 35.3 for cubic Cu_2_O, further confirming phase purity and correct indexing. Electron energy loss spectroscopy (EELS) confirms the coexistence of Co and Cu at these locations, indicating the atomic dispersion of Co within the Cu_2_O matrix (Fig. 1f). High-resolution TEM (HRTEM) image revealed the (111) and (220) lattice planes of Cu_2_O (Fig. S9), with measured *d*-spacings of 0.273 and 0.149 nm, respectively. Additional HRTEM images confirmed the absence of aggregated metal cluster, and elemental mapping verified the homogeneous dispersion of other dopants on the Cu_2_O surface, such as Cr (Fig. S10), Mn (Fig. S11), Fe (Fig. S12), and Ni (Fig. S13). While some local enrichment of dopants is observed in EDX maps for certain samples (e.g., Mn), XAFS confirms atomic-level dispersion without metal-metal clustering.

High-resolution X-ray photoelectron spectroscopy (XPS) spectra of the Co 2*p* and Cu 2*p* regions (Fig. S14) indicate that both Co and Cu exist in high oxidation states, suggesting surface dominated by oxidized and/or hydroxylated species. Cu LMM Auger spectroscopy resolves Cu valence states with higher precision than conventional Cu 2*p* XPS (Fig. S15). Both Cu_2_O and Co/Cu_2_O are dominated by monovalent Cu^I^ (~916.8 eV), with no detectable divalent Cu^II^ (~917.7 eV) and only trace metallic Cu^0^ (~918.6 eV) from surface reduction^40^. Co doping reduces the Cu^0^ fraction, stabilizing the Cu^I^ state via electronic lattice interactions^41^. In the XPS spectra of metal/Cu_2_O samples (Figs. S16–S19), it is known that a main peak at approximately 933–935 eV with pronounced shake-up satellite features can be observed in the Cu 2*p* region, consistent with Cu^II^. Similarly, deconvolution of the Cr 2*p* region yields components characteristic of Cr^III^, indicating Cr–O coordination environments in the near-surface region. The Mn 2*p* spectrum is consistent with a mixture of Mn^III^ and Mn^IV^, suggesting a multivalent Mn–O coordination framework. Furthermore, the Fe 2*p* spectrum displays prominent peaks and satellites typical of Fe^III^, while the Ni 2*p* region is dominated by features characteristic of Ni^II^ with distinct satellite structures. Collectively, these results demonstrate the stabilization of Cr, Mn, Fe, and Ni high oxidation states within an oxygen-rich Cu_2_O lattice. This environment promotes the formation of abundant lattice defects and surface hydroxyl groups, which enhance the surface redox activity^42^. Three-dimension (3D) surface plots display varying peak heights (Fig. 1e), with relatively lower intensities correlated with the position of Co atoms. Complementary EELS confirms the atomic-level coexistence of Co and Cu (Fig. 1f), showing distinct L-edge peaks at 780 eV (Co L_3_) and 934 eV (Cu L_3_). The characteristic fine structure of Co L-edge provides definitive evidence for atomically dispersed Co^III^ species within the Cu_2_O matrix. The absence of spectral signatures for Co nanoparticle, combined with the observed atomic dispersion, demonstrates the precision of the synthesis in achieving single-atom-level Co substitution.

**Fig. 2:**
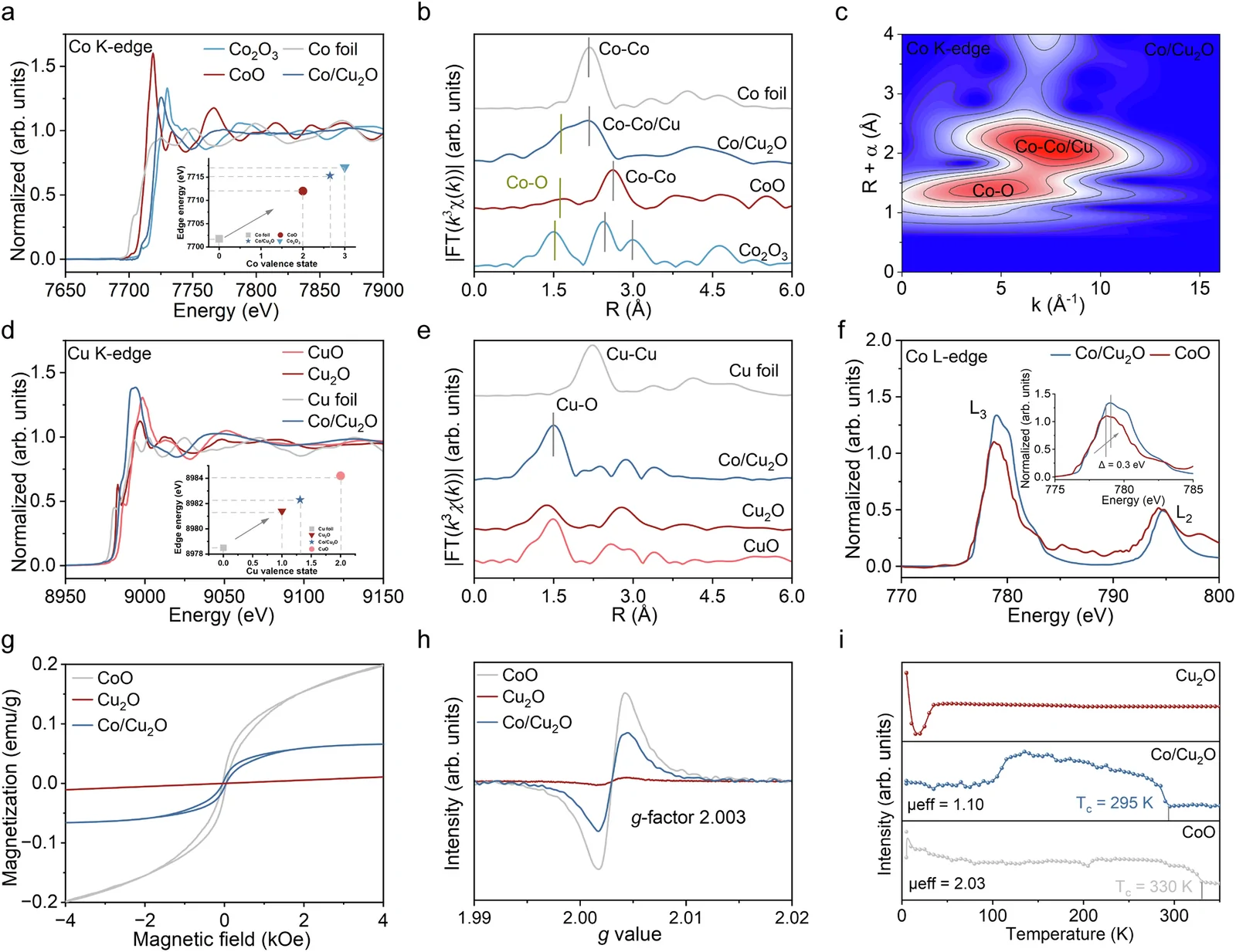
X-ray absorption spectroscopy, electron paramagnetic resonance, and magnetic susceptibility of the catalysts. **a** Co K-edge X-ray absorption near-edge structure (XANES) spectra of Co/Cu_2_O with CoO, Co_2_O_3_ and Co foil included as reference standards. The inset presents the calibration curve of absorption edge energy versus Co valence state, applied to determine the average oxidation state of Co in the Co/Cu_2_O sample. **b** Fourier-transformed extended X-ray absorption fine structure (FT-EXAFS) spectra in R space at the Co K-edge for Co/Cu_2_O, CoO, Co_2_O_3_ and Co foil. **c** Wavelet transform (WT) EXAFS contour plot of the Co K-edge for Co/Cu_2_O, which resolves scattering contributions in both k-space and R-space. **d** Cu K-edge XANES spectra of Co/Cu_2_O, with CuO, Cu_2_O, and Cu foil as reference standards. The inset shows the calibration curve of absorption edge energy versus Cu valence state for quantitative oxidation state analysis. **e** Fourier-transformed Cu K-edge EXAFS spectra in R space for Co/Cu_2_O, CuO, Cu_2_O, and Cu foil. **f** Co L-edge XANES spectra of Co/Cu_2_O and CoO reference, displaying the L_3_ and L_2_ absorption features. The inset provides a magnified view of the L_3_ peak region, where an energy shift of 0.3 eV is observed. **g** Field-dependent magnetization (M–H) curves of Co/Cu_2_O, Cu_2_O, and CoO collected at room-temperature (25 °C) over a magnetic field range of −4 to 4 kOe. **h** Room-temperature (25 °C) electron paramagnetic resonance (EPR) spectra of Co/Cu_2_O, CoO and Cu_2_O. **i** Temperature-dependent magnetic susceptibility curves of Co/Cu_2_O, CoO and Cu_2_O, measured across the temperature range of 5–350 K. The effective magnetic moment (μeff) and Curie temperature (Tc) are labeled for the magnetic samples. Source data are provided as a Source data file.

X-ray absorption fine structure (XAFS) spectroscopy was used to determine the coordination environment and oxidation state of the metal-doped Cu_2_O catalysts. The Co K-edge XANES spectrum of Co/Cu_2_O shows an edge position and near-edge features qualitatively similar to those of Co_2_O_3_ (Fig. 2a), suggesting a predominantly trivalent Co state. The slightly attenuated white-line intensity may reflect increased covalency or local distortion induced by lattice confinement. However, definitive oxidation state assignment relies on a combination of edge position, pre-edge features, and complementary techniques (XPS, EELS, magnetic measurements). The Co K-edge energy of Co/Cu_2_O is 7721.8 eV, compared to 7722.3 eV for Co_2_O_3_ and 7720.5 eV for CoO, which is consistent with a Co^III^-rich environment but does not exclude a small fraction of Co^II^. The *k*^3^-weighted Fourier transformed (FT) of the Co K-edge EXAFS resolves two distinct coordination shells: a primary Co–O scattering path at 1.5 Å and secondary contributions from Co–M (M = Co/Cu) interactions at 2.2 Å (Fig. 2b), comprising both octahedral–octahedral (Co_Oh_–M_Oh_) and octahedral–tetrahedral (Co_Oh_–M_Td_) configurations. The 3D wavelet transforms analysis confirms the absence of metallic Co nanoparticles (Fig. 2c and Fig. S20), with all intensity maxima corresponding exclusively to atomic-scale Co–O and Co–M scattering paths. Complementary Cu K-edge measurements show that the absorption-edge position of Co/Cu_2_O lies between Cu foil (Cu^0^) and Cu_2_O (Cu^I^) (Fig. 2d and Fig. S21), indicating minimal perturbation of the Cu oxidation state (predominantly +1) upon Co substitution. While EXAFS fitting of Cr/Cu_2_O, Mn/Cu_2_O, Fe/Cu_2_O, and Ni/Cu_2_O shows only a single scattering path in the first shell and no significant second-shell features (Figs. S22–S25), consistent with highly disordered or oxygen-rich coordination, Co/Cu_2_O exhibits a distinct second-shell peak (Fig. 2e and Fig. S20) attributable to Co–Cu backscattering at 2.85 Å. This feature arises from the favorable lattice incorporation of Co^III^ into the Cu_2_O matrix. EXAFS fitting confirms the local coordination environments of Co, Cr, Mn, Fe, and Ni dopants in Cu_2_O (Figs. S26–S30). All transition metals exist predominantly as M-O species rather than metallic phases, with distinct bond lengths and coordination numbers compared to their bulk oxides (Tables S3–S7). These atomically dispersed M-O sites anchored in the Cu_2_O lattice serve as the intrinsic active centers for efficient nitrate to ammonia electroreduction. Soft X-ray absorption spectroscopy (XAS) at the Co-L_2,3_ edges provide direct evidence for spin-state modification in Co/Cu_2_O (Fig. 2f). The spectra reveal three aspects of information: (i) a + 0.3 eV shift in the L_3_ edge relative to CoO, consistent with the higher oxidation state of Co inferred from XANES; (ii) distinctive intensity redistribution between the L_2_ and L_3_ regions, indicative of reduced 3*d*-electron occupancy (*t*_2_g^5^*e*g^1^ configuration); and (iii) consistency with calculations for intermediate-spin Co^III^ (*S* = 1). Collectively, these XAFS and soft-XAS results demonstrate that lattice-confined Co^III^ sites adopt a spin-altered electronic configuration that enhances *d*–*p* hybridization with adjacent oxygen atoms and maintains integration within the Cu_2_O framework. The combined EXAFS, HAADF-STEM and EELS are consistent with atomically dispersed Co within the Cu_2_O matrix, with no evidence of Co clusters within the detection limits of these techniques.

Magnetic characterization of Co/Cu_2_O, Cu_2_O, and CoO was conducted to probe their electron configuration and spin states. Magnetic hysteresis loops measured at 300 K (Fig. 2g and Fig. S31) revealed distinct behaviors, key parameters, including saturation magnetization (*M*s), remanent magnetization (*M*r), and coercivity (*H*c), are summarized in Table S8. Undoped Cu_2_O exhibited no hysteresis under an applied magnetic field, consistent with its diamagnetic nature^43^. In contrast, Co/Cu_2_O displayed a clear ferromagnetic hysteresis loop, confirming Co-induced spin polarization. Although the *M*s values range from 0.053 to 0.42 emu·g^−1^ across the samples, the emergence of ferromagnetism in Co/Cu_2_O highlights the pronounced impact of atomic-scale Co incorporation on the magnetic properties. The observed ferromagnetism in Co/Cu_2_O sample indicates that localized 3*d*-electron reorganization at the Co sites induces spin polarization. This phenomenon was occurred without a significant increase in the overall *M*s, suggesting that the effect arises primarily from spin reorientation rather than moment amplification. The pronounced hysteresis in Co/Cu_2_O further supports the enhancement of localized spin polarization upon Co doping.

Electron paramagnetic resonance (EPR) spectroscopy, a technique highly sensitive to unpaired electrons, was employed to further substantiate this interpretation. The resonance condition is given by Δ*E* = *hυ* = *gμ*_**B**_*β*, where *h* is Planck’s constant (6.626 × 10^−27^ erg/s), *v* is the microwave frequency (9.85 GHz), *g* is Landé factor (2.003 ± 0.002), *μ*_**B**_ is the Bohr magneton (9.274 × 10^−21^ erg/G, and **B** is the applied magnetic field (G)^44^. The EPR spectra show that all samples exhibit a *g*-factor of 2.003 (Fig. 2h), indicating similar spin–orbit coupling environments. However, undoped Cu_2_O showed a negligible EPR response at 25 °C, whereas CoO exhibits a markedly stronger signal than Co/Cu_2_O, reflecting their distinct spin states. These results are consistent with the magnetization-field (M–H) data, undoped Cu_2_O shows no significant magnetic response, while Co/Cu_2_O exhibits magnetic behavior intermediate between those of Cu_2_O and CoO, underscoring the localized-spin effects induced by Co substitution.

To further probe the magnetic behavior, temperature-dependent magnetic susceptibility (M–T) measurements were conducted (Fig. 2i). The data obey the Curie–Weiss law^45,46^: *χ* = *C/*(*T* *–* *θ*), *C* = *μ*_eff_^2^/8, where *χ* is the magnetic susceptibility, *C* is the Curie constant, *θ* is the Weiss temperature, and *μ*_eff_ is the effective magnetic moment. The Curie–Weiss fitting allows analysis of the exchange interactions, where a negative *θ* value indicates dominant antiferromagnetic coupling in these systems. The derived *μ*_eff_ values are consistent with intermediate-spin Co^III^ states (*S* = 1), where the orbital angular momentum is largely quenched by the octahedral crystal field. Collectively, these results corroborate that Co incorporation modifies the spin configuration and enhances the materials magnetic response, consisting with the M–H and EPR analyses presented above.

### NO_3_RR performance

**Fig. 3:**
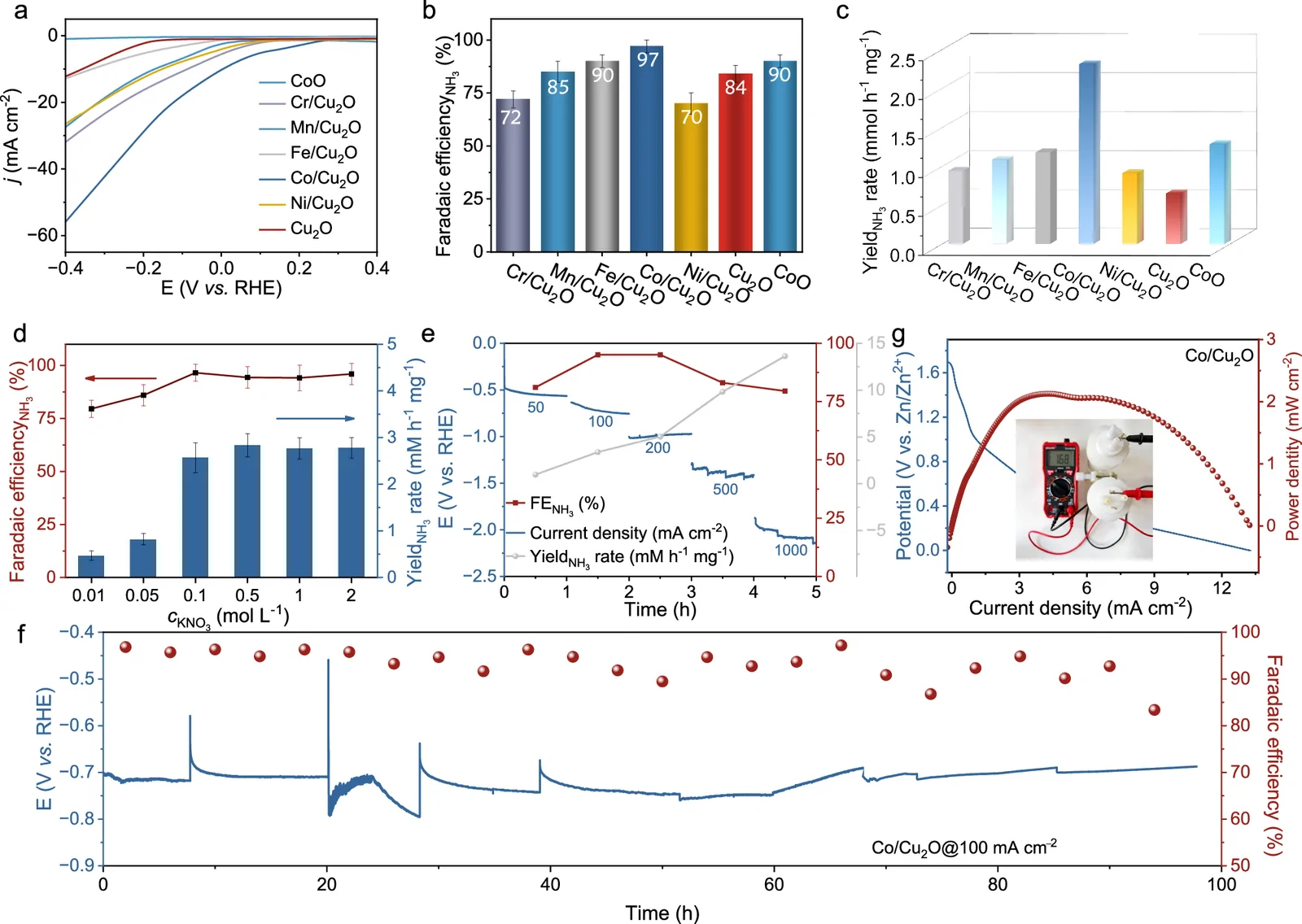
NO_3_RR electrocatalytic performances. **a** Linear sweep voltammetry (LSV) polarization curves of Co/Cu_2_O, Cr/Cu_2_O, Mn/Cu_2_O, Fe/Cu_2_O, Ni/Cu_2_O, Cu_2_O, and CoO catalysts, recorded at a rotation rate of 900 rpm in 0.1 M KOH + 0.1 M KNO_3_ electrolyte at 25 °C. Measurements were collected over a potential window of −0.6 to 0.5 V *vs*. reversible hydrogen electrode (RHE) without iR compensation, with a catalyst loading of 0.1 mg cm^−2^ for all samples. **b** Faradaic efficiency of NH_3_ production for each catalyst at the optimal applied potential. All data are all presented as mean ± standard deviation (s.d.), with error bars derived from three independent replicate electrolysis experiments. **c** Yield rate for NH_3_ of the series of catalysts, calculated from quantitative product analysis after controlled-potential electrolysis. **d** NO_3_RR performance of Co/Cu_2_O measured at graded KNO_3_ concentrations, showing the variation of Faradaic efficiency and NH_3_ yield rate with reactant concentration. All data are shown as mean ± s.d. (*n* = 3). Error bars represent standard deviation. **e** Chronopotentiometry profiles of Co/Cu_2_O recorded at stepwise increased current densities in 0.1 M KNO_3_  +  0.1 M KOH electrolyte, paired with the NH_3_ yield rates obtained at each current density level. **f** Long-term electrocatalytic stability test of Co/Cu_2_O for NO_3_RR, conducted at a constant current density of 100 mA cm^−2^ in a continuous-flow H-cell with 0.1 M KNO_3_ + 0.1 M KOH electrolyte at room-temperature (25 °C). The blue curve tracks the working potential over 100 h of continuous operation, and red dots represent the Faradaic efficiency of NH_3_ production measured at selected time intervals. **g** Discharge curve and corresponding power density profile of a Zn-NO_3_^−^ battery assembled with Co/Cu_2_O as the cathode catalyst. The inset shows a digital photograph of the assembled single cell during open-circuit voltage measurement. Source data are provided as a Source data file.

The electrocatalytic performance of Co/Cu_2_O for the NO_3_RR was evaluated using a rotating-disk electrode (RDE) setup in 0.1 M KOH electrolyte with or without 0.1 M KOH + 0.1 M KNO_3_. Linear sweep voltammetry (LSV) curves show that Co/Cu_2_O delivers substantially higher current densities than Cu_2_O, CoO, and other metal/Cu_2_O samples (Fig. 3a), indicating its competitive NO_3_RR activity. Co/Cu_2_O achieves the highest NH_3_ yield and FE among all tested catalysts (Fig. 3b, c), consisting with a synergistic tandem catalysis mechanism. At a benchmark current density of 10 mA cm^–2^, Co/Cu_2_O operates at the lowest potential (Table S9), highlighting its high catalytic efficiency. Polarization curves acquired at varying rotation rates indicated negligible mass-transfer resistance at speed exceeding 900 rpm (Fig. S33). Measurements in the nitrate-free electrolyte confirmed effective suppression of the HER. Chronoamperometry and colorimetric assays quantified NO_2_^−^ and NH_3_ production (Figs. S32–S39). Co/Cu_2_O delivering an FE of 96% and an NH_3_ yield of ~1.4 mM h^−1^ mg^−1^ at −0.3 V (*vs*. RHE). Even after normalization by the electrochemical surface area (ECSA) (Figs. S40–S43 and Table S10), Co/Cu_2_O maintains competitive NO_3_RR performance at low overpotentials, underscoring its high intrinsic activity. These results demonstrate that Co/Cu_2_O achieves optimized NO_3_RR performance, driven by its electronic structure. This structure is characterized by electron redistribution at the Co–Cu interfacial, where electrons donation from Co to Cu_2_O optimizes *d*-band center. This optimized electronic configuration facilitates NO_3_^−^ adsorption and activation while suppressing the competing HER.

The FE and NH_3_ yield of Co/Cu_2_O were benchmarked against leading reported metal-based NO_3_RR catalysts (Table S11), demonstrating its competitive performance in both metrics. To evaluate its practical applicability, Co/Cu_2_O was systematically tested under industrially relevant operating conditions. As shown in Fig. 3d, the catalyst maintained high activity over a broad nitrate concentrations range (0.01–2.0 M), demonstrating its adaptability to variable feedstock compositions. Chronopotentiometry at industrially relevant current densities of 50–1000 mA cm^−2^ revealed its robust performance (Fig. 3e). As that, although HER competition intensified above 200 mA cm^–2^, the Co/Cu_2_O catalyst sustains an FE of ~80% for NH_3_ and achieved a yield of 13.6 mM h^−1^ mg^−1^. Long-term stability tests at 100 mA cm^−2^ further confirmed stable operation for nearly 100 h without detectable activity loss (Fig. 3f). These results underscore the competitive catalytic properties of Co/Cu_2_O. Inspired by the NO_3_RR activity of Co/Cu_2_O, we assembled a Zn-NO_3_^−^ battery device using Co/Cu_2_O as the cathode, a Zn plate as the anode, and 1.0 M KOH + 0.1 M KNO_3_ as electrolyte (Fig. S44). This battery delivered a stable open circuit voltage (OCV) of 1.68 V *vs*. Zn/Zn^2+^, a peak power density of 2.4 mW cm^−2^, and a discharge current density of 4.0 mA cm^−2^ at 0.45 V (Fig. 3g). Furthermore, the Zn-NO_3_^−^ battery exhibited no degradation over 900 min of galvanostatic discharge and charge cycling (Fig. S45), demonstrating stable cycling performance.

The chemical stability of catalyst was confirmed by post-cycling XPS analysis (Fig. S46), which shows no change in the element’s oxidation states. HAADF-STEM images (Fig. S47) reveal well-preserved lattice fringes. Complementary EELS analysis verifies the retention of Co species and the structural integrity of the single-atom Co sites after prolonged operation. Furthermore, EDS elemental mapping shows no detectable changes compared to the pristine sample, conclusively demonstrating its structural and compositional stability under operating conditions. Critically, the combined data from XPS, EELS, and EDS show no evidence of Co nanoparticle aggregation or leaching within the detection limits of these techniques, confirming that the active single-atom sites remain intact throughout extended electrolysis.

**Fig. 4:**
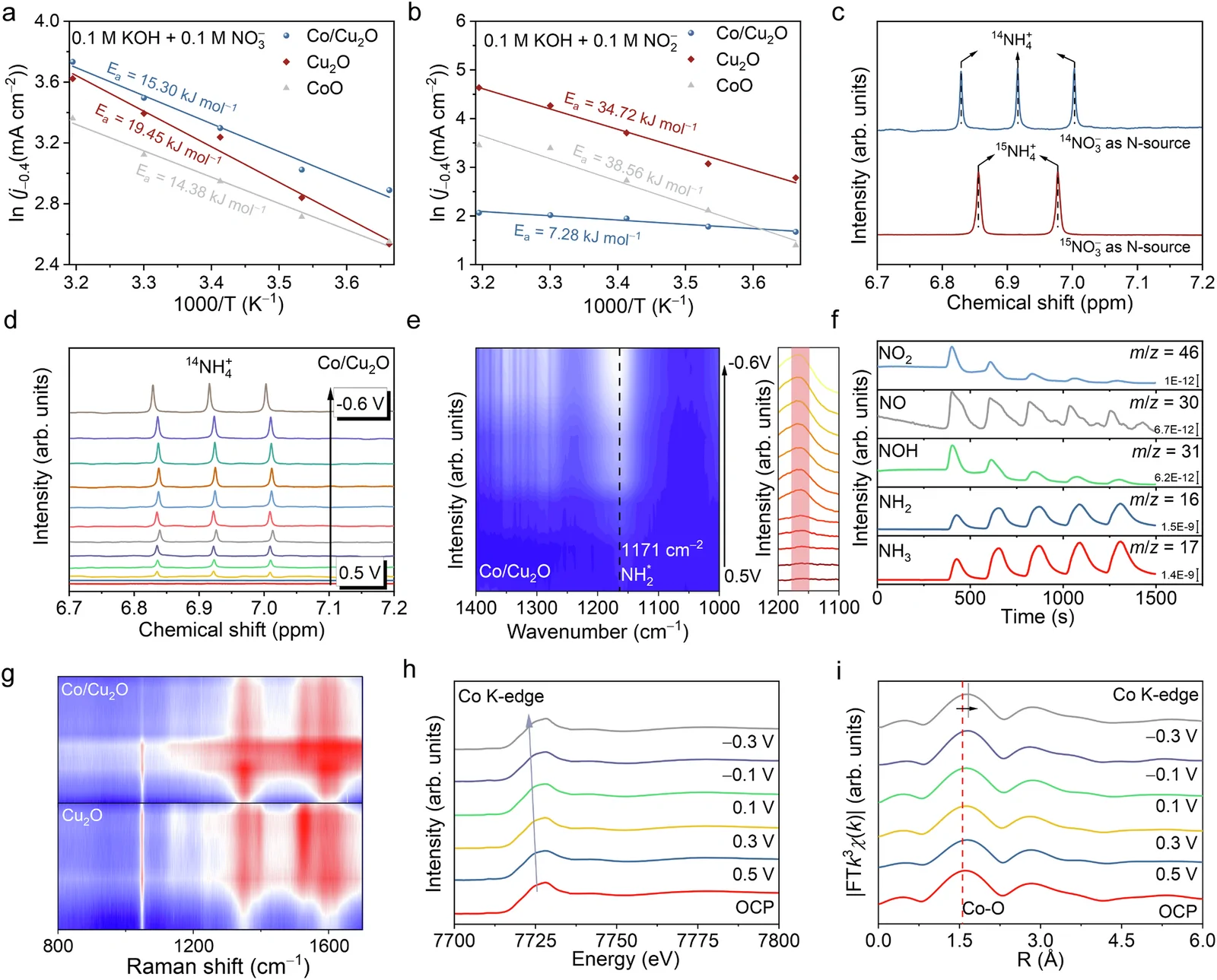
Investigation of active sites. Arrhenius plot and derived apparent activation energy (*E*_a_) values of Co/Cu_2_O, CoO, and Cu_2_O catalysts in **a** 0.1 M KOH + 0.1 M NO_3_^–^ electrolyte and **b** 0.1 M KOH + 0.1 M NO_2_^–^ electrolyte. The current density recorded at −0.4 V *vs*. reversible hydrogen electrode (RHE) under graded temperatures is adopted for *E*_a_ calculation. **c** ^1^H nuclear magnetic resonance (^1^H NMR) spectra of NO_3_RR products using ^14^NO_3_^–^ and ^15^NO_3_^–^ as the nitrogen source, respectively, confirming that the generated ammonium originates from the electrochemical reduction of nitrate reactant rather than side reactions. **d** ^1^H NMR spectra of NO_3_RR products over Co/Cu_2_O collected at a series of applied potentials (from 0.4 V to −0.6 V *vs*. RHE), showing the potential-dependent formation trend of NH_4_^+^. **e** In situ Fourier transform infrared (FTIR) spectra of Co/Cu_2_O collected during NO₃RR across the potential range from 0.5 V to −0.6 V *vs*. RHE at room-temperature (25 °C). **f** In situ differential electrochemical mass spectrometry (DEMS) profiles of NO_3_RR over Co/Cu_2_O monitoring the dynamic evolution of critical nitrogen-containing intermediate species with reaction time. **g** In situ Raman spectra of Co/Cu_2_O (top) and Cu_2_O (bottom) collected at various applied potentials and open circuit potential (OCP). In situ X-ray absorption spectroscopy characterizations of Co/Cu_2_O at different applied potentials. **h** Co K-edge X-ray absorption near-edge structure (XANES) spectra, reflecting the dynamic valence state evolution of Co sites under working conditions. **i** Fourier-transformed extended X-ray absorption fine structure (FT-EXAFS) spectra in R space. Source data are provided as a Source data file.

The origin of the enhanced catalytic activity of single-atom Co confined in Cu_2_O lattices was investigated using a combination of in-situ and quasi-in-situ characterizations paired with electrochemical analyses. Arrhenius plots were constructed from temperature-dependent LSV measurements on CoO, Co/Cu_2_O, and Cu_2_O (Figs. S48 and S49), and the corresponding activation energies (*E*_a_) were extracted via linear-regression. Comparative studies in electrolytes containing NO_3_^−^ versus NO_2_^−^ reveal distinct catalytic behaviors (Fig. 4a, b). Specifically, CoO and Co/Cu_2_O exhibit a lower activation barrier for the *NO_3_ to *NO_2_ conversion, highlighting the critical role of Co. In contrast, Cu_2_O shows higher activity for NO_2_RR, as evidenced by its lower *E*_a_. These observations indicate the Cu sites effectively promote the downstream reaction steps. These complementary roles establish a synergistic catalytic mechanism: Co preferentially activates the initial NO_3_^−^ to NO_2_^−^ step, whereas Cu optimizes the subsequent NO_2_RR dominated transformations, thereby enhancing the overall reaction efficiency.

Isotope-labeling experiments with ^15^N-labeled KNO_3_ were performed to unequivocally identify the origin of the produced ammonia. ^1^H-NMR analysis of the post-electrolysis electrolyte detected the characteristic double peaks for ^15^NH_4_^+^ when ^15^N-labeled KNO_3_ was used (Fig. 4c), and the triplet for ^14^NH_4_^+^ with unlabeled KNO_3_, confirming that the NH_3_ originated exclusively from electrocatalytic NO_3_⁻ reduction. Consistently, potential-dependent ^1^H-NMR spectra showed the emergence of ^14^NH_4_^+^ signals at 0.3 V (*vs*. RHE) (Fig. 4d), in agreement with LSV and chronoamperometry, thus defining the onset potential for efficient NH_3_ production.

In situ infrared (IR) spectroscopy was used to monitor the reaction intermediates during NO_3_RR in 0.1 M KOH + 0.1 M KNO_3_ electrolyte. Upon decreasing the potential, a distinct band at 1171 cm^−1^ emerged for Co/Cu_2_O, Cu_2_O, and CoO (Fig. 4e and Fig. S50), which is attributed to the *NH_2_ intermediate formed in the terminal step of the NO_3_RR. Both Co/Cu_2_O and CoO exhibited *NH_2_ band at ~0.3 V, underscoring the role of Co in promoting this step. However, the attenuated *NH_2_ band intensity for CoO indicates its limited efficiency in facilitating subsequent *NO_2_ protonation, consistent with its higher activation energy barrier. These results indicate that Co sites are essential for *NH_2_ generation, while their catalytic effectiveness is strongly influenced by the local chemical environment. The enhanced performance of Co/Cu_2_O is attributed to synergistic Co–Cu interactions. Besides, as shown in Fig. 4f, the in situ differential electrochemical mass spectrometry (DEMS) study reveals the dynamic evolution of key reaction intermediates and products. Mass signals corresponding to NO_2_ (*m/z* = 46), NO (*m/z* = 30), and NOH (*m/z* = 31) appear early in the reaction, confirming the sequential formation of nitrogen-oxygen intermediates^47^. Notably, signals for NH_2_ (*m/z* = 16) and NH_3_ (*m/z* = 17) emerge shortly afterward, with their intensities progressively increasing over time. This sequential evolution directly corroborates the stepwise reaction pathway: NO_3_^−^ → NO_2_^−^ → NO → NOH → NH_2_ → NH_3_. Combined with the *NH_2_ intermediate signal observed in in situ FTIR, these DEMS results provide unambiguous mechanistic evidence for the NO_3_⁻ to NH_3_ conversion process on the Co/Cu_2_O catalyst.

To further elucidate the reduction pathway, in-situ electrochemical Raman spectroscopy was employed to probe Co/Cu_2_O and Cu_2_O (Fig. 4g and Fig. S51). At an applied potential of 0.5 V (*vs*. RHE), both catalysts displayed Raman bands at 1049 cm^−1^ (aqueous NO_3_^−^) and at 1352/1525 cm^−1^ (adsorbed NO_3_^−^, denoted as NO_3_^−^-Ad). Co/Cu_2_O exhibited significantly higher band intensities, suggesting stronger NO_3_^−^ adsorption. As the potential decreased to 0.3 V, a NO_2_^−^ band emerged at 1330 cm^−1^ on Co/Cu_2_O, assigned to the *υ*(N-O) stretching mode of adsorbed NO_2_⁻. This band gradually diminished and disappeared by −0.6 V. Concurrently, NH_3_-related bands were observed at 0.1 V, 1130 cm^−1^ (*υ*_2_ mode of NH_3_) and 1480 cm⁻^1^ (antisymmetric vibration of NH_4_^+^). The intensity of these bands increased at more negative potentials, indicating efficient conversion of NO_2_^−^ to NH_3_ through sequential generation, desorption, readsorption and transformation steps. In contrast, Cu_2_O exhibited negligible changes in the NO_2_^−^ band intensity even at −0.6 V. Furthermore, its NH_3_-formation bands appeared at 0 V, which is 100 mV more negative than the corresponding potential on Co/Cu_2_O. Collectively, these results indicate that Co/Cu_2_O facilitates direct NO_3_^−^ reduction at more positive potentials, owing to its strong activity for the entire reaction pathway.

The dynamic evolution of the local electronic and coordination structure of Co/Cu_2_O under operating NO_3_RR conditions was probed by in situ Co K-edge XANES and EXAFS measurements. In stark contrast to the expected reduction under cathodic potentials, the main absorption edge exhibits a pronounced positive shift as the potential decreases from 0.5 V to −0.3 V (Fig. 4h), indicating a gradual increase in the average Co oxidation state from Co^II^ to Co^III^. This counterintuitive oxidation arises from the strong oxidizing nature of adsorbed nitrate and its key reduction intermediates, which dynamically oxidize surface Co^II^ sites to form high-valent Co^III^-O active species. While the pristine Co/Cu_2_O features predominantly high-spin Co^II^ (*S* = 3/2), oxidation to intermediate-spin Co^III^ (*S* = 1) under conditions strengthens *d–p* hybridization with adsorbates. Such potential-induced spin transitions have been reported in related oxide systems (e.g., LaCoO_3_^48^, SrCoO_3_^49^) and directly optimize binding of the rate-limiting *NO intermediate. The Co oxidation state fully reverts to its initial value upon returning to open circuit potential, demonstrating the reversible nature of this dynamic redox process. In situ EXAFS at the Co K-edge (Fig. 4i) was analyzed and compared with the spectrum of a standard CoO reference. No additional coordination shells or scattering paths were detected across the potential window, further confirming the stability of the local Co–O environment during the electrochemical cycling. Taken together, these results highlight the robust structural integrity and reversible redox behavior of the Co sites in Co/Cu_2_O under NO_3_RR conditions.

### Mechanism for the enhanced NO_3_RR activity

**Fig. 5:**
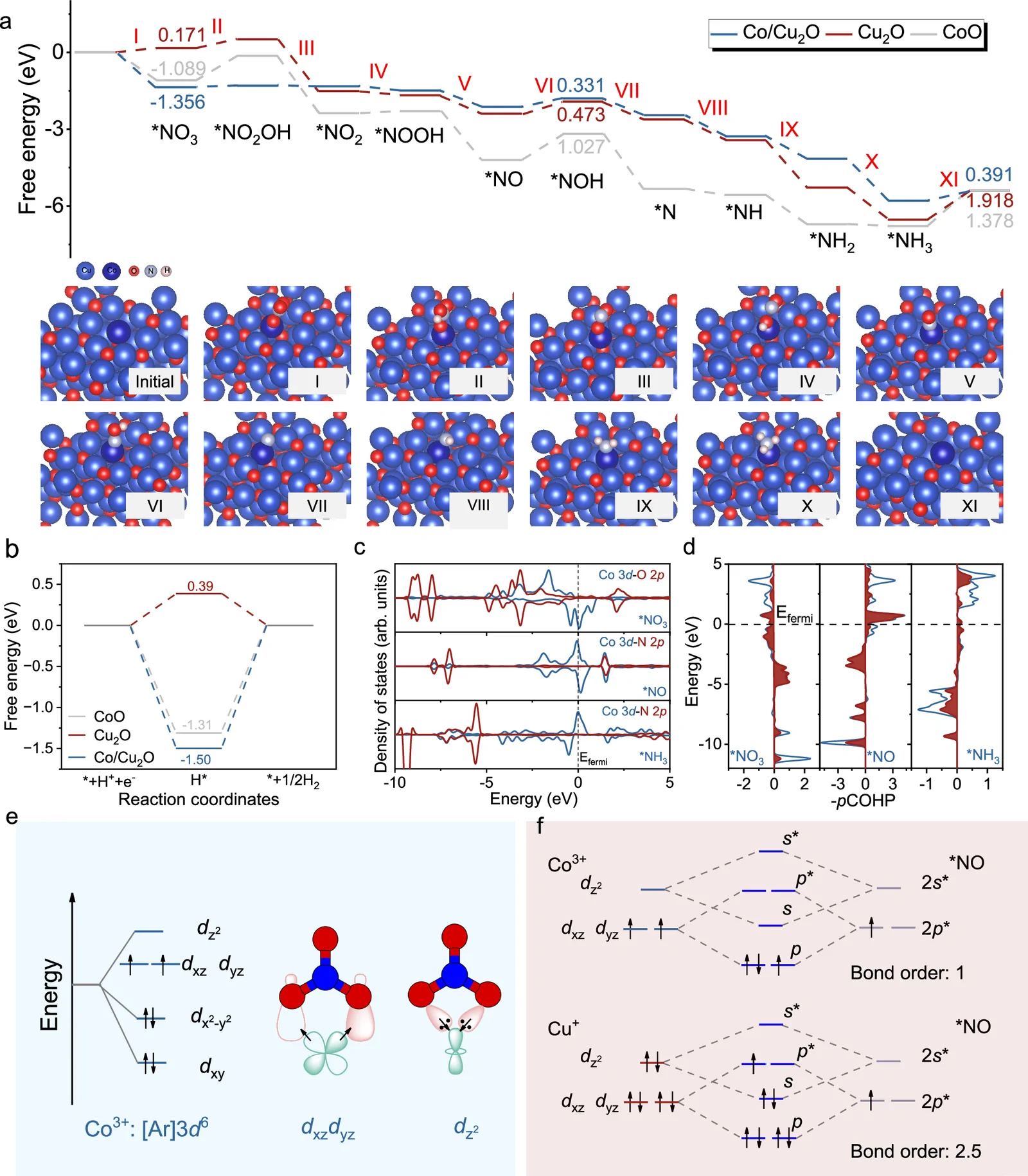
Theoretical study of the enhanced performance of Co/Cu_2_O electrocatalyst. **a** Gibbs free-energy diagram of NO_3_RR pathway on Co/Cu_2_O, CoO, and Cu_2_O catalysts. Optimized atomic configurations of each reaction intermediate adsorbed on the Co/Cu_2_O surface are presented below the energy profile. **b** Calculated hydrogen adsorption free energy on the catalytic sites of Co/Cu_2_O, CoO, and Cu_2_O, describing the thermodynamic favorability of the competitive hydrogen evolution side reaction. **c** Projected density of states (PDOS) of Co 3*d* orbitals and the adsorbed *NO_3_, *NO, *NH_3_ intermediates on Co/Cu_2_O, revealing the electronic coupling between the Co active center and reaction intermediates. The Fermi level is indicated by the dashed line. **d** Integrated crystal orbital Hamilton population (ICOHP) analysis of the bonding interactions between Co 3*d* orbitals and adsorbed *NO_3_, *NO, and *NH_3_ species. **e** Schematic illustration of the electronic ground state of lattice Co^3+^ in the Co/Cu_2_O matrix, and the orbital interaction between Co 3*d* orbitals and NO_3_^–^ adsorbate, showing the selective orbital overlap between *d*_xz_/*d*_yz_ and *d*_z_^2^ orbital with adsorbate. **f** Schematic diagram of orbital hybridization between adsorbed *NO molecule orbitals and metal 3*d* orbitals of Co/Cu_2_O (top) and Cu_2_O (bottom). Source data are provided as a Source data file.

DFT calculations were performed to investigate the origin of the enhanced NO_3_RR performance of Co/Cu_2_O for NH_3_ generation through a systematic analysis of electronic structures and reaction energetics. Comparative DFT evaluations of NO_3_^−^ and proton (H*) adsorption energies on CoO, Cu_2_O, and Co/Cu_2_O (Figs. S52–S54) suggest that Co/Cu_2_O exhibits the most favorable energetics for both adsorbates, which may facilitate the subsequent reduction steps along the NO_3_RR pathway. The relatively strong proton binding on Co/Cu_2_O effectively suppresses the competing HER, which likely contributes to the high selectivity toward NH_3_. Reaction pathway analysis identifies the *NO to *NOH conversion as a potential RDS for Co/Cu_2_O, with a calculated free-energy barrier of approximately 0.331 eV (Fig. 5a). In contrast, CoO and Cu_2_O exhibited higher calculated barriers (1.027 and 0.473 eV, respectively) for this step, suggesting a limitation in their NH_3_ production efficiency. Thermodynamic analysis further indicates that Co/Cu_2_O shows more favorable*NO_3_ adsorption and *NH_3_ desorption energetics compared with CoO and Cu_2_O within our computational model, which is broadly consistent with experimental observations. A detailed investigation of HER energetics reveals that Cu_2_O possesses the highest calculated HER barrier, which may be attributable to overly strong proton binding (Fig. 5b and Fig. S55). Although Co/Cu_2_O exhibits a more negative ΔGH (i.e., stronger H* binding) than Cu_2_O within the DFT model, the competitive adsorption of NO_3_^–^ and its reduction intermediates (NO, NOH) at the Co sites effectively blocks H formation and suppresses HER under operating conditions. This site-blocking effect, combined with the faster kinetics of NO_3_RR, likely contributes to the high selectivity toward NH_3_.

The thermodynamic origin of this behavior was investigated by analyzing the Co 3*d*-orbital energy-level splitting in the Co/Cu_2_O system. In this structure, Co^III^ substitutes for a two-coordinate, surface-unsaturated Cu site. Under a local out-of-plane electric field (along the *z-*direction), the Co *d*-orbitals undergo crystal-field splitting, which preferentially raises the energies of the *d*_z_^2^, *d*_xz_, and *d*_yz_ orbitals. This electronic configuration enables effective *s–p–d* hybridization and may facilitates coordination bond formation. The dense metal-atom packing in Cu_2_O induces weak M-Co interactions^50^, which may stabilize Co in an electron-deficient trivalent state. Magnetic characterization is consistent with a middle-spin configuration for Co^III^ (Fig. 5e), with crystal-field splitting playing an important role in modulating the catalytic activity. The interaction between Co centers and nitrate appears to enhances NO_3_RR through two possible synergistic mechanisms: (i) nitrate oxygen atoms donate lone-pair electrons to from *σ*-coordinate bonds with Co centers; (ii) nitrate π* orbitals (associated with N-O bonds) accept electrons via *d–π* back-donation from filled Co *d*-orbitals. This dual-interaction scheme polarizes the nitrate anion, strengthens its adsorption, and promotes the subsequent reduction steps.

The *d*_xz_ and *d*_yz_ orbitals exhibit a strong propensity to hybridize with the O 2*p*-orbitals reaction intermediates, which may facilitate bond formation. Bonding-level theory was used to examine orbital hybridization during the potential RDS (*NO to *NOH). As shown in Fig. 5f, for Co^III^, hybridization between the *NO 2*π* orbital and the degenerate *d*_xz_/*d*_yz_ orbitals yields a *π*-bond with a bond order of approximate 1. The calculated π-bond order for Cu^I^–NO is higher than that for Co^III^–*NO, yet Cu^I^ exhibits weaker *NO adsorption within the DFT model. This apparent discrepancy arises because the bond order alone does not account for orbital energy alignment. For Cu^I^ (3*d*^10^), the filled *d*-orbitals lie close in energy to the *NO *π* orbitals, leading to significant back-donation that populates π*-antibonding states and weakens the net interaction. In contrast, Co^III^ (*t*_2_g^5^*e*g^1^) achieves *d*–π* hybridization with less antibonding occupation, resulting in stronger adsorption. Charge density difference analysis of adsorbed NO_3_^−^ is consistent with electron transfer from the metal *d* orbitals (Cu/Co) to NO_3_^−^ via *d* → π* back-donation (Fig. S56), an effect that is most pronounced for Co/Cu_2_O. Projected density of states (PDOS) analysis reveals pronounced hybridization between the Co 3*d* and nitrate O 2*p* orbitals near the Fermi level (Fig. 5c and Figs. S57–S59). This hybridization may account for the robust adsorption NO_3_^−^ on Co/Cu_2_O and may facilitates the initial steps of the NO_3_RR. For adsorbed *NO, hybridization between Co 3*d* and N 2*p* orbitals occurs at higher energies. Integrated crystal orbital Hamilton population (ICOHP) analyses (Fig. 5d, Figs. S60 and S61) further show contributions from the *d*-orbitals to antibonding states. This combination may weakens *NO adsorption and lowers the calculated free-energy barrier for the NO to NOH conversion.

To further validate our computational approach, we performed GGA + U calculations incorporating Hubbard U corrections for all catalyst models. The PDOS and ICOHP analyses for the key intermediates (*NO_3_, *NO, and *NH_3_) are presented in Figs. S62–S69. As shown in these figures, the GGA + U results reproduced the same relative activity order observed at the standard GGA level. Co/Cu_2_O consistently displayed the strongest *d‑p* orbital hybridization with *NO_3_, evidenced by the most pronounced peak overlap near the Fermi level and the most negative ICOHP values, suggesting the most favorable bonding interaction among the models considered. Although the U correction systematically shifted the *d* band centers to lower energies, it did not alter the relative trends among the four catalysts. This indicates that our original conclusions based on GGA calculations are robust and not sensitive to the choice of U value within the tested range. Therefore, the enhanced activity of Co/Cu_2_O may originates primarily from its geometric and electronic structure rather than from correlation effects captured solely by the U term.

## Discussion

In summary, this work elucidates the critical relationship between *NO reaction intermediates and the NO_3_RR performance of Co/Cu_2_O hybrids. The strategic incorporation of Co into the Cu_2_O matrix significantly enhanced the efficiency of NH_3_ synthesis. This architectural framework not only polarizes the Co 3*d*-orbitals but also creates adsorption sites with activity different from those of Cu_2_O. A key contribution is the introduction of a descriptor based on the *d*–*p* hybridization state between Co/Cu_2_O and *NO intermediates, which provides atomic-level insights into how heteroatom substitution modulates redox kinetics. DFT calculations reveal that moderate Co substitution optimizes *d*–*p* hybridization by generating unoccupied antibonding (π*) states. This optimized electronic configuration enables two key effects: (i) accelerate interfacial charge transfer; and (ii) balanced *NO adsorption/desorption dynamics that synergistically enhance the redox kinetics. The optimized Co/Cu_2_O catalyst achieves a high FE of 96.5% for NH_3_ and maintains stable performance over 100 h at 100 mA cm^−2^, which is competitive with most reported catalysts. These findings deepen the fundamental understanding of NO_3_RR mechanisms at the atomic scale and establish clear design principles for high-performance electrocatalysts. The integrated theoretical-experimental approach presented here provides a framework for engineering transition-metal-oxide hybrids with catalytic activity for nitrogen-cycle reactions.

## Methods

### Chemicals and reagents

All chemicals were of analytical grade and used as received without further purification. Cr(NO_3_)_3_·9H_2_O, Mn(NO_3_)_2_·4H_2_O, Fe(NO_3_)_3_·9H_2_O, Co(NO_3_)_2_·6H_2_O, Ni(NO_3_)_2_·6H_2_O, Cu(NO_3_)_2_·9H_2_O, KOH, anhydrous ethanol, KNO_3_, NaBH_4_, and HCl were purchased from Sinopharm Chemical Reagent Co., Ltd. Potassium sodium tartrate tetrahydrate, Nessler’s reagent, NaNO_2_, N‑(1‑naphthyl)ethylenediamine dihydrochloride, carbon nanotubes, sulfanilamide, NH_4_Cl‑^15^N, and KNO_3_‑^15^N were obtained from Beijing InnoChem Science & Technology Co., Ltd. Nafion 117 solution (~5 wt%) was acquired from Sigma‑Aldrich. All aqueous solutions were prepared using deionized water (resistivity ≥ 18.2 MΩ·cm).

### Synthesis of Co/Cu_2_O catalyst

Initially, 70 mg of carbon nanotubes were dispersed in 50 mL of ethanol by sonication for 30 min to obtain Solution A. Subsequently, 3.0 mmol Co(NO_3_)_2_·6H_2_O and 5.0 mmol Cu(NO_3_)_2_·9H_2_O were dissolved in 20 mL ethanol under magnetic stirring for 10 min to form Solution B. Solution B was added dropwise to Solution A under vigorous stirring, and the mixture was subsequently stirred for an additional 10 h, yielding Solution C. Next, 1.25 mmol NaBH_4_ in 10 mL deionized water was added dropwise to Solution C while maintaining an ice-water bath. Finally, the precipitates were collected by centrifugation, washed thoroughly with ice-cold ethanol and deionized water, and freeze-dried to obtain the Co/Cu_2_O.

### Synthesis of metal/Cu_2_O catalyst

The same procedure was used to prepare Cr/Cu_2_O, Mn/Cu_2_O, Fe/Cu_2_O, and Ni/Cu_2_O, replacing the Co(NO_3_)_2_·6H_2_O with the corresponding metal nitrate. CoO was prepared using a process similar to that for metal/Cu_2_O, except for the use of Cu(NO_3_)_2_·9H_2_O.

### Physical characterizations

XRD patterns were collected using a Rigaku D/Max-2500 diffractometer with Cu K_α1_ radiation (*λ* = 1.5406 Å). ICP-OES analysis was conducted on an Agilent 7700x instrument to determine the bulk elemental compositions. UV-vis absorption spectra were acquired at room temperature (25 °C) using a Beijing General T6 New Century spectrophotometer. FT-IR measurements were performed on a Nicolet MAGNA-IR 750 spectrometer for the identification of surface functional groups. EPR spectra were collected on a Bruker EMX-8 spectrometer with a microwave frequency of 9.5 GHz at 100 K. Raman spectroscopy was carried out on a Renishaw inVia confocal microscope with a 532 nm excitation laser at a power of 20 mW. SEM (JEOL JSM-6700F) and TEM (FEI Tecnai G20) were both operated at an accelerating voltage of 200 kV for macroscopic and nanoscale observation, respectively. HAADF-STEM was conducted on a JEOL JEM-ARM200F system. XPS data were collected using an ESCALAB 250 spectrometer with a monochromatic Al Kα X-ray source^1^.H NMR spectra were recorded on a Bruker Avance spectrometer at a proton frequency of 600 MHz. Electrolyte pH values were monitored in real time throughout electrochemical tests with a Mettler Toledo LE438 pH electrode, which was calibrated against standard buffer solutions before each measurement. XANES and EXAFS spectra at the Cu and Co K-edges were collected at the BL14W1 beamline of the Shanghai Synchrotron Radiation Facility (SSRF), with beamline energy calibrated against pure Cu and Co metal foils. Online DEMS was performed on a QAS100 system (Linglu Instrument, Shanghai) to track gaseous and volatile intermediates and products in real time.

### In situ FTIR spectroscopy

In situ attenuated total reflection surface‑enhanced infrared absorption spectroscopy (ATR‑SEIRAS) was performed using a Thermo Scientific Nicolet iS50 spectrometer equipped with a liquid‑nitrogen‑cooled MCT detector. The catalyst was deposited onto a silicon prism coated with a gold thin layer (approximately 50 nm thick), which served as the working electrode. A Pt mesh and an Ag/AgCl (saturated KCl) electrode were used as the counter and reference electrodes, respectively. The electrolyte was 0.1 M KOH containing 0.1 M KNO_3_. Spectra were collected over a potential range from −0.454 V to −1.564 V *vs*. Ag/AgCl, with a hold time of approximately 5 min at each potential to reach a steady state. The spectrum collected at open‑circuit potential (OCP) was used as the background reference. All reported spectra were obtained after subtracting the background. All potentials were converted to the RHE scale using the Nernst equation: *E*_RHE_ = *E*_Ag/AgCl_ + 0.0591 pH + 0.197 V.

### In situ Raman spectroscopy

In situ Raman measurements were carried out in a custom‑built two‑compartment three‑electrode electrochemical cell equipped with a quartz optical window. A glassy carbon electrode (GCE, 8 mm diameter) was polished with 0.05 µm alumina suspension and rinsed with deionized water prior to use. The catalyst suspension (10 µL, 5 mg mL^−1^) was drop‑cast onto the GCE and dried at 25 °C, yielding a catalyst loading of approximately 0.2 mg cm^−2^. A Pt mesh served as the counter electrode, and an Ag/AgCl (saturated KCl) electrode was used as the reference. The electrode potential was controlled using a CHI 760E electrochemical workstation. Raman spectra were acquired on Raman microscope (Zhongke Kaili Instrument Equipment (Suzhou) Co., Ltd) using a 532 nm laser excitation, a 50× objective (NA = 0.55), and a CCD detector. The laser power was maintained at 50% of the maximum output (~5 mW) to avoid laser‑induced heating or photodegradation of the catalyst. The Raman frequency was calibrated against the Si phonon band at 520.7 cm^−1^ before each measurement. Each spectrum was collected with an acquisition time of 40 s, and the signal was averaged over three accumulations to improve the signal‑to‑noise ratio.

### In situ differential electrochemical mass spectroscopy (DEMS)

On-line DEMS (QAS100, Shanghai Linglu Instrument) combined with an electrochemical workstation (CHI 760E) was employed in a typical three-electrode electrochemical cell (Fig. S70). A calibrated Ag/AgCl and platinum wire were used as the reference and counter electrodes, respectively. And an Au film sputtered on a porous PTFE membrane was used as the working electrode, where the catalyst was sprayed on the Au film and then dried. The hydrophobic PTFE membrane (Cobetter, PF-002HS) permits gas flow while rejecting liquid. To trap the water vapor for averting potential damages to the mass spectrometer, a cold trap cooled with dry ice was installed between the electrochemical cell and the vacuum chamber. 0.1 M KOH solution with 0.1 M KNO_3_ was used as the electrolyte. Five consecutive CV cycles were conducted with a scan rate of 10 mV s^−1^ over a potential range of −0.454 V to −1.564 V *vs*. Ag/AgCl, while recording the mass signals *m*/*z* of different volatile products.

### In situ X‑ray absorption fine structure (XAFS) spectroscopy

Prior to in-situ XAFS measurements, the working electrode was pre-conditioned by cyclic voltammetry (10 cycles) from −0.454 V to −1.564 V *vs*. Ag/AgCl at a scan rate of 10 mV s^−1^ in 0.1 M KOH + 0.1 M KNO_3_. The catalyst was hand-brushed onto a carbon paper substrate (1 × 2 cm^2^) with a loading of approximately 2.0 mg cm^−2^. In situ Co K-edge XANES and EXAFS spectra were collected at the BL14W1 beamline of the Shanghai Synchrotron Radiation Facility (SSRF). A Si(111) double-crystal monochromator was used for energy selection. Data were acquired in fluorescence mode using a Lytle detector for catalyst samples and in transmission mode for reference standards (Co foil). The energy was calibrated using the first inflection point of a Co foil spectrum, measured simultaneously with each sample. Spectra were recorded at three representative potentials: OCP, −0.454 V, and −1.564 V *vs*. Ag/AgCl. At each potential, the electrode was equilibrated for 30 min before spectrum acquisition to ensure a steady state. Each spectrum was the average of three consecutive scans. No error bars are shown for the XAFS data because they represent single measurements at each condition.

### ^15^N isotope-labeling experiment

To confirm the nitrogen source of the produced NH_3_ isotope-labeling experiments were conducted using 0.1 M KOH containing 0.1 M K^15^NO_3_ as the electrolyte. Electrolysis was performed at −0.3 V *vs*. RHE for 1 h in a standard H‑type electrochemical cell. After electrolysis, the electrolyte was collected and analyzed by ^1^H NMR spectroscopy. The ^15^${{{{\rm{NH}}}}}_{4}^{+}$ in the electrolyte was quantified using the same procedure as that for ^14^${{{{\rm{NH}}}}}_{4}^{+}$. All NMR spectra were recorded on a Bruker Avance instrument at 600 MHz with water suppression.

### Electrocatalytic measurement

Electrochemical characterization was performed on a CHI 760E workstation (CH Instruments, USA) under ambient conditions (25 °C). A three-electrode H-type cell configuration was adopted, comprising a catalyst-coated working electrode, an Ag/AgCl (saturated KCl) reference electrode, and a carbon rod counter electrode. The cathodic and anodic compartments (40 mL) were separated by a Nafion-117 membrane, with either 0.1 M KOH or 0.1 M KNO_3_ + 0.1 M KOH serving as the electrolyte. The pH of the 0.1 M KOH solution was determined to be 13.0 ± 0.1 from three independent measurements using a pre-calibrated pH meter. Unless otherwise noted, all potentials are reported without iR compensation.

For chronoamperometric tests, working electrodes were fabricated by loading catalyst ink onto carbon paper (1 × 1 cm^2^), with a catalyst loading of 1 mg per electrode. The ink was formulated by dispersing 5 mg of catalyst powder in a mixture of 450 μL deionized water, 450 μL isopropyl alcohol, and 100 μL Nafion solution under ultrasonication until homogeneous. An aliquot of 200 μL of the resulting suspension was then drop-cast onto the carbon paper substrate and air-dried at room temperature. Current densities are normalized to catalyst mass, geometric electrode area, or ECSA as indicated in each figure caption.

All electrochemical measurements, except for long-term stability tests, were performed in triplicate using independently prepared electrodes and fresh electrolyte to ensure reproducibility; results are reported as mean ± standard deviation. Potentials were converted to the RHE scale using:

1 $${E}_{{{{\rm{RHE}}}}}={E}_{{{{\rm{Ag}}}}/{{{\rm{AgCl}}}}}+0.197{{{\rm{V}}}}+0.0591\times {{{\rm{pH}}}}$$

where *E*_Ag/AgCl_ refers to the potential directly measured against the Ag/AgCl reference electrode.

ECSA was derived from the double-layer capacitance (*C*_*dl*_), which was determined by CV in the non-Faradaic potential window of 0.1 M KOH electrolyte at scan rates 10–100 mV s^−1^. The charging current density (*I*_*c*_) was plotted against scan rate (*ν*), and *C*_*dl*_ was obtained from the slope:

2 $${I}_{C}=v\times {C}_{{dl}}$$

3 $${{{\rm{ECSA}}}}=\frac{{C}_{{dl}}}{{C}_{s}}$$

where *C*_*s*_ is the specific capacitance of flat metallic surface.

LSV was conducted on a RDE with a catalyst loading of 40 μg on the glassy carbon disk, at rotation speeds of 100–1600 rpm and a scan rate of 5 mV s^−1^ under Ar flow. The electrode surface was conditioned with 50 CV cycles prior to each measurement, and all reported LSV curves were collected after five pre-scans to achieve a stable baseline.

The Faradaic efficiency (FE) for NH_3_ and ${{{{\rm{NO}}}}}_{2}^{-}$ production was calculated using the following equations:

4 $${{{{\rm{FE}}}}}_{{{{{\rm{NH}}}}}_{3}}=\frac{{Q}_{{{{{\rm{NH}}}}}_{3}}}{Q}=\frac{{n}_{{{{{\rm{NH}}}}}_{3}}\times V\times {c}_{{{{{\rm{NH}}}}}_{3}}\times F}{Q}\,=\,\frac{8\times F\times \,{c}_{{{{{\rm{NH}}}}}_{3}}\times V}{17\,\times \,Q}$$

5 $${{{{\rm{FE}}}}}_{{{{{\rm{NO}}}}}_{2}^{-}}=\frac{{Q}_{{{NO}}_{2}^{-}}}{Q}=\frac{{n}_{{{NO}}_{2}^{-}}\times V\times {c}_{{{NO}}_{2}^{-}}\times F}{Q}=\frac{2\times F\times {c}_{{{NO}}_{2}^{-}}\times V}{46\,\times \,Q}$$

In these equations, *Q* stands for the total charge consumed during electrolysis (Coulomb), *F* denotes the Faraday constant (96,485 C mol^−1^), ${c}_{{{NH}}_{3}}$ and ${c}_{{{NO}}_{2}^{-}}$ represent the measured concentrations of NH_3_ and ${{{{\rm{NO}}}}}_{2}^{-}$ in the catholyte (mol L^−1^), and *V* refers to the volume of catholyte (40 mL).

The corresponding product yield rate (*r*) was determined by:

6 $$r=\frac{C\times V}{t\times m}$$

where *C* is the measured product concentration, *t* represents the duration of electrolysis, and *m* is the mass of catalyst loaded on the working electrode.

To evaluate the scalability of the system, chronopotentiometric tests were performed in an enlarged H-type cell (100 mL) with carbon paper (1 × 1 cm^2^) loaded with 2 mg of catalyst as the working electrode, a platinum mesh (1.5 × 2 cm^2^) as the counter electrode, and an Ag/AgCl (saturated KCl) reference electrode. All other conditions were consistent with the chronoamperometric experiments.

### DFT calculations

First-principles calculations were carried out using the Vienna ab initio Simulation Package (VASP)^51^. The exchange-correlation functional was treated with the Perdew-Burke-Ernzerhof (PBE) formulation within the generalized gradient approximation (GGA). Core-valence interactions were described by the projector augmented wave (PAW) method^52^. A plane-wave energy cutoff of 450 eV was applied throughout^53^.

Geometry optimizations were considered converged when the residual forces on each atom fell below 0.02 eV/Å^54^, and the energy change between consecutive steps was less than 10^−6^ eV^55^. A vacuum layer of 20 Å was introduced along the surface normal to avoid spurious interactions between periodic replicas^56,57^. Van der Waals corrections were incorporated via Grimme’s D3 scheme with Becke-Johnson damping. The reliability of the computational protocol was verified through systematic convergence tests and comparison with experimental benchmarks.

The Gibbs free energy change (ΔG) for each elementary step was obtained from^58^:

7 $$\Delta {G}^{*}=\Delta E+\Delta {E}_{{ZPE}}-T\times \Delta S+\Delta {U}_{0\to 300{{{\rm{K}}}}}$$

where ∆*E* is the DFT-derived reaction energy, ∆*E*_ZPE_ is the zero-point energy correction, ∆*S* is the entropy change, ∆*U*_0→300K_ is the thermal correction (excluding ZPE), and T is the temperature (300 K).

To properly describe electron correlation in the 3*d* orbitals of transition-metal ions, the GGA + U method was employed with effective Hubbard parameters (U_eff_) of 5.0 eV (Cu 3*d*), 3.3 eV (Co 3*d*), and 6.0 eV (Ni 3*d*), consistent with literature values^59–62^. The DFT-D3 dispersion correction was applied uniformly to all models.

## Supplementary information

Supplementary Information

Description of Additional Supplementary Files

Supplementary Data 1

Transparent Peer Review file

## Source data

Source Data 1

Source Data 2

## Data Availability

The Source data file, containing all numerical data for the main and Supplementary figures, is provided with this paper. The atomic coordinates of the DFT structures are available as Supplementary Data. Source data are provided with this paper.
