# Supplementary material for: Spin state and orbital engineering of cuprous oxide for efficient nitrate reduction
Source: Nat Commun. 2026 Aug 15;17:9827. doi: 10.1038/s41467-026-76420-8 (PMC13575217; doi:10.1038/s41467-026-76420-8)
Supplement: Supplementary file 1 [file 41467_2026_76420_MOESM1_ESM.pdf]

Supplementary Information

Spin State and Orbital Engineering of Cuprous Oxide for Efficient Nitrate Reduction

Huicong Xia[1,2,#], Haihui Lan[3,#], Yue Yu[1], Yifan Wei[1], Zixin Li[1], Renqin Yu[4], Siran Xu[1], Yunchuan Tu[4], Mingkai Liu[5,*], and Jia-Nan Zhang[1,2,*]

1 College of Materials Science and Engineering, Zhengzhou University, Zhengzhou 450001, P. R. China.

2 College of Chemistry, Zhengzhou University, Laboratory of Pingyuan, Zhengzhou 450001, P. R. China.

3 Department of Chemistry, Massachusetts Institute of Technology, Cambridge 02139, United States.

4 School of Chemistry and Chemical Engineering, Chongqing University, Chongqing 401331, P. R. China.

5 School of Chemistry and Chemical Engineering, Anhui University of Technology, Ma'anshan, 243032, P. R. China.

[#]H. Xia and H. Lan contributed equally to this work.

[*]Corresponding authors: liumingkai@ahut.edu.cn; zjn@zzu.edu.cn

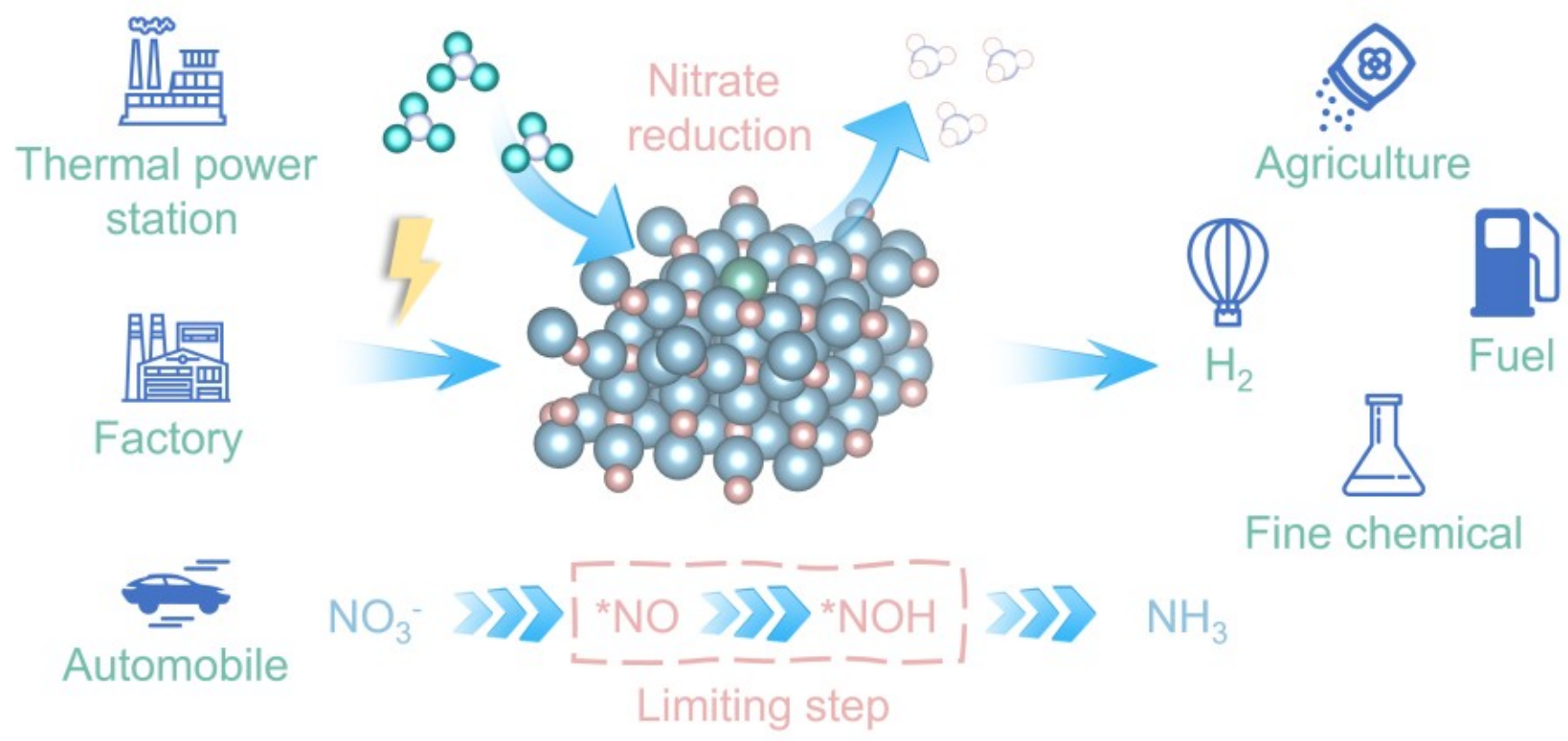

Supplementary Figure 1. Schematic illustration of the electrochemical conversion of nitrate to ammonia driven by renewable electricity under ambient conditions.

This schematic depicts a sustainable electrochemical strategy for ambient nitrate-to-ammonia conversion powered by renewable electricity. It converts nitrate pollutants from industrial and municipal sources into value-added ammonia, with the *NO to *NOH hydrogenation identified as the rate-determining step. This approach enables simultaneous environmental remediation and green chemical production.

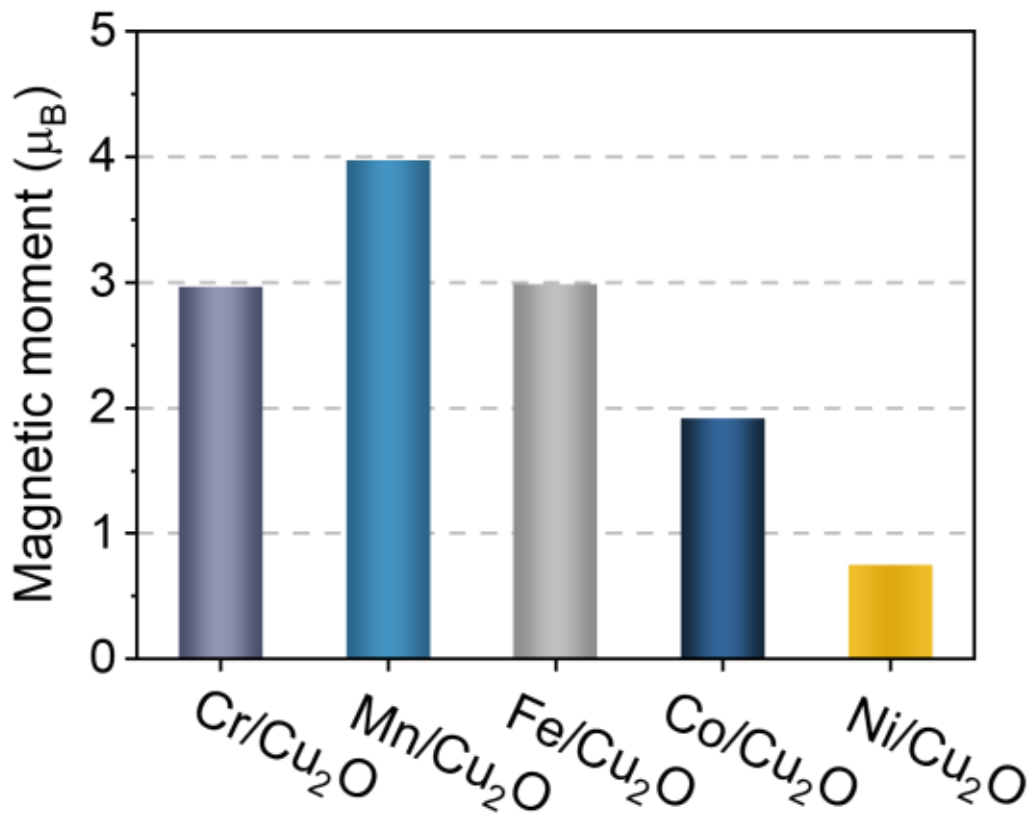

Supplementary Figure 2. Theoretically calculated effective magnetic moments of transition metal-doped Cu_2O catalyst systems. Source data are provided as a Source Data file.

This figure presents the theoretically calculated effective magnetic moments of various M/Cu_2O catalysts. Mn/Cu_2O exhibits the highest magnetic moment (~4.0 μ_B), while Ni/Cu_2O shows the lowest value (~0.7 μ_B). These variations directly reflect the number of unpaired 3*d* electrons in doped metals, which serve as a critical electronic descriptor for their nitrate reduction catalytic activity.

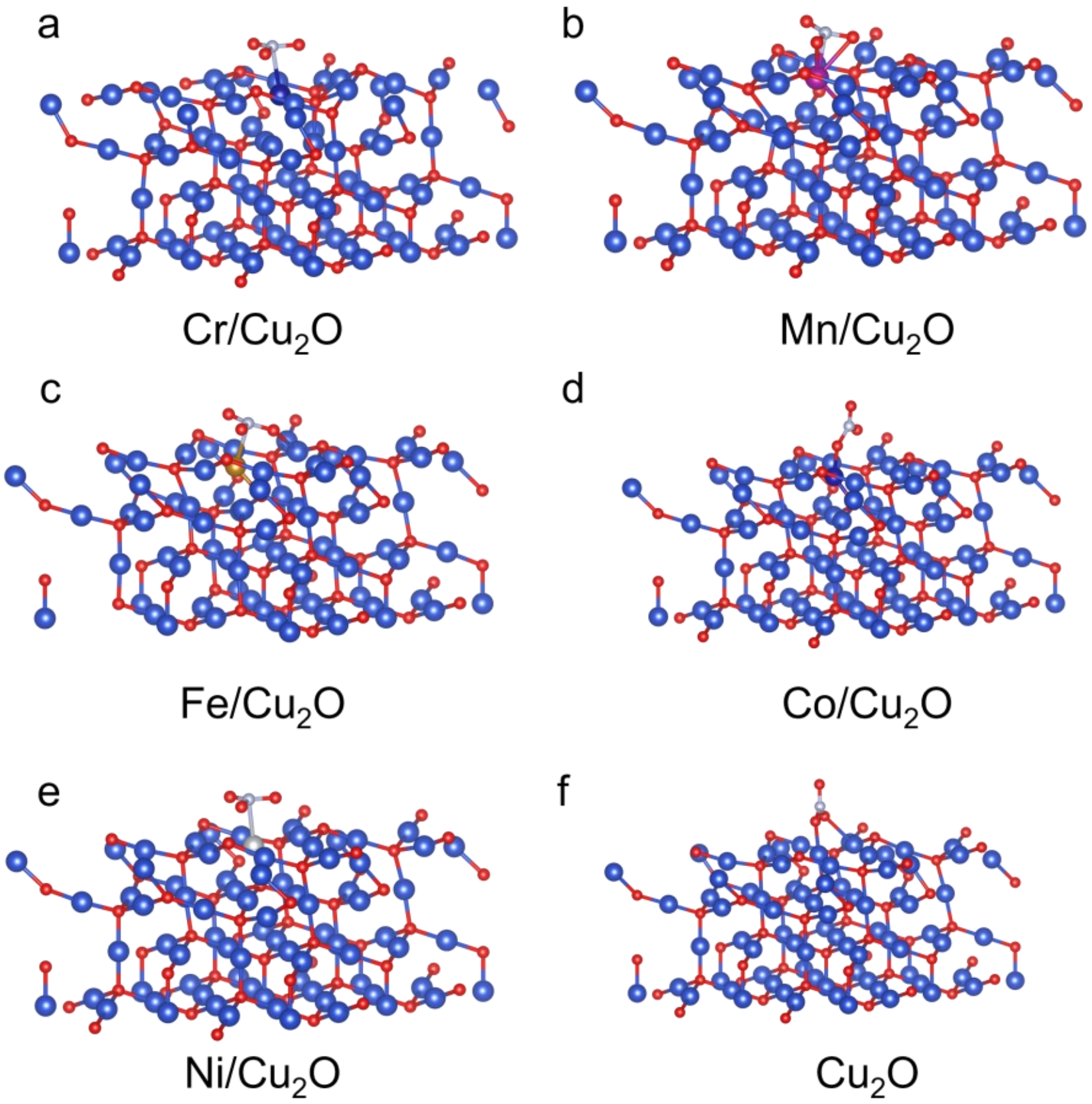

Supplementary Figure 3. Optimized geometric configurations of NO_3 intermediate adsorbed on transition metal-doped Cu_2O and pristine Cu_2O model surfaces. All atomic structures are obtained after full geometric relaxation based on density functional theory calculations. Blue spheres represent copper atoms from the Cu_2O substrate, red spheres represent oxygen atoms from both the oxide lattice and the adsorbed nitrate intermediate. The doped transition metal cation at the adsorption site is marked with a distinct colored sphere in each doped system. Side view of the $*NO_3$ adsorption structure on the (a) Cr/Cu_2O, (b) Mn/Cu_2O, (c) Fe/Cu_2O (d) Co/Cu_2O, (e) Ni/Cu_2O, and (f) Cu_2O surface.

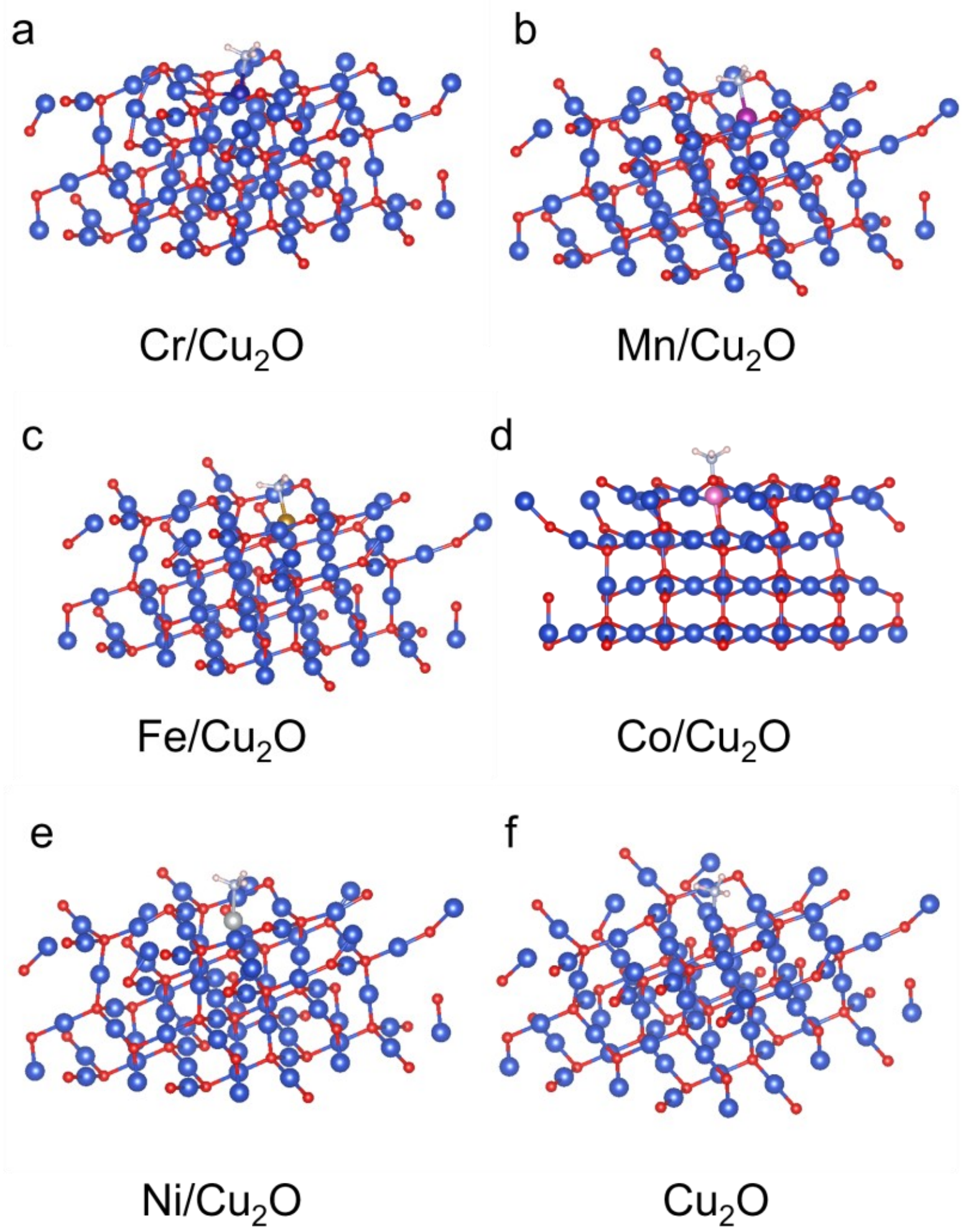

Supplementary Figure 4. Optimized geometric configurations of the adsorbed NH_3 intermediate on transition metal-doped Cu_2O and pristine Cu_2O model surfaces. All atomic structures are obtained after full geometric relaxation based on density functional theory calculations, and all panels are presented in side view. Blue spheres represent copper atoms from the Cu_2O substrate; red spheres represent oxygen atoms from the oxide lattice. Within the adsorbed $*NH_3$ species, light gray spheres correspond to nitrogen atoms and small white spheres correspond to hydrogen atoms. The transition metal dopant located at the adsorption site is marked with a distinct colored sphere in each doped system. Relaxed $* NH_3$ adsorption structure on the (a) Cr/Cu_2O, (b) Mn/Cu_2O, (c) Fe/Cu_2O, (d) Co/Cu_2O, (e) Ni/Cu_2O, and (f) Cu_2O surface.

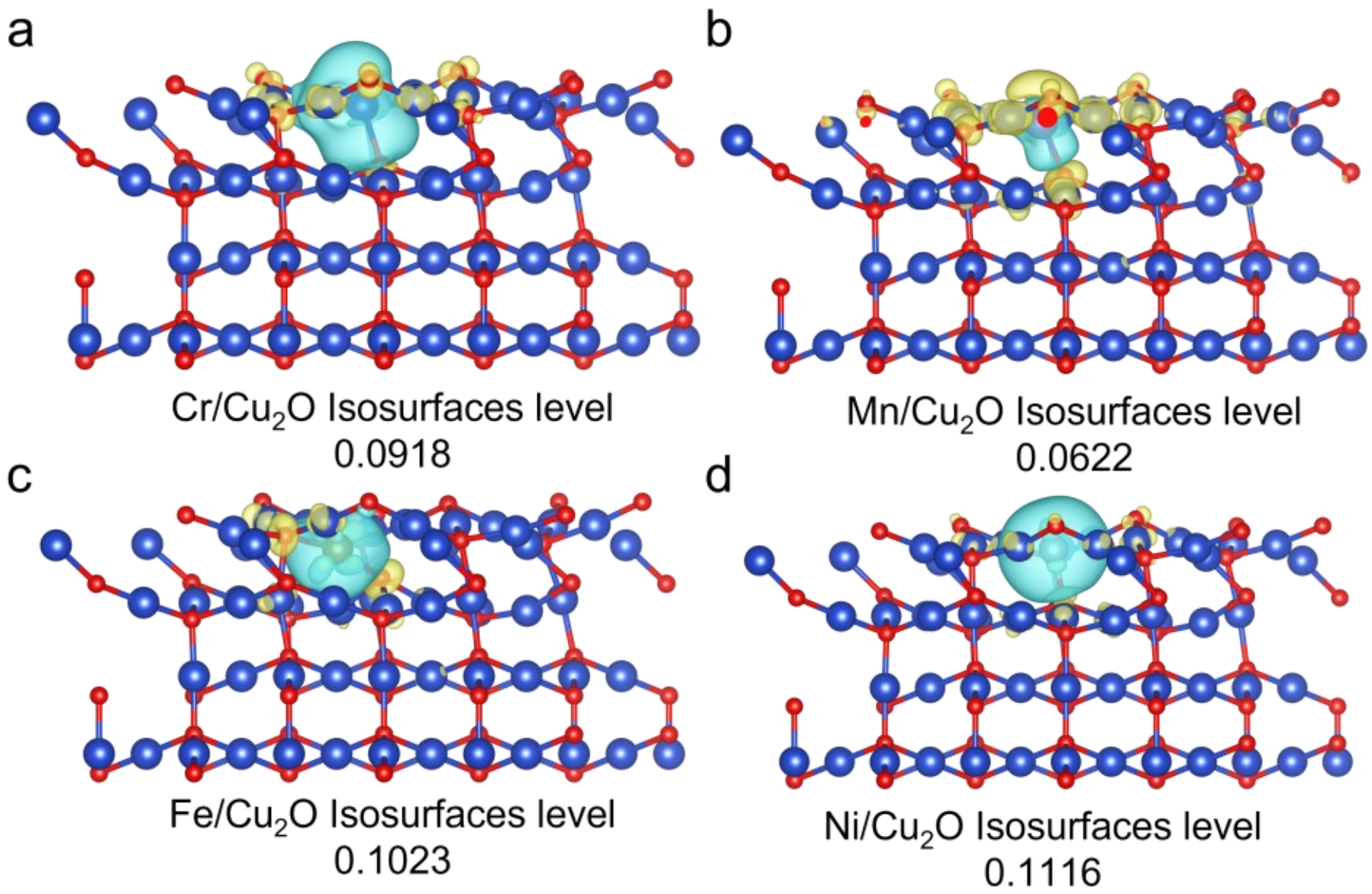

Supplementary Figure 5. Differential spin density distributions of transition metal-doped Cu_2O model systems. All atomic structures are derived from density functional theory calculations and displayed in side view. The differential spin density describes the spatial redistribution of spin-polarized electrons introduced by the incorporation of transition metal dopants into the Cu_2O lattice. Cyan isosurfaces correspond to regions with net spin density accumulation, while yellow isosurfaces correspond to regions with net spin density depletion. Blue spheres represent copper atoms from the Cu_2O host lattice, and red spheres represent lattice oxygen atoms. The numerical value marked under each panel indicates the isosurface level applied for visualization. Differential spin density map of the (a) Cr/Cu_2O, (b) Mn/Cu_2O, (c) Fe/Cu_2O, and (d) Ni/Cu_2O model system.

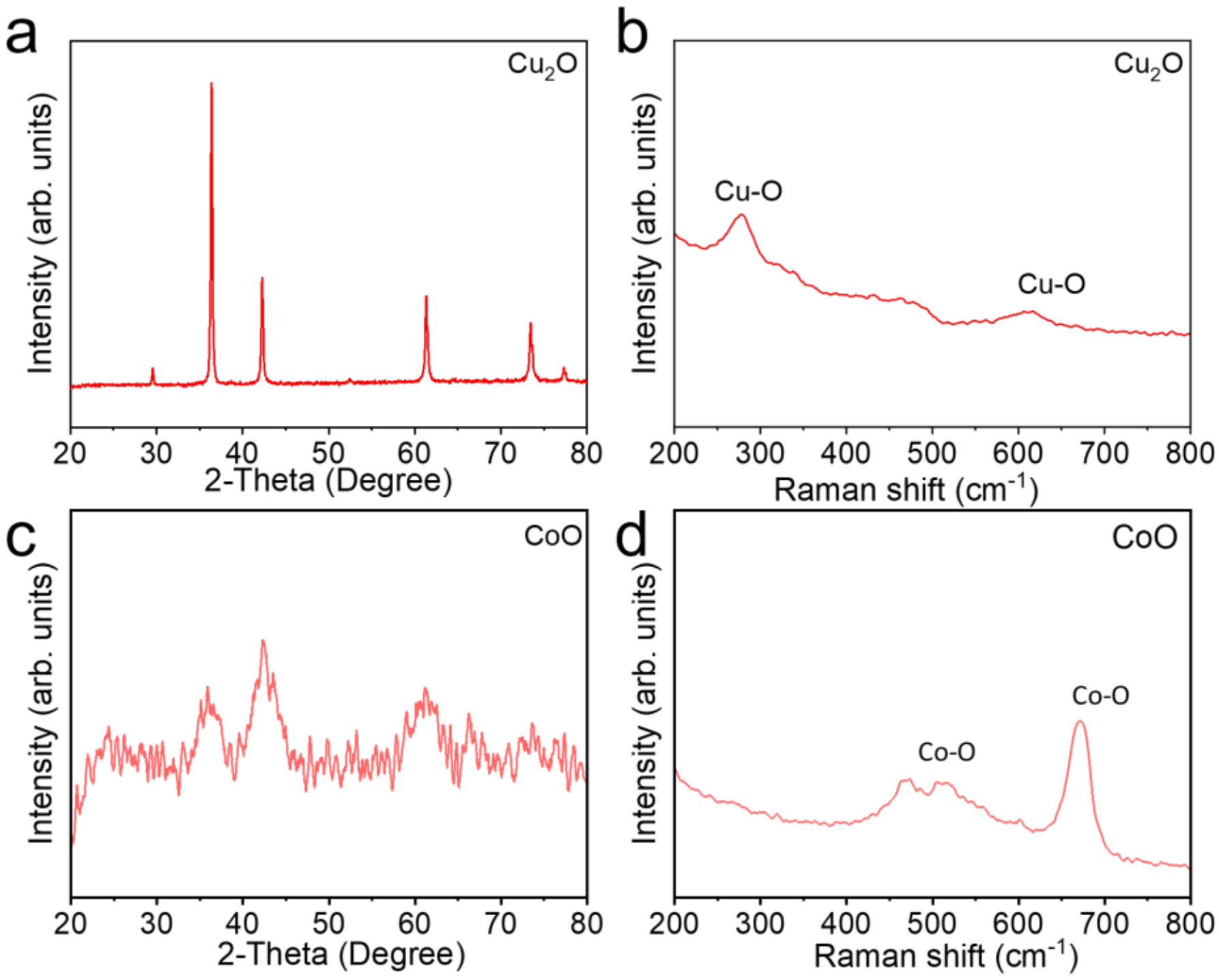

Supplementary Figure 6. Phase and structural characterization. (a) X-ray diffraction (XRD) pattern of the as-prepared Cu_2O sample. (b) Raman spectrum of the Cu_2O sample. (c) XRD pattern of the CoO reference sample. (d) Raman spectrum of the CoO reference sample. Source data are provided as a Source Data file.

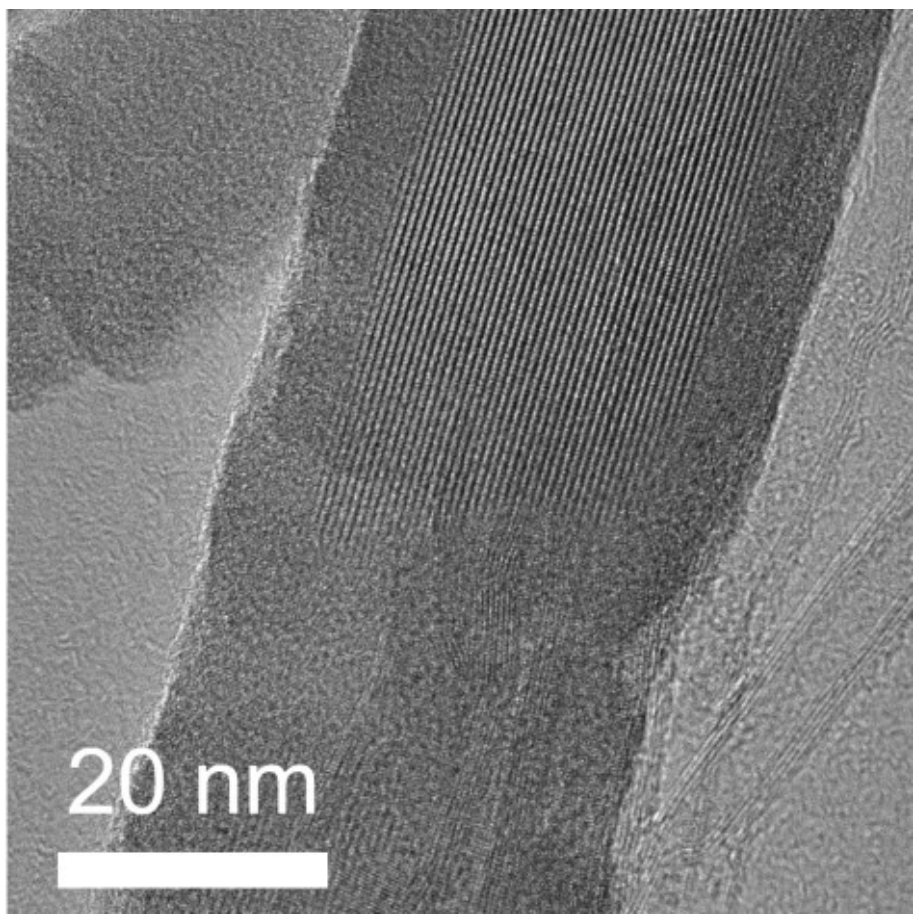

Supplementary Figure 7. Low-magnification transmission electron microscopy image of the Co/Cu_2O.

This figure shows the high-resolution TEM image of Co/Cu_2O. The well-defined continuous lattice fringes confirm the high crystallinity of the Cu_2O support. Co atoms are homogeneously doped into the Cu_2O lattice by substituting Cu sites without forming segregated secondary phases, providing abundant isolated active sites for nitrate electroreduction.

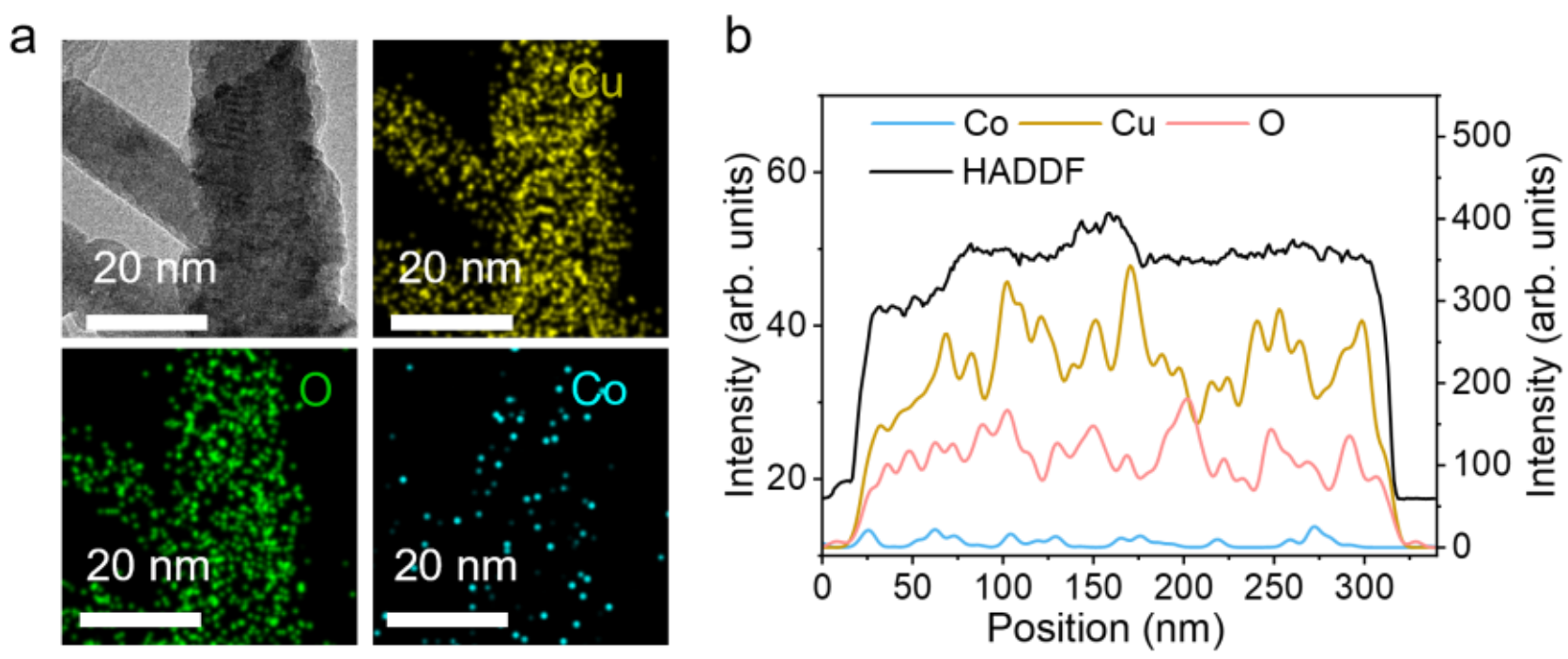

Supplementary Figure 8. Elemental distribution characterization of the Co/Cu_2O sample. (a) High-angle annular dark-field scanning transmission electron microscopy (HAADF-STEM) image and corresponding energy-dispersive X-ray spectroscopy (EDS) elemental mapping images of Co/Cu_2O. (b) EDS elemental line scanning profiles acquired across the cross-section of a Co/Cu_2O particle. The horizontal axis represents the scanning position in nanometers. The black curve corresponds to the HAADF intensity, while the yellow, red and blue curves represent the characteristic signal intensities of copper, oxygen and cobalt elements, respectively, showing the concentration variation of each element along the scanning path. Source data are provided as a Source Data file.

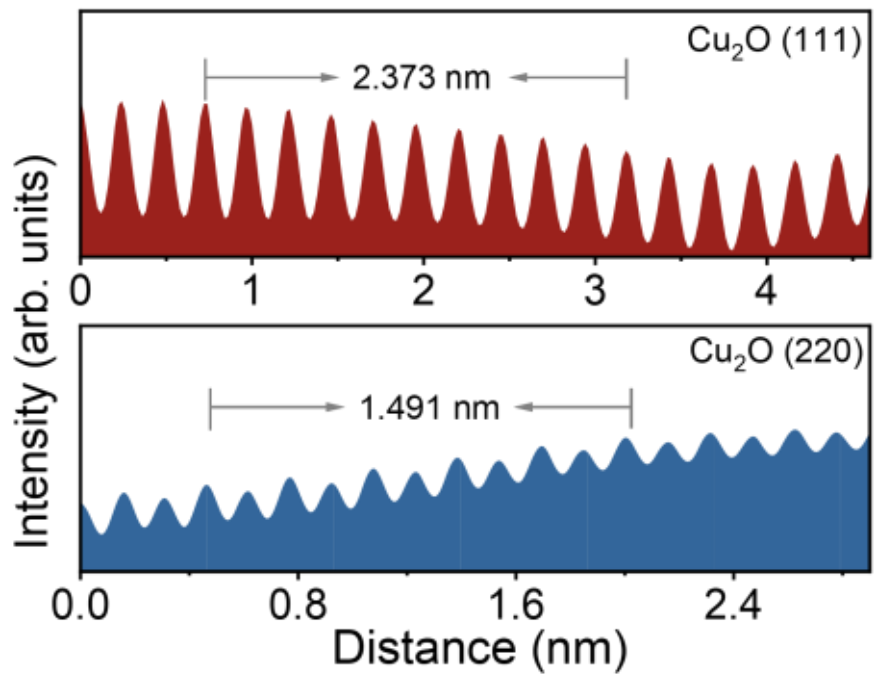

Supplementary Figure 9. Atomic intensity line-scanning analysis derived from the HAADF-STEM image presented in Figure 1c. Source data are provided as a Source Data file.

This figure presents the atomic intensity line scanning profiles extracted from the high-resolution TEM image. The measured lattice spacings of 2.373 nm and 1.491 nm correspond perfectly to the (111) and (220) crystal planes of cubic Cu_2O, respectively, confirming the high crystallinity and pure phase of the synthesized support.

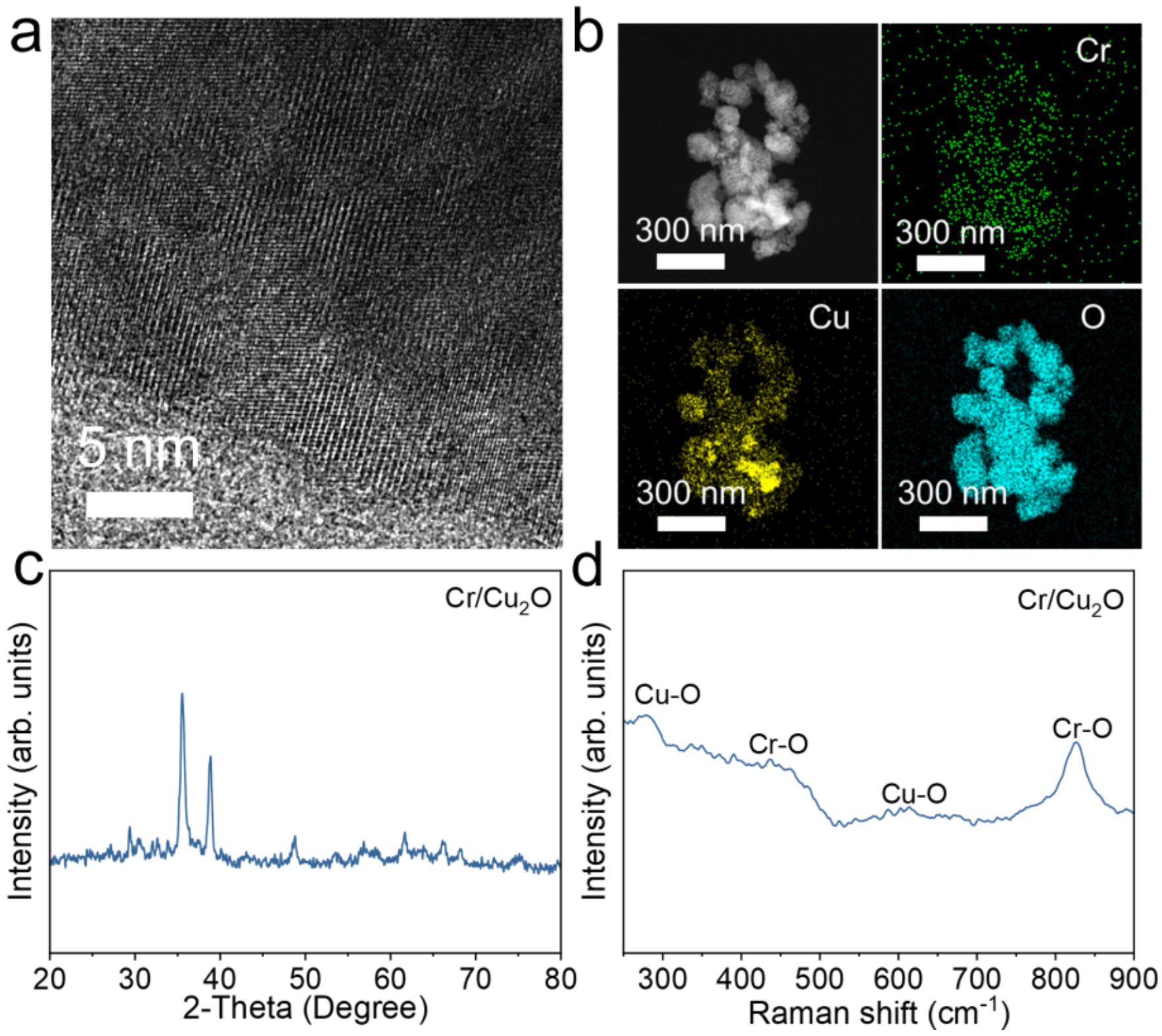

Supplementary Figure 10. Morphological, structural and phase characterization. (a) High-resolution transmission electron microscopy (HRTEM) image of the as-prepared Cr/Cu_2O sample. (b) HAADF-STEM morphological image and corresponding energy-dispersive X-ray spectroscopy (EDS) elemental mapping images of Cr/Cu_2O. (c) X-ray diffraction (XRD) pattern and (d) Raman spectrum of Cr/Cu_2O. Source data are provided as a Source Data file.

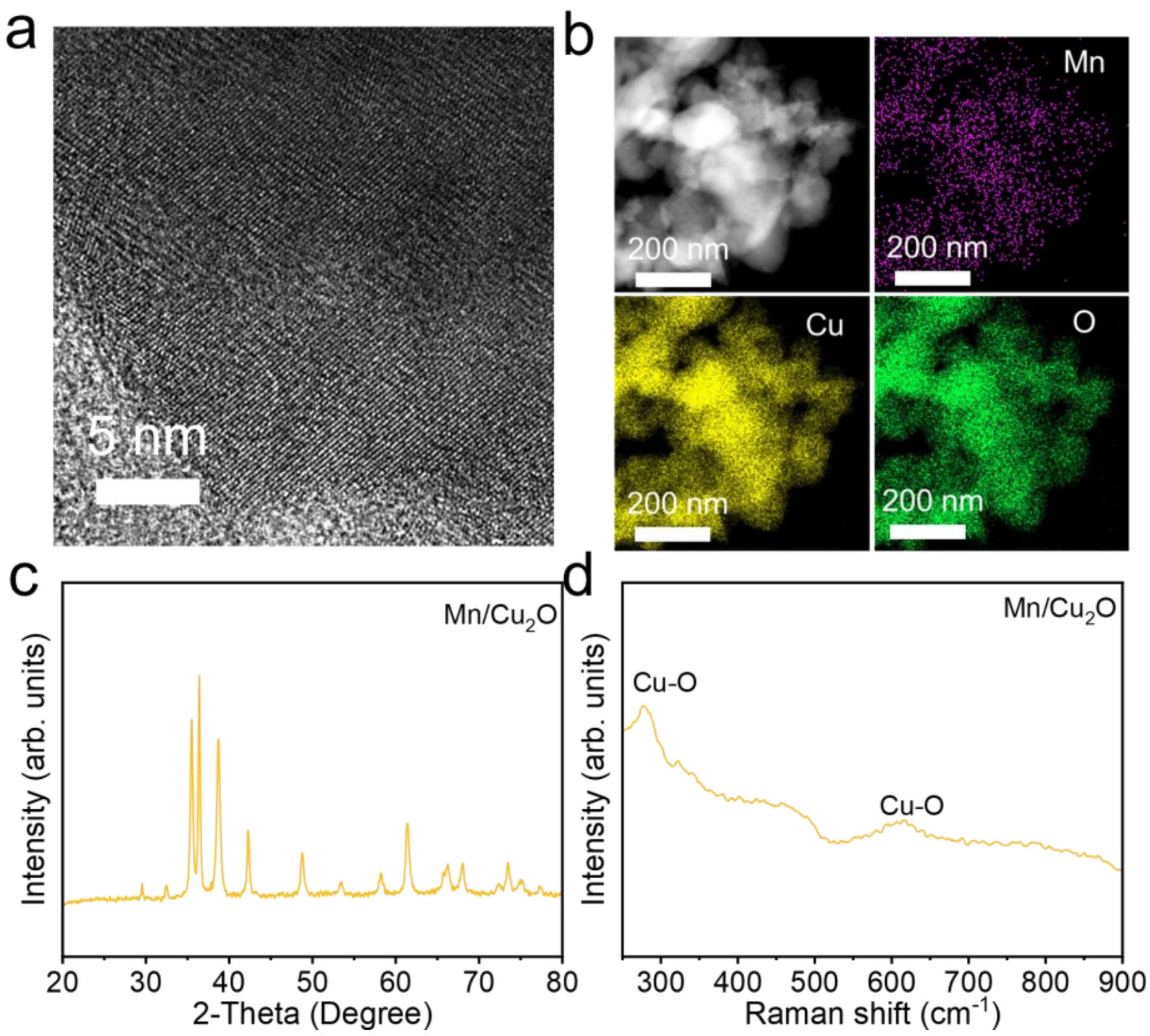

Supplementary Figure 11. Morphological, structural and phase characterization. (a) High-resolution transmission electron microscopy (HRTEM) image of the as-prepared Mn/Cu_2O sample. (b) HAADF-STEM morphological image and corresponding energy-dispersive X-ray spectroscopy (EDS) elemental mapping images of Mn/Cu_2O. (c) X-ray diffraction (XRD) pattern and (d) Raman spectrum of Mn/Cu_2O. Source data are provided as a Source Data file.

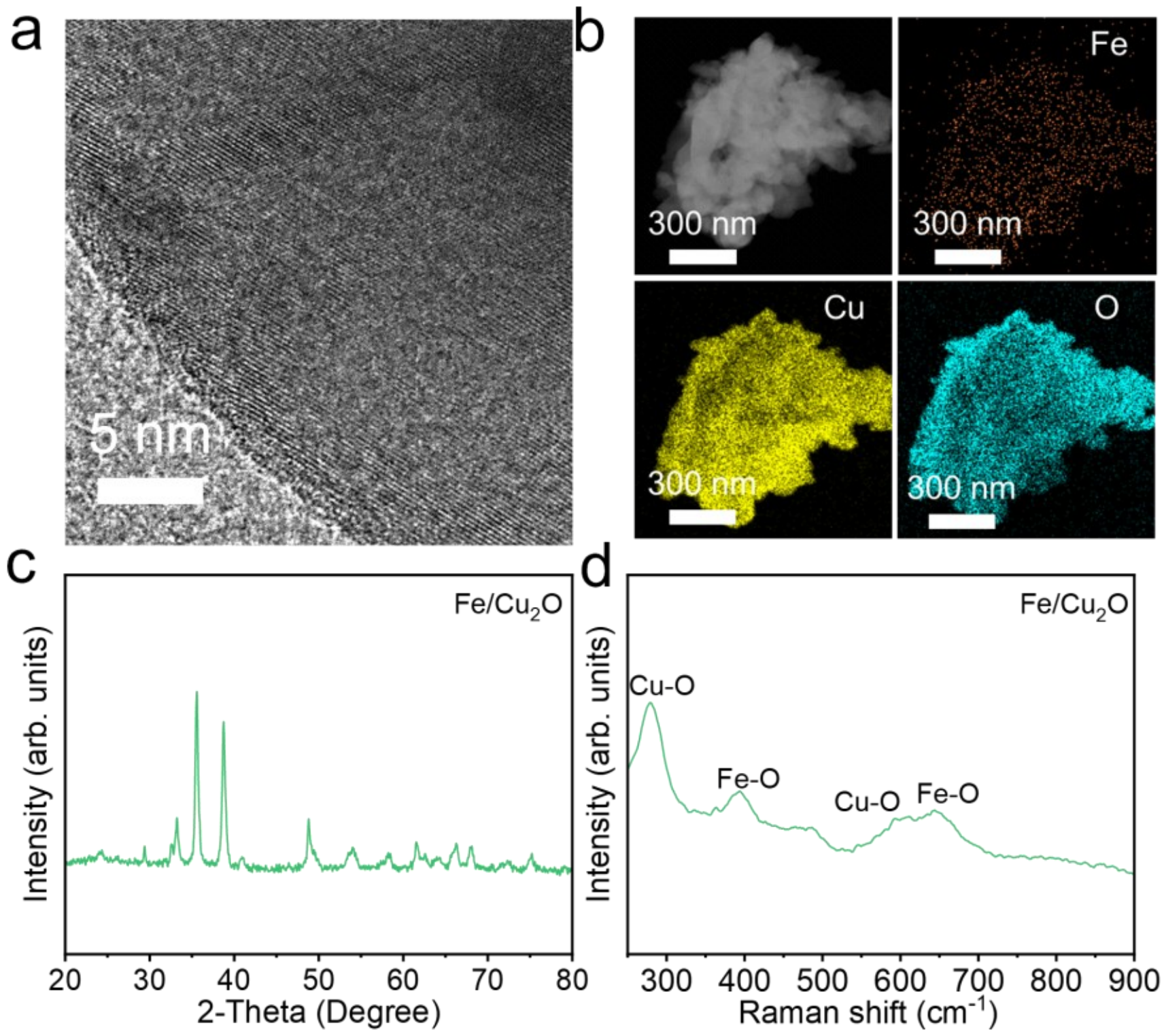

Supplementary Figure 12. Morphological, structural and phase characterization. (a) High-resolution transmission electron microscopy (HRTEM) image of the as-prepared Fe/Cu_2O sample. (b) HAADF-STEM morphological image and corresponding energy-dispersive X-ray spectroscopy (EDS) elemental mapping images of Fe/Cu_2O. (c) X-ray diffraction (XRD) pattern and (d) Raman spectrum of Fe/Cu_2O. Source data are provided as a Source Data file.

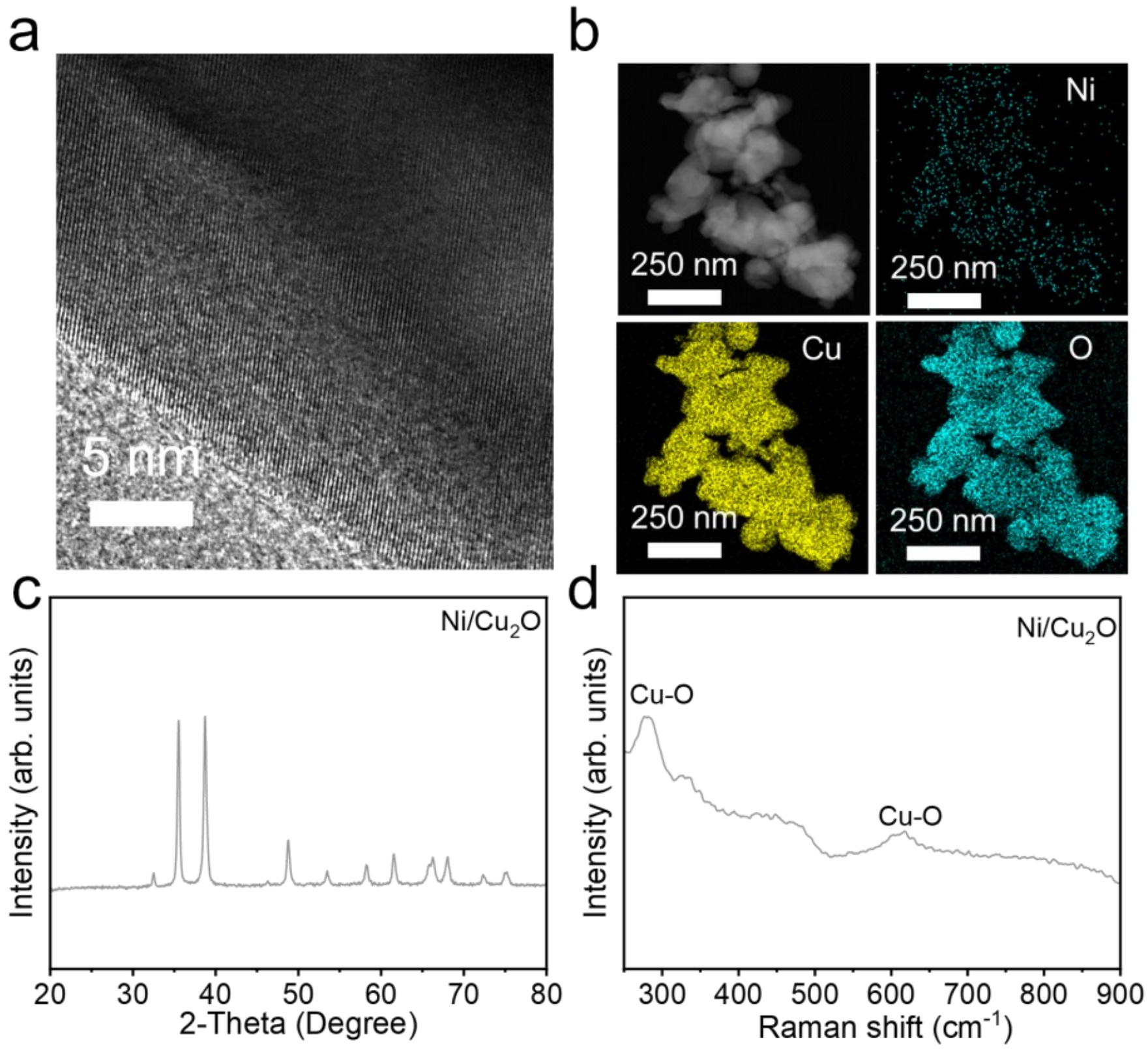

Supplementary Figure 13. Morphological, structural and phase characterization. (a) High-resolution transmission electron microscopy (HRTEM) image of the as-prepared Ni/Cu_2O sample. (b) HAADF-STEM morphological image and corresponding energy-dispersive X-ray spectroscopy (EDS) elemental mapping images of Ni/Cu_2O. (c) X-ray diffraction (XRD) pattern and (d) Raman spectrum of Ni/Cu_2O. Source data are provided as a Source Data file.

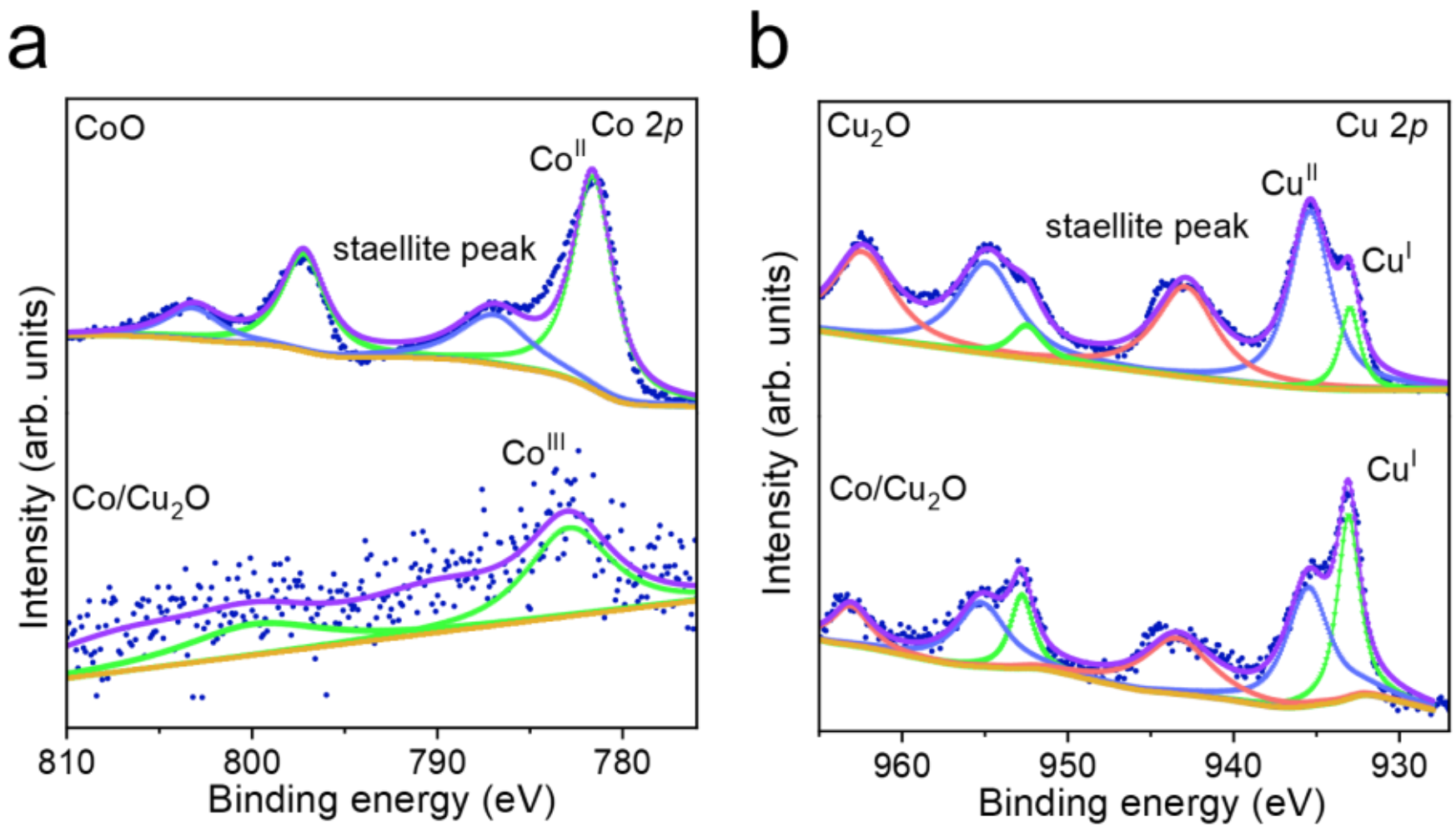

Supplementary Figure 14. High-resolution X-ray photoelectron spectroscopy analysis. (a) Co 2*p* core-level X-ray photoelectron spectroscopy (XPS) spectra of the CoO reference sample (upper panel) and the Co/Cu_2O sample (lower panel). (b) Cu 2*p* core-level XPS spectra of the pristine Cu_2O sample (upper panel) and the Co/Cu_2O sample (lower panel).

This figure presents the XPS spectra of Co 2*p* and Cu 2*p*. Co in pure CoO exists predominantly as Co^{II} with distinct satellite peaks, while Co in Co/Cu_2O is mainly Co^{III}. Compared with pure Cu_2O, Co/Cu_2O shows significantly reduced Cu^{II} content and dominant Cu^{I} species, indicating electron transfer from Co to Cu that modulates the electronic structure of active sites. Source data are provided as a Source Data file.

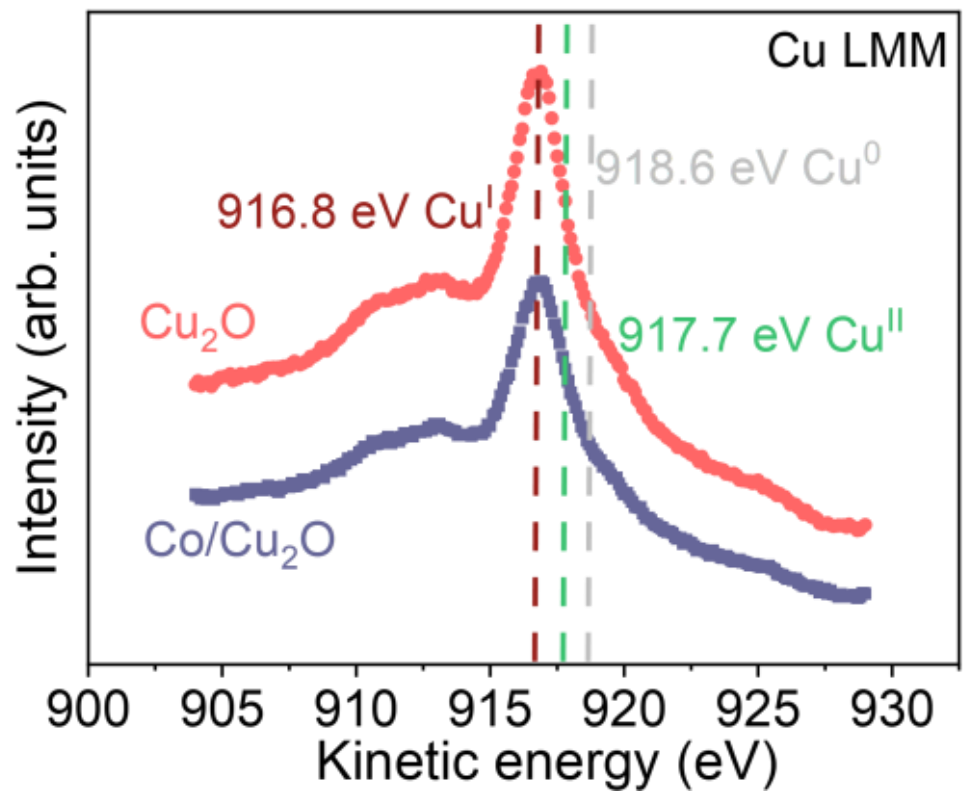

Supplementary Figure 15. Cu LMM Auger electron spectra for surface copper valence state analysis of Cu_2O and Co/Cu_2O catalysts. The red curve corresponds to the Cu LMM Auger electron spectrum of the pristine Cu_2O sample, and the blue curve corresponds to that of the Co/Cu_2O catalyst. Source data are provided as a Source Data file.

Cu LMM Auger electron spectroscopy is utilized to resolve Cu chemical states with superior precision relative to conventional Cu 2*p* XPS, as Auger transitions exhibit substantially larger chemical shifts, enabling unambiguous differentiation of Cu^0, Cu^I, and Cu^{II} species. As shown in the spectra, both pristine Cu_2O and Co/Cu_2O exhibit a dominant Auger peak at ~916.8 eV assigned to monovalent Cu^I, confirming that the Cu_2O matrix remains the primary phase after Co incorporation.

No discernible signal at 917.7 eV, the characteristic kinetic energy of divalent Cu^{II}, is detected in either sample, ruling out the presence of CuO or other Cu^{II}-containing impurities. A minor high-energy shoulder corresponding to metallic Cu^0 (~918.6 eV) appears on both surfaces, arising from mild spontaneous reduction of surface Cu^I under ambient exposure. Notably, Co/Cu_2O shows a reduced relative Cu^0 content compared with pure Cu_2O, indicating that lattice Co doping stabilizes the Cu^I oxidation state via electronic metal-support interactions. This preserved Cu^I-rich surface optimizes reaction intermediate binding, contributing to enhanced NO_3RR activity.

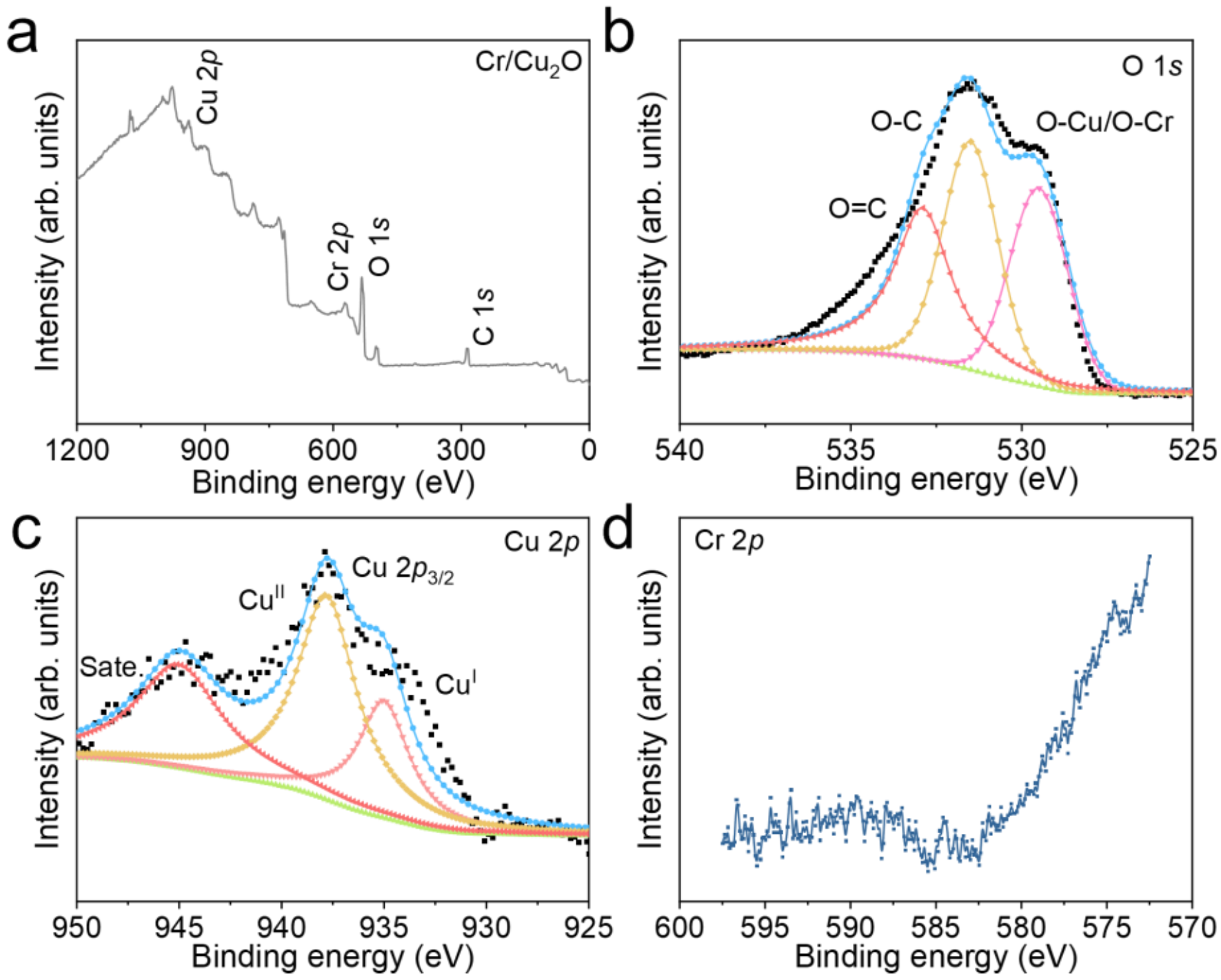

Supplementary Figure 16. X-ray photoelectron spectroscopy (XPS) characterization. (a) Full survey XPS spectrum of the Cr/Cu_2O sample. High-resolution (b) O 1*s*, (b) Cu 2*p*, and (d) Cr 2*p* core-level XPS spectrum of the Cr/Cu_2O sample. Source data are provided as a Source Data file.

This figure presents the XPS spectra of Cr/Cu_2O. The survey spectrum confirms the presence of Cu, Cr, and O elements. High-resolution Cu 2*p* spectrum shows dominant Cu^{I} species with minor Cu^{II}, while O 1*s* spectrum verifies metal-oxygen bond formation. The weak Cr 2*p* signal indicates successful low-content atomic doping that modulates the electronic structure of Cu_2O.

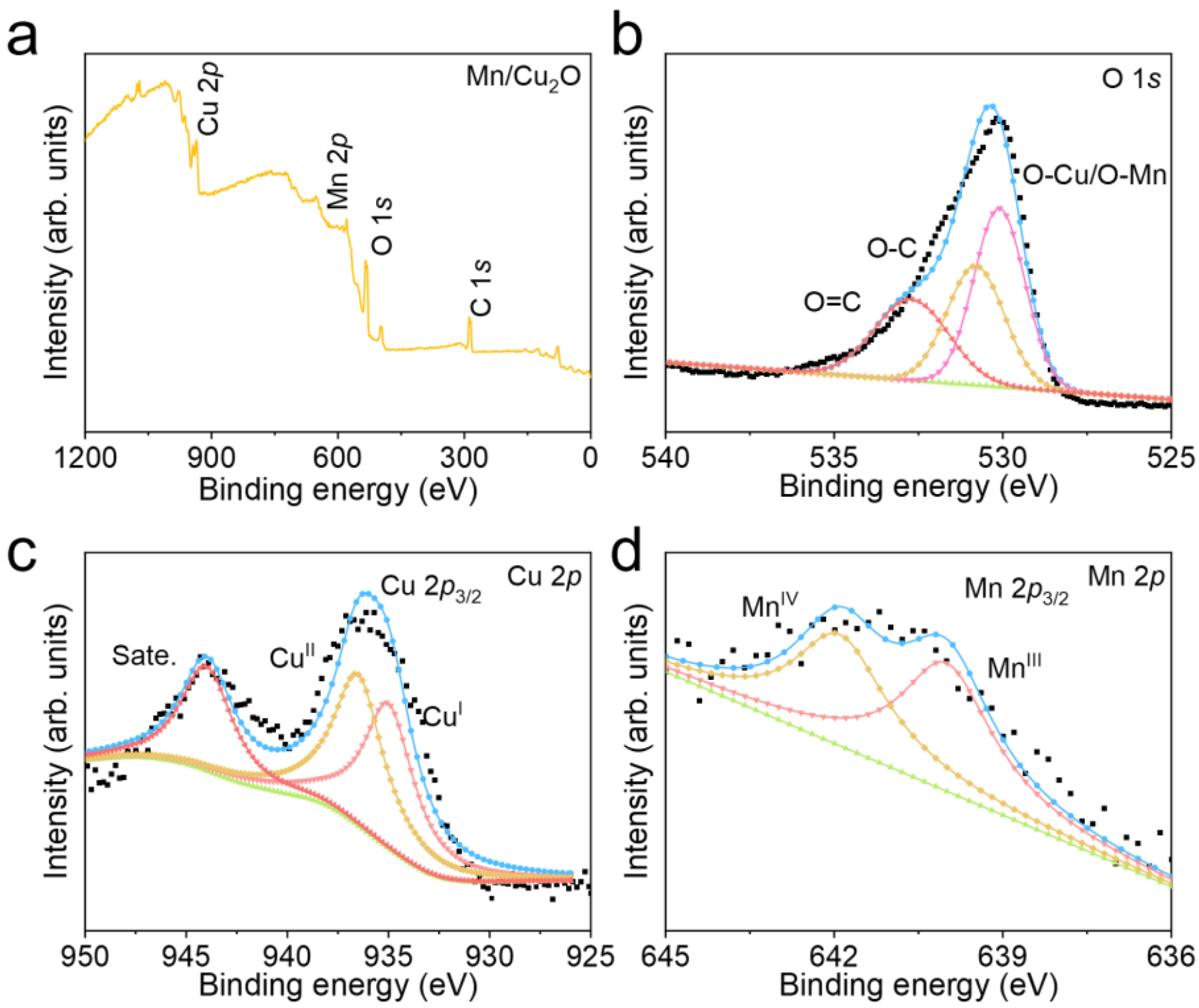

Supplementary Figure 17. X-ray photoelectron spectroscopy (XPS) characterization. (a) Full survey XPS spectrum of the Mn/Cu_2O sample. High-resolution (b) O 1*s*, (b) Cu 2*p*, and (d) Mn 2*p* core-level XPS spectrum of the Mn/Cu_2O sample. Source data are provided as a Source Data file.

This figure shows the XPS characterization of Mn/Cu_2O. The survey spectrum confirms the coexistence of Cu, Mn, and O elements. High-resolution Cu 2*p* spectrum reveals dominant Cu^{I} species with minor Cu^{II}, while Mn 2*p* spectrum indicates mixed Mn^{III}/Mn^{IV} valence states. The O 1*s* spectrum verifies M-O bond formation, demonstrating strong electronic interactions between Mn dopants and Cu_2O support that optimize active site properties.

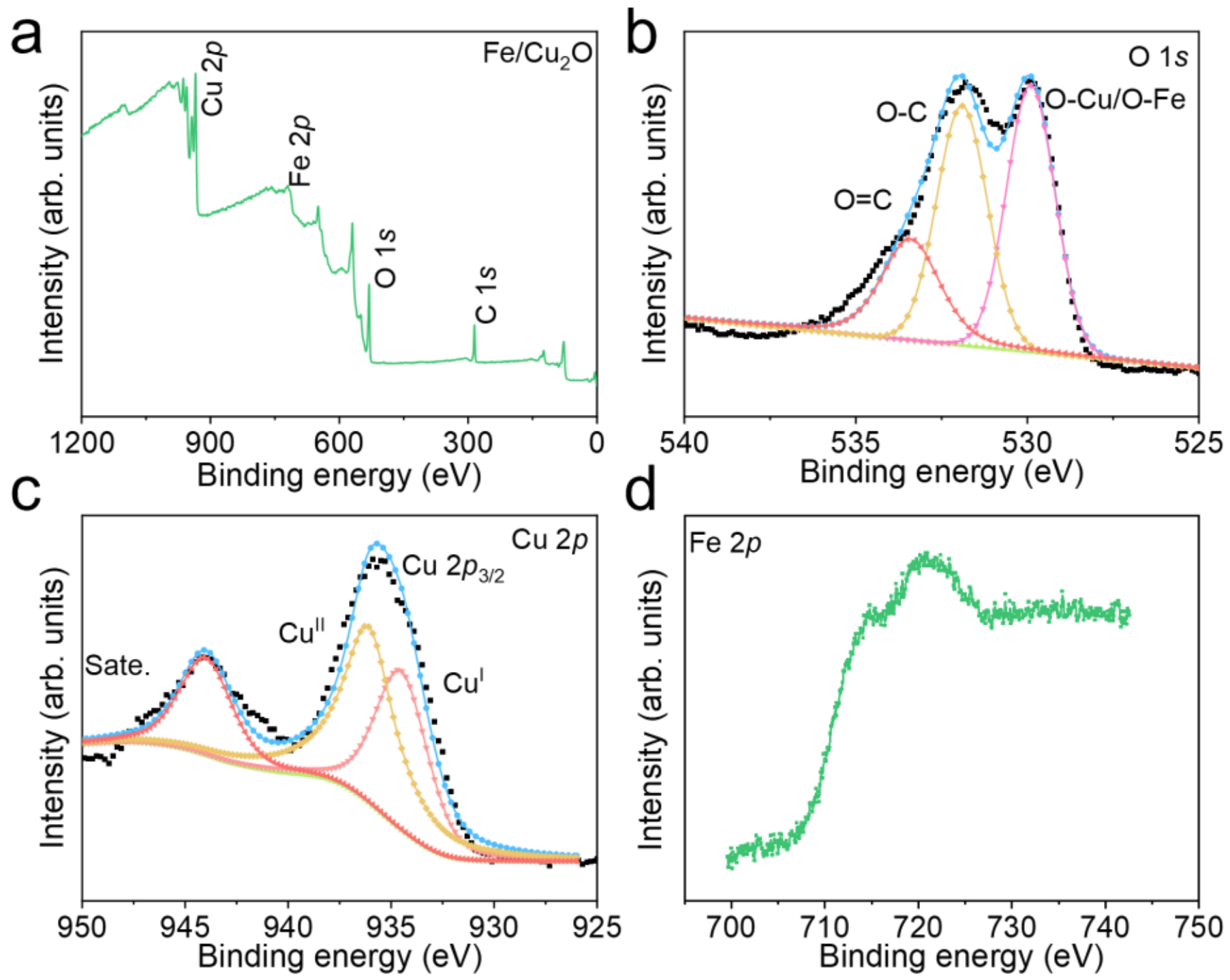

Supplementary Figure 18. X-ray photoelectron spectroscopy (XPS) characterization. (a) Full survey XPS spectrum of the Fe/Cu_2O sample. High-resolution (b) O 1*s*, (b) Cu 2*p*, and (d) Fe 2*p* core-level XPS spectrum of the Fe/Cu_2O sample. Source data are provided as a Source Data file.

This figure presents the XPS characterization of Fe/Cu_2O. The survey spectrum confirms the presence of Cu, Fe, and O elements. High-resolution Cu 2*p* spectrum shows dominant Cu^{I} species with minor Cu^{II} and characteristic satellite peaks. The O 1*s* spectrum is deconvoluted into O=C, O-C, and metal-oxygen (O-Cu/O-Fe) bonds. The broad weak Fe 2*p* signal indicates successful low-content atomic doping, which induces strong electronic interactions with the Cu_2O support to optimize active site properties.

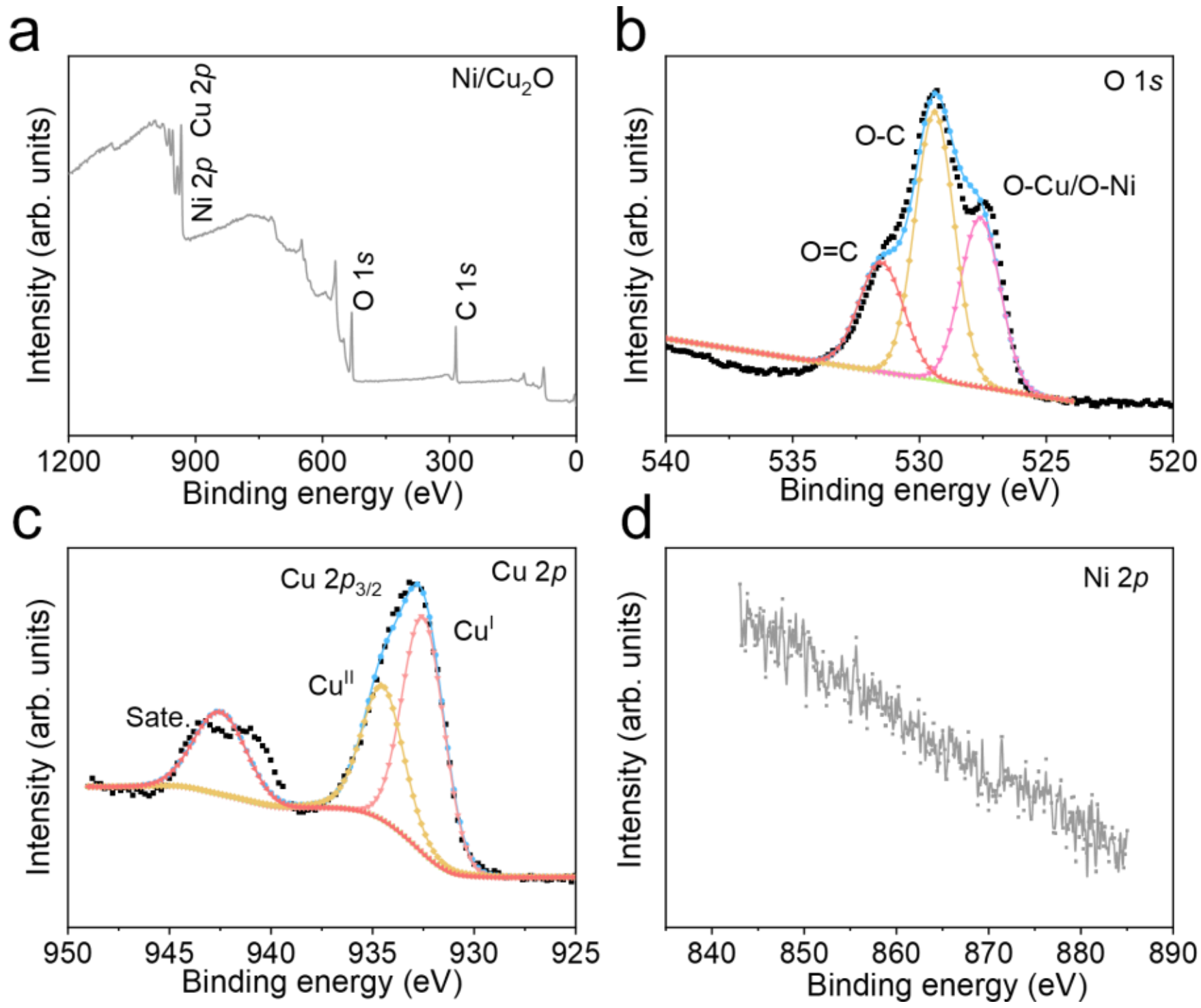

Supplementary Figure 19. X-ray photoelectron spectroscopy (XPS) characterization. (a) Full survey XPS spectrum of the Ni/Cu_2O sample. High-resolution (b) O 1*s*, (b) Cu 2*p*, and (d) Ni 2*p* core-level XPS spectrum of the Ni/Cu_2O sample. Source data are provided as a Source Data file.

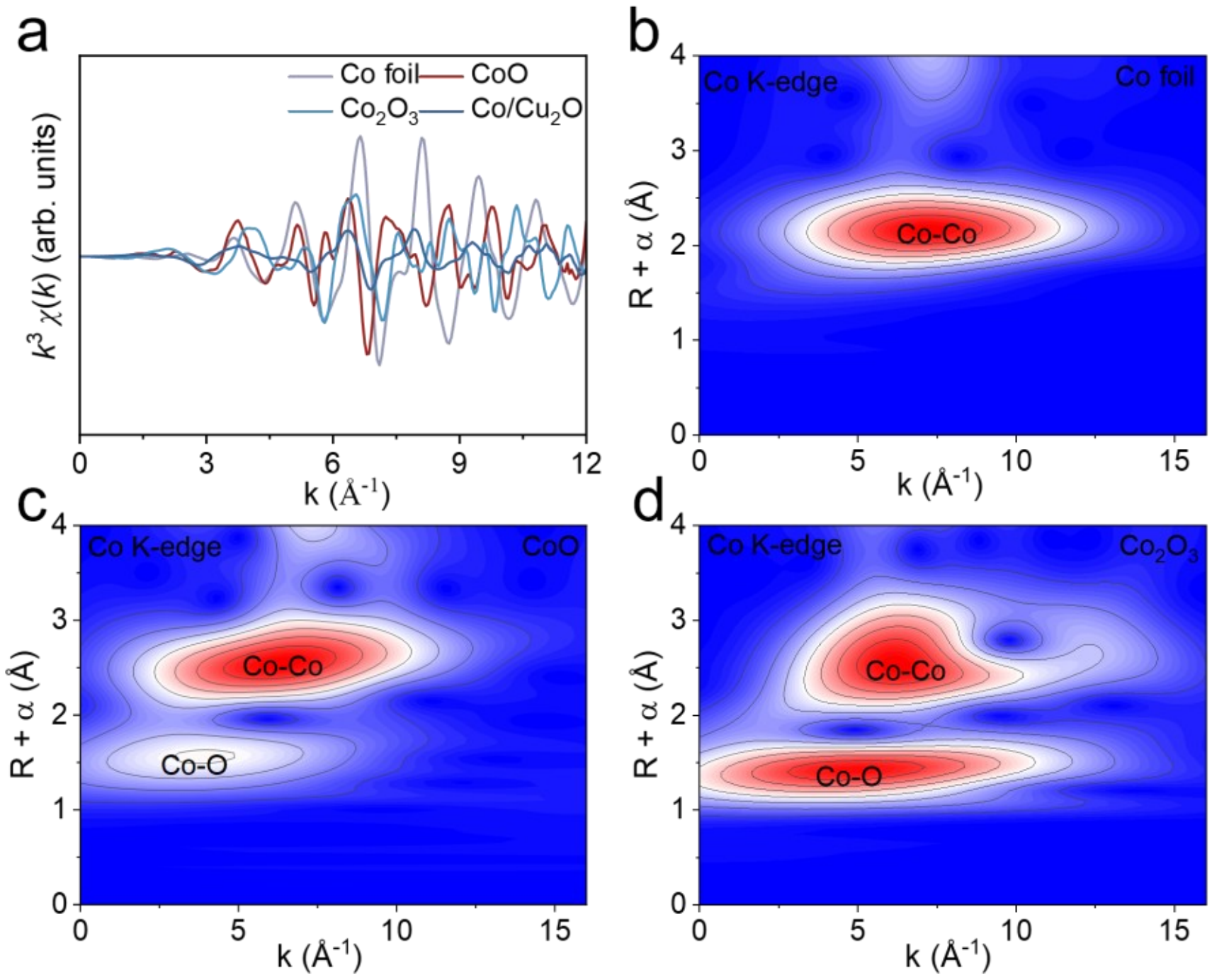

Supplementary Figure 20. Co K-edge extended X-ray absorption fine structure analysis of reference samples and the Co/Cu_2O catalyst. (a) k^3-weighted extended X-ray absorption fine structure (EXAFS) oscillation curves in k-space for Co foil, CoO, Co_2O_3 reference samples and the Co/Cu_2O catalyst. Wavelet transform (WT) contour plots of the Co K-edge EXAFS signal for three reference samples. WT contour plot of (b) Co foil, (c) CoO, and (d) Co_2O_3. Source data are provided as a Source Data file.

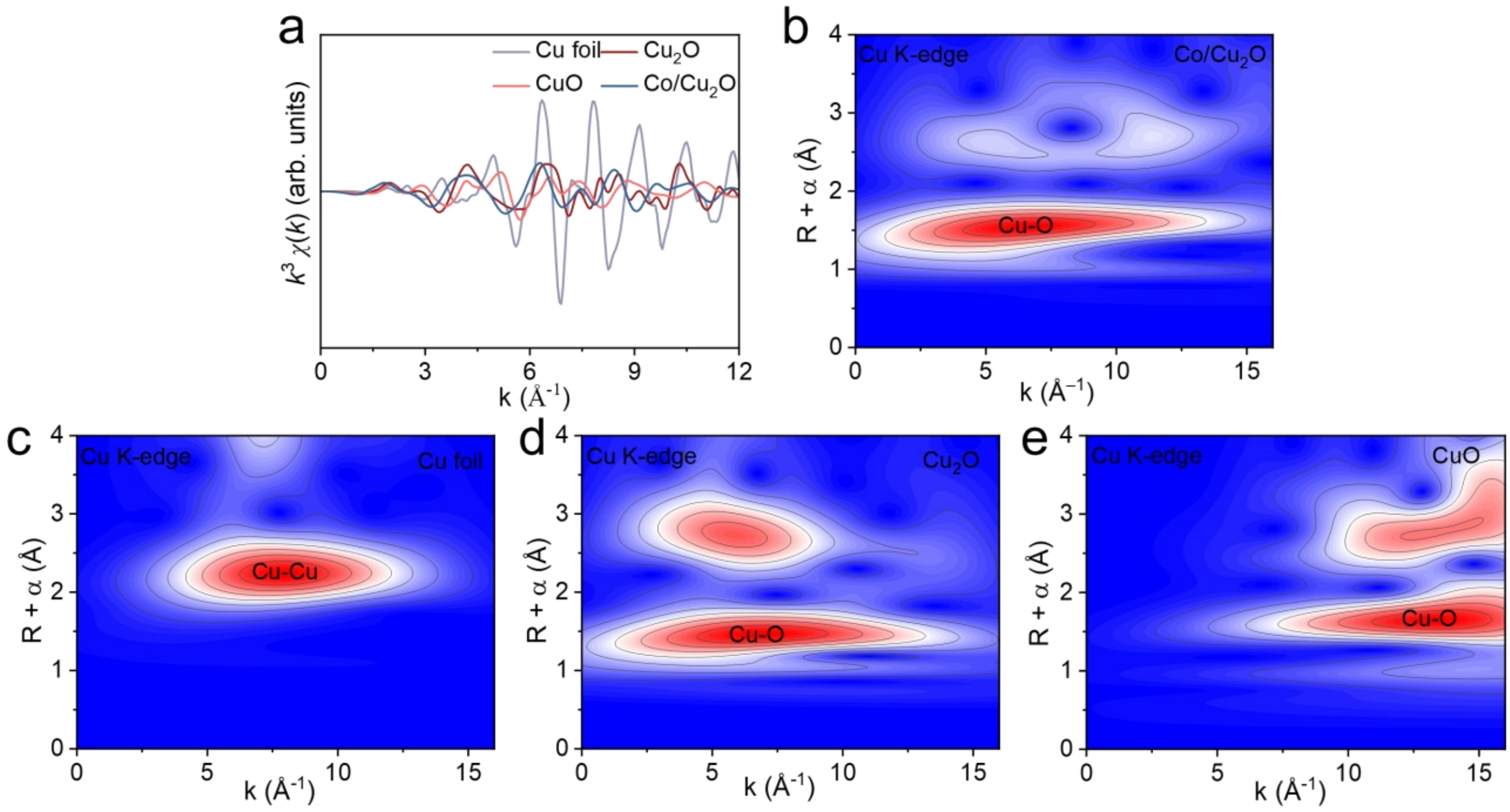

Supplementary Figure 21. Cu K-edge extended X-ray absorption fine structure analysis of reference samples and the Co/Cu_2O catalyst. (a) k^3-weighted extended X-ray absorption fine structure (EXAFS) oscillation curves in k-space for Co foil, CuO, Cu_2O reference samples and the Co/Cu_2O catalyst. Wavelet transform (WT) contour plots of the Cu K-edge EXAFS signal for four reference samples. WT contour plot of (b) Co/Cu_2O, (c) Cu foil, (d) Cu_2O, and (e) CuO. Source data are provided as a Source Data file.

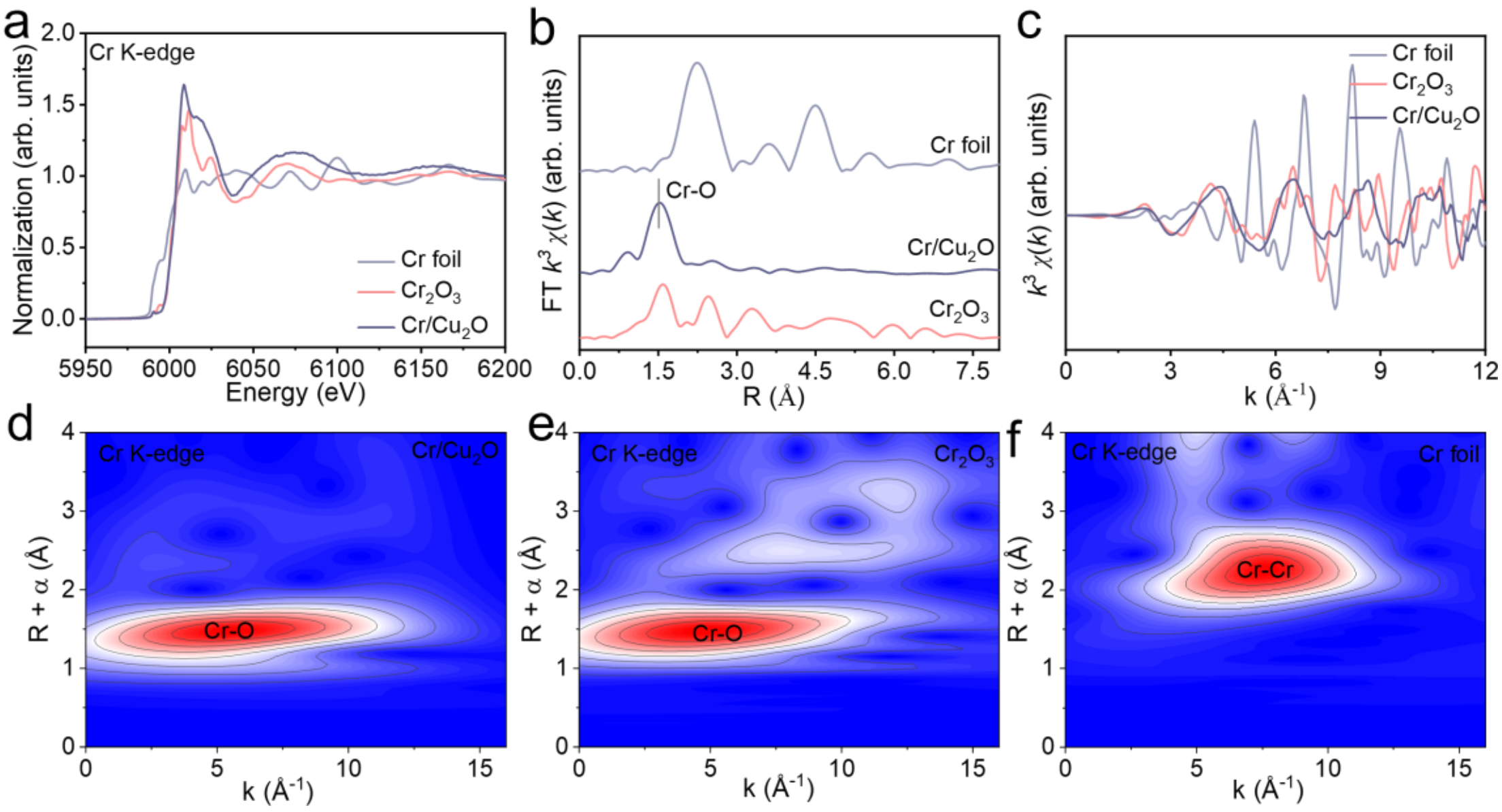

Supplementary Figure 22. Cr K-edge X-ray absorption spectroscopy characterization of reference samples and the Cr/Cu_2O catalyst. (a) Normalized Cr K-edge X-ray absorption near-edge structure (XANES) spectra for Cr/Cu_2O, Cr_2O_3, and Cr foil. (b) k^3-weighted Fourier-transformed extended X-ray absorption fine structure (FT-EXAFS) spectra for Cr/Cu_2O, Cr foil, and Cr_2O_3. (c) k^3-weighted extended X-ray absorption fine structure (EXAFS) oscillation curves in k-space for Cr foil and Cr_2O_3 reference samples and the Cr/Cu_2O catalyst. Wavelet transform (WT) contour plots of the Cr K-edge EXAFS signal for three reference samples. WT contour plot of (d) Cr/Cu_2O, (e) Cr_2O_3, and (f) Cr foil. Source data are provided as a Source Data file.

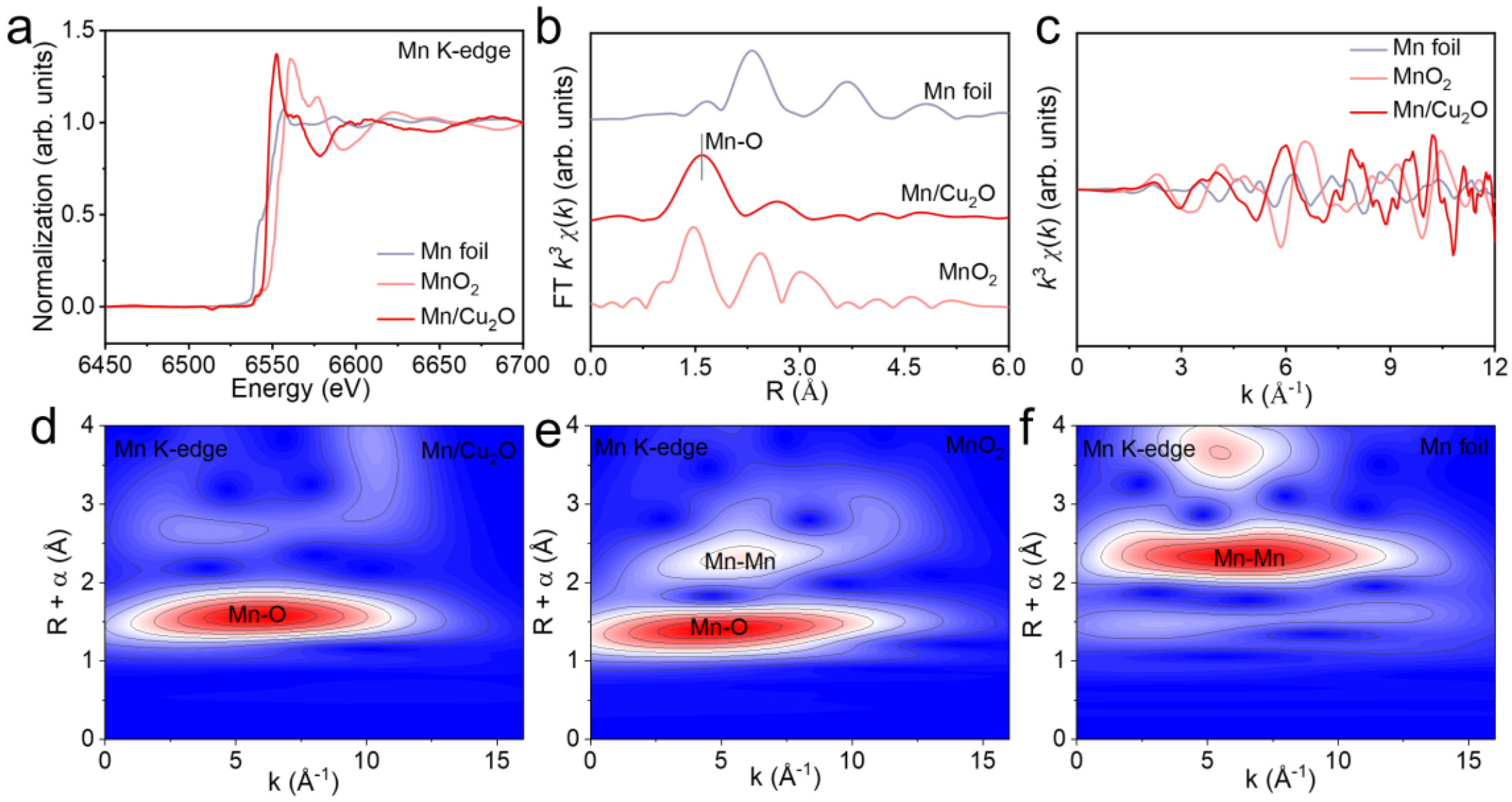

Supplementary Figure 23. Mn K-edge X-ray absorption spectroscopy characterization of reference samples and the Mn/Cu_2O catalyst. (a) Normalized Mn K-edge X-ray absorption near-edge structure (XANES) spectra for Mn/Cu_2O, MnO_2, and Mn foil. (b) k^3-weighted Fourier-transformed extended X-ray absorption fine structure (FT-EXAFS) spectra for Mn/Cu_2O, MnO_2, and Mn foil. (c) k^3-weighted extended X-ray absorption fine structure (EXAFS) oscillation curves in k-space for Mn foil and MnO_2 reference samples and the Mn/Cu_2O catalyst. Wavelet transform (WT) contour plots of the Mn K-edge EXAFS signal for three reference samples. WT contour plot of (d) Mn/Cu_2O, (e) MnO_2, and (f) Mn foil. Source data are provided as a Source Data file.

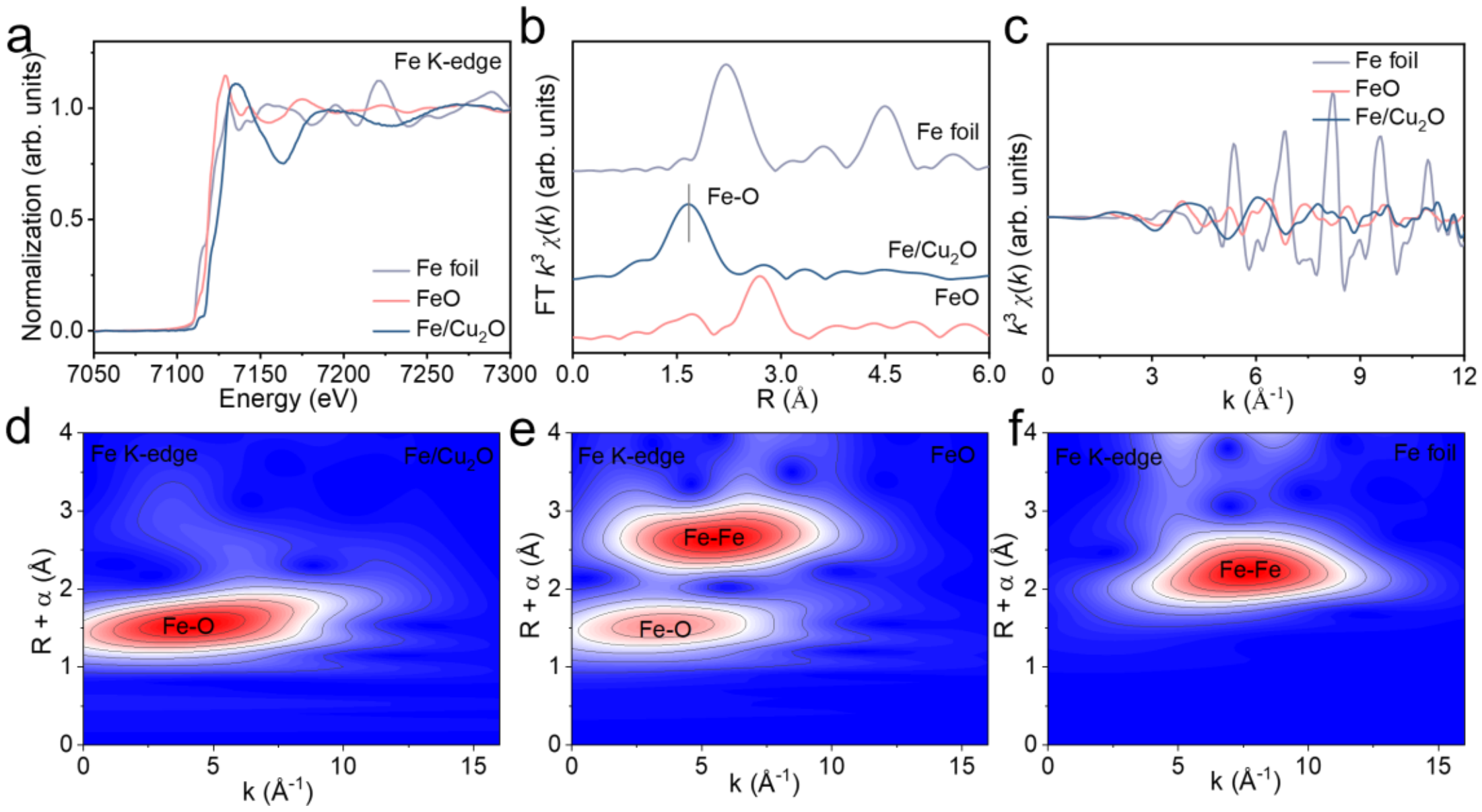

Supplementary Figure 24. Fe K-edge X-ray absorption spectroscopy characterization of reference samples and the Fe/Cu_2O catalyst. (a) Normalized Fe K-edge X-ray absorption near-edge structure (XANES) spectra for Fe/Cu_2O, Fe foil, and FeO. (b) k^3-weighted Fourier-transformed extended X-ray absorption fine structure (FT-EXAFS) spectra for Fe/Cu_2O, Fe foil, and FeO. (c) k^3-weighted extended X-ray absorption fine structure (EXAFS) oscillation curves in k-space for Fe foil and FeO reference samples and the Fe/Cu_2O catalyst. Wavelet transform (WT) contour plots of the Fe K-edge EXAFS signal for three reference samples. WT contour plot of (d) Fe/Cu_2O, (e) FeO, and (f) Fe foil. Source data are provided as a Source Data file.

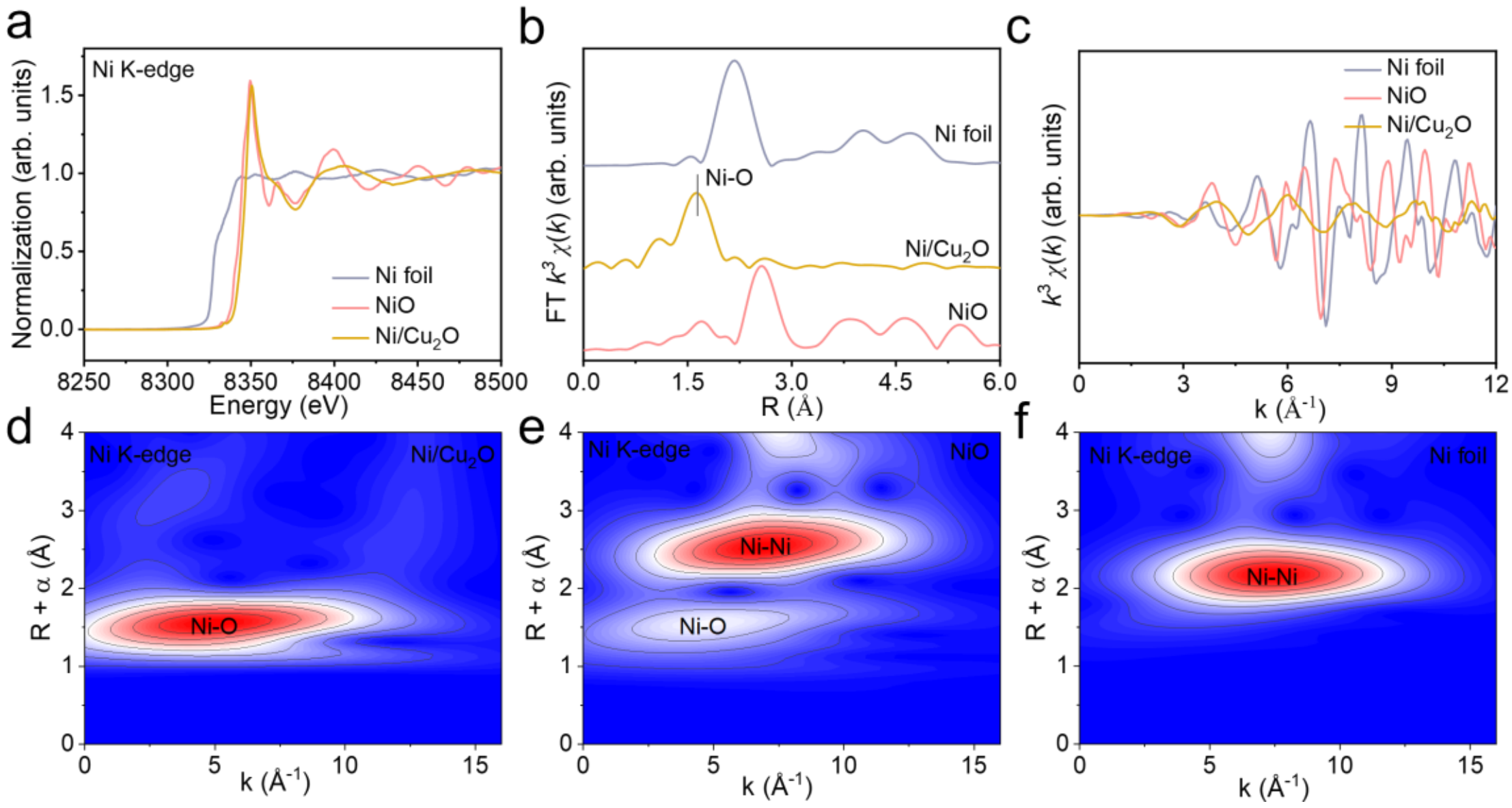

Supplementary Figure 25. Ni K-edge X-ray absorption spectroscopy characterization of reference samples and the Ni/Cu_2O catalyst. (a) Normalized Ni K-edge X-ray absorption near-edge structure (XANES) spectra for Ni/Cu_2O, NiO, and Ni foil. (b) k^3-weighted Fourier-transformed extended X-ray absorption fine structure (FT-EXAFS) spectra for Ni/Cu_2O, Ni foil, and NiO. (c) k^3-weighted extended X-ray absorption fine structure (EXAFS) oscillation curves in k-space for Ni foil and NiO reference samples and the Ni/Cu_2O catalyst. Wavelet transform (WT) contour plots of the Ni K-edge EXAFS signal for three reference samples. WT contour plot of (d) Ni/Cu_2O, (e) NiO, and (f) Ni foil. Source data are provided as a Source Data file.

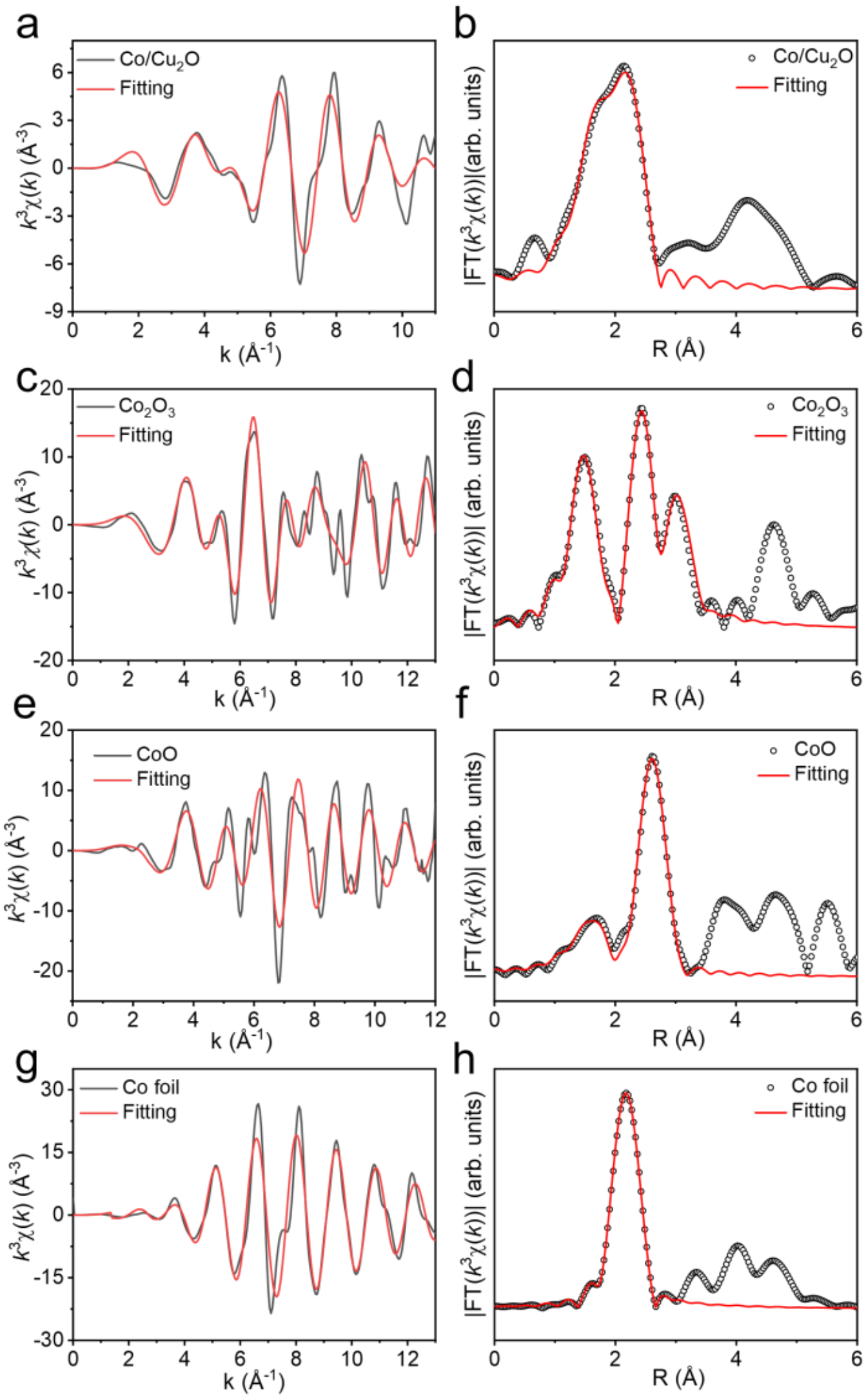

Supplementary Figure 26. **Co K-edge extended X-ray absorption fine structure fitting results for the Co/Cu_2O catalyst and reference samples**. (a) k^3-weighted EXAFS oscillation function $k^3\chi(k)$ of the Co/Cu_2O. (b) The Fourier-transformed k^3-weighted EXAFS spectrum of Co/Cu_2O in R-space. (c) k^3-weighted EXAFS oscillation function $k^3\chi(k)$ of the Co_2O_3. (d) The Fourier-transformed k^3-weighted EXAFS spectrum of Co_2O_3 in R-space. (e) k^3-weighted EXAFS oscillation function $k^3\chi(k)$ of the CoO. (f) The Fourier-transformed k^3-weighted EXAFS spectrum of CoO in R-space. (g) k^3-weighted EXAFS oscillation function $k^3\chi(k)$ of the Co foil. (h) The Fourier-transformed k^3-weighted EXAFS spectrum of Co foil in R-space. Source data are provided as a Source Data file.

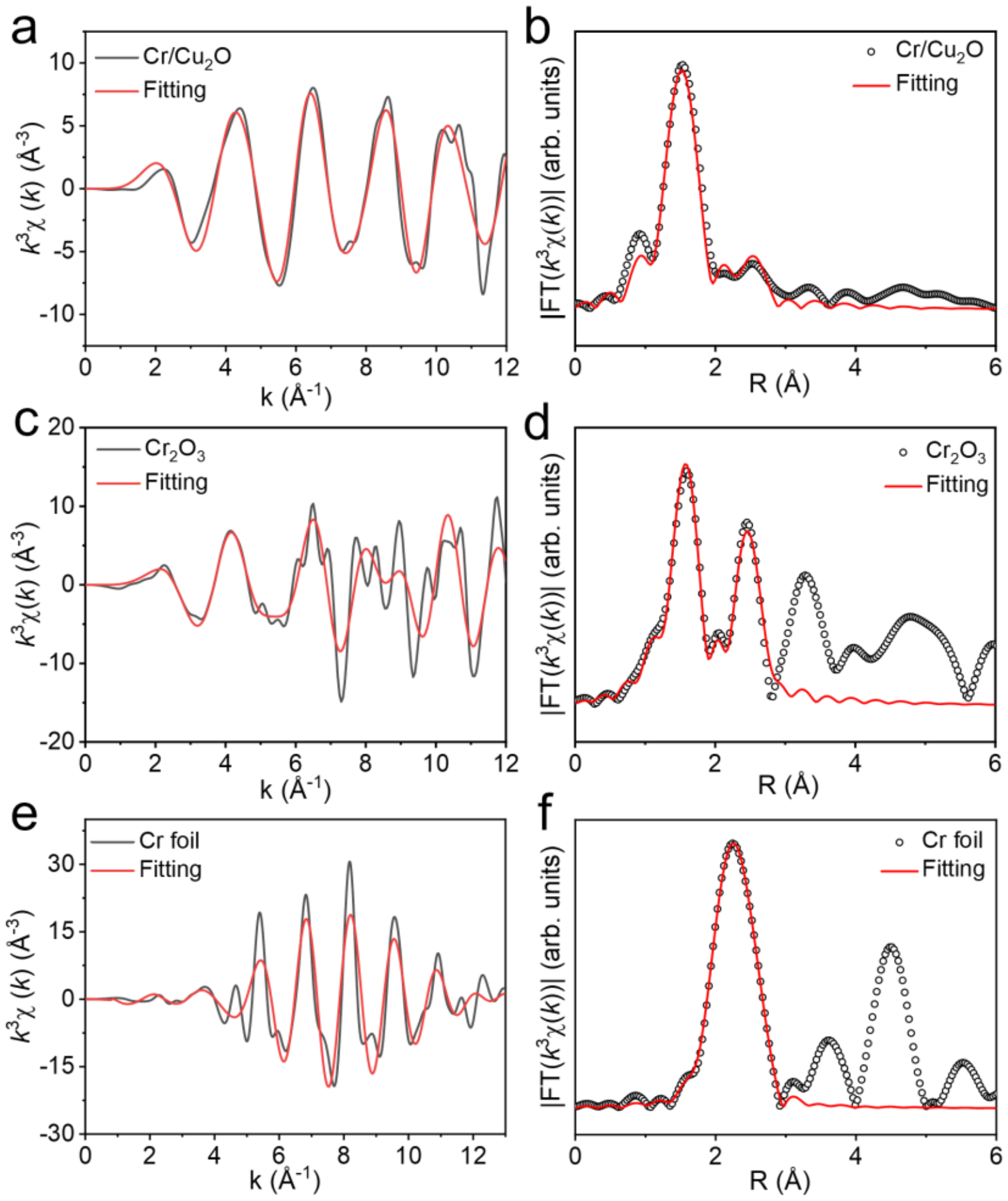

Supplementary Figure 27. Cr K-edge extended X-ray absorption fine structure fitting results for the Cr/Cu_2O catalyst and reference samples. (a) k^3-weighted EXAFS oscillation function $k^3\chi(k)$ of the Cr/Cu_2O. (b) The Fourier-transformed k^3-weighted EXAFS spectrum of Cr/Cu_2O in R-space. (c) k^3-weighted EXAFS oscillation function $k^3\chi(k)$ of the Cr_2O_3. (d) The Fourier-transformed k^3-weighted EXAFS spectrum of Cr_2O_3 in R-space. (e) k^3-weighted EXAFS oscillation function $k^3\chi(k)$ of the Cr foil. (f) The Fourier-transformed k^3-weighted EXAFS spectrum of Cr foil in R-space. Source data are provided as a Source Data file.

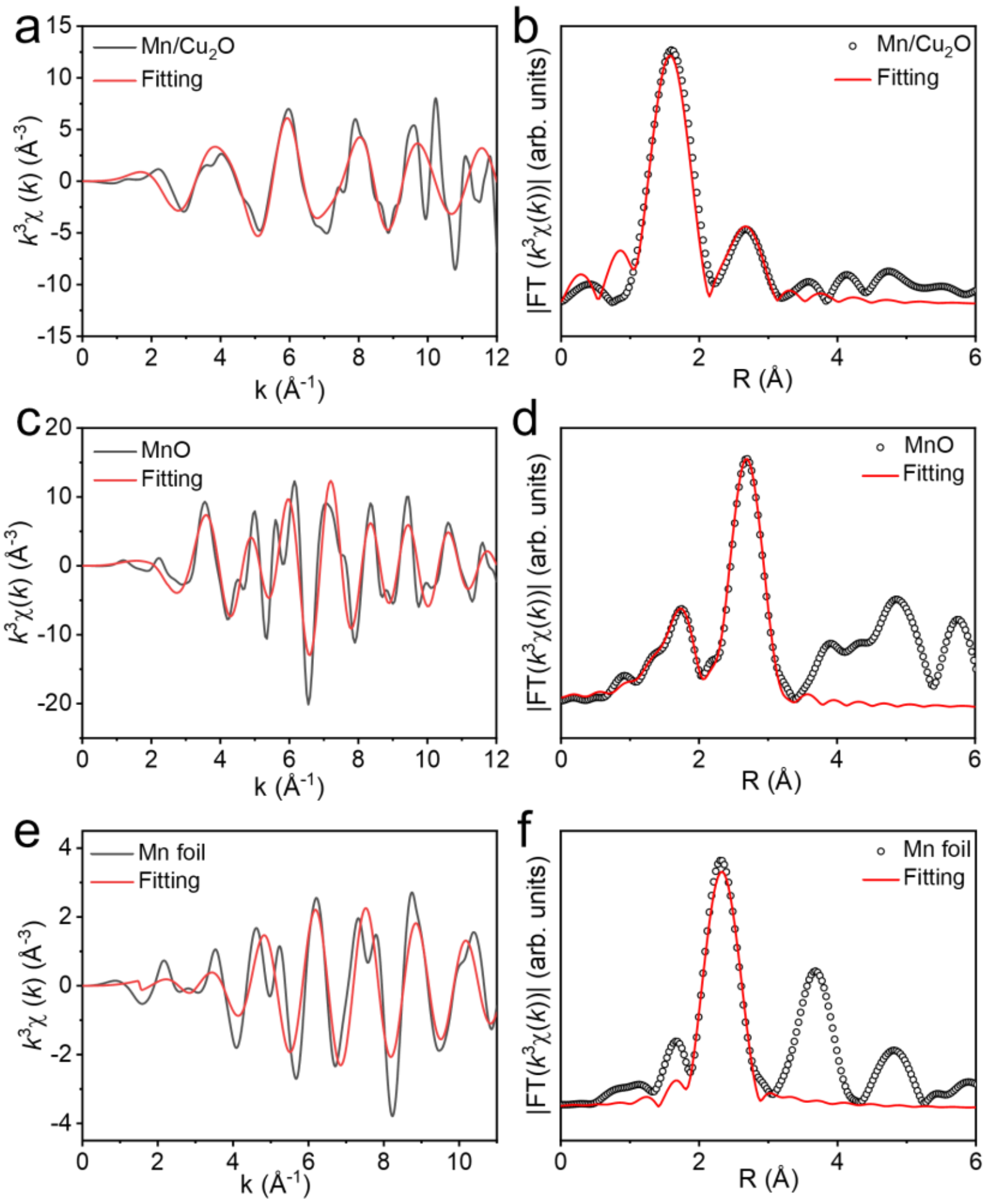

Supplementary Figure 28. **Mn K-edge extended X-ray absorption fine structure fitting results for the Mn/Cu_2O catalyst and reference samples.** (a) k^3-weighted EXAFS oscillation function $k^3\chi(k)$ of the Mn/Cu_2O. (b) The Fourier-transformed k^3-weighted EXAFS spectrum of Mn/Cu_2O in R-space. (c) k^3-weighted EXAFS oscillation function $k^3\chi(k)$ of the MnO. (d) The Fourier-transformed k^3-weighted EXAFS spectrum of MnO in R-space. (e) k^3-weighted EXAFS oscillation function $k^3\chi(k)$ of the Mn foil. (f) The Fourier-transformed k^3-weighted EXAFS spectrum of Mn foil in R-space. Source data are provided as a Source Data file.

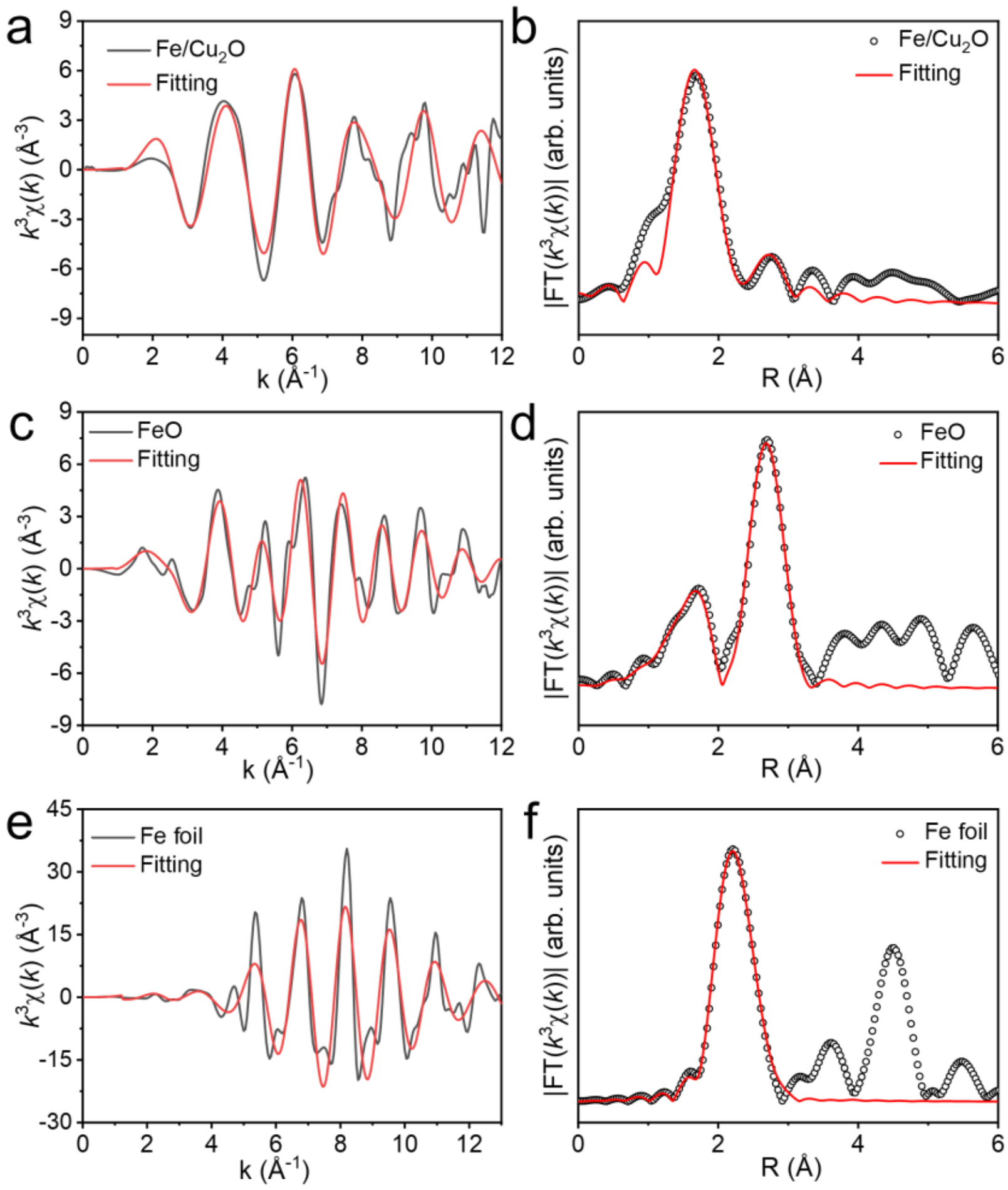

Supplementary Figure 29. **Fe K-edge extended X-ray absorption fine structure fitting results for the Fe/Cu_2O catalyst and reference samples.** (a) k^3-weighted EXAFS oscillation function $k^3\chi(k)$ of the Fe/Cu_2O. (b) The Fourier-transformed k^3-weighted EXAFS spectrum of Fe/Cu_2O in R-space. (c) k^3-weighted EXAFS oscillation function $k^3\chi(k)$ of the FeO. (d) The Fourier-transformed k^3-weighted EXAFS spectrum of FeO in R-space. (e) k^3-weighted EXAFS oscillation function $k^3\chi(k)$ of the Fe foil. (f) The Fourier-transformed k^3-weighted EXAFS spectrum of Fe foil in R-space. Source data are provided as a Source Data file.

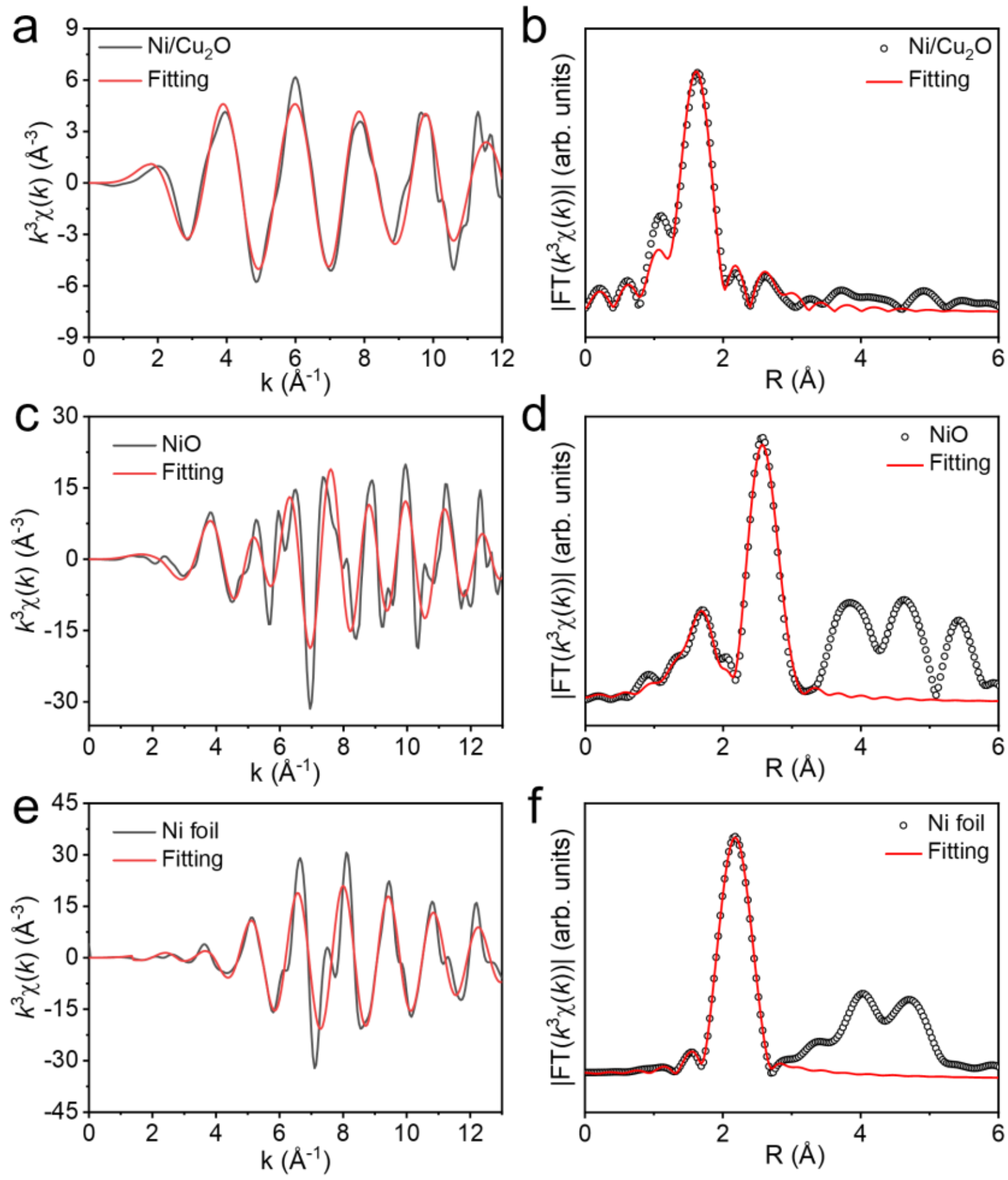

Supplementary Figure 30. **Ni K-edge extended X-ray absorption fine structure fitting results for the Ni/Cu_2O catalyst and reference samples**. (a) k^3-weighted EXAFS oscillation function $k^3\chi(k)$ of the Ni/Cu_2O. (b) The Fourier-transformed k^3-weighted EXAFS spectrum of Ni/Cu_2O in R-space. (c) k^3-weighted EXAFS oscillation function $k^3\chi(k)$ of the NiO. (d) The Fourier-transformed k^3-weighted EXAFS spectrum of NiO in R-space. (e) k^3-weighted EXAFS oscillation function $k^3\chi(k)$ of the Ni foil. (f) The Fourier-transformed k^3-weighted EXAFS spectrum of Ni foil in R-space. Source data are provided as a Source Data file.

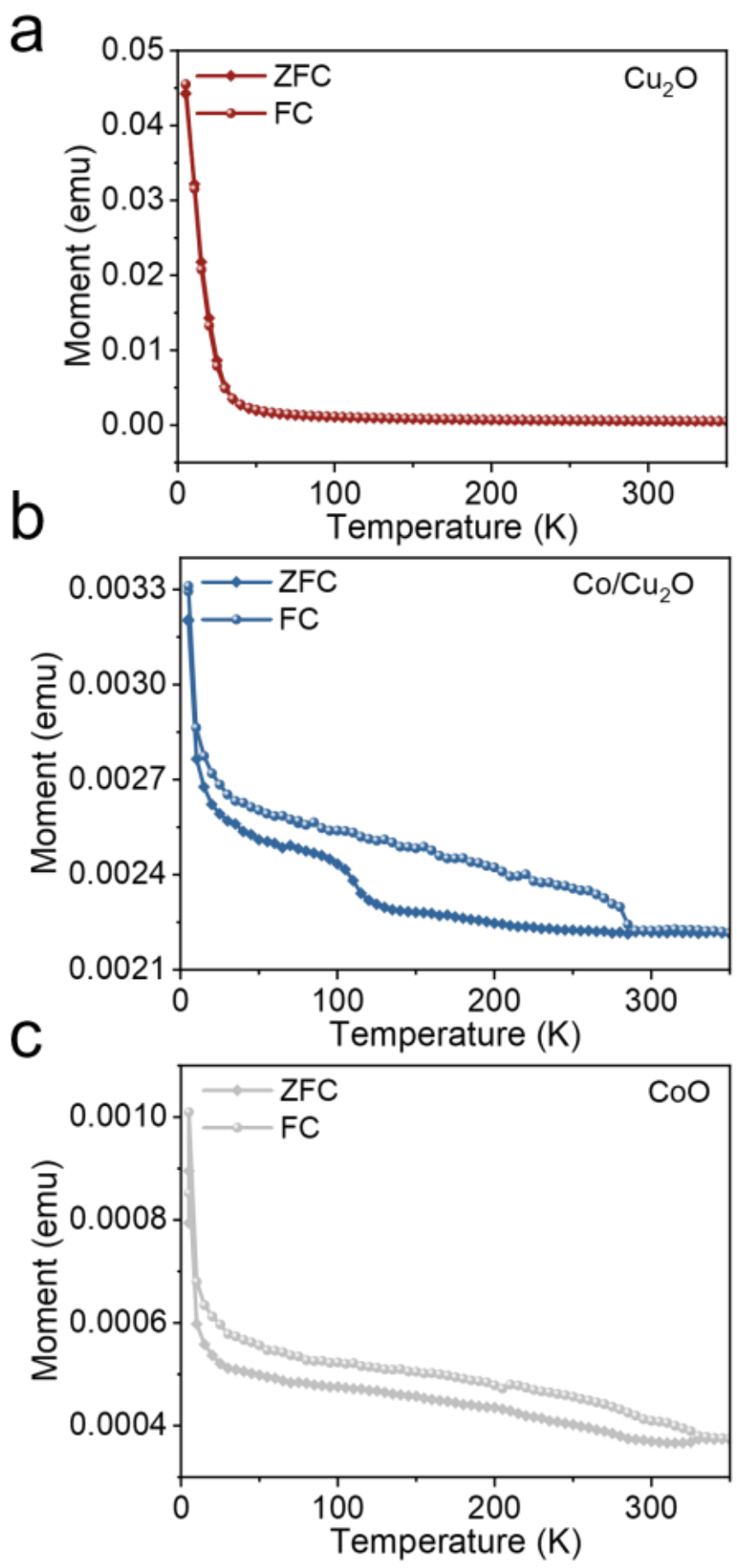

Supplementary Figure 31. Zero-field-cooled (ZFC) and field-cooled (FC) magnetization profiles. ZFC and FC magnetization curves of the (a) Cu_2O, (b) Co/Cu_2O, and (c) CoO. Source data are provided as a Source Data file.

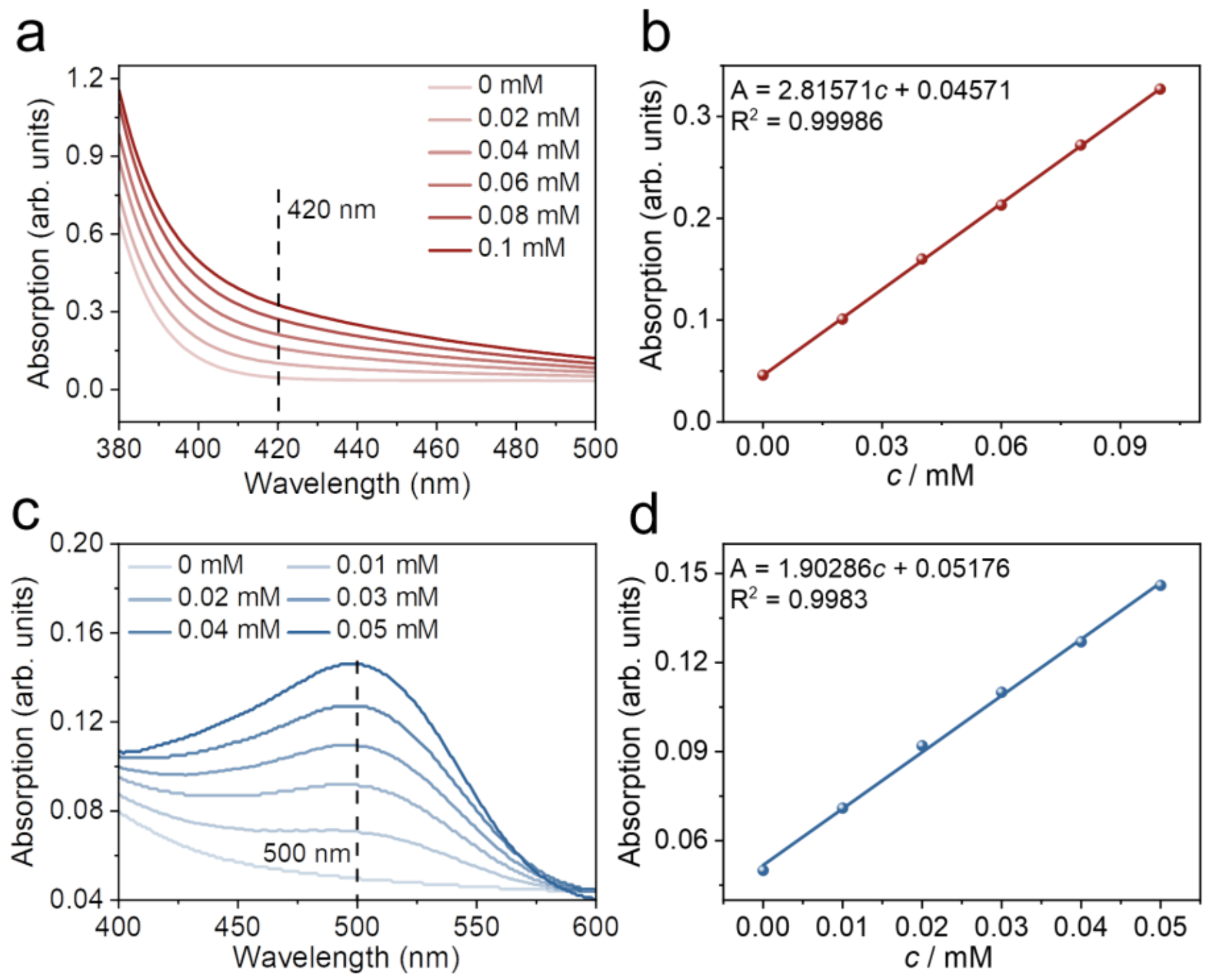

Supplementary Figure 32. UV-Vis absorption calibration curves for quantitative analysis of nitrite and ammonium products. (a) UV-Vis curves of $NaNO_2$ solutions with standards concentrations. (b) The linear standard curve for calculating of NO_2^- production. (c) UV-Vis curves of NH_4Cl solutions with standards concentrations. (d) The linear standard curve for calculating of NH_4^+ production. Source data are provided as a Source Data file.

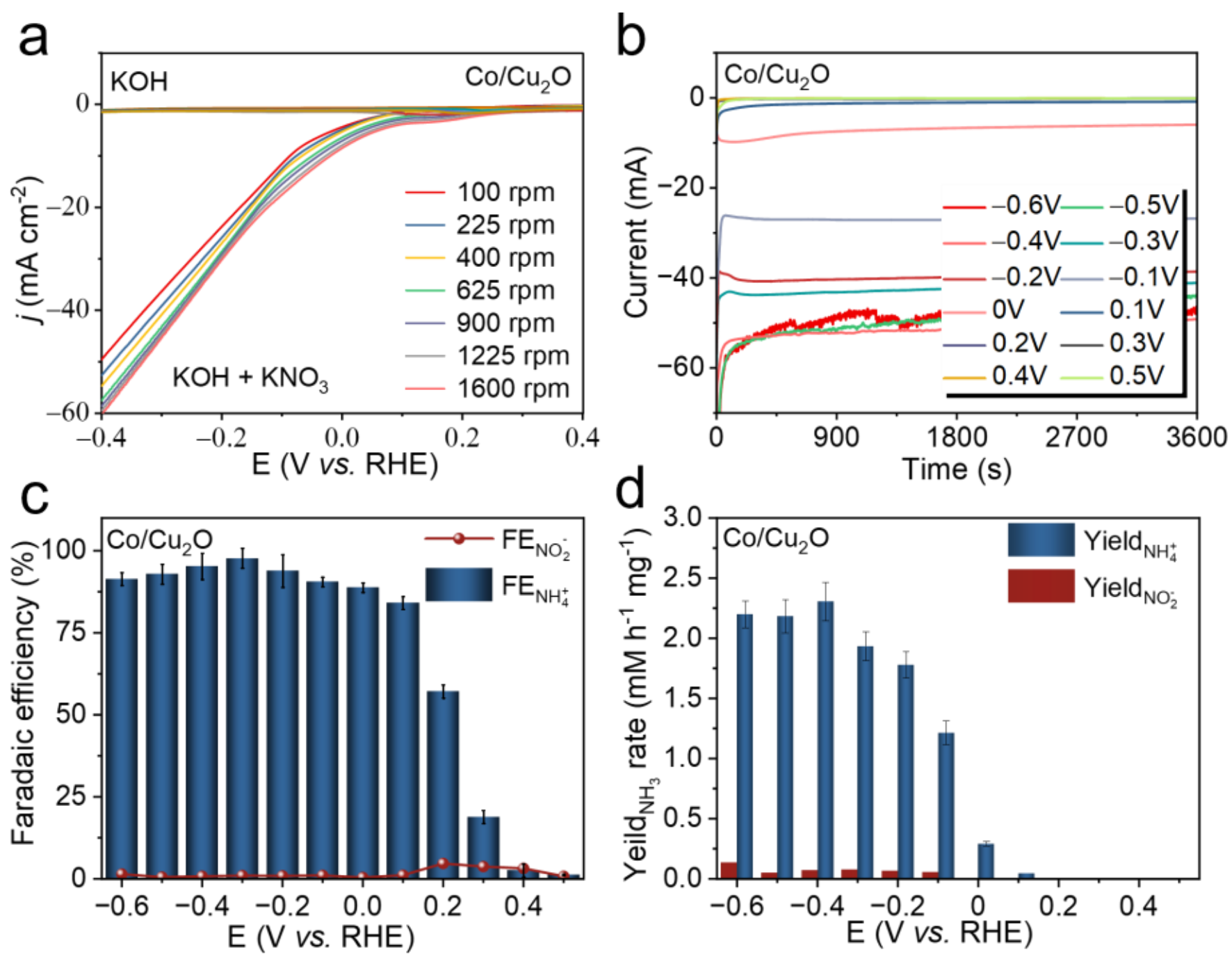

Supplementary Figure 33. Electrochemical nitrate reduction reaction performance. (a) Linear sweep voltammetry (LSV) curves of Co/Cu_2O collected in 0.1 M KOH and 0.1 M KOH + 0.1 M KNO_3 electrolyte at rotation rates from 100 rpm to 1600 rpm. (b) Chronoamperometric (i-t) profiles of Co/Cu_2O recorded in 0.1 M KOH + 0.1 M KNO_3 electrolyte at graded constant applied potentials from −0.6 V to 0.5 V *vs*. RHE, with a total test duration of 3600 s for each potential condition. (c) Faradaic efficiencies of NH_4^+ and NO_2^- over Co/Cu_2O at different applied potentials. All error bars represent the standard deviation calculated from three independent electrolysis replicate experiments. (d) NH_3 yield rate at different potential of Co/Cu_2O, respectively. All error bars correspond to the standard deviation derived from three independent quantitative measurements. Source data are provided as a Source Data file.

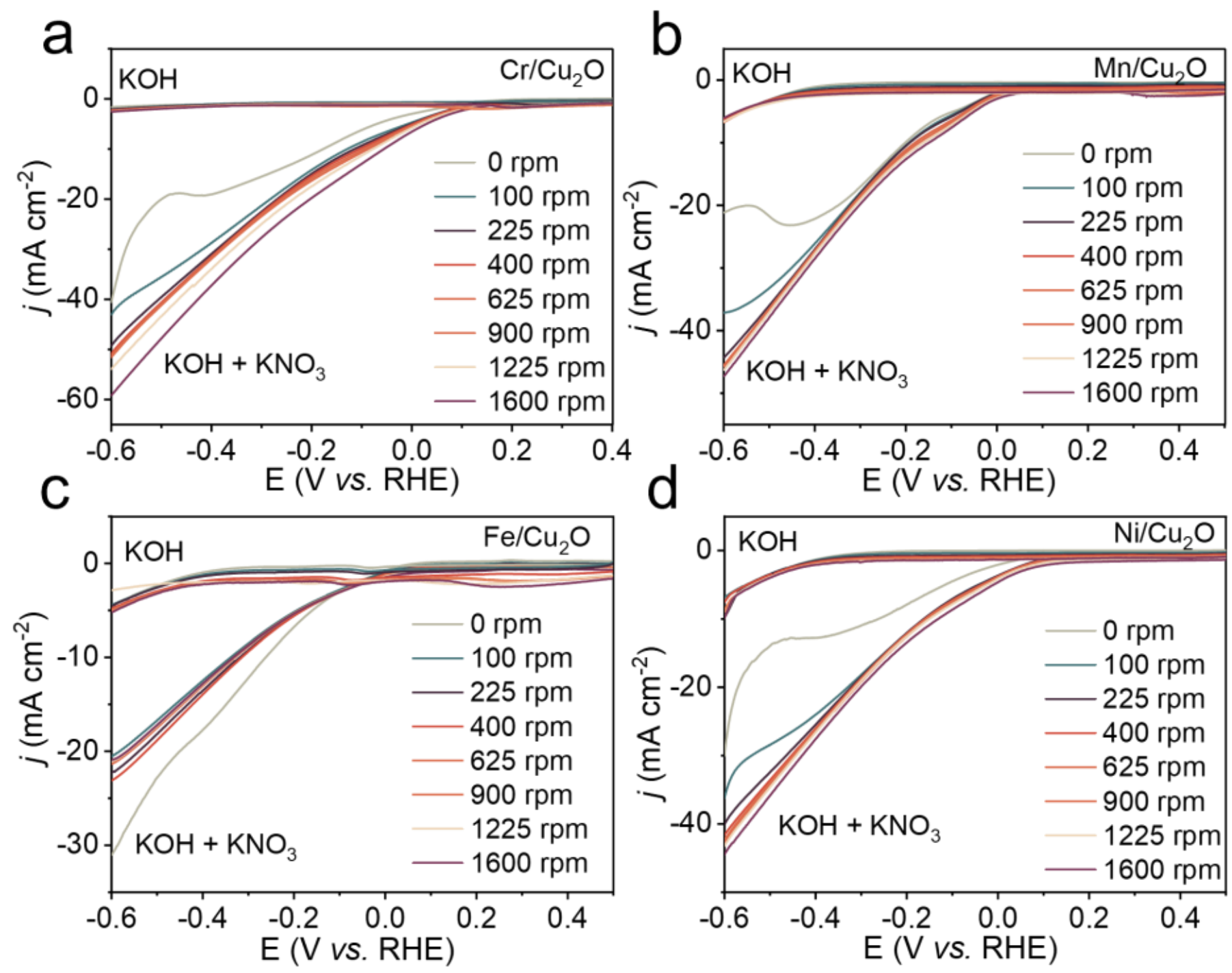

Supplementary Figure 34. Linear sweep voltammetry curves of nitrate reduction reaction. LSV curves of NO_3RR on (a) Cr/Cu_2O, (b) Mn/Cu_2O, (c) Fe/Cu_2O, and (d) Ni/Cu_2O under different rotation rates in 0.1 M KOH and 0.1 M KOH + 0.1 M KNO_3 solution, respectively. Source data are provided as a Source Data file.

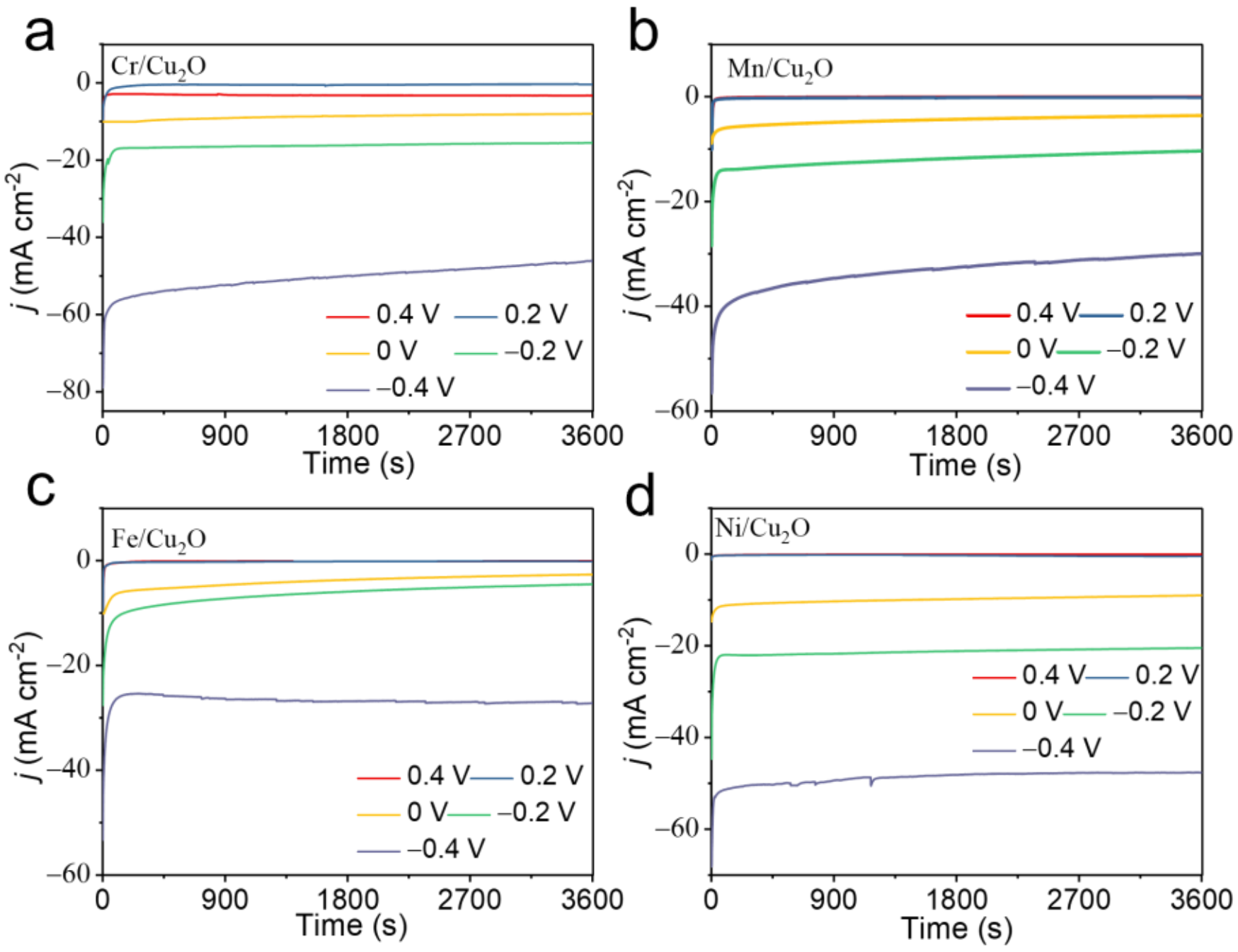

Supplementary Figure 35. Chronoamperometric (i–t) profiles. The i-t curves of (a) Cr/Cu_2O, (b) Mn/Cu_2O, (c) Fe/Cu_2O, and (d) Ni/Cu_2O at different potential, respectively. Source data are provided as a Source Data file.

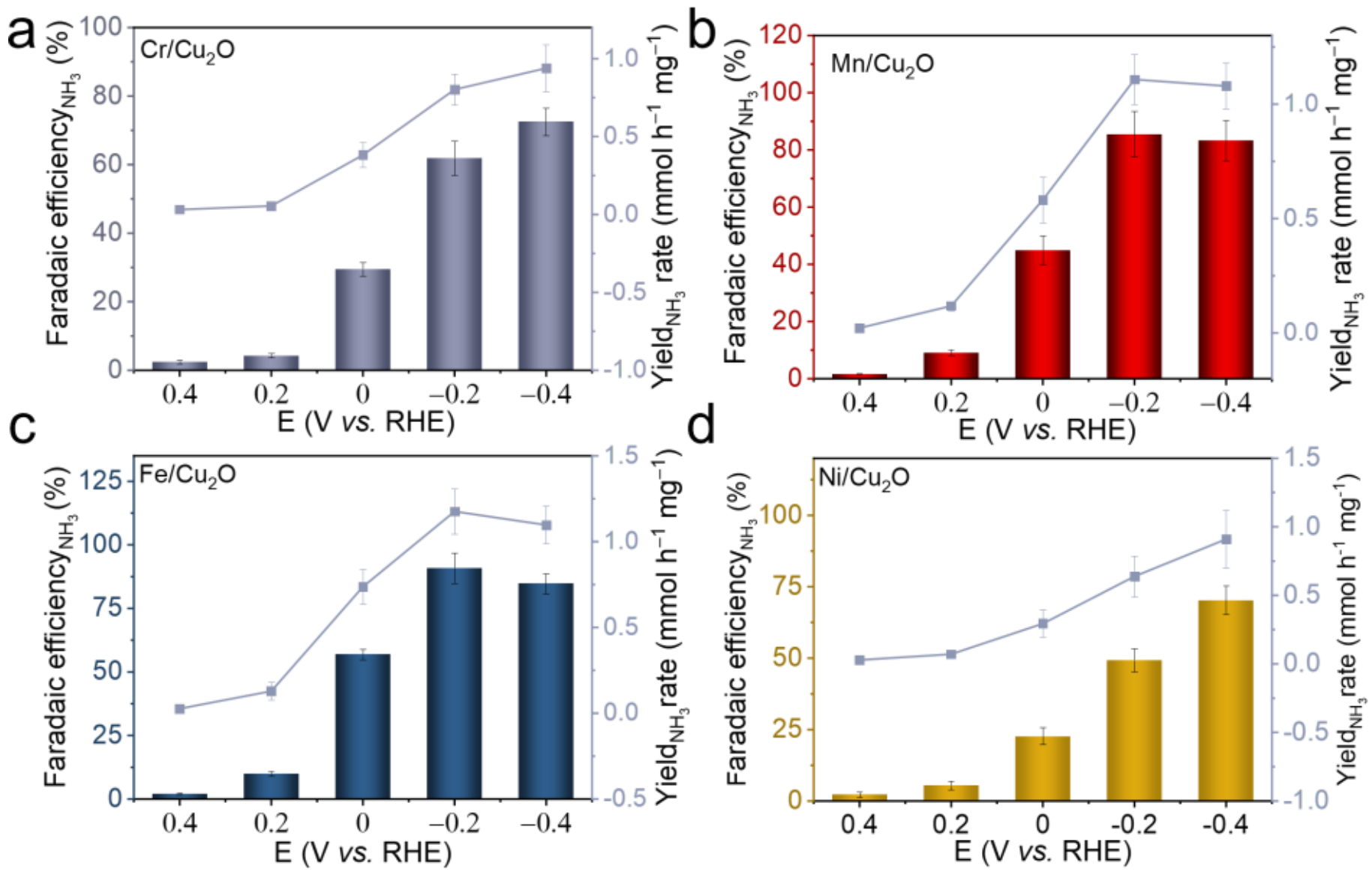

Supplementary Figure 36. Faradaic efficiency of ammonia and ammonia yield rate. (a) Faradaic efficiency of NH_3 (gray columns, left axis) and NH_3 yield rate (line with square markers, right axis) of Cr/Cu_2O, measured at a series of applied potentials from 0.4 V to −0.4 V *vs.* reversible hydrogen electrode (RHE). (b) Faradaic efficiency of NH_3 (red columns, left axis) and NH_3 yield rate (line with square markers, right axis) of Mn/Cu_2O, measured at a series of applied potentials from 0.4 V to −0.4 V *vs.* RHE. (c) Faradaic efficiency of NH_3 (dark blue columns, left axis) and mass-normalized NH_3 yield rate (line with square markers, right axis) of Fe/Cu_2O, measured at a series of applied potentials from 0.4 V to −0.4 V *vs.* RHE. (d) Faradaic efficiency of NH_3 (golden columns, left axis) and mass-normalized NH_3 yield rate (line with square markers, right axis) of Ni/Cu_2O, measured at a series of applied potentials from 0.4 V to −0.4 V *vs.* RHE. All error bars represent the standard deviation calculated from three independent controlled-potential electrolysis replicate experiments. Source data are provided as a Source Data file.

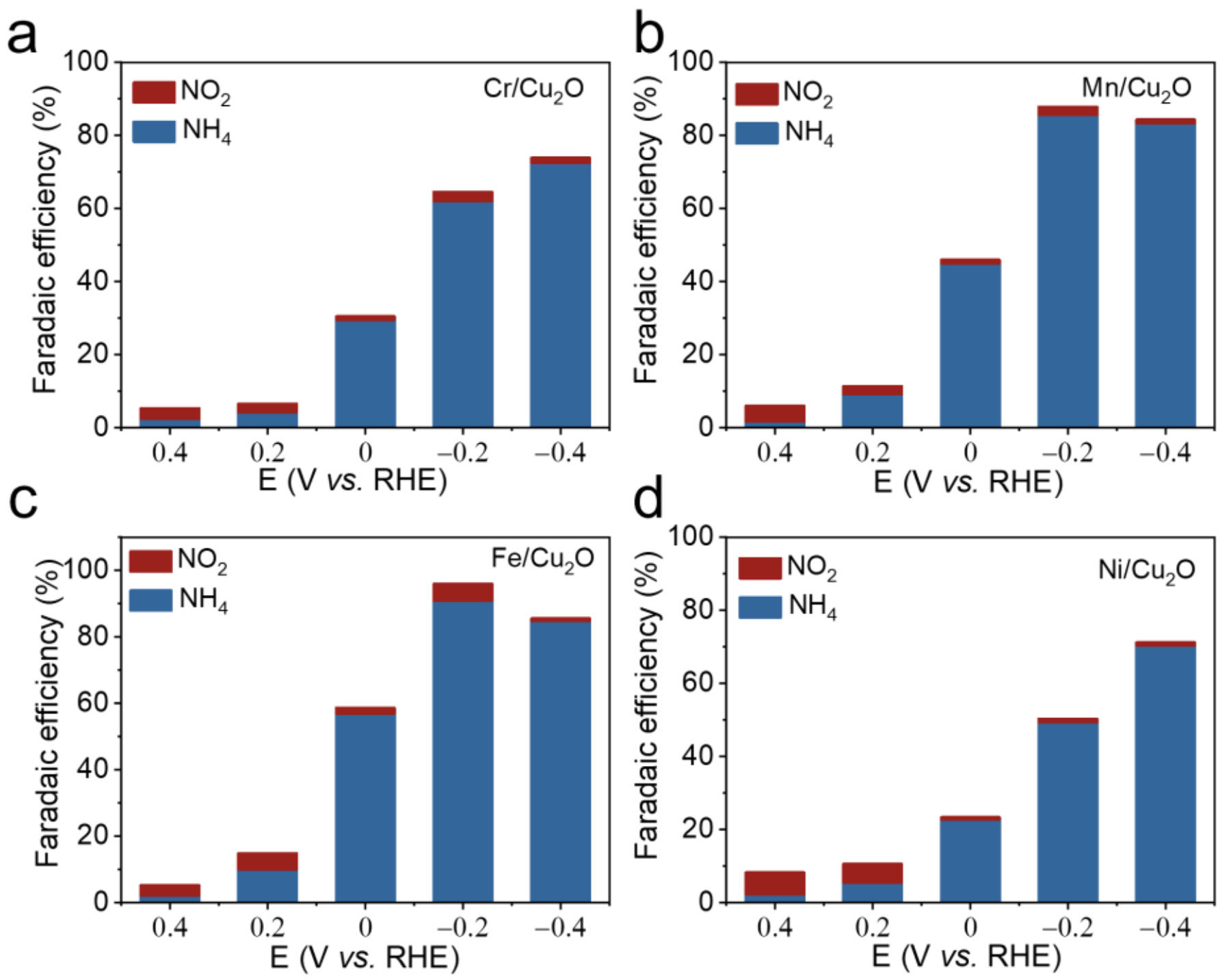

Supplementary Figure 37. Faradaic efficiency distributions of nitrite and ammonium products. The FE plots of (a) Cr/Cu_2O, (b) Mn/Cu_2O, (c) Fe/Cu_2O, and (d) Ni/Cu_2O at different potential, respectively. Source data are provided as a Source Data file.

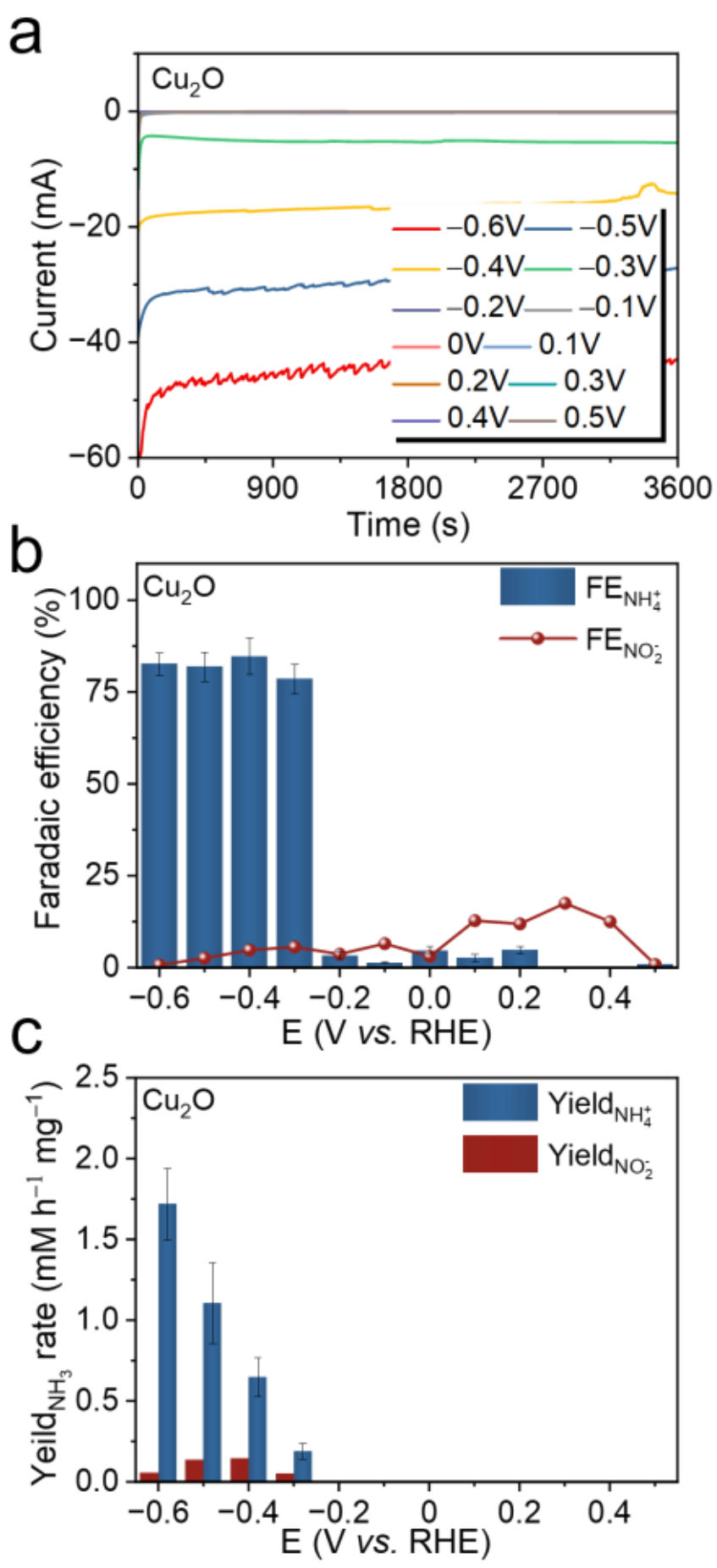

Supplementary Figure 38. Electrochemical nitrate reduction reaction performance. (a) Chronoamperometric (i–t) profiles of pristine Cu_2O measured in 0.1 M KOH + 0.1 M KNO_3 electrolyte at graded constant applied potentials from −0.6 V to 0.5 V *vs*. reversible hydrogen electrode (RHE). Each measurement is conducted for a total duration of 3600 s. (b) Faradaic efficiencies of NH_4^+ (blue columns) and NO_2^- (red line with circular markers) over pristine Cu_2O at different applied potentials. All error bars represent the standard deviation calculated from three independent controlled-potential electrolysis replicate experiments. (c) Yield rates of NH_4^+ (blue columns) and NO_2^- (red columns) over Cu_2O at different applied potentials. All error bars correspond to the standard deviation derived from three independent quantitative product detection measurements. Source data are provided as a Source Data file.

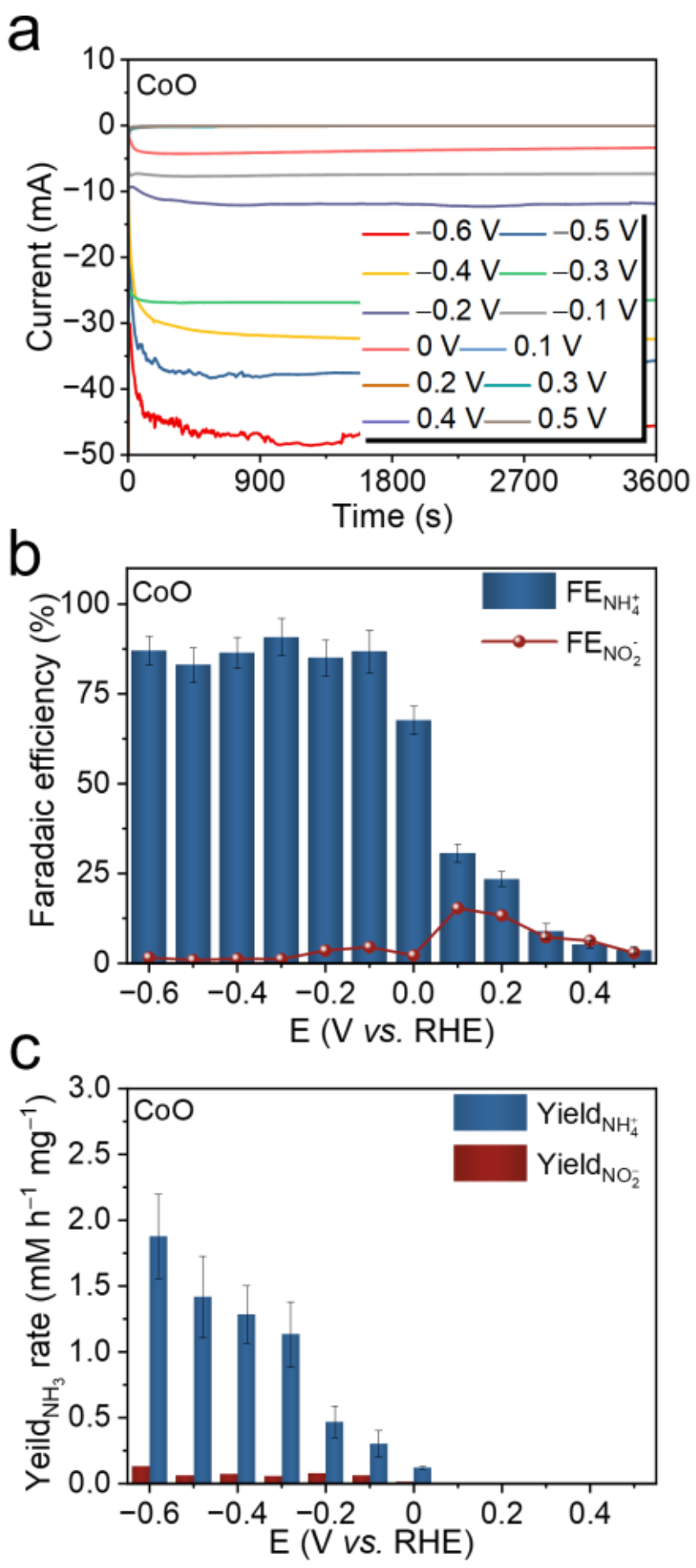

Supplementary Figure 39. Electrochemical nitrate reduction reaction performance. (a) Chronoamperometric (i–t) profiles of CoO measured in 0.1 M KOH + 0.1 M KNO_3 electrolyte at graded constant applied potentials from −0.6 V to 0.5 V *vs.* reversible hydrogen electrode (RHE). Each electrolysis measurement is conducted for a total duration of 3600 s. (b) Faradaic efficiencies of NH_4^+ (blue columns) and NO_2^- (red line with circular markers) over CoO at different applied potentials. All error bars represent the standard deviation calculated from three independent controlled-potential electrolysis replicate experiments. (c) Yield rates of NH_4^+ (blue columns) and NO_2^- (red columns) over CoO at different applied potentials. All error bars correspond to the standard deviation derived from three independent quantitative product detection measurements. Source data are provided as a Source Data file.

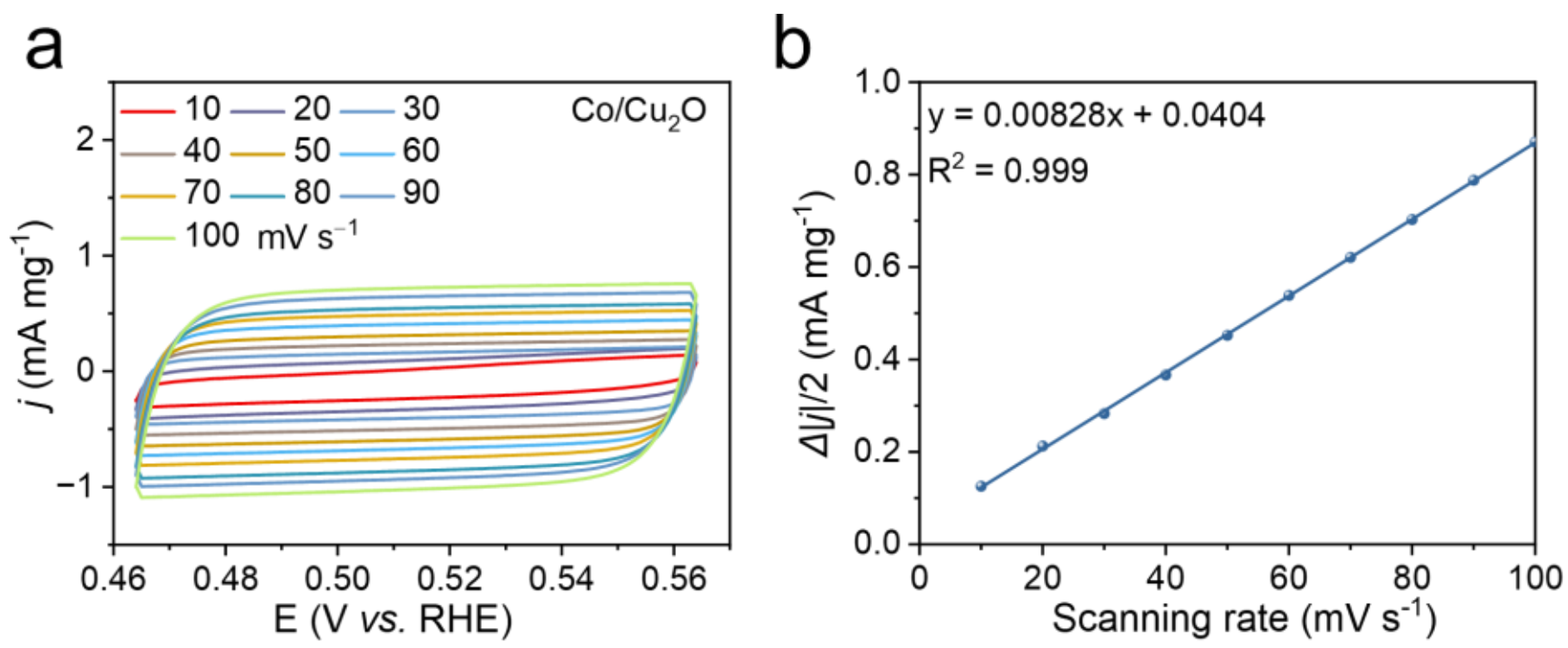

Supplementary Figure 40. Electrochemical double-layer capacitance measurement. (a) Cyclic voltammetry (CV) profiles of the Co/Cu_2O catalyst. (b) Linear fitting plot of half the anodic-cathodic current difference against scan rate. Source data are provided as a Source Data file.

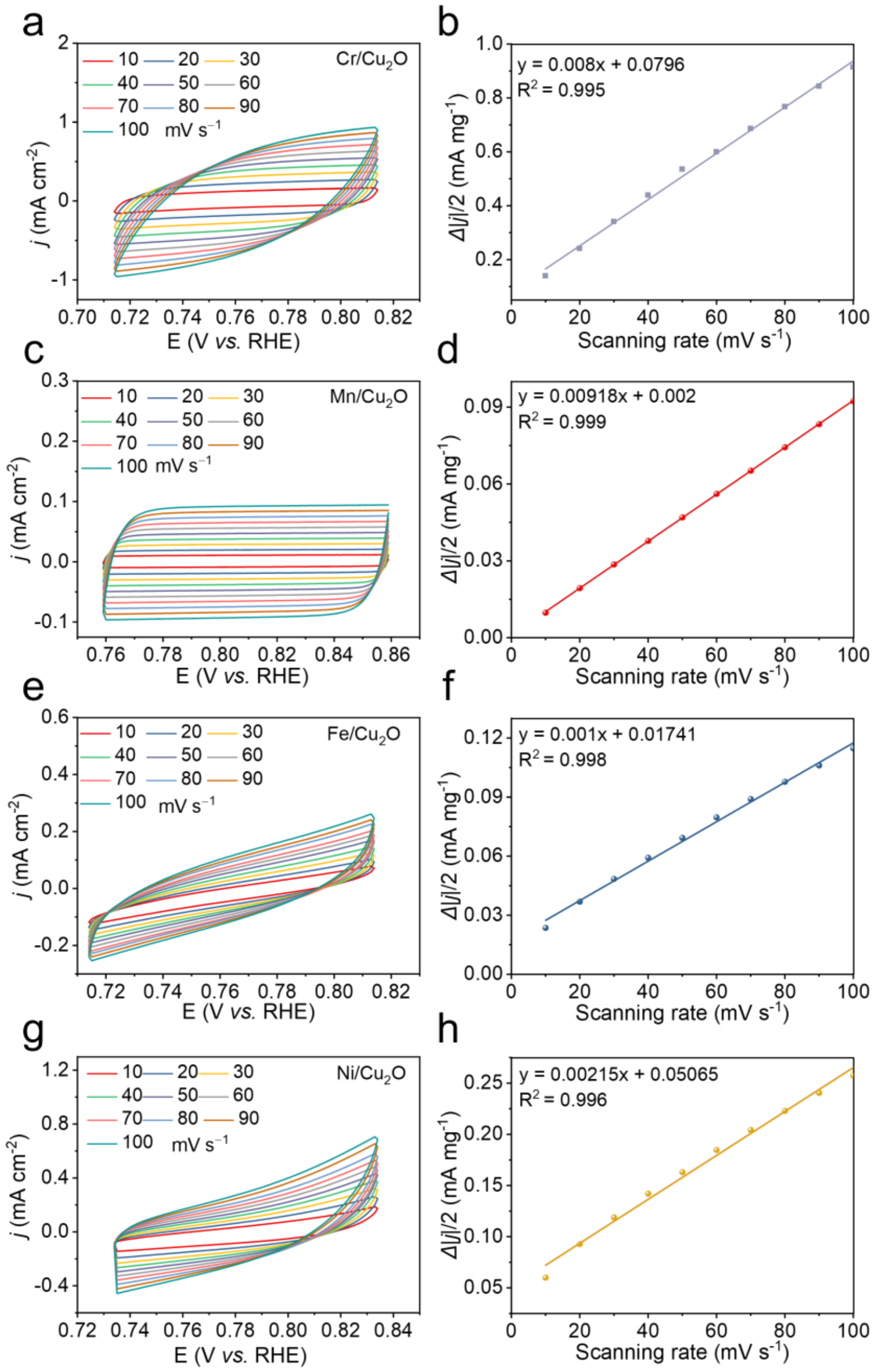

Supplementary Figure 41. Electrochemical double-layer capacitance measurement. The cyclic voltammetry of double layer capacitance of (a,b) Cr/Cu_2O, (c,d) Mn/Cu_2O, (e,f) Fe/Cu_2O, and (g,h) Ni/Cu_2O and its relation to scanning rate, respectively. Source data are provided as a Source Data file.

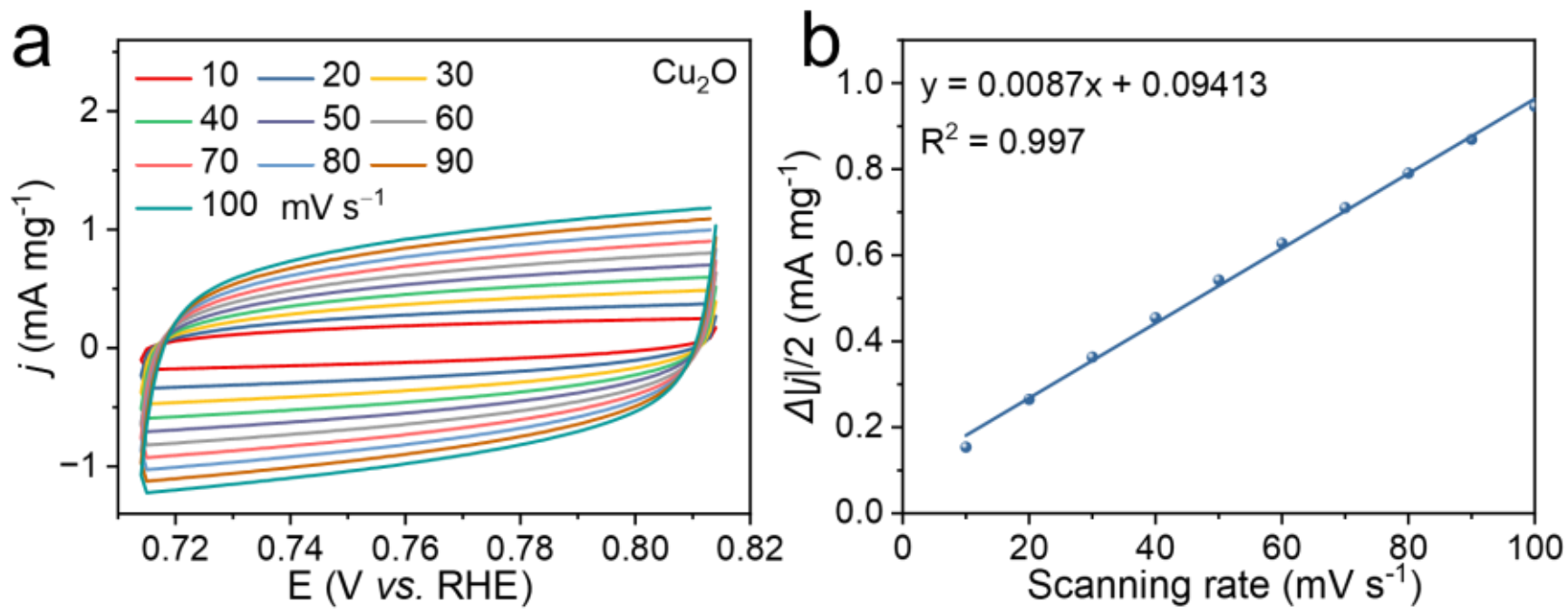

Supplementary Figure 42. Electrochemical double-layer capacitance measurement. (a) Cyclic voltammetry (CV) profiles of the Cu_2O catalyst. (b) Linear fitting plot of half the anodic-cathodic current difference against scan rate. Source data are provided as a Source Data file.

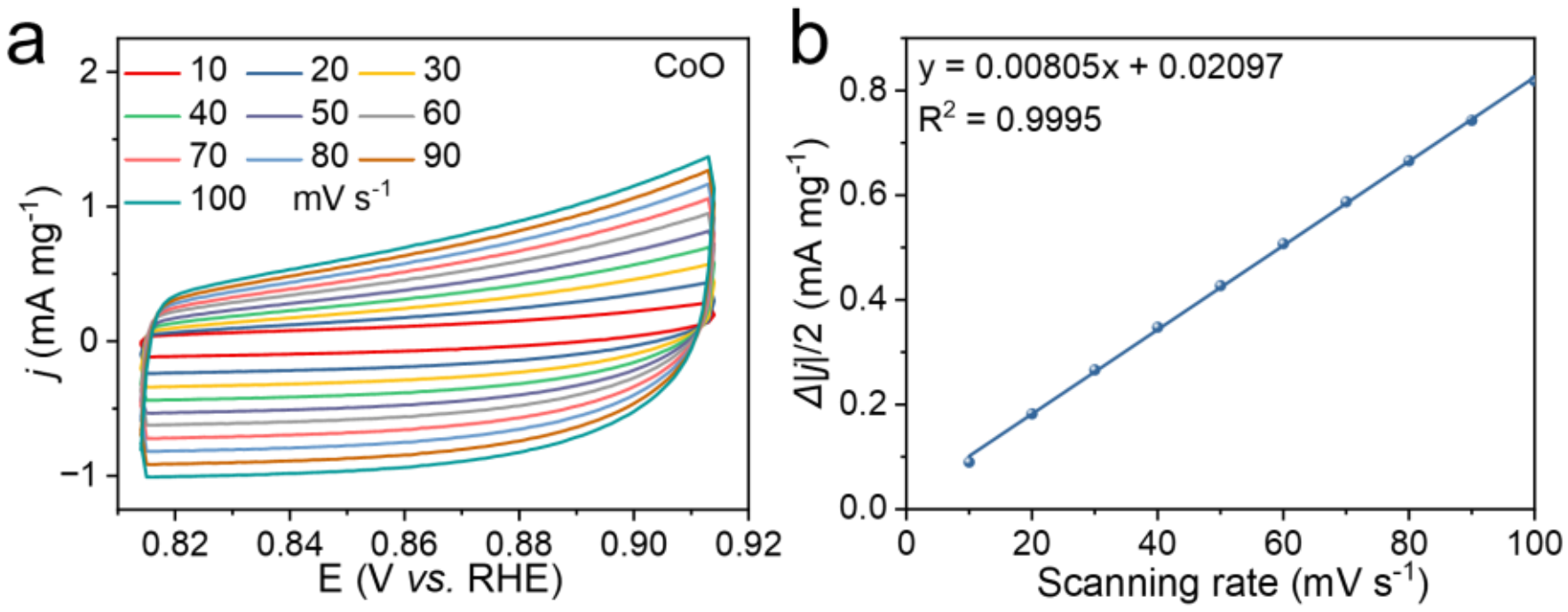

Supplementary Figure 43. Electrochemical double-layer capacitance measurement. (a) Cyclic voltammetry (CV) profiles of the CoO catalyst. (b) Linear fitting plot of half the anodic-cathodic current difference against scan rate. Source data are provided as a Source Data file.

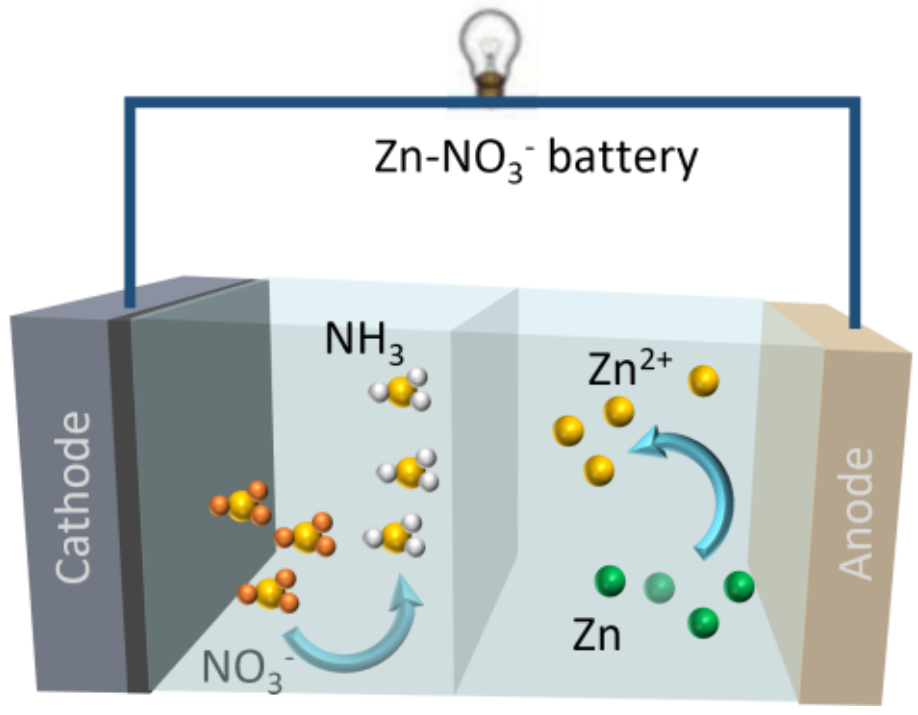

Supplementary Figure 44. Schematic diagram of the Zn-NO_3^- battery. This schematic illustrates the Zn-NO_3^- battery configuration, where Zn anode undergoes oxidation to Zn^{2+} while NO_3^- is reduced to NH_3 at the cathode, simultaneously generating electricity.

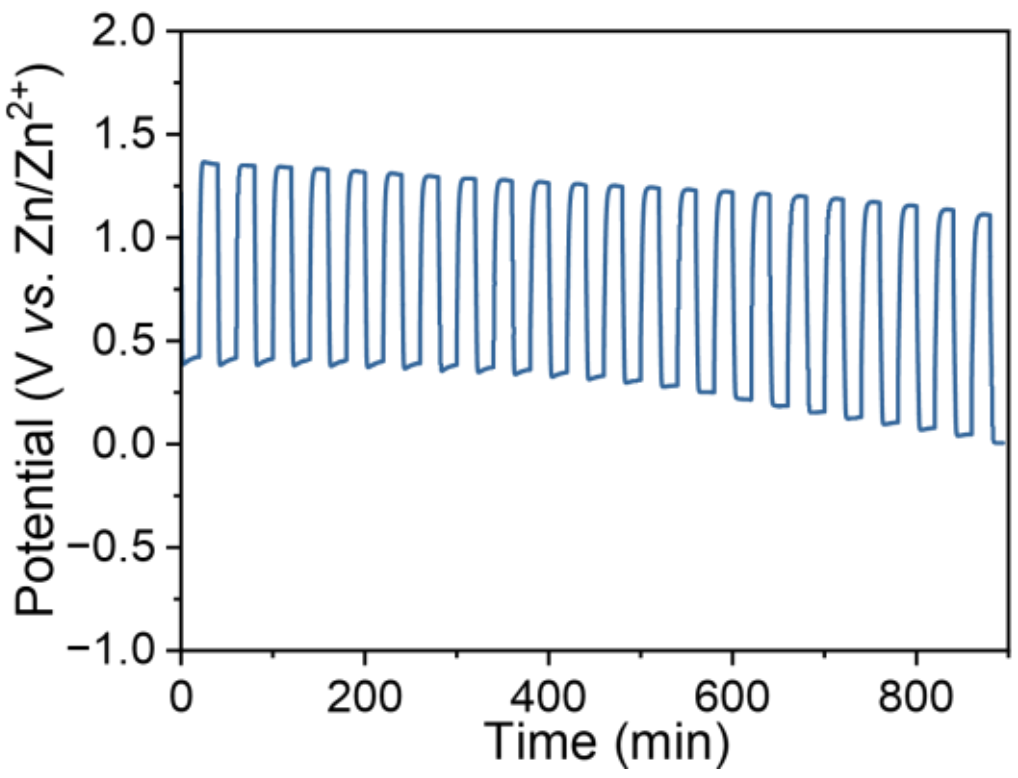

Supplementary Figure 45. Galvanostatic discharge−charge profiles of Co/Cu_2O at 2.0 mA cm^{-2}. Source data are provided as a Source Data file.

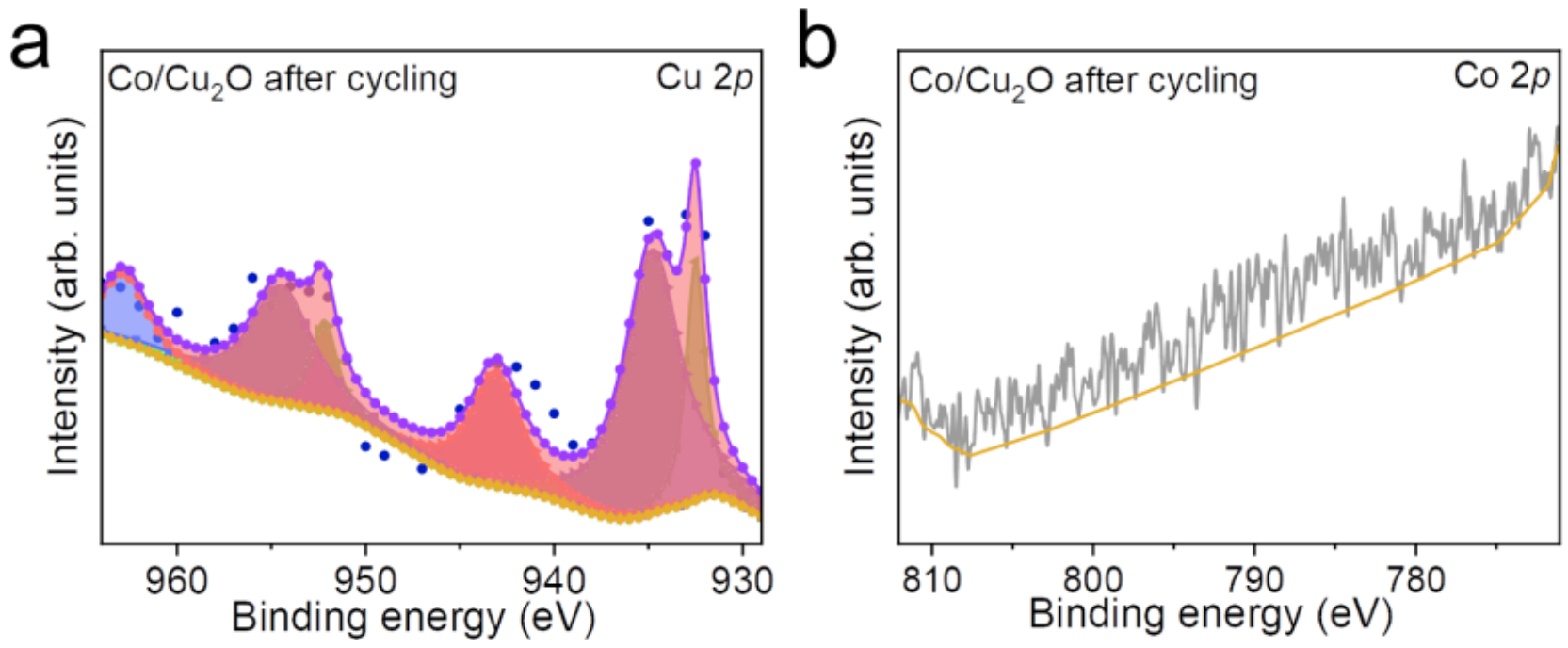

Supplementary Figure 46. High-resolution X-ray photoelectron spectroscopy characterization. (a) Cu 2*p* XPS spectrum and (b) Co 2*p* XPS spectrum of Co/Cu_2O after cycling test. Source data are provided as a Source Data file.

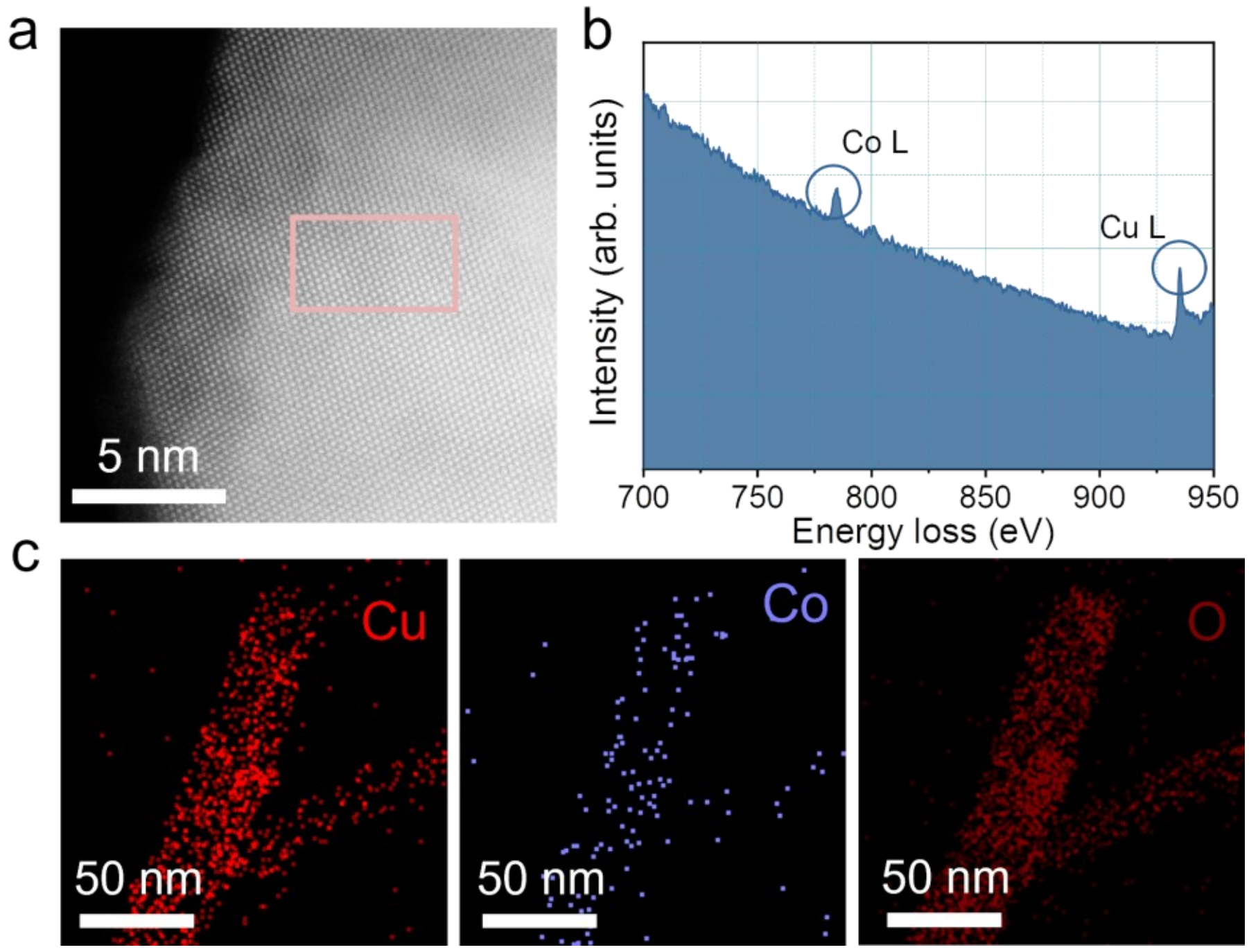

Supplementary Figure 47. Electron microscopic and elemental characterization. (a) High-angle annular dark-field scanning transmission electron microscopy (HADDF-STEM) image, (b) Electron energy loss spectroscopy (EELS), and (c) Energy-dispersive X-ray spectroscopy (EDS) mapping of Co/Cu_2O after cycling test. Source data are provided as a Source Data file.

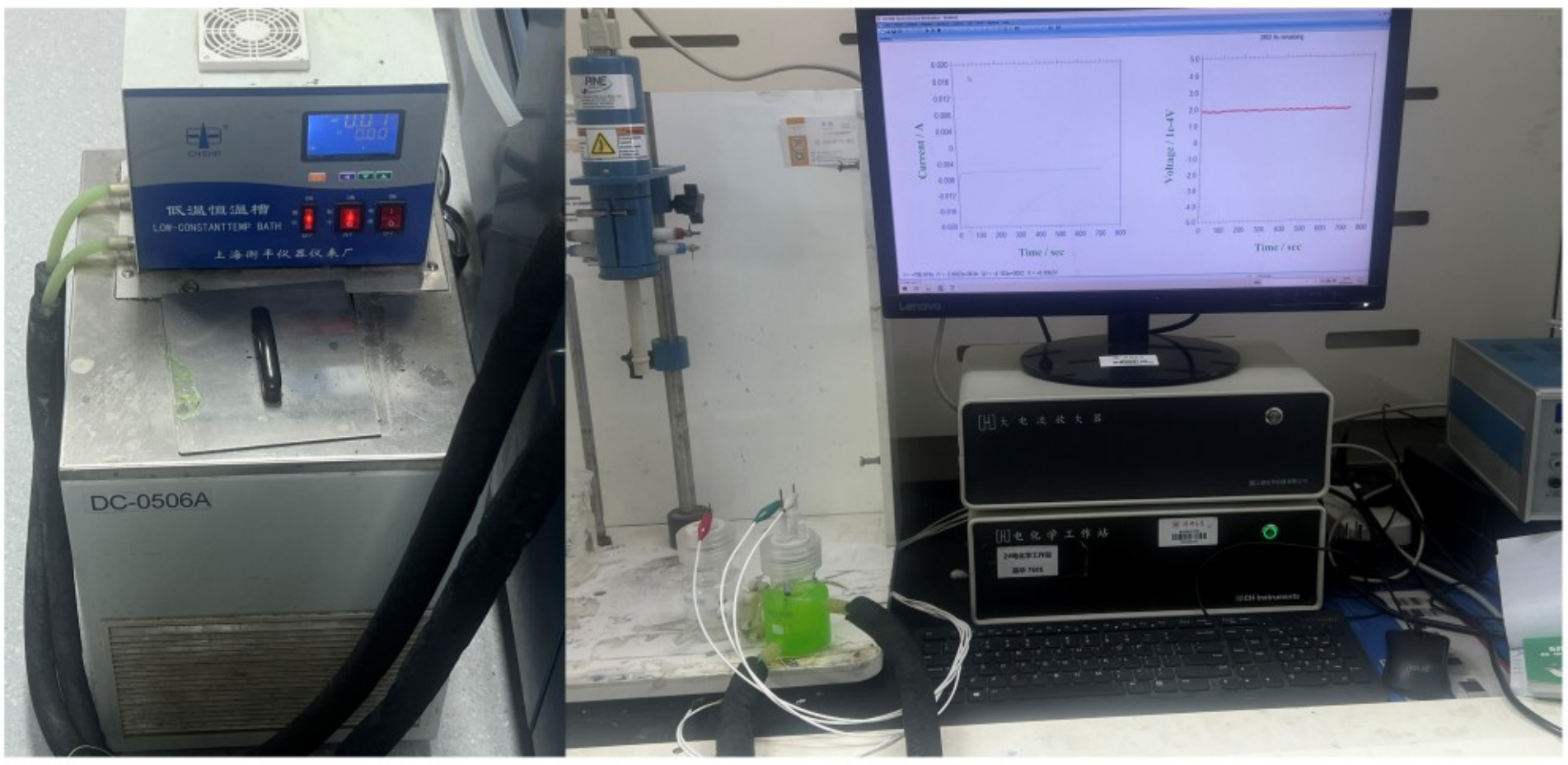

Supplementary Figure 48. A digital photo of the temperature-controlled electrochemical setup at different temperatures. This experimental setup integrates a DC-0506A precision constant-temperature circulator and a high-current electrochemical workstation for variable-temperature electrocatalytic characterization. The thermostat accurately modulates cell temperature, and the workstation collects real-time current and voltage signals visualized on the monitor. This platform facilitates systematic investigation of temperature effects on nitrate reduction kinetics and reaction activation energy.

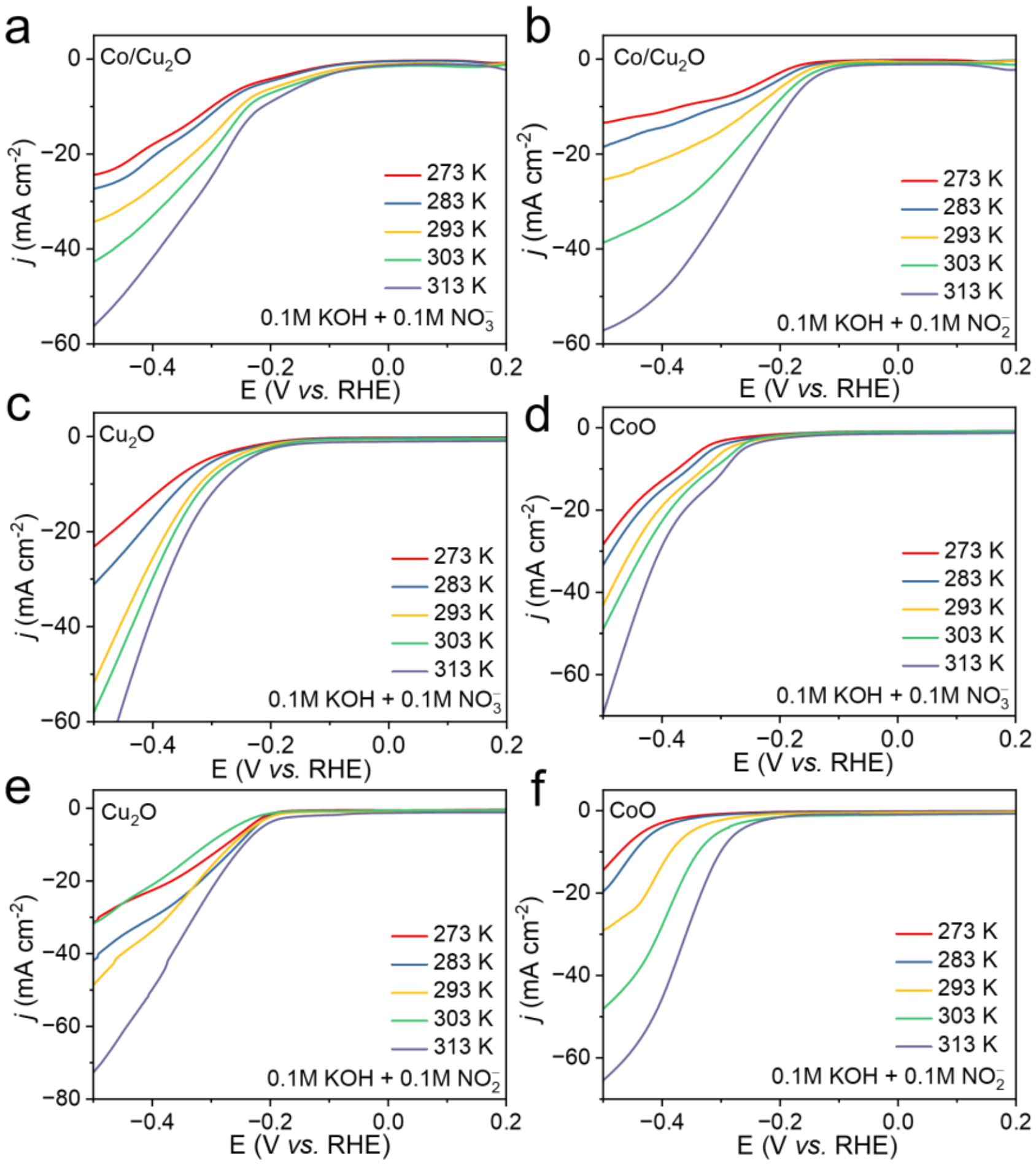

Supplementary Figure 49. Temperature-dependent linear sweep voltammetry (LSV) curves. LSV curves of (a) Co/Cu_2O, (c) Cu_2O, and (e) CoO in 0.1 M KOH + 0.1 M NO_3^- and (b) Co/Cu_2O, (d) Cu_2O, and (f) CoO in 0.1 M KOH + 0.1 M NO_2^- with a scan rate of 5.0 mV s^{-1} at 273, 283, 298, 303, and 313 K, respectively. LSV results reveal rising cathodic reduction current for all three catalysts as temperature increases from 273 K to 313 K in both NO_3^- and NO_2^- electrolytes. Co/Cu_2O consistently achieves larger current densities than pristine Cu_2O and CoO. Higher temperature reduces reaction activation barrier and accelerates intermediate conversion, confirming Co doping prominently improves intrinsic catalytic kinetics toward nitrate/nitrite reduction. Source data are provided as a Source Data file.

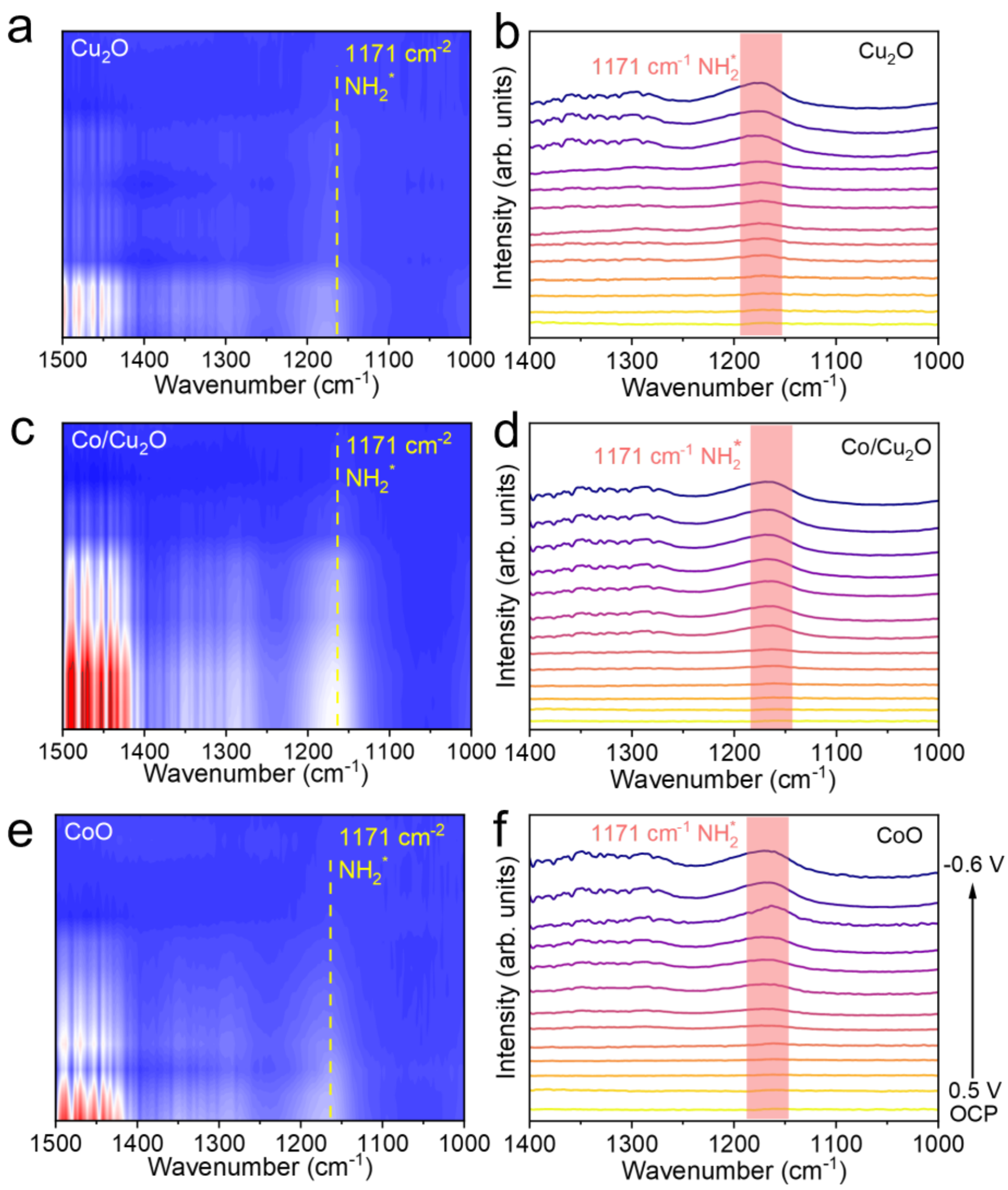

Supplementary Figure 50. *In-situ* Fourier transform infrared (FTIR). *In-situ* FTIR plots of (a,b) Cu_2O, (c,d) Co/Cu_2O, and (e,f) CoO in 0.1 M KOH + 0.1 M NO_3^-, respectively. Source data are provided as a Source Data file.

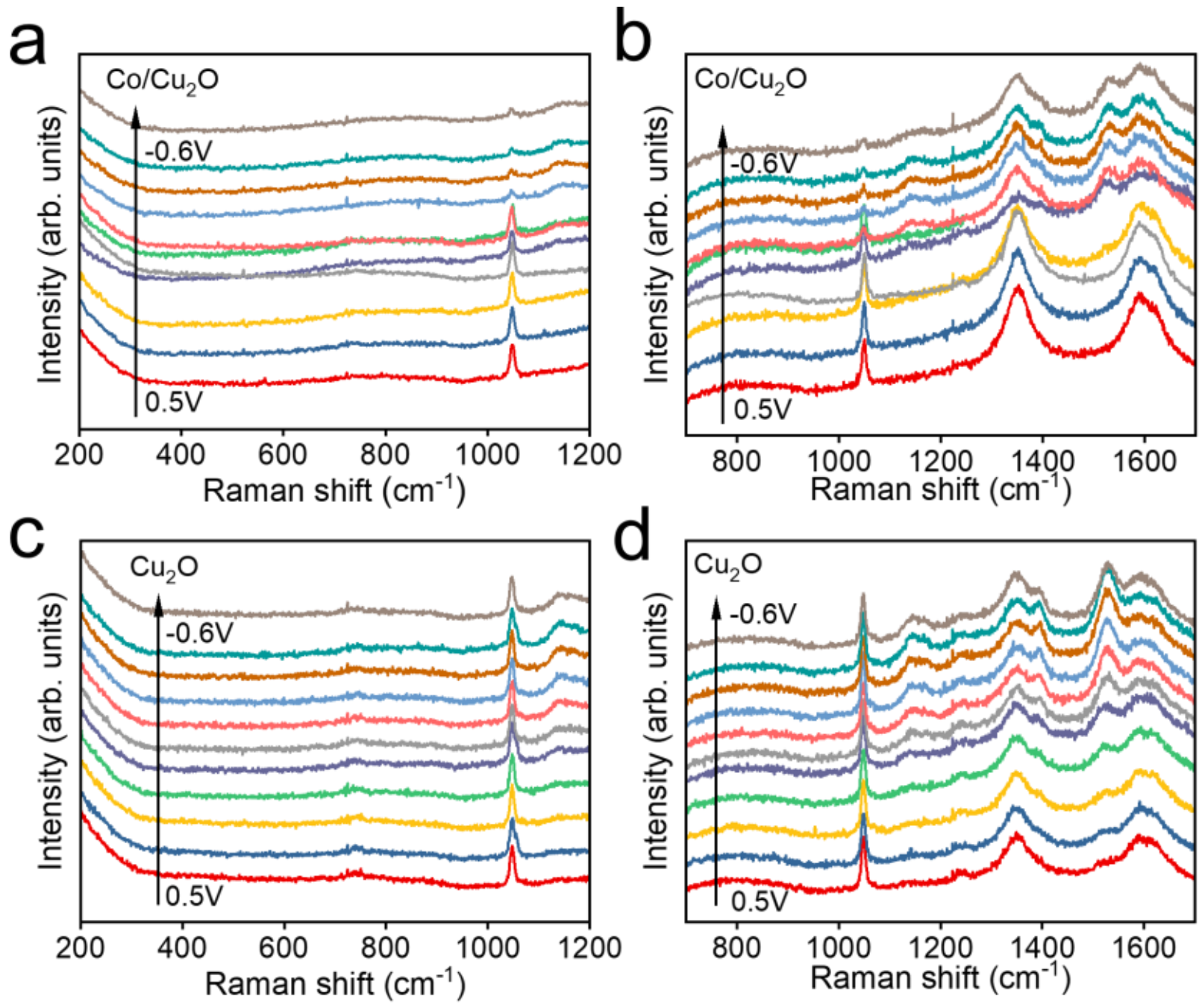

Supplementary Figure 51. *In-situ* Raman spectroscopic characterization. *In-situ* Raman spectra of (a,b) Co/Cu_2O and (c,d) Cu_2O at different applied potentials for NO_3RR, respectively. Source data are provided as a Source Data file.

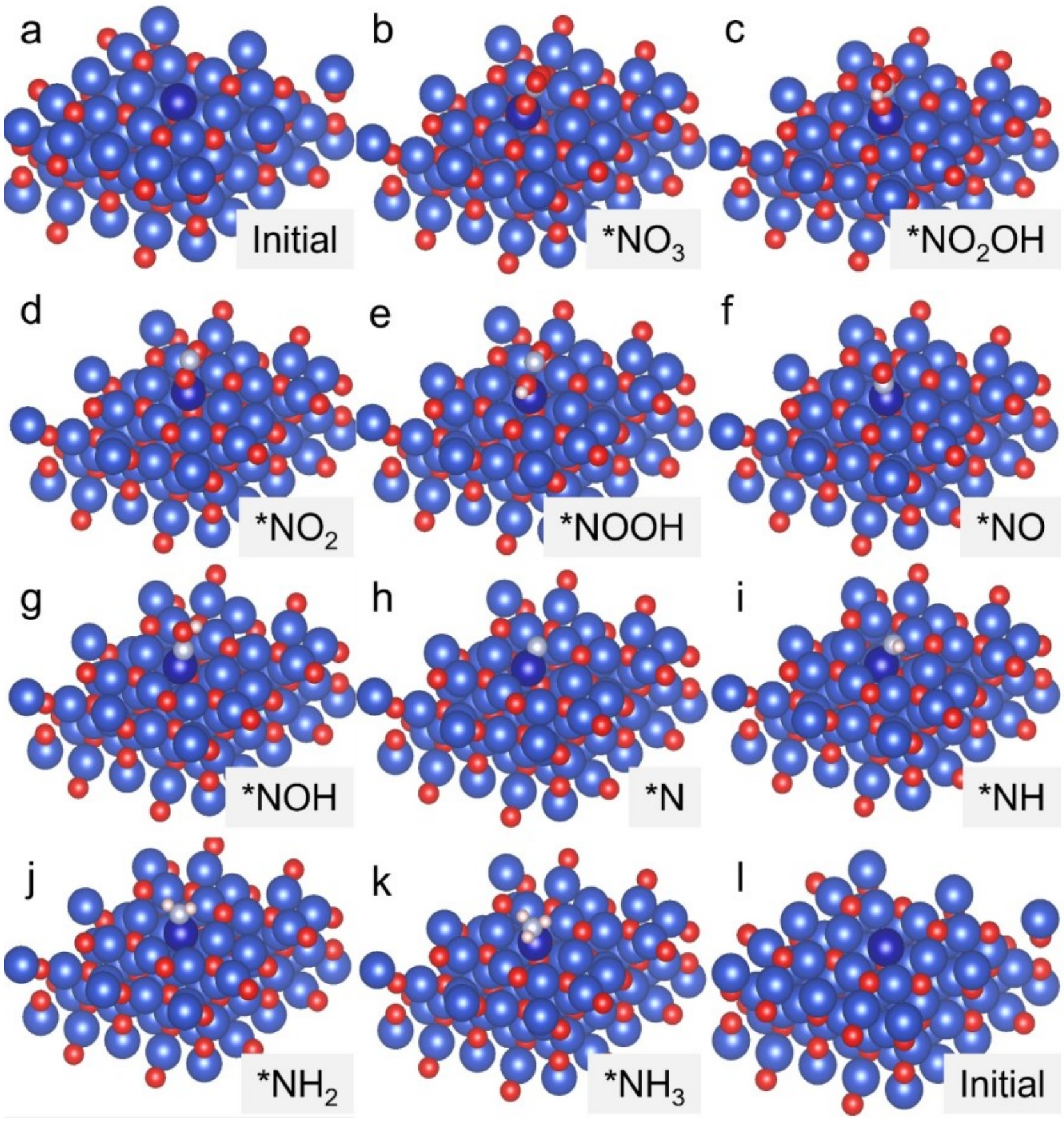

Supplementary Figure 52. Atomic structural models. Adsorption models of different intermediates on Co/Cu_2O surface. These structural models display sequential adsorption configurations of all critical intermediates ranging from *NO_3 to *NH_3 during stepwise nitrate reduction on the Co/Cu_2O surface. The doped Co active center modulates the binding affinity of each adsorbed intermediate and drives successive deoxygenation and protonation processes. This favorable adsorption behavior fundamentally accelerates the full conversion of nitrate into ammonia and rationalizes the favorable adsorption behavior of Co/Cu_2O.

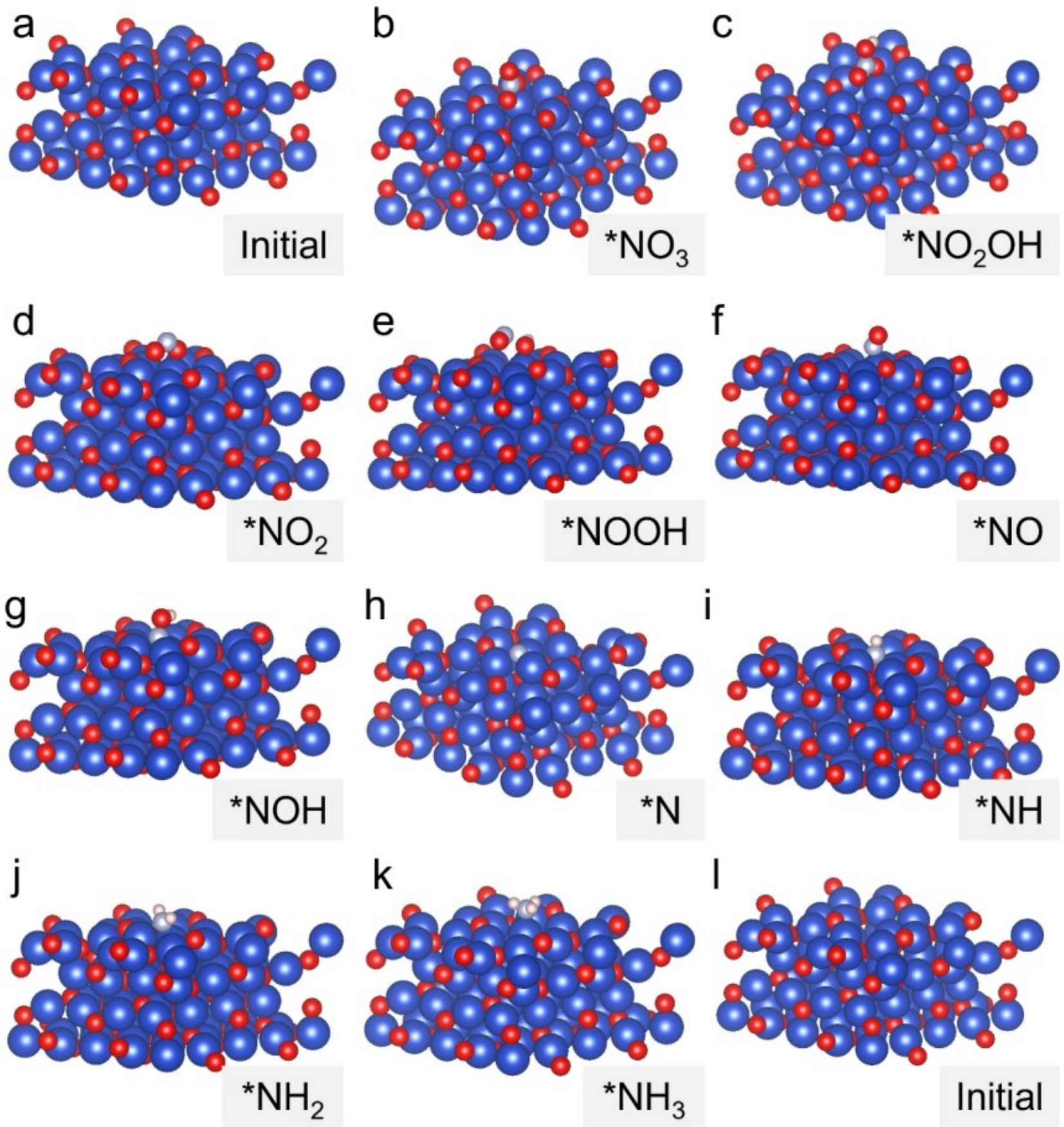

Supplementary Figure 53. Atomic structural models. Adsorption models of different intermediates on Cu_2O surface. These structural models illustrate sequential adsorption configurations of all critical nitrate-reduction intermediates from $*NO_3$ to $*NH_3$ on Cu_2O. Without Co dopant to modulate electronic properties, bare Cu sites exhibit non-optimal binding affinities for these reactive species. Such mismatched adsorption elevates reaction energy barriers, resulting in inferior electrocatalytic NO_3^- to NH_3 performance compared to Co-modified Co/Cu_2O.

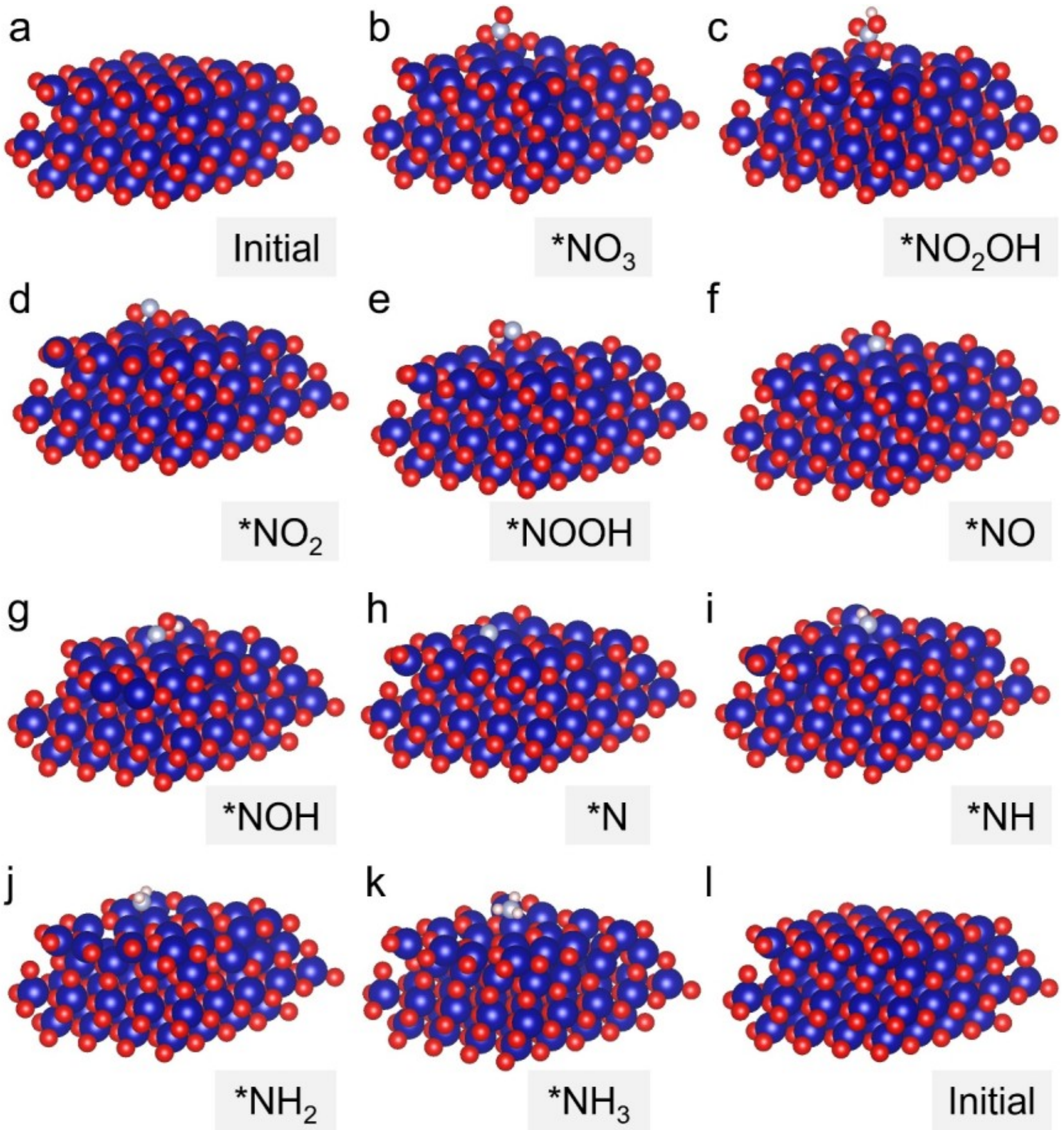

Supplementary Figure 54. Atomic structural models. Adsorption models of different intermediates on CoO surface. These configurations present sequential adsorption geometries of all key nitrate-reduction intermediates from $*NO_3$ to $*NH_3$ on bare CoO surface. Lacking electronic modulation from the Cu host lattice, pure Co sites exhibit mismatched binding affinities toward reactive intermediates. Such inappropriate adsorption elevates energy barriers for successive protonation and deoxygenation steps, resulting in inferior electrocatalytic activity compared with the Co/Cu_2O hybrid catalyst.

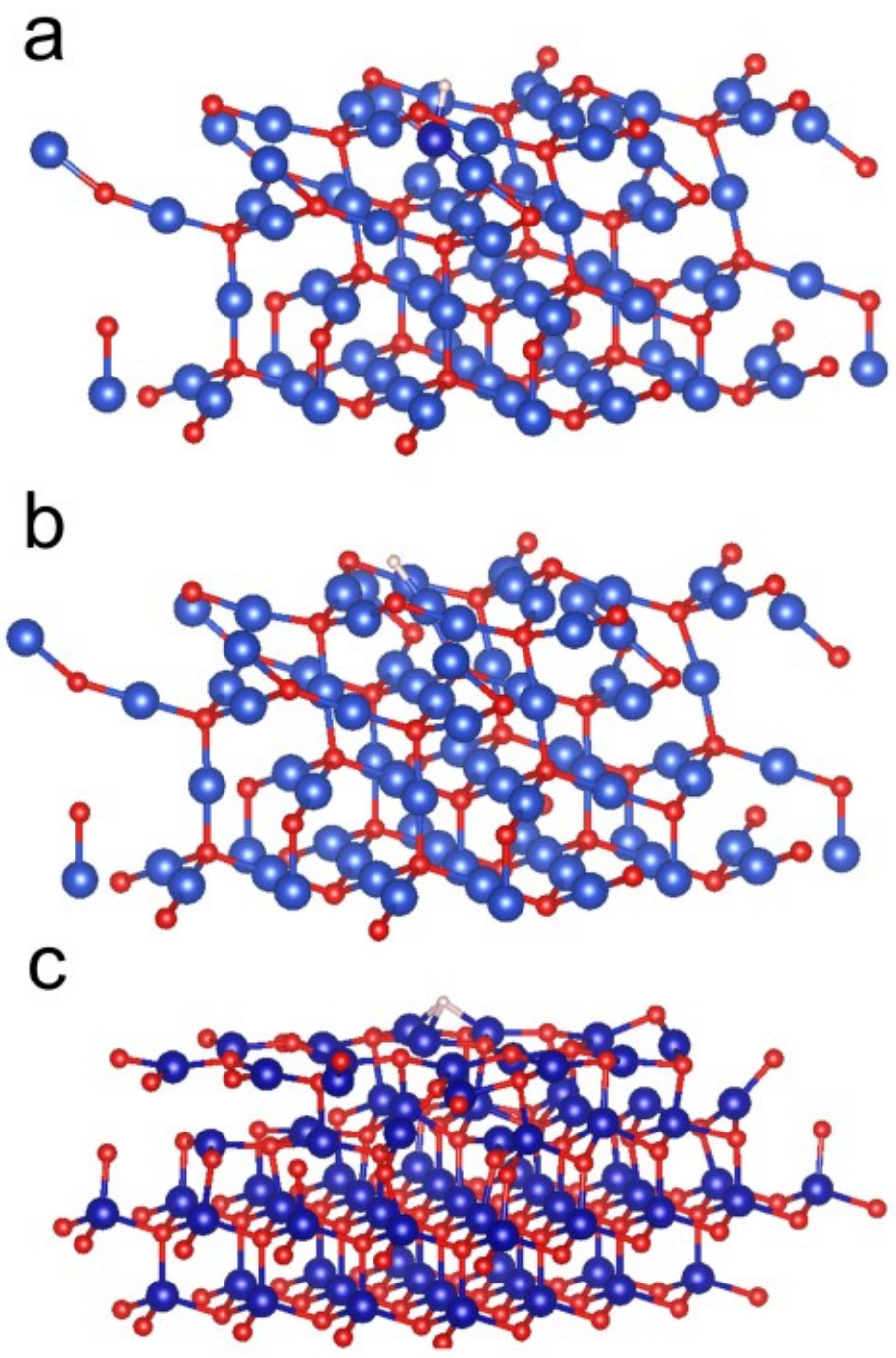

Supplementary Figure 55. Geometry atomic slab models. Adsorption models of H* on different models surface of (a) Co/Cu_2O, (b) Cu_2O, and (c) CoO, respectively. These geometric structures depict H* adsorption configurations over Co/Cu_2O, Cu_2O, and CoO surfaces. Co doping tunes electronic distribution of the host lattice and achieves moderate H* binding, effectively suppressing competitive hydrogen evolution. In contrast, improper H* adsorption on Cu_2O and CoO favors parasitic HER, consuming active sites and deteriorating nitrate reduction selectivity.

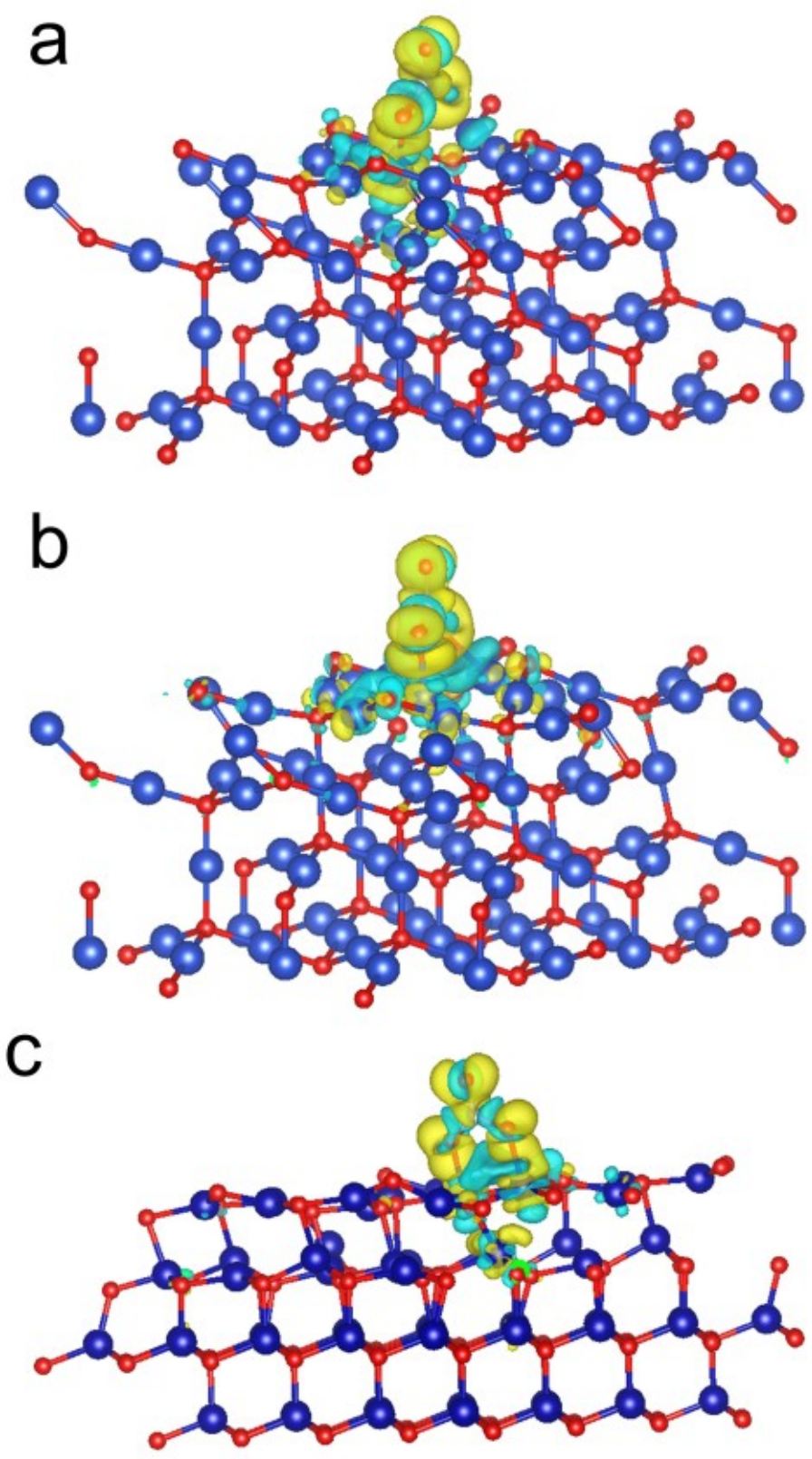

Supplementary Figure 56. Charge density difference. Charge density differences and charge transfer of (a) Co/Cu_2O, (b) Cu_2O, and (c) CoO after adsorption of NO_3^-. Charge density difference diagrams visualize interfacial electron redistribution after NO_3^- adsorption on three catalysts. Co/Cu_2O displays substantially more intensive charge transfer between Co sites and adsorbed nitrate compared with pristine Cu_2O and CoO. Such promoted electron injection efficiently activates nitrate by elongating N−O bonds and greatly accelerates the subsequent NO_3^- to NH_3 electroreduction pathway.

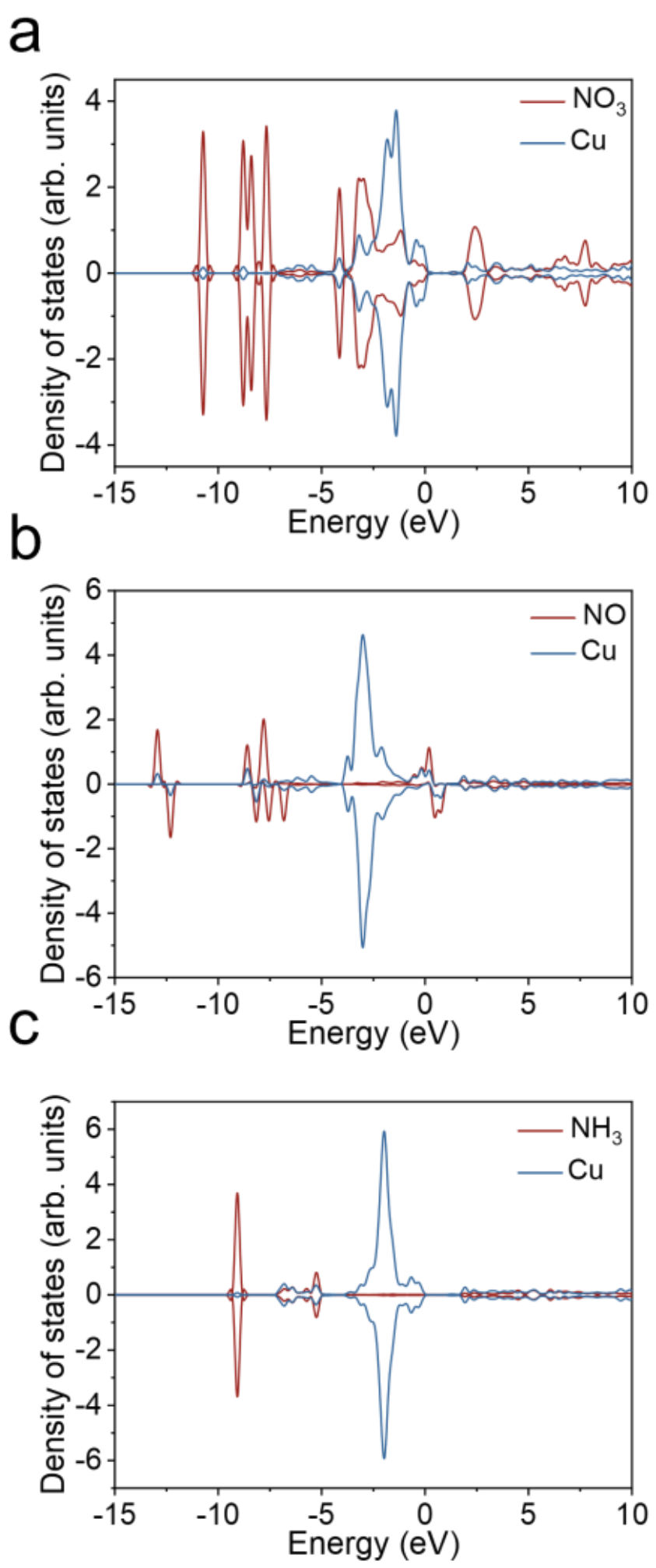

Supplementary Figure 57. Projected density of states (PDOS) analysis. PDOS of Cu 3*d* orbital and (a) *NO_3, (b) *NO, and (c) *NH_3 on Cu_2O, respectively. The PDOS depicts orbital overlap between Cu 3*d* and three key nitrate-reduction intermediates on bare Cu_2O. Distinct mismatches in peak alignment near the Fermi level reveal uneven orbital hybridization for *NO_3, *NO, and *NH_3. Such unoptimized electronic coupling causes inappropriate intermediate binding, raising reaction barriers and limiting the intrinsic catalytic activity of pristine Cu_2O. Source data are provided as a Source Data file.

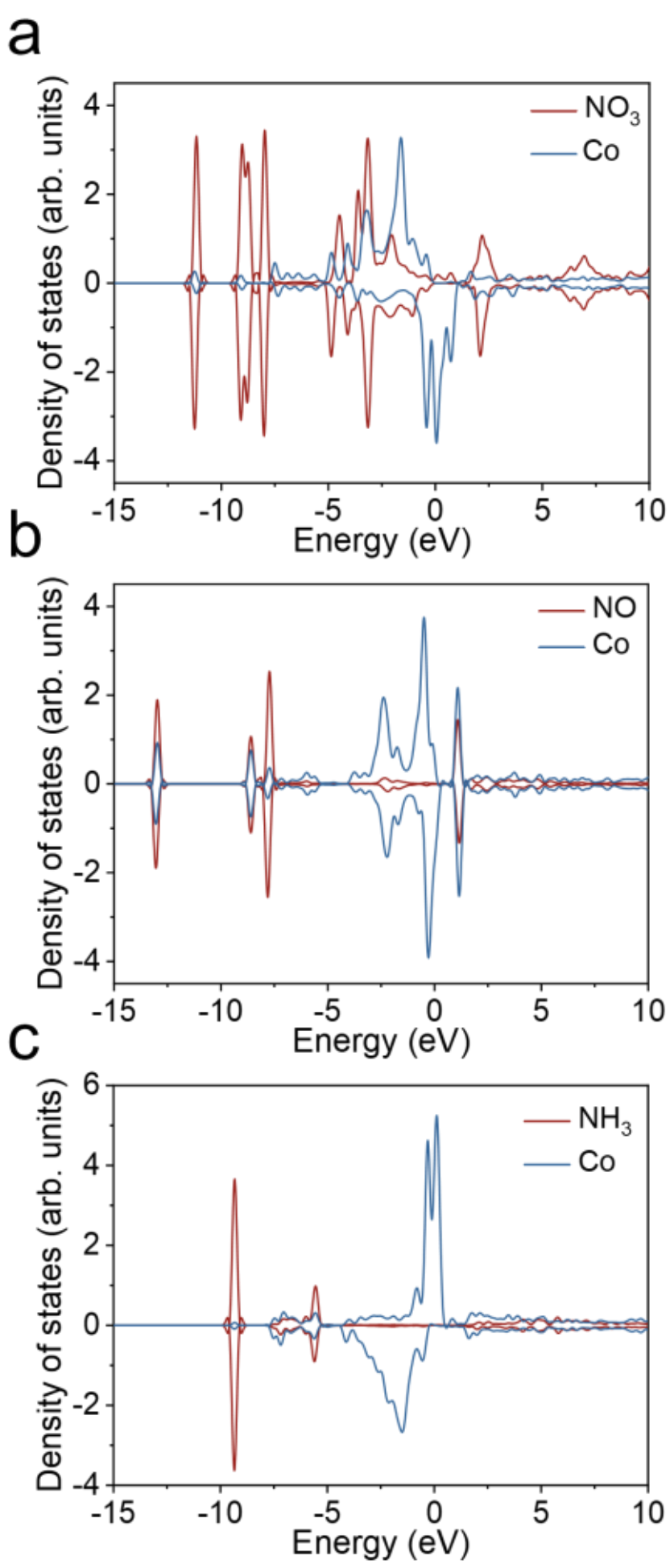

Supplementary Figure 58. Projected density of states (PDOS) analysis. PDOS of Co 3*d* orbital and (a) $*NO_3$, (b) *NO, and (c) $*NH_3$ on CoO, respectively. These PDOS plots reveal orbital hybridization between Co 3*d* orbitals and $*NO_3$, *NO, $*NH_3$ on CoO. Visible mismatched peak overlap near the Fermi level induces unbalanced binding strength for all intermediates. Excessively strong adsorption traps reactive species on Co sites, elevating deprotonation barriers and deteriorating NO_3RR catalytic performance relative to doped Co/Cu_2O. Source data are provided as a Source Data file.

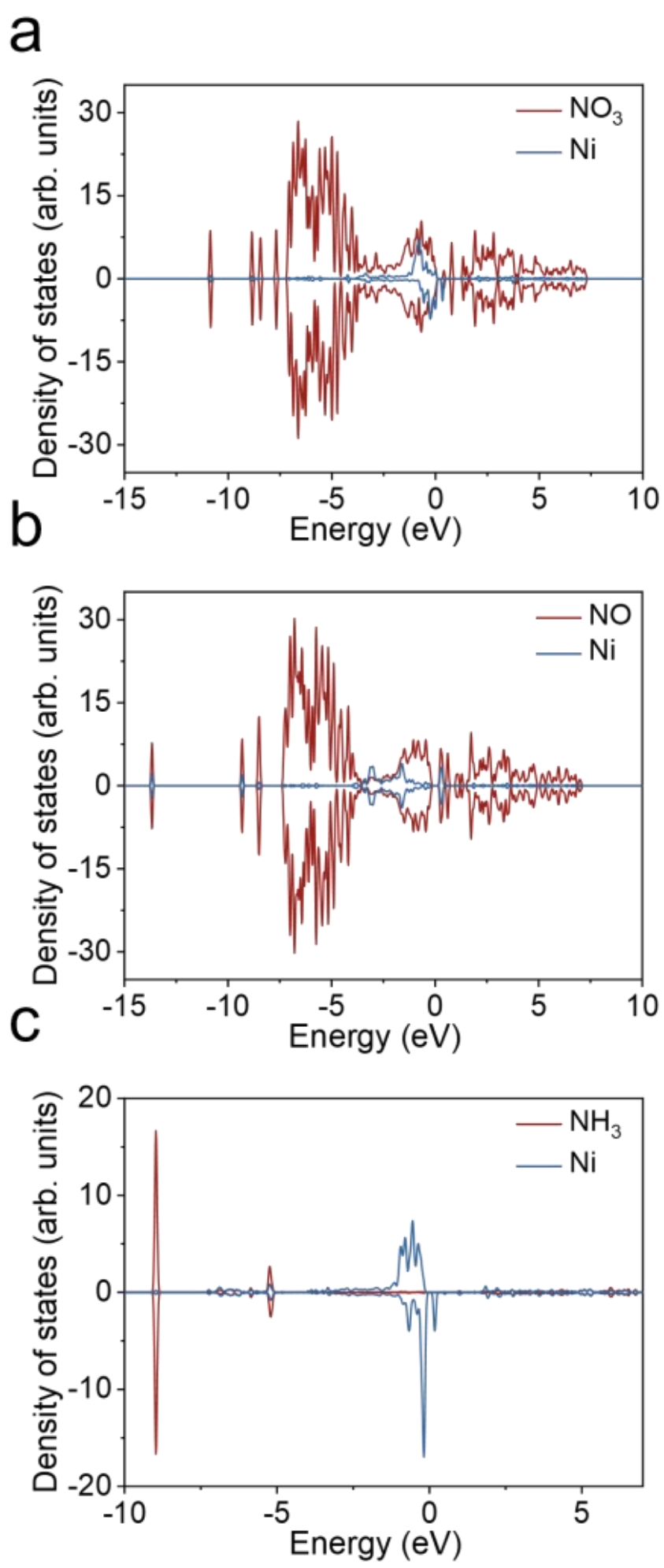

Supplementary Figure 59. Projected density of states (PDOS) analysis. PDOS of Ni 3*d* orbital and (a) $*NO_3$, (b) *NO, and (c) $*NH_3$ on Ni/Cu_2O, respectively. These PDOS plots characterize orbital hybridization between Ni 3*d* orbitals and three critical nitrate-reduction intermediates ($*NO_3$, *NO, $*NH_3$) on Ni-modified Cu_2O. Ni doping effectively modulates electronic structure, creating well-matched orbital overlap near the Fermi level for all adsorbates, especially the rate-determining *NO intermediate. This balanced binding affinity lowers thermodynamic barriers for successive deoxygenation and protonation steps, fundamentally explaining the enhanced electrocatalytic NO_3RR performance of Ni/Cu_2O compared to pristine Cu_2O. Source data are provided as a Source Data file.

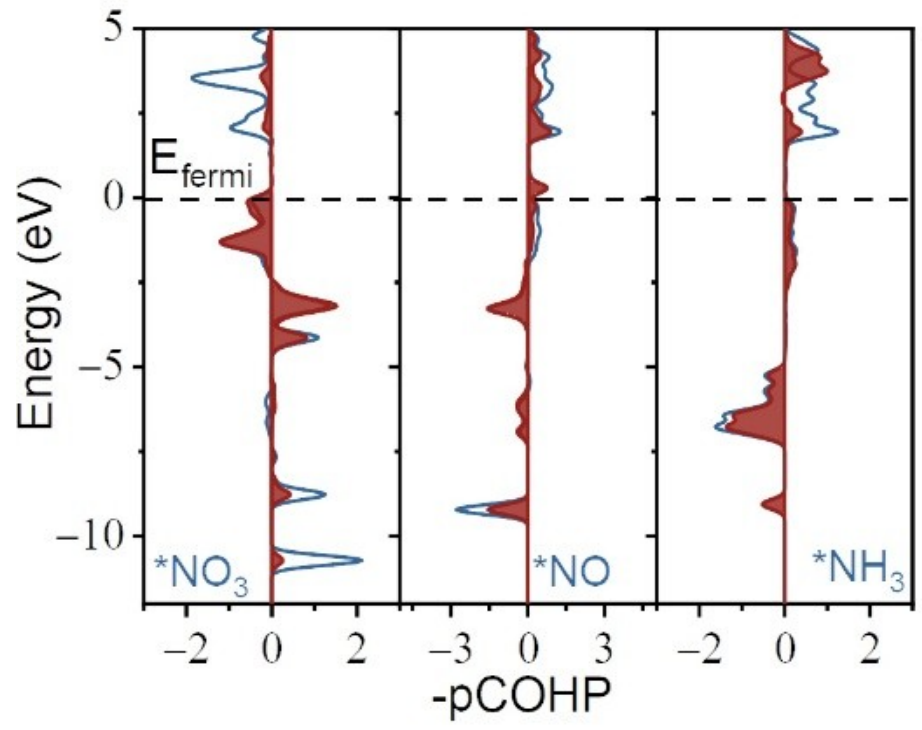

Supplementary Figure 60. Integrated crystal orbital Hamilton population (ICOHP) evolution of *NO_3/Cu 3*d*, *NO/Cu 3*d*, and *NH_3/Cu 3*d* on Cu_2O. The -pCOHP curves characterize orbital bonding between Cu 3*d* orbitals and three key intermediates *NO_3, *NO, *NH_3 on pristine Cu_2O. Distinct variations in bonding peak intensities indicate mismatched orbital overlap and nonideal binding affinity for each adsorbate. Such inappropriate intermediate anchoring increases reaction thermodynamic barriers and restricts the intrinsic NO_3RR performance of bare Cu_2O. Source data are provided as a Source Data file.

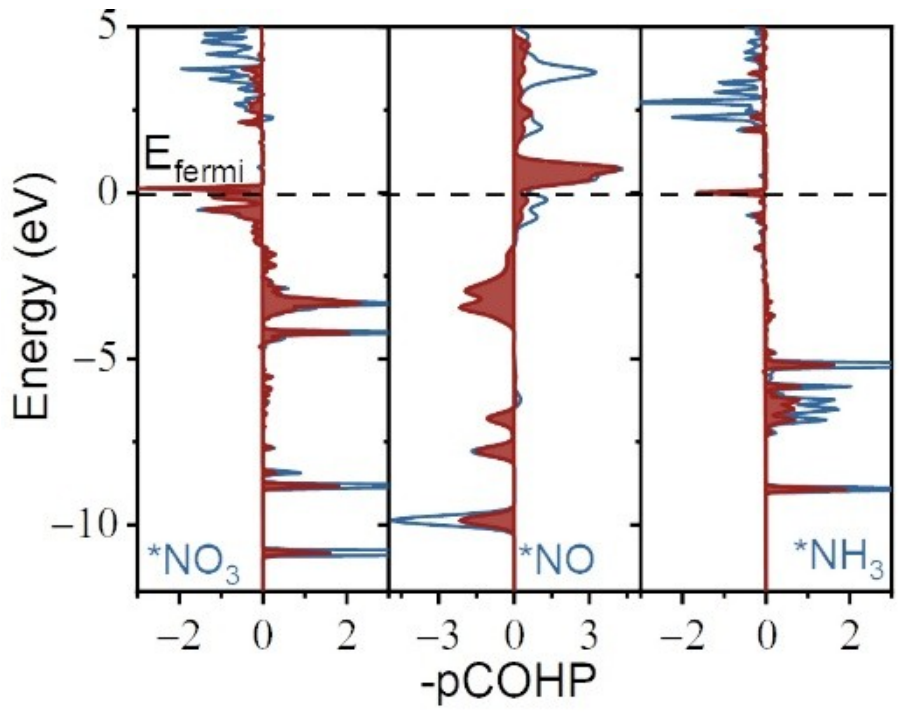

Supplementary Figure 61. Integrated crystal orbital Hamilton population (ICOHP) evolution of $*NO_3$/Ni 3*d*, *NO/Ni 3*d*, and $*NH_3$/Ni 3*d* on Ni/Cu_2O. The -pCOHP spectra unravel orbital hybridization between Ni 3*d* orbitals and three sequential nitrate-reduction intermediates on Ni-modified Cu_2O. Ni doping modulates bonding peak distribution near the Fermi level and optimizes orbital overlap strength for $*NO_3$, *NO, and $*NH_3$. Such well-balanced intermediate binding lowers reaction energy barriers and facilitates smooth stepwise conversion toward ammonia. Source data are provided as a Source Data file.

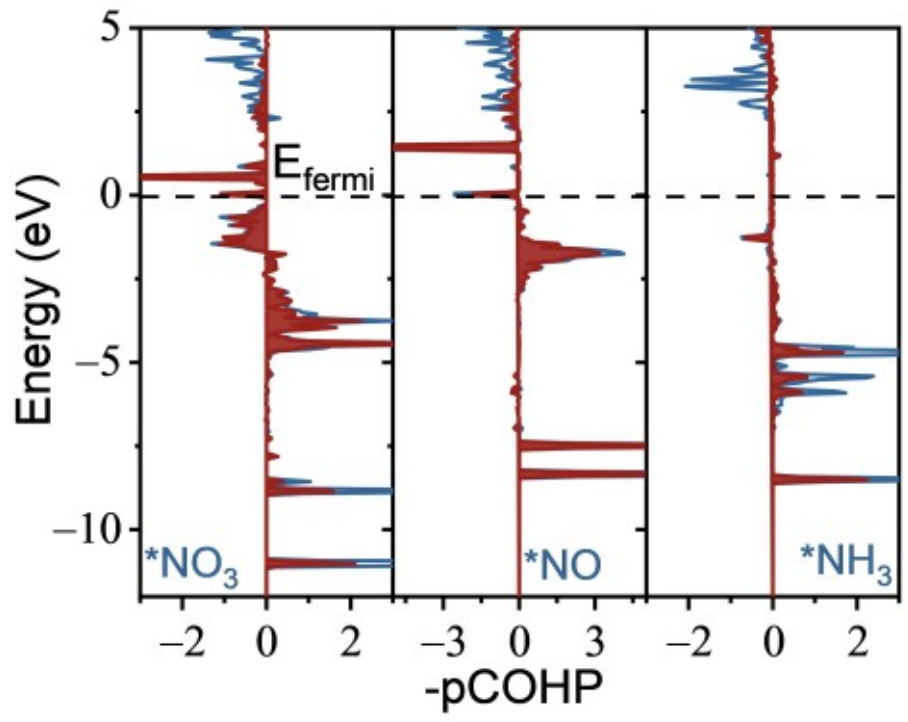

Supplementary Figure 62. Integrated crystal orbital Hamilton population (ICOHP) evolution of *NO_3/Co 3*d*, *NO/Co 3*d*, *NH_3/Co 3*d* on Co/Cu_2O at the GGA+U level with the Hubbard = U 3.3 eV. These GGA+U (U = 3.3 eV) -pCOHP profiles quantify orbital bonding interactions between Co 3*d* orbitals and three key nitrate-reduction intermediates (*NO_3, *NO, *NH_3) on Co/Cu_2O. Positive peaks below the Fermi level represent dominant bonding, with Co doping tuning binding strength to an optimal range for all adsorbates, especially the rate-limiting *NO. Unlike pristine Cu_2O with mismatched adsorption, this balanced coupling avoids weak nitrate activation and severe intermediate poisoning, directly lowering reaction barriers for efficient NO_3RR conversion. Source data are provided as a Source Data file.

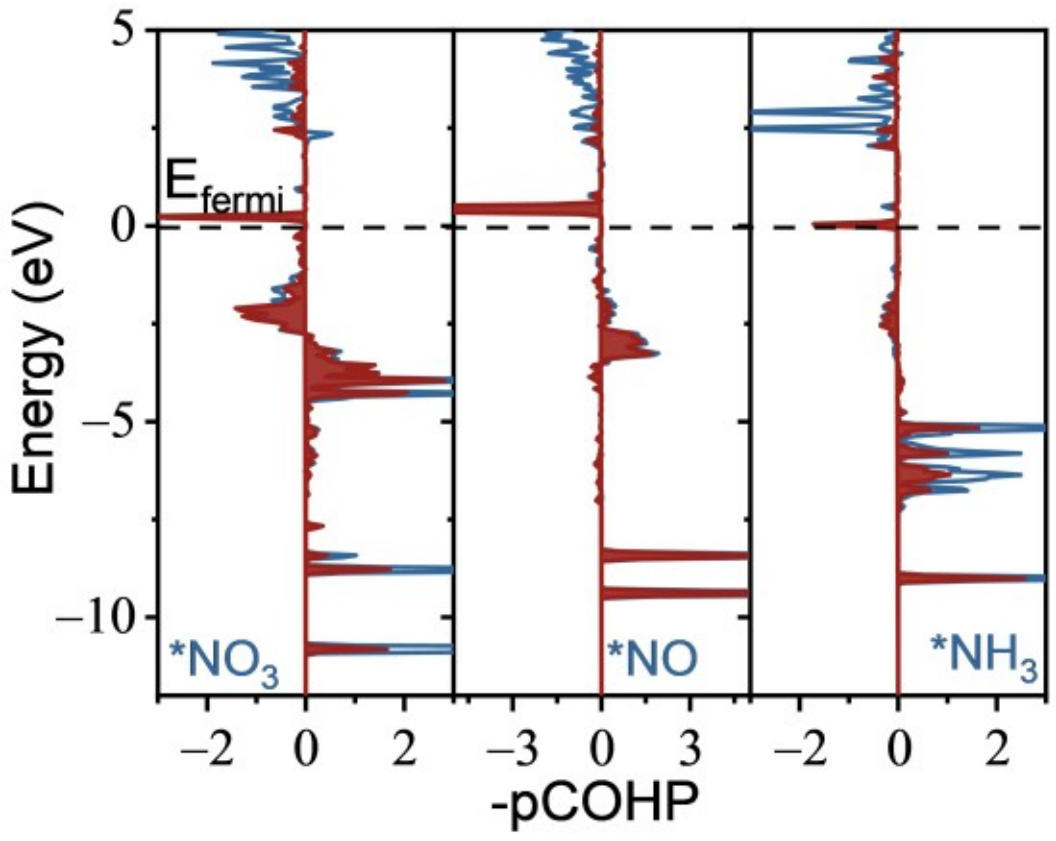

Supplementary Figure 63. Integrated crystal orbital Hamilton population (ICOHP) evolution of $*NO_3$/Ni 3*d*, *NO/Ni 3*d*, $*NH_3$/Ni 3*d* on Ni/Cu_2O at the GGA+U level with the Hubbard U = 6.0 eV. These GGA+U (U = 6.0 eV) -pCOHP profiles quantify orbital bonding between Ni 3*d* orbitals and $*NO_3$, *NO, $*NH_3$ on Ni/Cu_2O. Compared to Co/Cu_2O, Ni sites exhibit marginally stronger binding to the rate-limiting *NO intermediate, raising the deoxygenation barrier slightly. Nevertheless, both doped catalysts deliver far more balanced adsorption than pristine Cu_2O, with Co/Cu_2O still showing the optimal binding energetics for efficient NO_3RR conversion. Source data are provided as a Source Data file.

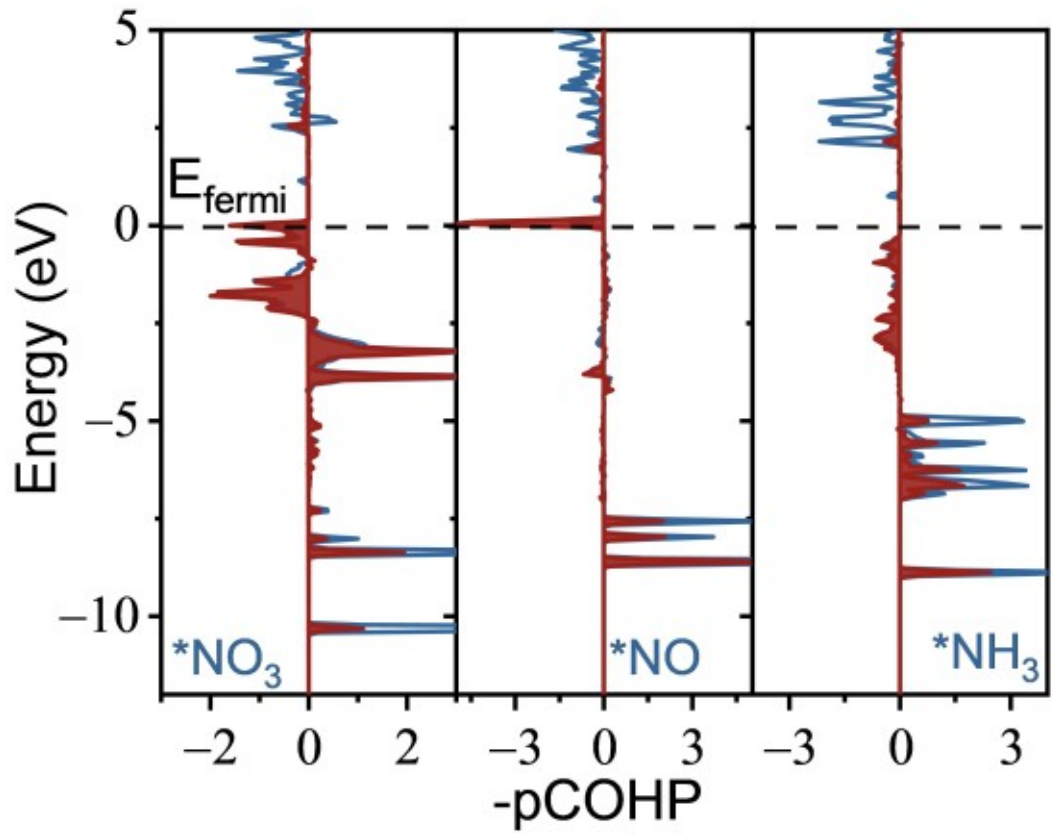

Supplementary Figure 64. Integrated crystal orbital Hamilton population (ICOHP) evolution of *NO_3/Cu 3*d*, *NO/Cu 3*d*, *NH_3/Cu 3*d* on Cu_2O at the GGA+U level with the Hubbard U = 5.0 eV. These GGA+U (U = 5.0 eV) -pCOHP profiles quantify orbital bonding interactions between Cu 3*d* orbitals and *NO_3, *NO, *NH_3 on pristine Cu_2O. Unlike Co/Cu_2O with well-balanced intermediate binding, bare Cu sites exhibit severely mismatched adsorption: excessively weak *NO_3 activation and overly strong *NO trapping. This unbalanced coupling drastically elevates the rate-limiting deoxygenation barrier, directly accounting for the inferior NO_3RR electrocatalytic performance relative to Co-doped Cu_2O. Source data are provided as a Source Data file.

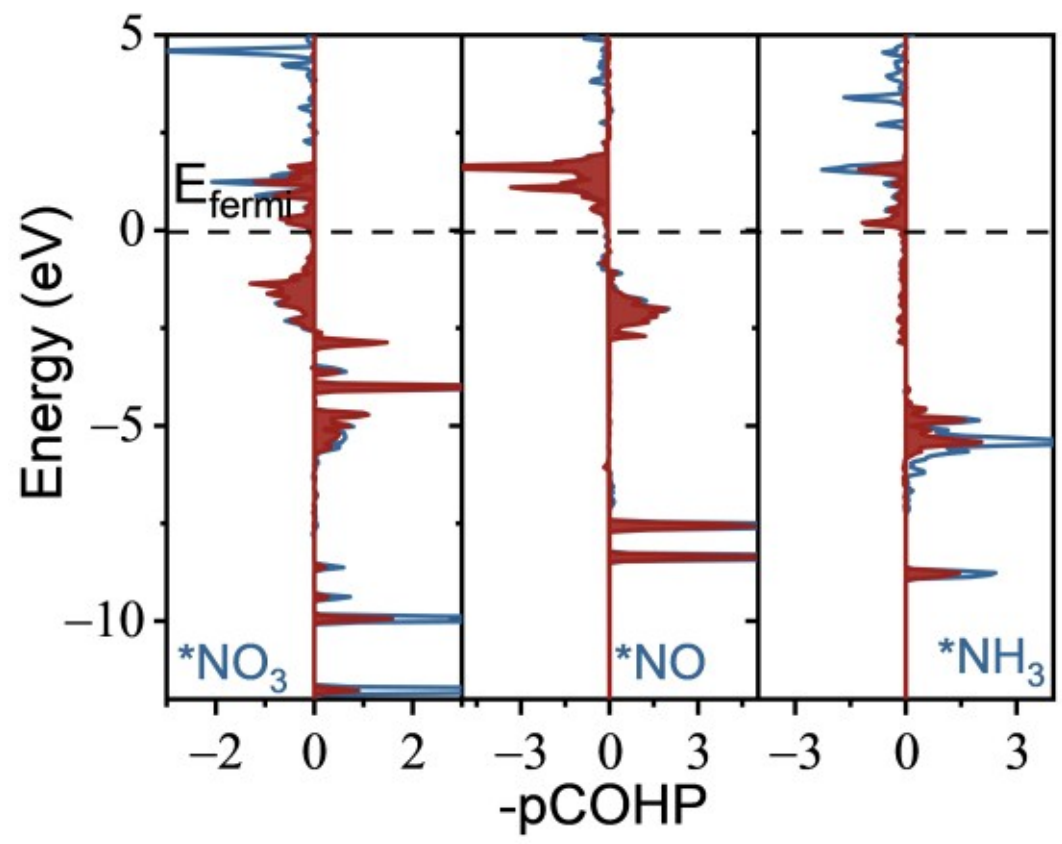

Supplementary Figure 65. Integrated crystal orbital Hamilton population (ICOHP) evolution of $*NO_3$/Co 3*d*, *NO/Co 3*d*, $*NH_3$/Co 3*d* on CoO at the GGA+U level with the Hubbard U = 3.3 eV. These GGA+U (U = 3.3 eV) -pCOHP profiles quantify orbital bonding interactions between Co 3*d* orbitals and $*NO_3$, *NO, $*NH_3$ on pure CoO. Unlike Co/Cu_2O with well-balanced intermediate binding, pure Co sites exhibit excessively strong adsorption for all intermediates, especially the rate-limiting *NO. This overbinding traps reactive species on the surface, severely impeding subsequent protonation and deoxygenation steps, resulting in drastically inferior NO_3RR electrocatalytic performance compared to Co/Cu_2O. Source data are provided as a Source Data file.

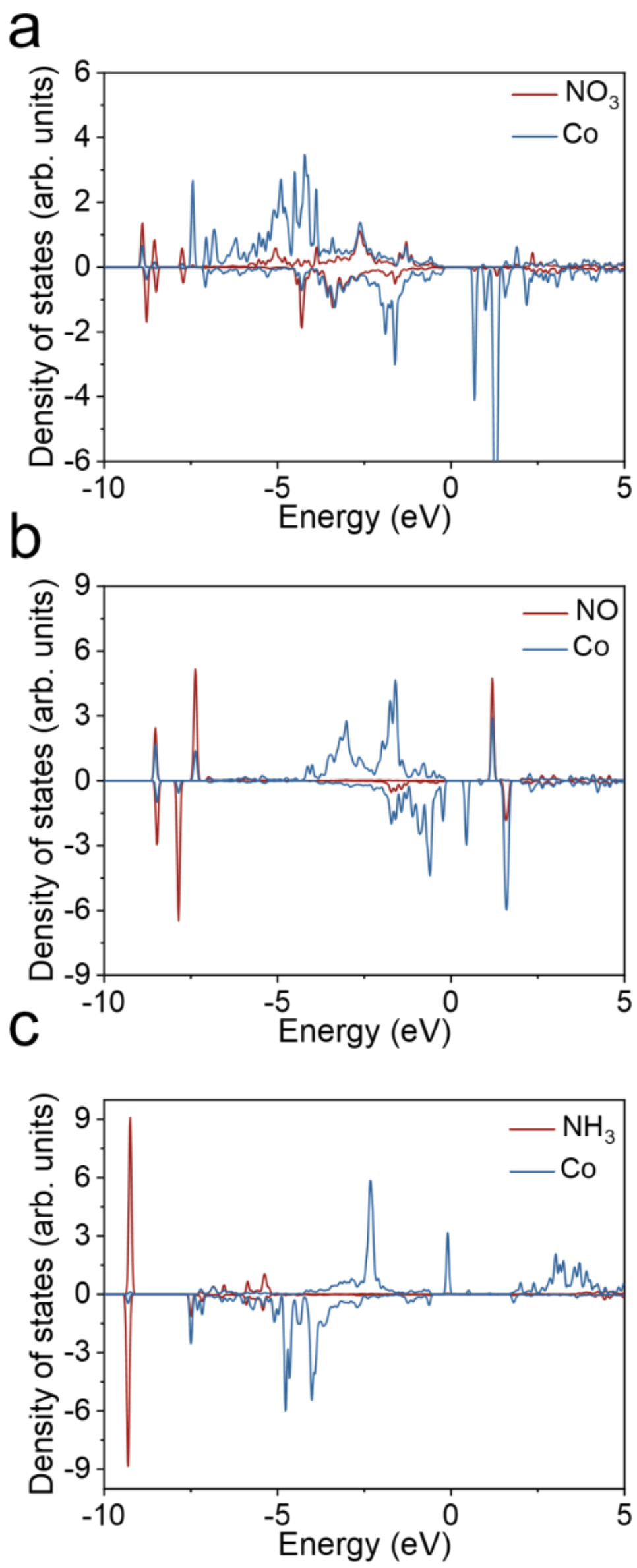

Supplementary Figure 66. Projected density of states (PDOS) analysis. PDOS of Co 3*d* orbital and (a) *NO_3, (b) *NO, (c) *NH_3 on Co/Cu_2O at the GGA+U level with the Hubbard U = 3.3 eV, respectively. These GGA+U (*U* = 3.3 eV) PDOS plots reveal strong, well-matched orbital hybridization between Co 3*d* orbitals and *NO_3, *NO, and *NH_3 on Co/Cu_2O. Significant overlap near the Fermi level, particularly with the rate-limiting *NO intermediate, facilitates efficient electron transfer and tunes binding strength to an optimal range. This avoids both weak nitrate activation on pristine Cu_2O and severe intermediate poisoning on pure CoO, directly boosting NO_3RR electrocatalytic performance. Source data are provided as a Source Data file.

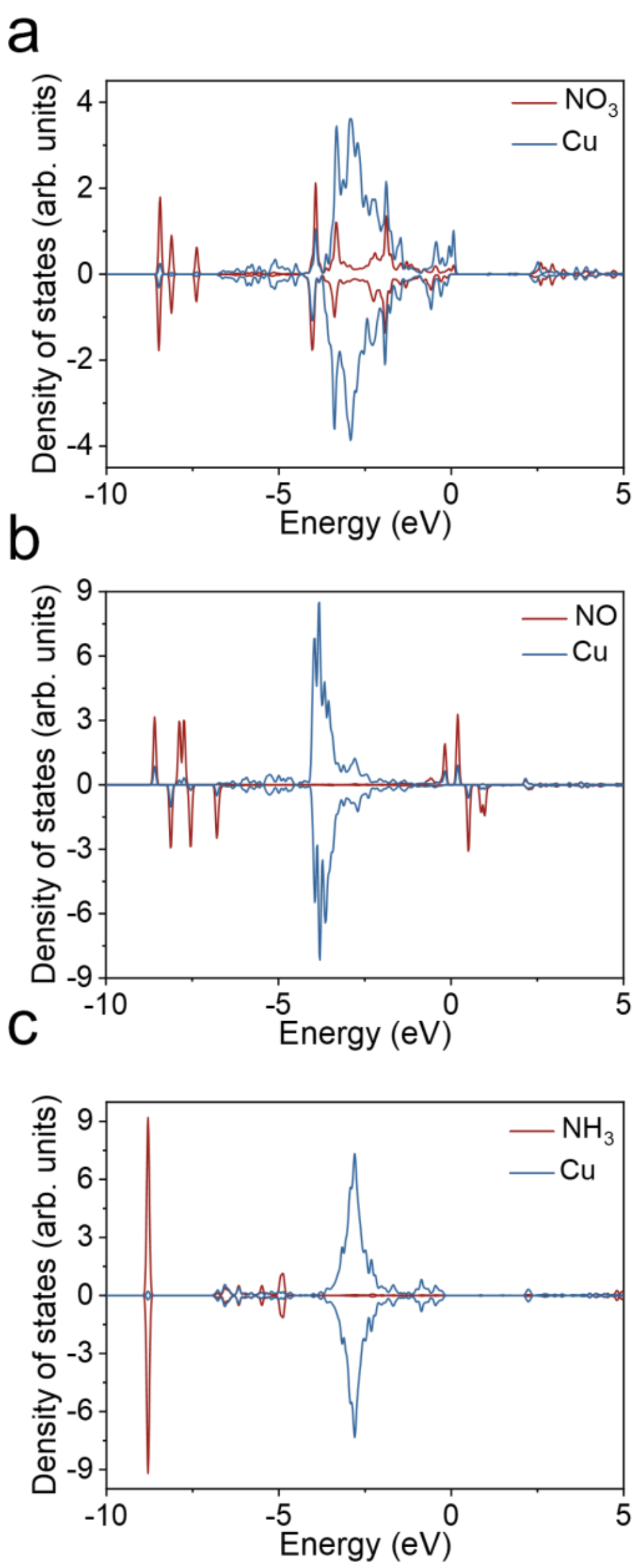

Supplementary Figure 67. Projected density of states (PDOS) analysis. PDOS of Cu 3*d* orbital and (a) $*NO_3$, (b) *NO, (c) $*NH_3$ on Cu_2O at the GGA+U level with the Hubbard U = 5.0 eV, respectively. These GGA+U (U = 5.0 eV) PDOS plots reveal severely mismatched orbital hybridization between Cu 3*d* orbitals and $*NO_3$, *NO, $*NH_3$ on pristine Cu_2O. Unlike Co/Cu_2O with well-balanced Fermi-level overlap, bare Cu sites show weak $*NO_3$ activation and excessively strong *NO trapping. This unbalanced coupling impairs electron transfer, elevates the rate-limiting deoxygenation barrier, and directly accounts for the inferior NO_3RR electrocatalytic performance relative to Co-doped Cu_2O. Source data are provided as a Source Data file.

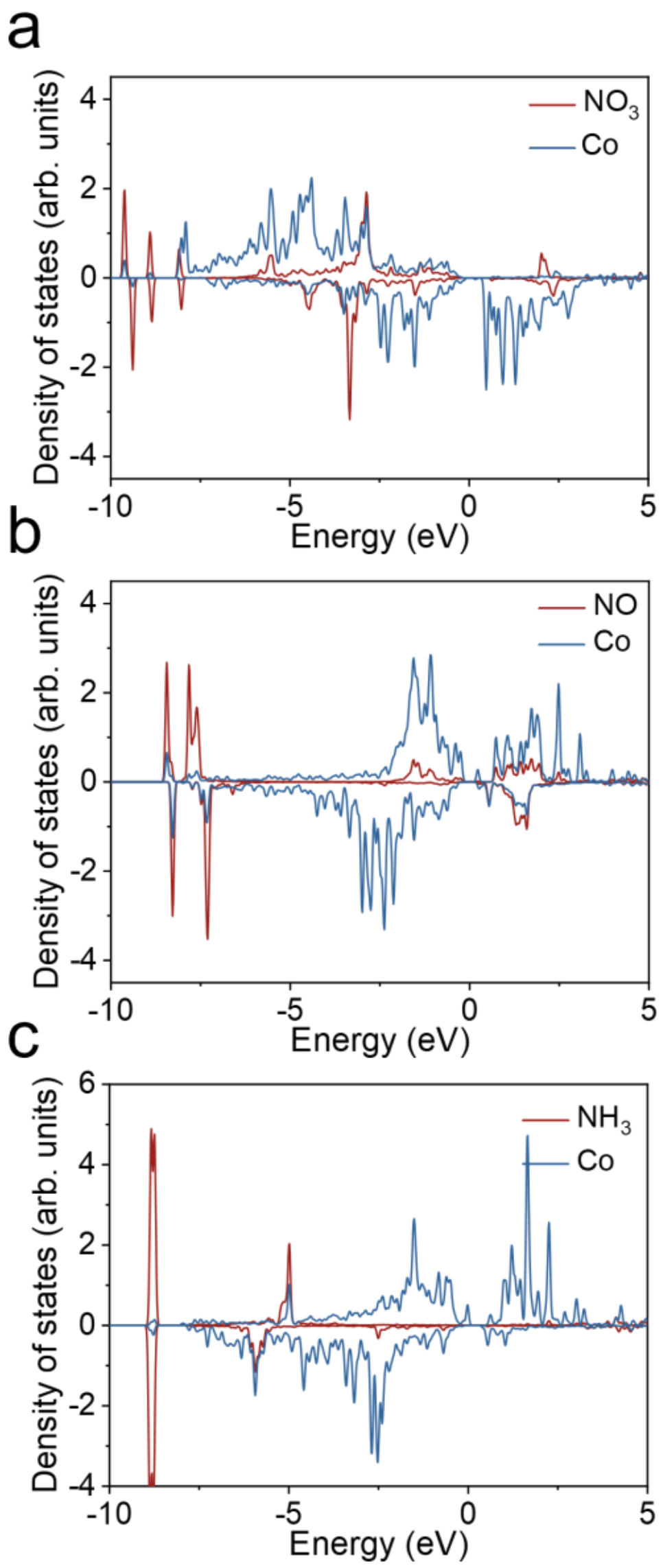

Supplementary Figure 68. Projected density of states (PDOS) analysis. PDOS of Co 3*d* orbital and (a) $*NO_3$, (b) *NO, (c) $*NH_3$ on CoO at the GGA+U level with the Hubbard U = 3.3 eV, respectively. These GGA+U (U = 3.3 eV) PDOS plots reveal excessive orbital hybridization between Co 3*d* orbitals and $*NO_3$, *NO, $*NH_3$ on pure CoO. Unlike Co/Cu_2O with well-balanced Fermi-level overlap, pure Co sites exhibit overly strong binding to all intermediates, especially the rate-limiting *NO. This overbinding traps reactive species, blocks active sites, and severely impedes subsequent protonation steps, resulting in drastically inferior nitrate-to-ammonia electrocatalytic performance relative to Co/Cu_2O. Source data are provided as a Source Data file.

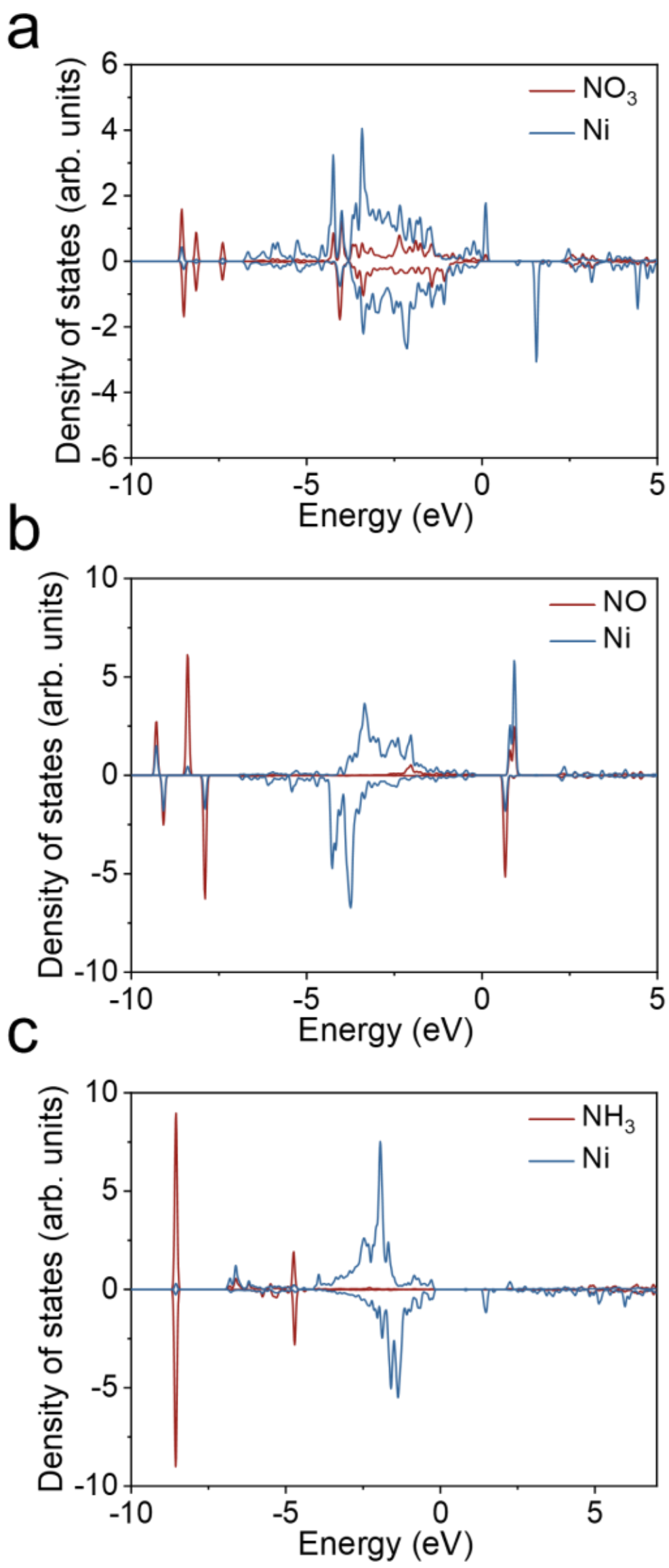

Supplementary Figure 69. Projected density of states (PDOS) analysis. PDOS of Ni 3*d* orbital and (a) $*NO_3$, (b) *NO, (c) $*NH_3$ on Ni/Cu_2O at the GGA+U level with the Hubbard U = 6.0 eV correction, respectively. These GGA+U (U = 6.0 eV) PDOS plots show well-matched orbital hybridization between Ni 3*d* orbitals and $*NO_3$, *NO, $*NH_3$ on Ni/Cu_2O. Compared to Co/Cu_2O, Ni sites exhibit slightly stronger Fermi-level overlap with the rate-limiting *NO intermediate, leading to marginally higher binding energy and deoxygenation barrier. Nevertheless, both doped catalysts outperform pristine Cu_2O and pure CoO, with Co/Cu_2O remaining the optimal system for nitrate-to-ammonia conversion. Source data are provided as a Source Data file.

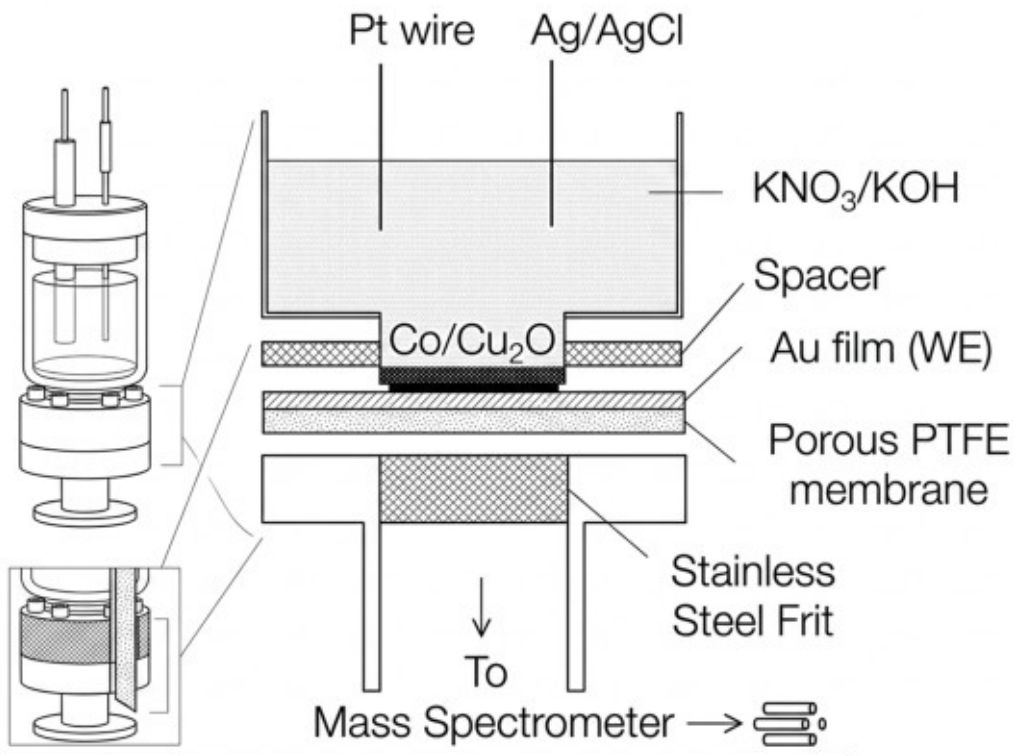

Supplementary Figure 69. *In-situ* differential electrochemical mass spectrometry schematic diagram.

Supplementary Table 1. The spin moment of sample.

Sample	Spin moment (μ_B)
Cu_2O	0
Cr/Cu_2O	2.961
Mn/Cu_2O	3.973
Fe/Cu_2O	3.973
Co/Cu_2O	1.150
Ni/Cu_2O	0.746
CoO	2.077

Supplementary Table 2. Inductively coupled plasma optical emission spectrometry (ICP-OES) of Metal/Cu_2O.

Sample	Metal (wt%)
Cu_2O	0
Cr/Cu_2O	0.2
Mn/Cu_2O	0.2
Fe/Cu_2O	0.4
Co/Cu_2O	0.3
Ni/Cu_2O	0.2

Supplementary Table 3. EXAFS fitting parameters at the Co K-edge for various samples. (S_0^2 = 0.78 from Co-foil)

	shell	CN^a	R^b(Å)	σ^{2c}(Å^2)	ΔE_0^{d}(eV)	R factor
Co-foil	Co-Co	12	2.49±0.01	0.0063	7.3±0.3	0.0014
CoO	Co-O	5.9±0.4	2.12±0.01	0.0125	-3.8±0.3	0.0065
	Co-Co	12.2±0.4	3.00±0.01	0.0088		
Co_2O_3	Co-O	5.4±0.2	1.92±0.01	0.0035	-5.1±0.2	0.0050
	Co-Co	6.0±0.2	2.87±0.01	0.0060		
	Co-Co	6.2±0.3	3.37±0.01	0.0050		
Co/Cu_2O	Co-O	4.4±0.3	2.07±0.01	0.0102	1.0±0.7	0.0129
	Co-Co/Cu	4.3±0.4	2.52±0.01	0.0121		

^{a}CN: coordination numbers; ^{b}R: bond distance; $^c\sigma^2$: Debye-Waller factors; $^d\Delta E_0$: the inner potential correction. R factor: goodness of fit. A reasonable range of EXAFS fitting parameters: $0.70 < S_0^2 < 1.00$; CN > 0; $\sigma^2 > 0$ Å^2; $|\Delta E_0| < 15$ eV; R factor < 0.02.

Supplementary Table 4. EXAFS fitting parameters at the Cr K-edge for various samples. (S_0^2 = 0.78 from Cr-foil)

	shell	CN[a]	R[b](Å)	σ^{2c}(Å^2)	$\Delta E_0{}^d$(eV)	R factor
Cr-foil	Cr-Cr	8	2.49±0.01	0.0067	2.7±0.9	0.0058
	Cr-Cr	6	2.86±0.01	0.0045		
Cr_2O_3	Cr-O	6.0±0.3	1.99±0.01	0.0025	-7.4±0.8	0.0101
	Cr-Cr	1.0±0.2	2.61±0.01	0.0026		
	Cr-Cr	4.0±0.3	2.89±0.01	0.0049		
Cr/Cu_2O	Cr-O	4.7±0.3	1.97±0.01	0.0030	-3.9±0.7	0.0159
	Cr-Cu	1.3±0.5	2.94±0.01	0.0082		

[a]CN: coordination numbers; [b]R: bond distance; [c]σ^2: Debye-Waller factors; [d]ΔE_0: the inner potential correction. R factor: goodness of fit. A reasonable range of EXAFS fitting parameters: 0.70 < S_0^2 < 1.00; CN > 0; σ^2 > 0 Å^2; $|\Delta E_0|$ < 15 eV; R factor < 0.02.

Supplementary Table 5. EXAFS fitting parameters at the Mn K-edge for various samples. (S_0^2 = 0.71 from Mn-foil)

	shell	CN[a]	R[b](Å)	σ^{2c}($Å^2$)	ΔE_0[d](eV)	R factor
Mn-foil	Mn-Mn	2	2.58±0.01	0.0071	8.8±1.7	0.0184
MnO	Mn-O	6.1±0.3	2.21±0.01	0.0093	-5.0±0.5	0.0043
	Mn-Mn	12.2±0.4	3.13±0.01	0.0094		
Mn/Cu_2O	Mn-O	4.8±0.3	2.06±0.02	0.0032	-4.6±0.9	0.0188
	Mn-Mn	3.9±0.9	3.11±0.02	0.0140		

[a]CN: coordination numbers; [b]R: bond distance; [c]σ^2: Debye-Waller factors; [d]ΔE_0: the inner potential correction. R factor: goodness of fit. A reasonable range of EXAFS fitting parameters: $0.70 < S_0^2 < 1.00$; CN > 0; $\sigma^2 > 0$ $Å^2$; $|\Delta E_0| < 15$ eV; R factor < 0.02.

Supplementary Table 6. EXAFS fitting parameters at the Fe K-edge for various samples. (S_0^2 = 0.77 from Fe-foil)

	shell	CN[a]	R[b](Å)	σ^{2c}(Å^2)	ΔE_0^d(eV)	R factor
Fe-foil	Fe-Fe1	8	2.46±0.01	0.0050	5.7±1.0	0.0062
	Fe-Fe2	6	2.84±0.01	0.0060		
FeO	Fe-O	6.0±0.4	2.12±0.01	0.0144	3.4±0.4	0.0101
	Fe-Fe	12.2±0.5	3.07±0.02	0.0120		
Fe/Cu_2O	Fe-O	4.5±0.4	2.08±0.02	0.0032	5.1±1.4	0.0192
	Fe-Cu	1.7±0.4	2.94±0.02	0.0123		

[a]CN: coordination numbers; [b]R: bond distance; [c]σ^2: Debye-Waller factors; [d]ΔE_0: the inner potential correction. R factor: goodness of fit. A reasonable range of EXAFS fitting parameters: $0.70 < S_0^2 < 1.00$; CN > 0; $\sigma^2 > 0$ Å^2; $|\Delta E_0| < 15$ eV; R factor < 0.02.

Supplementary Table 7. EXAFS fitting parameters at the Ni K-edge for various samples. ($S_0^2 = 0.79$ from Ni-foil)

	shell	CN[a]	R[b](Å)	σ^{2c}(Å^2)	$\Delta E_0{}^d$(eV)	R factor
Ni-foil	Ni-Ni	12	2.48±0.01	0.0061	7.4±0.4	0.0018
NiO	Ni-O	5.9±0.4	2.09±0.01	0.0060	-4.0±0.3	0.0060
	Ni-Ni	12.0±0.4	2.95±0.01	0.0063		
Ni/Cu_2O	Ni-O	5.0±0.3	2.04±0.01	0.0029	-2.7±0.6	0.0193
	Ni-Cu	0.4±0.2	3.03±0.01	0.0032		

[a]CN: coordination numbers; [b]R: bond distance; [c]σ^2: Debye-Waller factors; [d]ΔE_0: the inner potential correction. R factor: goodness of fit. A reasonable range of EXAFS fitting parameters: $0.70 < S_0^2 < 1.00$; CN > 0; $\sigma^2 > 0$ Å^2; $|\Delta E_0| < 15$ eV; R factor < 0.02.

Supplementary Table 8. Magnetic parameters of the hybrids at 25°C.

Sample	M_s (emu g^{-1})	M_r (emu g^{-1})	M_r/M_s	Hc (Oe)
Cu_2O	0.053	none	none	none
Co/Cu_2O	0.054	0.0062	0.11	40.45
CoO	0.42	0.015	0.036	41.11

Supplementary Table 9. The corresponding potential of Metal/Cu_2O and CoO at j = 10 mA cm^{-2}.

Sample	$E_{j=10}$ (V *vs.* RHE)
CoO	none
Cr/Cu_2O	-0.093
Mn/Cu_2O	-0.176
Fe/Cu_2O	-0.332
Co/Cu_2O	0.008
Ni/Cu_2O	-0.158
Cu_2O	-0.363

Supplementary Table 10. A table of the double-layer capacitance derived ECSA.

Sample	mF cm^{-1}	ECSA (cm^{-1})
Cr/Cu_2O	0.97	1.21
Mn/Cu_2O	0.92	1.15
Fe/Cu_2O	0.89	1.11
Co/Cu_2O	0.83	1.03
Ni/Cu_2O	0.94	1.17
Cu_2O	0.87	1.05
CoO	0.81	1

Supplementary Table 11. Comparison of catalytic parameters of different NO_3RR electrocatalysts.

Catalyst	Electrolyte	NH_3 FE(%)	NH_3 yield rate	Ref.
Co/Cu_2O	0.1 M KNO_3 + 0.1 M KOH	96.5	2.8 mmol h^{-1} mg_{cat}^{-1}	This work
Cu/Cu_2O	200 ppm NO_3^--N + 0.5 M Na_2SO_4	95.8	0.2449 mmol h^{-1} cm^{-2}	Ref[1]
Cu–PTCDA	500 ppm NO_3^- + 0.5 M PBS	85.9	436 μg h^{-1} cm^{-2}	Ref[2]
Ru/O-doped-Ru	0.1 M KNO_3 + 0.1 M KOH	96	1.17 mmol h^{-1} cm^{-2}	Ref[3]
$Cu_{50}Ni_{50}$	100 mM KNO_3 + 1 M KOH	99	-	Ref[4]
Fe SAC	0.5 M KNO_3 + 0.1 M K_2SO_4	75	0.46 mmol h^{-1} cm^{-2}	Ref[5]
CuPd nanocubes	1 M KNO_3 + 1 M KOH	92.5	6.25 mol h^{-1} g^{-1}	Ref[6]
RuO_2 nanosheets	200 ppm NO_3^-—N + 0.5 M Na_2SO_4	97.46	0.1158 mmol h^{-1} cm^{-2}	Ref[7]
Ru_1Cu_{10}/rGO	1 M KNO_3 + 1 M KOH	98	0.38 mmol h^{-1} cm^{-2}	Ref[8]
$Ru_{15}Co_{85}$	0.1 M KNO_3 + 0.1 M KOH	97	3.2 mol g^{-1} h^{-1}	Ref[9]
MOF-818(Cu)−Co	0.1 M KNO_3 + 0.5 M K_2SO_4	99.5	1.06 mol g_{cat}^{-1} h^{-1}	Ref[10]
Cu-NC-800	0.5 M NO_3^- + 0.5 M H_2SO_4	98.6	11.8 mg h^{-1} cm^{-2}	Ref[11]

References

1. Wang Y, *et al.* Unveiling the activity origin of a copper-based electrocatalyst for selective nitrate reduction to ammonia. *Angew. Chem. Int. Ed.* **59**, 5350-5354 (2020).
2. Chen GF, *et al.* Electrochemical reduction of nitrate to ammonia via direct eight-electron transfer using a copper-molecular solid catalyst. *Nat. Energy* **5**, 605-613 (2020).
3. Li J, *et al.* Efficient ammonia electrosynthesis from nitrate on strained ruthenium nanoclusters. *J. Am. Chem. Soc.* **142**, 7036-7046 (2020).
4. Wang YH, *et al.* Enhanced nitrate-to-ammonia activity on copper-nickel alloys via tuning of intermediate adsorption. *J. Am. Chem. Soc.* **142**, 5702-5708 (2020).
5. Wu Z-Y, *et al.* Electrochemical ammonia synthesis via nitrate reduction on Fe single atom catalyst. *Nat. Commun.* **12**, 2870 (2021).
6. Gao Q, *et al.* Breaking adsorption-energy scaling limitations of electrocatalytic nitrate reduction on intermetallic CuPd nanocubes by machine-learned insights. *Nat. Commun.* **13**, 2338 (2022).
7. Wang Y, *et al.* Structurally disordered RuO_2 nanosheets with rich oxygen vacancies for enhanced nitrate electroreduction to ammonia. *Angew. Chem. Int. Ed.* **61**, e202202604 (2022).
8. Gao WS, *et al.* Alloying of Cu with Ru enabling the relay catalysis for reduction of nitrate to ammonia. *Adv. Mater.* **35**, 2202952 (2023).
9. Han S, *et al.* Ultralow overpotential nitrate reduction to ammonia via a three-step relay mechanism. *Nat. Catal.* **6**, 402-414 (2023).
10. Sun H, *et al.* Tandem Cu–Co Sites in MOF-818 for efficient ammonia electrosynthesis from nitrate in neutral media. *ACS Catal.* **15**, 16581-16590 (2025).
11. Zhang P, *et al.* Regulating Cu atom dispersity on nitrogen-doped carbon for boosting electrocatalytic nitrate reduction in strongly acidic media. *Small* **22**, e13666 (2026).
