# Supplementary material for: Spin state and orbital engineering of cuprous oxide for efficient nitrate reduction
Source: Nat Commun. 2026 Aug 15;17:9827. doi: 10.1038/s41467-026-76420-8 (PMC13575217; doi:10.1038/s41467-026-76420-8)
Supplement: Supplementary file 2 [file 41467_2026_76420_MOESM2_ESM.pdf]

Description of Additional Supplementary Files

File name: Supplementary Data 1

Description:

This file contains the optimized atomic coordinates of all DFT models used in this study. All structures were fully relaxed at the GGA (PBE) level with the computational parameters described in the Methods section. The file includes the following models:

Co/Cu_2O model: optimized structures of $*NH_3$ adsorption and *H adsorption on the Co/Cu_2O surface.

Cu_2O model: optimized structures of $*NH_3$ adsorption and *H adsorption on the pristine Cu_2O surface.

Cr/Cu_2O model: optimized structure of $*NH_3$ adsorption on the Cr/Cu_2O surface.

Mn/Cu_2O model: optimized structure of $*NH_3$ adsorption on the Mn/Cu_2O surface.

Fe/Cu_2O model: optimized structure of $*NH_3$ adsorption on the Fe/Cu_2O surface.

Ni/Cu_2O model: optimized structure of $*NH_3$ adsorption on the Ni/Cu_2O surface.

CoO model: optimized structure of *H adsorption on the CoO surface.
