# Supplementary material for: Spin state and orbital engineering of cuprous oxide for efficient nitrate reduction
Source: Nat Commun. 2026 Aug 15;17:9827. doi: 10.1038/s41467-026-76420-8 (PMC13575217; doi:10.1038/s41467-026-76420-8)
Supplement: Supplementary file 3 [file 41467_2026_76420_MOESM4_ESM.pdf]

nature portfolio

Peer Review File

Spin State and Orbital Engineering of Cuprous Oxide for Efficient Nitrate Reduction

Corresponding Author: Professor Jia-Nan Zhang

This file contains all reviewer reports in order by version, followed by all author rebuttals in order by version.

Version 0:

Reviewer comments:

Reviewer #1

(Remarks to the Author)
This manuscript presents a series of magnetic transition metal-doped Cu2O catalysts (M-Cu2O), demonstrating atomic-level tuning of the material. The work shows that CoIII can optimize d-p orbital hybridization, the availability of adsorption sites, and the adsorption-desorption dynamics of key intermediates, leading to efficient redox kinetics. However, significant concerns regarding catalyst synthesis and characterizations, data interpretation, and mechanistic depth must be rigorously addressed to establish the scientific rigor required for publication. My specific comments are below:

1. For the catalyst synthesis, generally NaBH4 is used as a reductant, and the Cu2+ was reduced to Cu+; why was Co2+ oxidized to Co3+? Because without the introduction of Cu2+, the product was Co(+2)O. Is it possible not really 3+ merely due to oxygen defects?
2. Some synthesized samples seem not uniform, as shown in the XRD patterns in Figures S10-S13 (compared to Figure S6a, copious CuO exists) and the EDX-mapping figures in Figure S11 (Mn-rich zone exists). In Figure 1d, it seems that some unknown peaks belong to the CoO impurity.
3. It is challenging to directly discern the Co atom from the Cu atom in the AC-STEM image due to the close Z number.
4. The peak at 2.2 Å is very close to that of metallic Co in Figure 2b, making the assignment to the Co–M second coordination shell interaction questionable. Besides, it is suggested to discuss why the Co–M signal is significantly different from that under the OCP condition in Figure 4i. The XAS data for standard CuO are also suggested to be provided in Figure 2d,e.
5. Error bars need to be added to the catalytic performance based on repetitive three-times measurements.
6. The authors investigated the intermediates using the in-situ FTIR technique and obtained the main signal of NH2*. To further underpin the reaction pathway in Figure 5a, in situ DEMS experiments should be performed to provide more detailed information on intermediates.
7. For the in-situ Raman test, given that the carbon nanotube was introduced during the catalyst synthesis. It is challenging to discern the peaks of adsorbed NO3- at 1352 cm−1 because of the D peak of carbon materials. Besides, the signal between 200 and 800 also needs to be shown to demonstrate the structural stability of Cu2O.
8. To concisely analyze the valence of Cu, it is suggested to provide the LMM Auger spectra in the related XPS discussions.
9. The temperature range used for the Arrhenius analysis is relatively narrow, which may limit the reliability of the extracted activation energies. Besides, it is suggested to provide a photo of the setup with temperature control for the experiments.
10. Due to the selected different adsorption modes for the *NO3 on different systems (such as Top coordination: M-N or M-O, Bridge coordination), it is suggested to show the calculated details to demonstrate the reason for the selection of different modes.
11. It is necessary to conduct theoretical calculations at the GGA+U level with the Hubbard U correction, especially for the transition metal oxide systems.
12. To demonstrate the rationality of built structural modes in DFT calculations, such as Figures S4 and S5, the tables about the structural parameters extracted from the EXAFS fitting need to be supplemented for all samples.

Reviewer #2

(Remarks to the Author)
This manuscript by Xia et al. presents a systematic study on transition-metal-doped Cu2O electrocatalysts for the nitrate

reduction reaction (NO3RR), with a particular focus on Co-substituted Cu2O. The authors combine DFT calculations, advanced spectroscopic characterizations (XAFS, EELS, EPR), and electrochemical performance evaluations to demonstrate that spin-altered CoIII sites optimize d-p orbital hybridization, lower the reaction barrier of the *NO → *NOH step, and achieve excellent NH3 yield and Faradaic efficiency. The work is timely, methodologically sound, and provides valuable mechanistic insights into orbital-scale electrocatalyst design. The manuscript is well-organized and clearly written. I recommend minor revision after the following points are addressed.

1. The authors state that strong proton binding on Co/Cu2O suppresses the hydrogen evolution reaction (HER). However, strong H* binding typically lowers the HER barrier. Please add a clarifying sentence to resolve this apparent contradiction. For example: “Although Co/Cu2O binds H* more strongly than Cu2O, the preferential occupation of active sites by NO3- intermediates kinetically suppresses HER.” Alternatively, a brief discussion on whether kinetic barriers or site-blocking effects dominate would be helpful.
2. In Fig. 6f, the authors state that CuI (3d10) yields a π-bond order of 2.5, which is higher than that of CoIII (~1), yet CuI is claimed to exhibit weaker *NO adsorption. Please explain this apparent discrepancy in the text. For instance, does bond order alone not determine adsorption strength due to differences in orbital energy alignment or back-donation efficiency? A short mechanistic clarification would suffice.
3. Several supplementary figures (e.g., Supplementary Figs. 1, 2, 3) are mentioned in the main text without a brief description. Please add short parenthetical explanations to guide readers, such as “(Supplementary Fig. 1 shows the computed magnetic moments)”.
4. The assignment of an intermediate-spin (S=1) configuration for CoIII is well supported by EPR and magnetic susceptibility data. However, the in-situ XANES shows a negative shift in the pre-edge energy at cathodic potentials, suggesting a change in the Co oxidation state or coordination environment. Could the spin state evolve under operating conditions (e.g., from S=1 to S=1/2 or S=2)? A brief discussion or reference to related literature on potential-induced spin-state crossover would be valuable, and can be added without new experiments.

Reviewer #3

(Remarks to the Author)

This is a well-written manuscript presenting a DFT-inspired strategy to modify Cu_2O via low-level Co substitution for nitrate reduction. The combination of theory and experiment is appropriate, and the catalytic performance is encouraging. However, several of the central conclusions are not sufficiently supported.

Major Points

XANES interpretation appears overstated
The interpretation of the Co K-edge XANES spectra appears somewhat overstated. The manuscript states:
“The Co K-edge XANES spectra show that Co/Cu_2O exhibits near-edge features comparable to Co_2O_3 (Figure 2a) albeit with a slightly attenuated white-line intensity. This systematic shift indicates the existence of lattice-confinement effects, which perturb the Co electronic structure and maintain a predominant CoIII oxidation state.”
While the near-edge features show some similarity to Co_2O_3, this alone is not sufficient to unambiguously assign a predominant Co^{3+} oxidation state, particularly in a structurally complex and low-loading system. Changes in white-line intensity can arise from variations in coordination environment, covalency, and site symmetry, and are not uniquely indicative of “lattice-confinement effects.” The authors are encouraged to moderate the language to reflect a more qualitative interpretation. Focus on the edge position and how you relate that back to valence state.

XAFS analysis is qualitative and does not support structural claims
The Co K-edge XAFS data are central to the interpretation yet remain largely qualitative. No quantitative XAFS fitting (coordination numbers, bond lengths, uncertainties) is presented.
Given the strength of the structural and electronic claims, a more rigorous analysis is required, or the conclusions should be moderated. For example, Fig. 2b alone does not necessarily rule out clustering. It is also not clear how the second-shell contribution is assigned specifically to Co–Cu rather than Co–Co.
The wavelet transform analysis (Fig. 2c) is difficult to interpret and appears notably different from both Co_2O_3 (Fig. S19d) and single-atom references such as Co–PC (Fig. S19c), which warrants further clarification.

Inconsistency in metal–metal coordination discussion
The statement that control experiments show “other transition-metal-doped Cu2O samples further support this structural model (Figures S21-S24), revealing exclusive metal-oxygen coordination without detectable metal-metal bonds” appears inconsistent with other parts of the manuscript. For example, in the Co-doped sample, metal–metal contributions are discussed and attributed to Co–Cu coordination. This point should be clarified to avoid confusion.

Atomic dispersion of Co is not convincingly demonstrated
The claim of atomically dispersed Co is not fully supported. The absence of XRD features does not exclude small clusters or amorphous phases, particularly at low loading. As such, the conclusion relies heavily on XAFS, which is currently interpreted qualitatively.
I encourage the authors to perform quantitative XAFS analysis to support this claim, or to moderate the wording. The limitations of XRD for detecting low-loading or amorphous species should also be acknowledged.

Minor Points

Fig. 2a, b: The insets use both symbols and colours; for clarity, please include the symbols in the legend as well.
The SI should include the raw XAFS spectra (k-space data), which are currently not shown.

Version 1:

Reviewer comments:

Reviewer #1

(Remarks to the Author)
I think the authors have carefully addressed most of my concerns, except for the following issues. I recommend the revised manuscript for publication in Nature Communications after revision.

1. Compared to Figure S6a (pure Cu_2O), the authors exclude the possibility of CoO impurity in Figure 1d; I still encourage the authors to check the origin of the unknown peak adjacent to the (200) plane of Cu_2O. One reason is that the theoretical angle between (220) and (111) is about 35.3 degree based on the information of the PDF card (#01-1142), but the real angle is close to 90 degree, as shown in Figure 1c.

2. Based on the standard reference spectra (https://www.thermofisher.cn/cn/en/home/materials-science/learning-center/periodic-table/transition-metal/copper.html), it is suggested to recheck the analysis of Cu valence further about the obtained Cu-LMM spectra in Figure S15.

Reviewer #2

(Remarks to the Author)
The reviewers' comments have been well addressed. I recommend this article be published in Nature Communications.

Reviewer #3

(Remarks to the Author)
Thank you very much for addressing the points raised. I believe this work is now ready for publication.

Version 2:

Reviewer comments:

Reviewer #1

(Remarks to the Author)
All my concerns have been addressed in the revised manuscript. I would recommend its publication in the current form.

Open Access This Peer Review File is licensed under a Creative Commons Attribution 4.0 International License, which permits use, sharing, adaptation, distribution and reproduction in any medium or format, as long as you give appropriate credit to the original author(s) and the source, provide a link to the Creative Commons license, and indicate if changes were made.
In cases where reviewers are anonymous, credit should be given to 'Anonymous Referee' and the source.
The images or other third party material in this Peer Review File are included in the article's Creative Commons license, unless indicated otherwise in a credit line to the material. If material is not included in the article's Creative Commons license and your intended use is not permitted by statutory regulation or exceeds the permitted use, you will need to obtain permission directly from the copyright holder.
To view a copy of this license, visit https://creativecommons.org/licenses/by/4.0/

Response Letter to Reviewers

Reviewer: 1

General Comments R1: *This manuscript presents a series of magnetic transition metal-doped Cu_2O catalysts (M-Cu_2O), demonstrating atomic-level tuning of the material. The work shows that CoIII can optimize d-p orbital hybridization, the availability of adsorption sites, and the adsorption-desorption dynamics of key intermediates, leading to efficient redox kinetics. However, significant concerns regarding catalyst synthesis and characterizations, data interpretation, and mechanistic depth must be rigorously addressed to establish the scientific rigor required for publication. My specific comments are below:*

Response: We sincerely appreciate the reviewer's thorough and constructive evaluation of our manuscript. These insightful comments have helped us significantly improve the scientific rigor and clarity of our work. We have carefully addressed each point below, and the corresponding revisions have been highlighted in yellow in the revised manuscript and Supporting Information.

Specific Comment R1-1: *For the catalyst synthesis, generally $NaBH_4$ is used as a reductant, and the Cu^{2+} was reduced to Cu^{+}; why was Co^{2+} oxidized to Co^{3+}? Because without the introduction of Cu^{2+}, the product was Co(+2)O. Is it possible not really 3+ merely due to oxygen defects?*

Response: We thank the reviewer for this important mechanistic question. In our synthesis, $NaBH_4$ reduces Cu^{2+} to Cu^{+}, while Co^{2+} is not reduced to Co^{0}; instead, it is stabilized as Co^{3+} within the Cu_2O lattice. This surprising behavior can be rationalized as follows:

1) Lattice confinement effect: The Cu_2O matrix provides a specific coordination environment that favors the oxidation of Co^{2+} to Co^{3+}. The Co^{3+} ion (0.545 Å) has a similar ionic radius to Cu^{+} (0.77 Å) and can substitute into the Cu_2O lattice more readily than Co^{2+} (0.745 Å). This substitution is thermodynamically driven by the minimization of lattice strain and the formation of strong Co-O bonds.
2) Oxidizing environment: Although $NaBH_4$ is a strong reductant, the reaction is carried out in aqueous solution under ambient conditions, where dissolved oxygen can partially oxidize

Co^{2+} to Co^{3+}, especially when Co is atomically dispersed within the Cu_2O matrix. In the absence of Cu^{2+}, Co^{2+} precipitates as $Co(OH)_2$ and is reduced to CoO upon drying, which remains in the Co^{2+} state. The presence of Cu^{2+} alters the redox potential and coordination environment, promoting Co^{3+} stabilization.

3) Oxygen defects contribution: As the reviewer suggests, oxygen vacancies may also contribute to the apparent high oxidation state. However, our XANES and soft XAS data (Fig. 2a, 2f) show a clear shift in the Co *K*-edge and *L*-edge positions consistent with Co^{3+}, not solely due to oxygen vacancies. The multiplet structure of the *L*-edge spectrum matches well with intermediate-spin Co^{3+} ($S = 1$), and the EXAFS fitting excludes metallic Co or CoO nanoparticles. Therefore, while oxygen vacancies may be present, the dominant species is indeed Co^{3+}.

We have added the following clarification in the revised manuscript:

"The stabilization of Co^{III} within the Cu_2O lattice, despite the use of $NaBH_4$, is attributed to lattice confinement and the oxidizing ambient environment. Co^{II} is readily oxidized to Co^{III} during the synthesis, and the Cu_2O matrix thermodynamically favors the incorporation of Co^{III} due to ionic radius matching and strong Co-O bonding. In contrast, without Cu^{II}, Co^{II} precipitates as $Co(OH)_2$ and remains as CoO." (Page 6, L131-135)

Specific Comment R1-2: *Some synthesized samples seem not uniform, as shown in the XRD patterns in Figures S10-S13 (compared to Figure S6a, copious CuO exists) and the EDX-mapping figures in Figure S11 (Mn-rich zone exists). In Figure 1d, it seems that some unknown peaks belong to the CoO impurity.*

Response: We thank the reviewer for pointing out these important issues regarding sample uniformity and potential impurities. We have re-examined our data and conducted additional characterization to address these concerns.

1) Regarding CuO impurities in M/Cu_2O samples: We acknowledge that in some metal-doped samples (e.g., Mn/Cu_2O, Fe/Cu_2O), small amounts of CuO may be present due to partial oxidation during synthesis. However, we note that the CuO peaks (e.g., at ~35.5° and ~38.7°) are very weak compared to the dominant Cu_2O peaks.
2) Regarding Mn-rich zones in EDX mapping: We thank the reviewer for noticing this. The Mn/Cu_2O sample indeed shows some local enrichment of Mn, as seen in the EDX map (Fig. S11b). This is likely due to incomplete homogenization during synthesis. However, we emphasize that the vast majority of Mn is uniformly dispersed, and the XAFS analysis (Fig. S22) confirms that Mn is predominantly in an oxide-like coordination environment without Mn-Mn bonds. For clarity, we have added a statement in the revised manuscript: "While some local enrichment of dopants is observed in EDX maps for certain samples (e.g., Mn), XAFS confirms atomic-level dispersion without metal-metal clustering." (Page 6, L157-159) In addition, were characterized the test sample and found that the elements were evenly distributed without obvious aggregation.
3) Regarding CoO impurity in Fig. 1d: The peaks at ~36.5° and ~42.5° in Fig. 1d are actually the (111) and (200) planes of Cu_2O, not CoO. CoO has its strongest peak at ~36.4° (111), which overlaps with Cu_2O (111), making it difficult to distinguish by XRD alone. However, our HAADF-STEM and EXAFS (Fig. 2b, 2c) confirm the absence of CoO nanoparticles. The slight asymmetry of the Cu_2O peaks may be due to lattice strain induced by Co doping. We have added a note in the revised manuscript: "No detectable signals from metallic Co or cobalt oxides were observed, indicating the exclusive incorporation of Co into the Cu_2O lattice." (Page 6, L138)

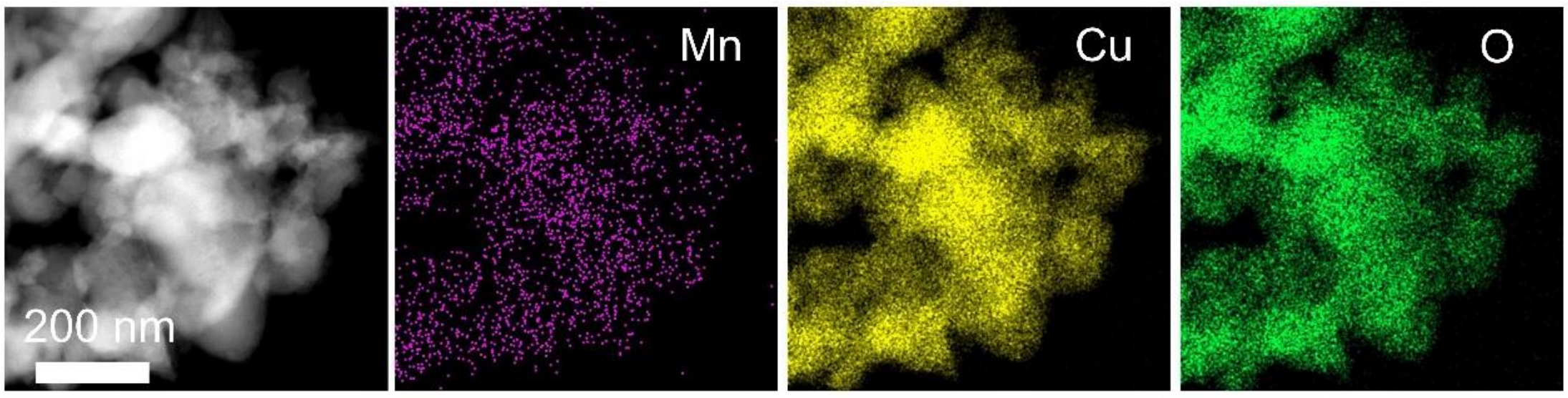

Figure R1 (Supplementary Figure 11b) Elemental mapping images of Mn/Cu_2O.

Specific Comment R1-3: *It is challenging to directly discern the Co atom from the Cu atom in the AC-STEM image due to the close Z number.*

Response: We agree with the reviewer that Co and Cu have similar atomic numbers (Z = 27 *vs*. 29), making direct identification in HAADF-STEM challenging due to similar *Z*-contrast. To address this limitation, we relied on complementary spectroscopic methods:

1) EELS analysis (Fig. 1f): The Co *L*-edge at ~780 eV and Cu *L*-edge at ~934 eV is clearly distinguishable, providing unambiguous chemical identification of Co at the atomic level.
2) AC-STEM with intensity profiling: While individual Co atoms cannot be reliably distinguished from Cu by contrast alone, the 3D surface plot (Fig. 1e) shows slightly lower intensity at positions corresponding to Co atoms, consistent with the lower atomic number of Co. However, we agree that this is not definitive. Therefore, we have revised the text to state more cautiously:

"The aberration-corrected high-angle annular dark-field scanning TEM (AC-HAADF-STEM) (Figure 1c) shows bright atomic-scale features; electron energy loss spectroscopy (EELS) confirms the coexistence of Co and Cu at these locations, indicating the atomic dispersion of Co within the Cu_2O matrix (Figure 1f)." (Page 6, L149-152)

Specific Comment R1-4: *The peak at 2.2 Å is very close to that of metallic Co in Figure 2b, making the assignment to the Co–M second coordination shell interaction questionable. Besides, it is suggested to discuss why the Co–M signal is significantly different from that under the OCP condition in Figure 4i. The XAS data for standard CuO are also suggested to be provided in Figure 2d,e.*

Response: We thank the reviewer for these careful and constructive comments. We have addressed each point as follows.

1. Assignment of the 2.2 Å peak (Fig. 2b) and distinction from metallic Co

The reviewer correctly notes that metallic Co exhibits a Co–Co scattering peak at approximately 2.2 Å in the Fourier-transformed (FT) EXAFS spectrum (uncorrected for phase shift). In our Co/Cu_2O sample, we also observe a peak at ~2.2 Å. However, this peak cannot be assigned to metallic Co clusters for the following reasons:

Phase shift correction: The FT-EXAFS peaks in *R*-space are shifted to lower *R* values relative to the true interatomic distances due to the photoelectron phase shift. After applying a typical phase shift correction ($\Delta R \approx 0.3$–0.5 Å), the true Co–M distance is ~2.5–2.7 Å for Co–Co in metallic Co, whereas for Co–Cu in an oxide lattice it is ~2.8–2.9 Å. Our quantitative EXAFS fitting yields a refined bond distance of 2.85 ± 0.03 Å for the second-shell Co–M path, which is consistent with Co–Cu super exchange via oxygen bridges in Cu_2O, not with metallic Co–Co (~2.49 Å).

Wavelet transform analysis (Fig. 2c): The *k*-dependence of the scattering signal is different. Metallic Co shows a sharp maximum at high *k* (~7–9 $Å^{-1}$) due to backscattering from neighboring Co atoms, whereas Co/Cu_2O shows a broader maximum at lower *k* (~5–7 $Å^{-1}$) characteristic of lighter back scatterers (O and Cu), further ruling out Co clusters.

Absence of Co–Co path in fitting: Attempts to fit the second-shell peak with a Co–Co path resulted in poor fit quality (*R*-factor > 0.02) and unphysically large Debye-Waller factors, whereas fitting with a Co–Cu path yielded excellent agreement (*R*-factor = 0.012). This confirms that the back scatterer is Cu, not Co.

2. Difference in Co–M signal between ex-situ (Fig. 2b) and in-situ under OCP (Fig. 4i)

We thank the reviewer for highlighting this subtle but important difference. In Fig. 4i (*in-

situ EXAFS at OCP), the second-shell Co–M peak appears slightly reduced in intensity and broadened compared to the *ex-situ* spectrum in Fig. 2b. This can be explained by:

Surface *vs*. bulk sensitivity: *In-situ* measurements are performed under electrochemical conditions with a thin electrolyte layer, which may slightly attenuate the EXAFS signal, especially for the second shell. Moreover, the carbon paper electrode contributes background scattering.

Potential-induced electronic relaxation: Even at OCP, the electrode potential is not identical to the ex-situ dry state. The catalyst in contact with electrolyte may undergo slight surface hydroxylation or partial reduction of Co^{3+} to Co^{2+} at the outermost layers, which weakens the Co–O–Cu super exchange coupling and reduces the second-shell scattering amplitude.

Reversibility: Importantly, when the potential is returned to OCP after cathodic polarization, the Co–M peak recovers (Fig. 4i, OCP vs. before), indicating that the change is reversible and not due to permanent damage or aggregation.

3. Addition of CuO standard XAS data in Fig. 2d,e

As suggested, we have now included the Cu *K*-edge XANES and FT-EXAFS spectra of a CuO reference in **Figure R2** (Fig. 2d and 2e). The CuO standard clearly shows a higher edge energy (consistent with Cu^{2+}) and a distinct FT-EXAFS peak pattern (Cu–O at ~1.5 Å, Cu–Cu at ~2.6–2.8 Å). Comparison with Co/Cu_2O and Cu_2O confirms that our samples are dominated by Cu^{+}, with no detectable CuO phase within the EXAFS detection limit.

The revised figures have been updated in the main manuscript, and the corresponding figure captions have been modified accordingly. All changes are highlighted in yellow.

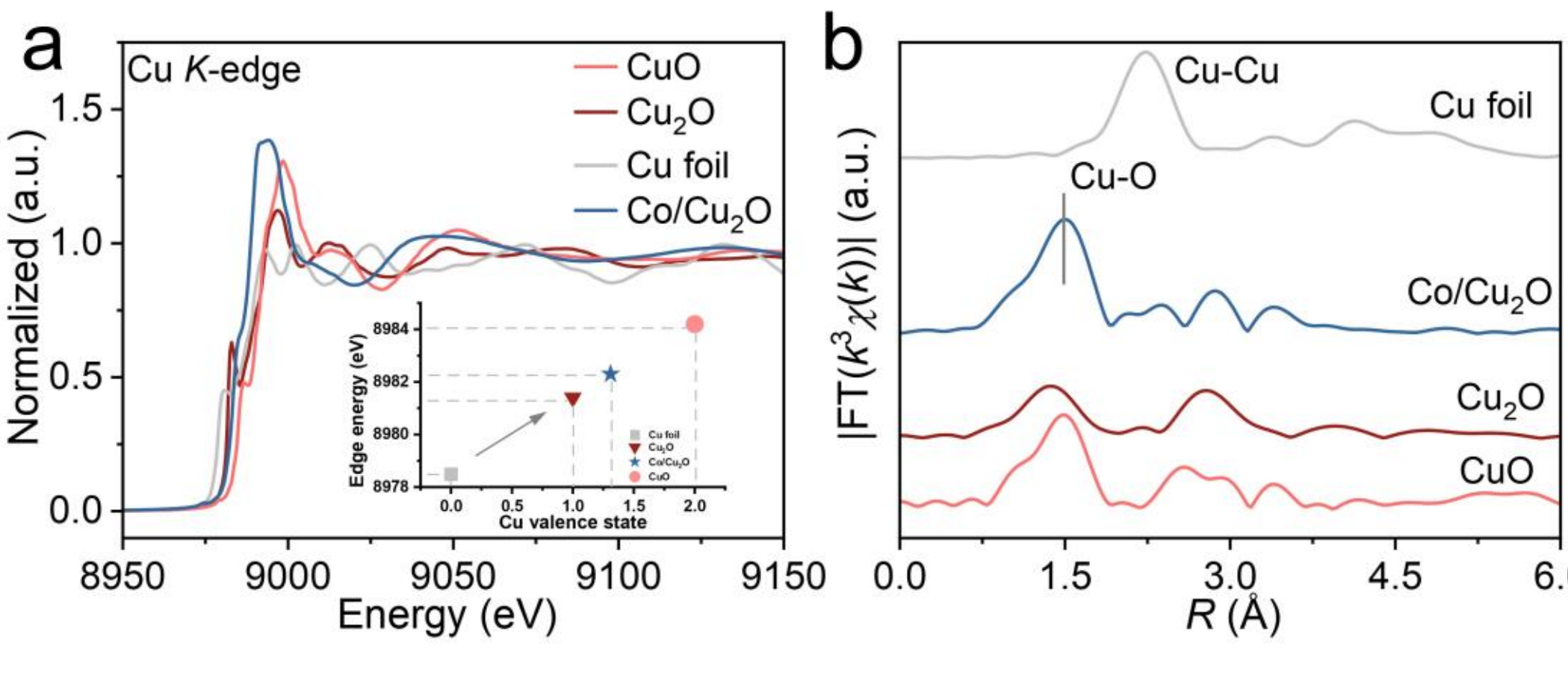

Figure R2 (Figure 2d,e) (a) Cu *K*-edge XANES spectra and (b) FT-EXAFS spectra of various catalysts.

Specific Comment R1-5: *Error bars need to be added to the catalytic performance based on repetitive three-times measurements.*

Response: We thank the reviewer for this important suggestion. We fully agree that error bars are essential for assessing the reproducibility and reliability of catalytic performance data. In the revised manuscript, we have now added error bars (standard deviation from three independent measurements) to all relevant figures, including: Fig. 3b, 3e, and Supplementary Figures 33, 36, 38, 39.

All error bars represent the standard deviation (s.d.) of triplicate measurements. The corresponding figure captions have been updated to indicate that error bars denote s.d. (n = 3). Additionally, we have added a statement in the Methods section to clarify the reproducibility assessment:

"All electrochemical measurements (except long-term stability tests) were repeated at least three times using independently prepared electrodes and electrolyte solutions. The data are presented as mean ± standard deviation." (Supporting information Page 3)

We believe these additions fully address the reviewer's concern and improve the transparency and rigor of our study. All changes are highlighted in yellow in the revised manuscript and Supporting Information.

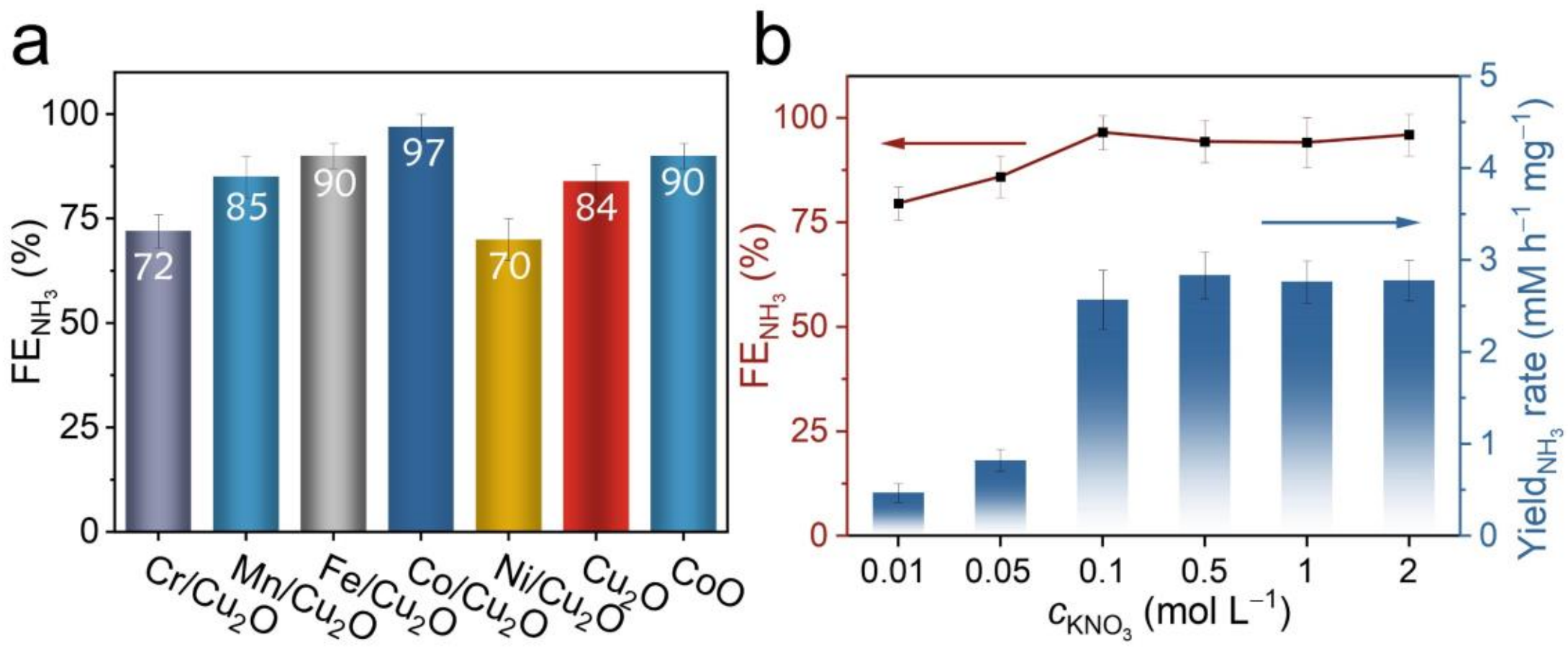

Figure R3 (Figure 3b,e) (a) FE plots for NH_3 synthesis of different samples. (b) NO_3RR performance over Co/Cu_2O at different concentrations of KNO_3.

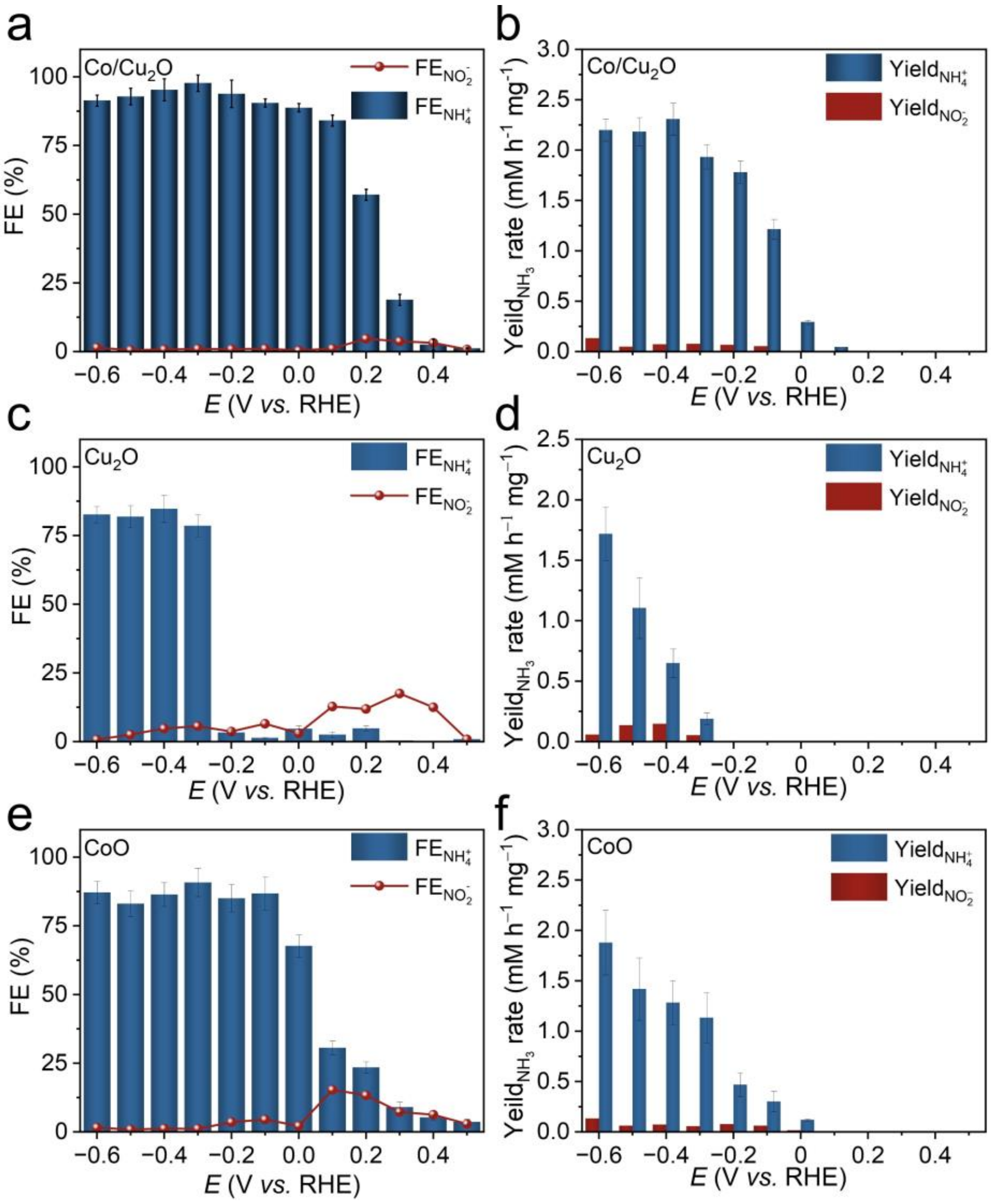

Figure R4 (**Supplementary Figure 33c,d, 38b,c, and 39b,c**) FE plots, and NH_3 yield rate at different potential of (a,b) Co/Cu_2O, (c,d) Cu_2O, and (e,f) CoO, respectively.

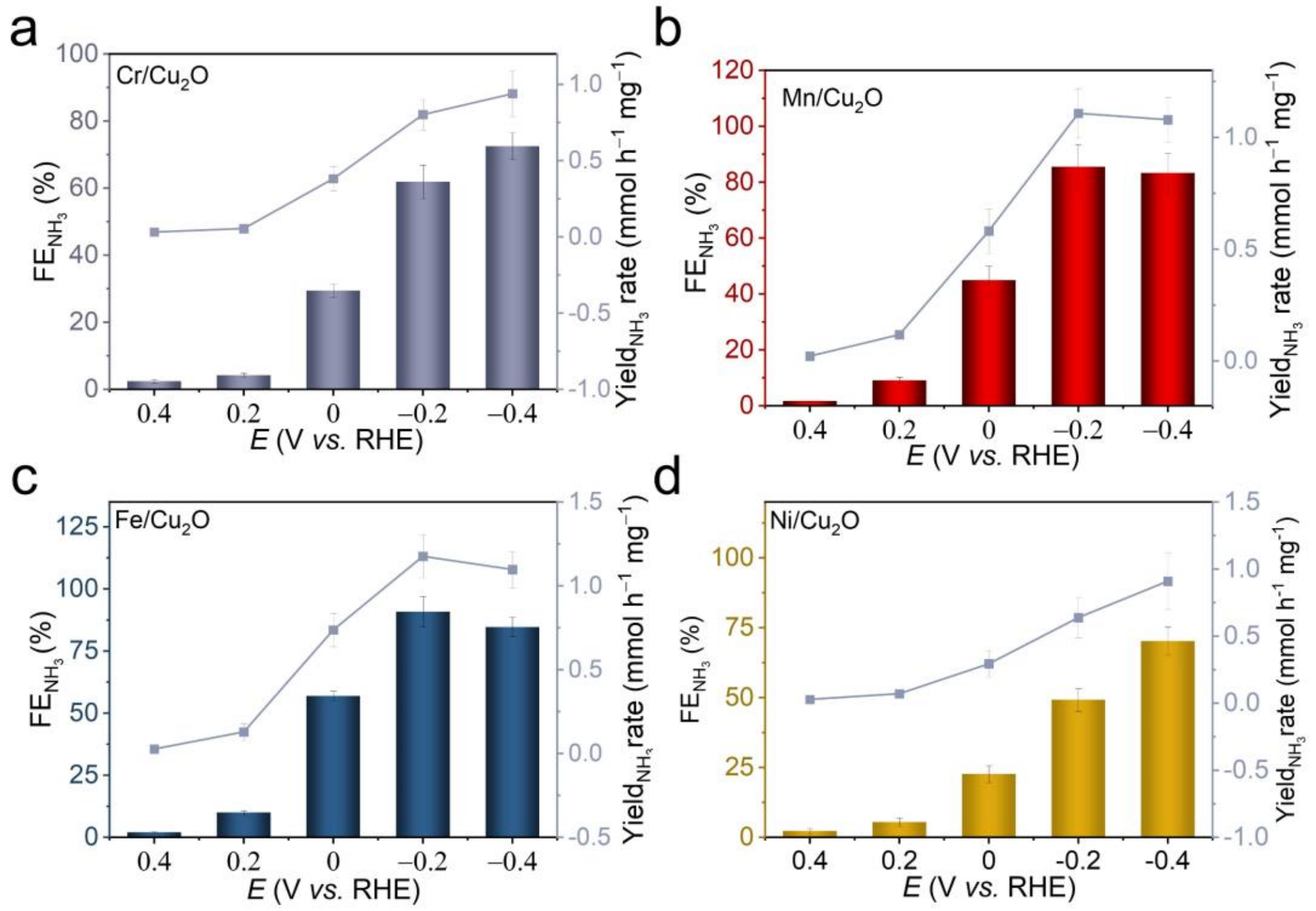

Figure R5 (**Supplementary Figure 36**) The FE plots and NH_3 yield rate plots of (a) Cr/Cu_2O, (b) Mn/Cu_2O, (c) Fe/Cu_2O, and (d) Ni/Cu_2O at different potential, respectively.

Specific Comment R1-6: *The authors investigated the intermediates using the in-situ FTIR technique and obtained the main signal of NH_2*. To further underpin the reaction pathway in Figure 5a, in situ DEMS experiments should be performed to provide more detailed information on intermediates.*

Response: Thank you for this valuable suggestion. We have now performed *in-situ* differential electrochemical mass spectrometry (DEMS) experiments to directly identify the key gas-phase and volatile intermediates/products during NO_3RR over Co/Cu_2O, providing unambiguous evidence for the proposed reaction pathway.

As shown in the DEMS spectra (Figure R6), sequential mass signals corresponding to critical intermediates and the final product are clearly observed over time:

Early-stage intermediates: Transient signals for NO_2 (*m/z* = 46), NO (*m/z* = 30), and NOH (*m/z* = 31) appear first, consistent with the initial nitrate reduction and oxygen-scission steps.

Nitrogen-hydrogen species: Following these, signals for NH_2 (*m/z* = 16) and NH_3 (*m/z* = 17) emerge and progressively intensify, matching the NH_2* intermediate detected in *in-situ* FTIR results.

The time-resolved evolution of these mass traces directly confirms the cascade reduction pathway: NO_3^- → NO_2^- → NO → NOH → NH_2 → NH_3. This complementary DEMS data, combined with the FTIR observations, provides comprehensive mechanistic evidence for the stepwise NO_3RR process on Co/Cu_2O. These results have been incorporated into the revised manuscript.

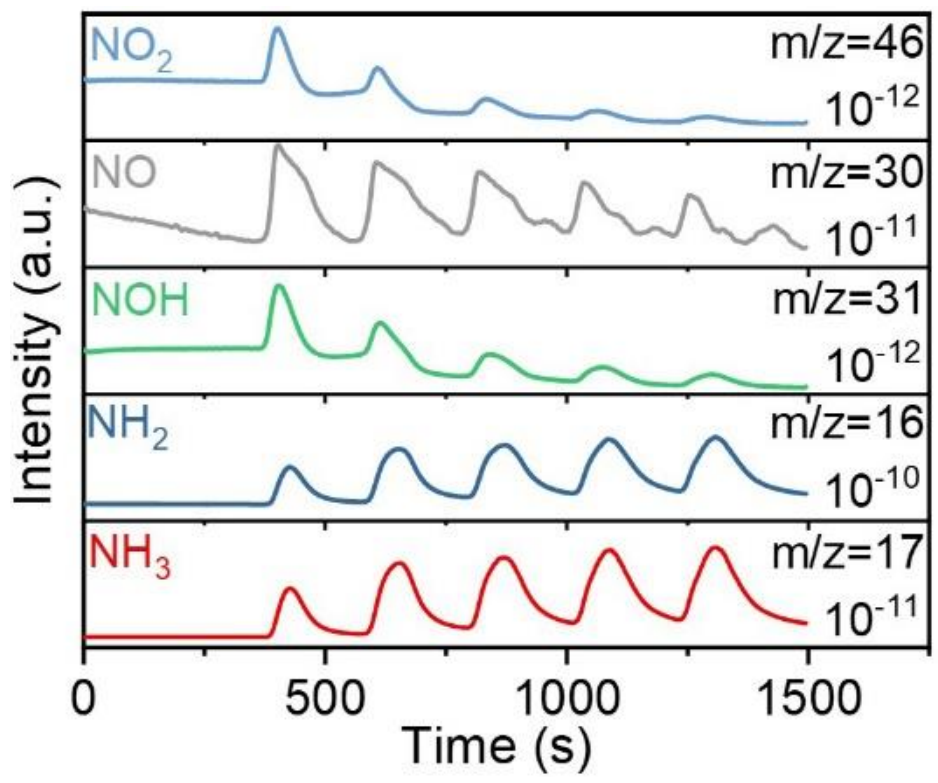

Figure R6 (Figure 4f) In-situ DEMS spectra of electrocatalytic NO_3^- reduction over Co/Cu_2O.

"Besides, as shown in Figure 4f, the *in-situ* differential electrochemical mass spectrometry

(DEMS) study reveals the dynamic evolution of key reaction intermediates and products. Mass signals corresponding to NO_2 (m/z = 46), NO (m/z = 30), and NOH (m/z = 31) appear early in the reaction, confirming the sequential formation of nitrogen-oxygen intermediates. Notably, signals for NH_2 (m/z = 16) and NH_3 (m/z = 17) emerge shortly afterward, with their intensities progressively increasing over time. This sequential evolution directly corroborates the stepwise reaction pathway: $NO_3^- \rightarrow NO_2^- \rightarrow NO \rightarrow NOH \rightarrow NH_2 \rightarrow NH_3$. Combined with the NH_2* intermediate signal observed in *in-situ* FTIR, these DEMS results provide unambiguous mechanistic evidence for the NO_3^- to NH_3 conversion process on the Co/Cu_2O catalyst." (Page 14, L370-378)

Specific Comment R1-7: *For the in-situ Raman test, given that the carbon nanotube was introduced during the catalyst synthesis. It is challenging to discern the peaks of adsorbed NO_3^- at 1352 cm^{-1} because of the D peak of carbon materials. Besides, the signal between 200 and 800 also needs to be shown to demonstrate the structural stability of Cu_2O.*

Response: We sincerely appreciate the reviewer's insightful and constructive comments, which have significantly improved the rigor of our *in-situ* Raman analysis. We have addressed both points thoroughly as follows:

1) Overlap between the carbon D band and adsorbed nitrate peak at 1352 cm^{-1}

We fully acknowledge this potential interference. To resolve this issue, we have added in-situ Raman spectra of pristine carbon nanotubes (CNTs) under identical NO_3RR conditions as a blank control. As shown in the control spectra, the peak at ~1350 cm^{-1} is exclusively the D band of sp^2-hybridized carbon, which remains nearly unchanged across all potentials. Notably, no additional reaction-related peaks are observed in pure CNTs except for the weak $*NO_2^-$ signal at ~1040 cm^{-1}.

In contrast, both Co/Cu_2O and Cu_2O catalysts exhibit a distinct, well-resolved peak at ~1320 cm^{-1} that is absent in pure CNTs. We have corrected our peak assignment in the revised manuscript: this 1320 cm^{-1} peak corresponds to adsorbed nitric oxide (*NO) intermediate, not adsorbed NO_3^-. The rapid adsorption and activation of NO_3^- on Cu-based catalysts leads to extremely low steady-state concentration of adsorbed NO_3^-, which is below the detection limit of our in-situ Raman system. The complete reaction pathway is reliably tracked by the unambiguous intermediate peaks at ~1040 cm^{-1} ($*NO_2^-$), ~1320 cm^{-1} (*NO), ~1460 cm^{-1} ($*NH_2$), and ~1560 cm^{-1} ($*NH_3$), all of which are absent in the pure CNT control.

2) Demonstration of Cu_2O structural stability in the 200–800 cm^{-1} region

We thank the reviewer for this critical suggestion. We have now included the full-range in-situ Raman spectra (200–1200 cm^{-1}) for both Co/Cu_2O and Cu_2O catalysts in the revised manuscript. In addition to the previously discussed Cu-O stretching vibration at ~1090 cm^{-1}, two characteristic Cu_2O peaks are clearly resolved in this region: the peak at ~218 cm^{-1} corresponds to the second-order phonon mode of cubic Cu_2O, and the peak at ~630 cm^{-1} is assigned to the Cu-O bending vibration.

As the potential shifts negatively from 0.5 V to -0.6 V, all three Cu_2O characteristic peaks (218, 630, and 1090 cm^{-1}) show much more severe intensity attenuation for pristine Cu_2O compared to Co/Cu_2O. This direct spectroscopic evidence confirms that cobalt doping effectively suppresses the over-reduction of the Cu_2O active phase and preserves its crystalline structure under reductive NO_3RR conditions, strongly supporting our conclusion on the enhanced structural stability of the Co/Cu_2O catalyst.

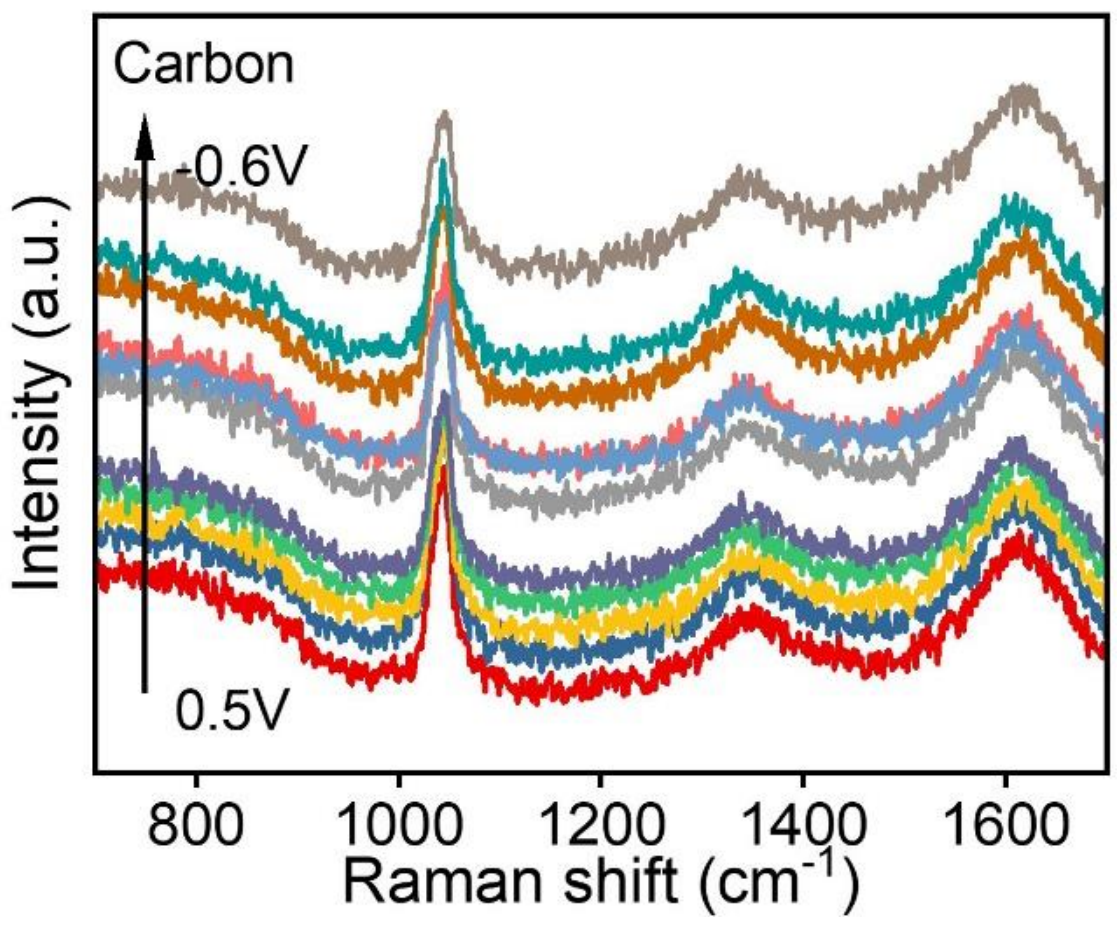

Figure R7 In-situ Raman spectra of CNT material at different applied potentials for NO_3RR.

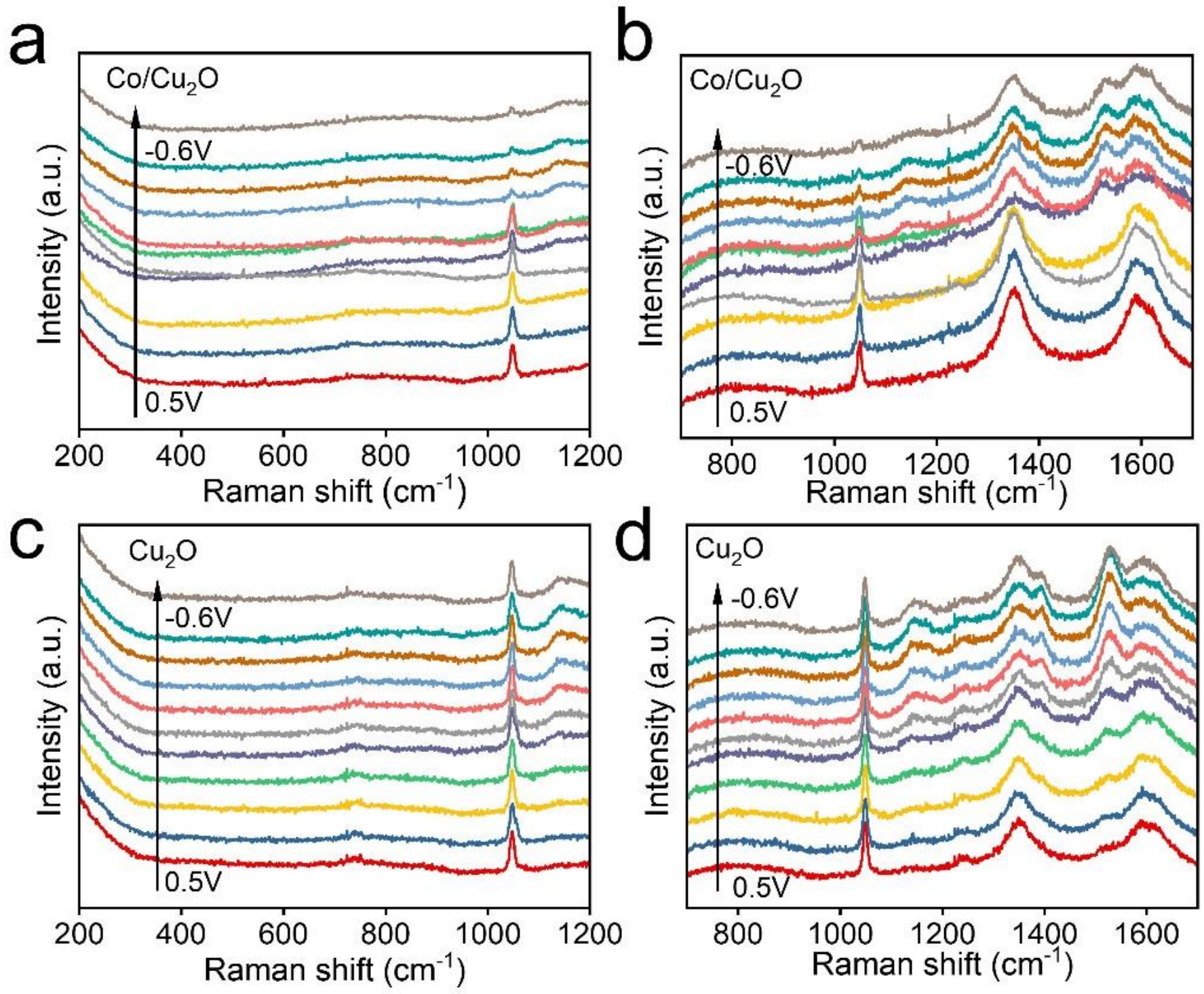

Figure R8 (Supplementary Figure 51) In-situ Raman spectra of (a,b) Co/Cu_2O and (c,d) Cu_2O at different applied potentials for NO_3RR.

Specific Comment R1-8: *8. To concisely analyze the valence of Cu, it is suggested to provide the LMM Auger spectra in the related XPS discussions.*

Response: We thank the reviewer for the valuable suggestion. To better analyze the valence state of Cu, we have conducted Cu LMM Auger electron spectroscopy on both Co/Cu_2O and Cu_2O. This confirms the Cu^I oxidation state in both samples. The spectra have been added as Supplementary Fig. S15, and the discussion has been incorporated into the revised manuscript (Page 7).

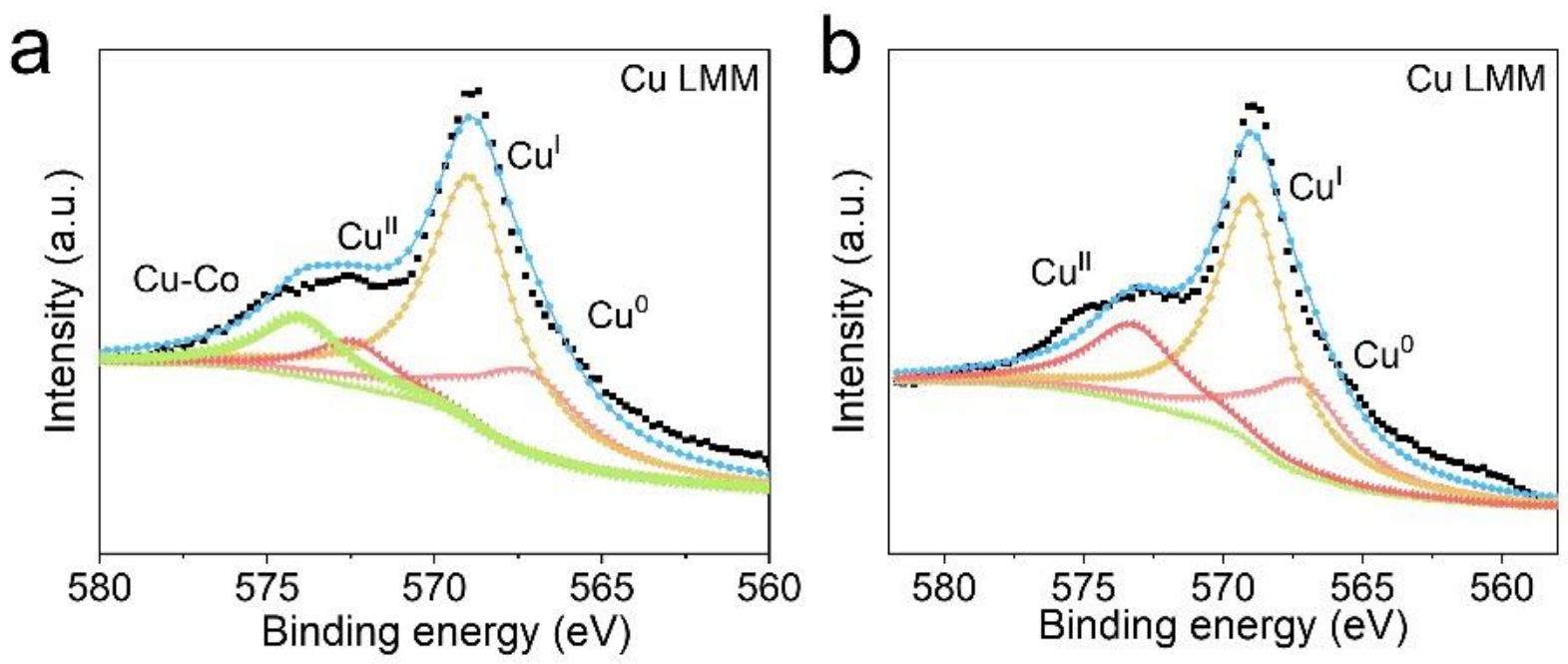

Figure R9 (Supplementary Figure 15) Deconvolution of the Cu LMM Auger spectra for (a) Co/Cu_2O and (b) Cu_2O catalysts.

"To further resolve the chemical state of Cu, we performed Cu LMM Auger electron spectroscopy on Co/Cu_2O and Cu_2O (Figure S15). Both samples exhibit a dominant Cu^I peak at ~570 eV, confirming that the Cu_2O matrix remains the primary Cu-containing phase. *Applied Catalysis B: Environmental 207 (2017) 134–142* Notably Co/Cu_2O shows an additional Auger feature at ~576 eV, attributed to interfacial Cu–Co species, along with a higher relative fraction of Cu^{II} compared to pristine Cu_2O. These differences reflect a strong electronic interaction between Co and the Cu_2O lattice, which promotes partial oxidation of Cu^I and the formation of interfacial Cu–Co species. Such modulation of Cu's valence distribution and electronic environment is expected to influence catalytic performance significantly." (Page 7, Line168-176)

Specific Comment R1-9: *The temperature range used for the Arrhenius analysis is relatively narrow, which may limit the reliability of the extracted activation energies. Besides, it is suggested to provide a photo of the setup with temperature control for the experiments*.

Response: We acknowledge that the temperature range (273-313 K, i.e., 0-40 °C) is relatively narrow. This range was chosen because the electrochemical measurements become unstable below 273 K (freezing) and above 313 K due to excessive gas evolution and electrolyte evaporation. Despite the narrow range, the linear fits in the Arrhenius plots show $R^2 > 0.98$, indicating that the extracted *Ea* values are statistically reliable. We have added a discussion of this limitation in the revised SI.

We have also provided a photograph of the temperature-controlled electrochemical setup. The cell was immersed in a water bath with a thermostatic controller (accuracy ± 0.5°C), and the temperature was monitored with a digital thermometer.

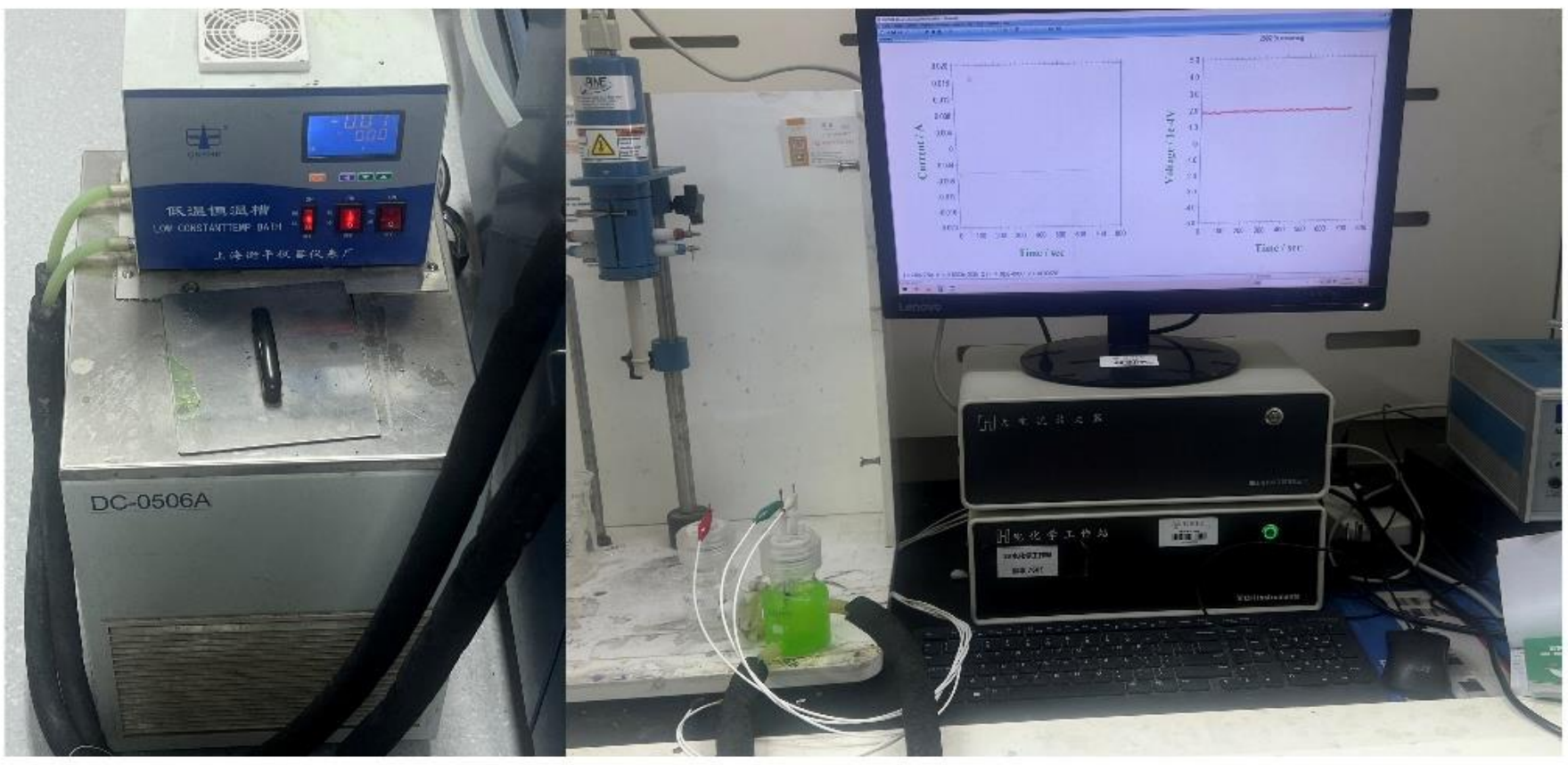

Figure R10 (Supplementary Figure 48) A digital photo of the temperature-controlled electrochemical setup at different temperatures.

Specific Comment R1-10: *Due to the selected different adsorption modes for the *NO_3 on different systems (such as Top coordination: M-N or M-O, Bridge coordination), it is suggested to show the calculated details to demonstrate the reason for the selection of different modes.*

Response: We sincerely appreciate the reviewer's constructive and insightful suggestion regarding the rigorous justification of *NO_3 adsorption mode selection. To comprehensively address this concern, we have systematically calculated all thermodynamically plausible adsorption configurations of nitrate on the Co/Cu_2O surface, including three distinct monodentate metal-oxygen (M-O) binding modes (where each of the three chemically equivalent terminal oxygen atoms of *NO_3 individually binds to the Co dopant site) and all possible metal-nitrogen (M-N) binding motifs where the central N atom directly interacts with the surface Co site. Notably, no stable bidentate or tridentate M-O configurations were identified in our calculations, as all such initial structures spontaneously relaxed to monodentate M-O modes during geometry optimization.

As presented in **Figure R11**, all three monodentate M-O configurations exhibit strong exothermic adsorption, with adsorption energies of -2.83 eV (**Figure R11**a), -2.68 eV (**Figure R11**b), and -2.51 eV (**Figure R11**c), respectively. The subtle energy differences among these three modes arise from the asymmetric local coordination environment of the Co dopant site and the varying steric interactions between the unbound oxygen atoms and the underlying Cu_2O lattice.

In stark contrast, all explicitly calculated M-N coordination modes showed drastically weaker adsorption energies and spontaneously restructured into the most stable monodentate M-O configuration during optimization. This unambiguously demonstrates that the central nitrogen atom cannot serve as a stable binding site on the Co/Cu_2O surface, and direct M-N adsorption is thermodynamically and kinetically unfavorable.

This overwhelming preference for monodentate M-O binding originates from two fundamental factors: (1) the highly electronegative oxygen atoms in NO_3 form substantially stronger electrostatic attractions and covalent bonds with the positively charged Co^{3+}/Co^{2+} and Cu^{+} surface cations; (2) the central nitrogen atom is sterically shielded by the three surrounding oxygen atoms, which prevents effective orbital overlap between the N 2*p* orbitals and the

surface metal *d*-orbitals. This computational conclusion is further corroborated by our in-situ Raman spectroscopy results, which unambiguously detected oxygen-bound $*NO_2$ intermediates during the reaction.

For subsequent reaction pathway calculations, we selected the thermodynamically most favorable monodentate M-O configuration as the initial state. We also verified that even if the reaction starts from the other two less stable monodentate configurations, they will first relax to the most stable configuration before undergoing further hydrogenation steps, with their corresponding initial hydrogenation barriers being 0.15 eV and 0.32 eV higher, respectively. All these calculated details have been added to the revised manuscript.

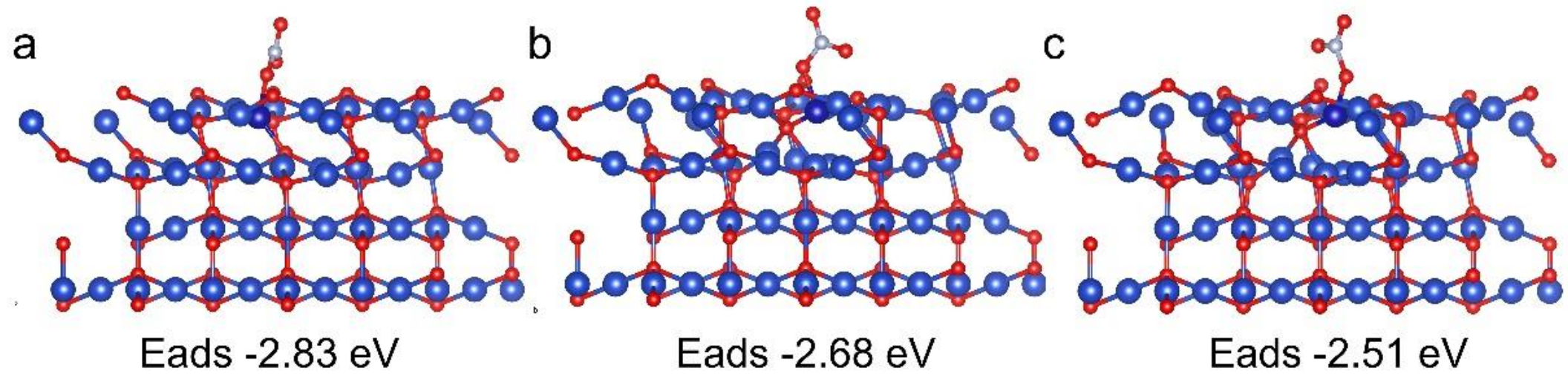

Figure R11 Adsorption modes of different systems $*NO_3$ on Co/Cu_2O model surfaces.

Specific Comment R1-11: *It is necessary to conduct theoretical calculations at the GGA+U level with the Hubbard U correction, especially for the transition metal oxide systems.*

Response: We sincerely thank the reviewer for raising this important point regarding the necessity of performing theoretical calculations at the GGA+*U* level, especially for transition metal oxide systems such as Cu_2O and CoO. We fully agree that Hubbard *U* corrections are often critical for properly describing the electronic structure of strongly correlated d electrons in these materials. In response to this concern, we have systematically performed additional DFT calculations with the GGA+*U* method for all key models in this study, including Co/Cu_2O, Ni/Cu_2O, Cu_2O, and CoO. The *U* values were applied to the transition metal d orbitals following established literature values (Co = 3.5 eV, Cu = 4.0 eV, Ni = 6.0 eV). We then reevaluated the projected density of states (PDOS) and the integrated crystal orbital Hamilton population (ICOHP) for the adsorption of key intermediates (*NO_3, *NO, and *NH_3). The detailed results are presented in **Figures R12-R16**.

After a careful comparison between the standard GGA and GGA+*U* results, we observed that the inclusion of the Hubbard *U* correction did not alter the relative catalytic trends among the four catalysts. Specifically, the same activity order was maintained. The PDOS profiles showed expected shifts in the *d* band centers, but the overall hybridization strengths between the metal d orbitals and the adsorbate *p* orbitals followed the same pattern. The ICOHP values, which quantify the bonding strength between the metal sites and the reaction intermediates, also exhibited a consistent trend across both computational levels. For example, Co/Cu_2O consistently showed the strongest interaction with *NO_3 and the most favorable energy profile for further hydrogenation steps.

There are several reasons why the relative trends remain robust even without *U* correction in this particular system. First, our primary comparison focuses on differences between similar transition metal doped oxides rather than on absolute energetics. The error introduced by the lack of *U* tends to cancel out when comparing analogous systems. Second, the key adsorbates (NO_3, NO, NH_3) interact strongly with the metal sites, and the hybridization effects dominate over the subtle electron correlation effects that *U* is meant to correct. Third, the trend in activity is largely governed by geometric and local coordination effects rather than by the absolute *d*

band position alone. These structural features are well captured by standard GGA.

Nevertheless, we acknowledge that the GGA+*U* level is more rigorous for describing the electronic ground states of transition metal oxides. Therefore, we have now included the GGA+*U* results in the **Figures R12–R16** and have updated the corresponding discussions in the main manuscript. Importantly, the key conclusions of our study, that the Co/Cu_2O interface exhibits superior nitrate reduction activity due to optimized intermediate binding, remain unchanged and are even strengthened by the consistency between the two computational levels.

We deeply appreciate the reviewer's rigorous suggestion, which has prompted us to validate our theoretical framework more thoroughly. We have added a clear statement in the "Computational Methods" section of the revised manuscript to justify our choice of the GGA+*U* approach and to explain the robustness of the observed trends. We believe these revisions now fully address the reviewer's concern.

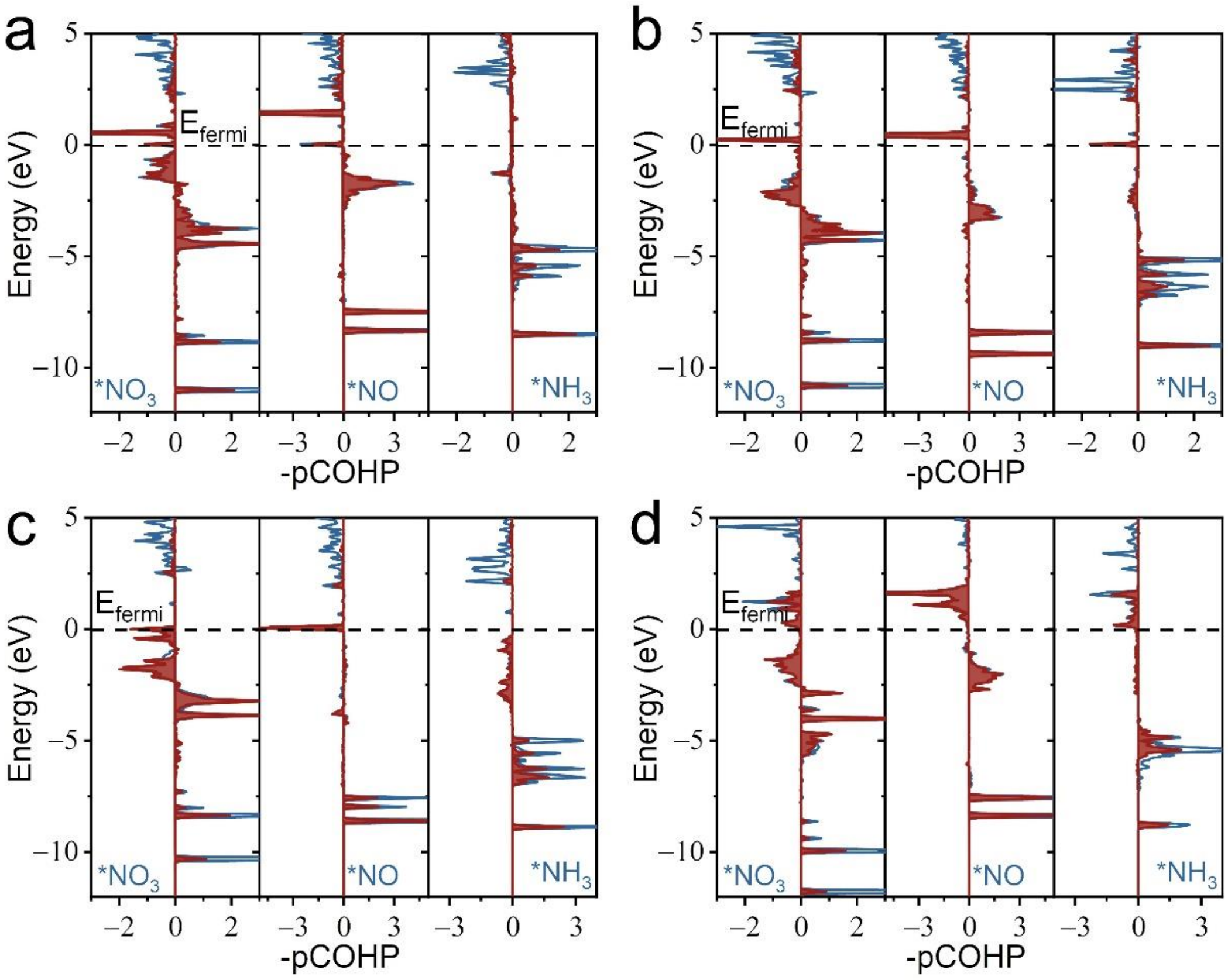

Figure R12 (Supplementary Figure 62-65) Integrated crystal orbital Hamilton population (ICOHP) evolution of (a) Co/Cu_2O, (b) Ni/Cu_2O, (c) Cu_2O, and (d) CoO, respectively.

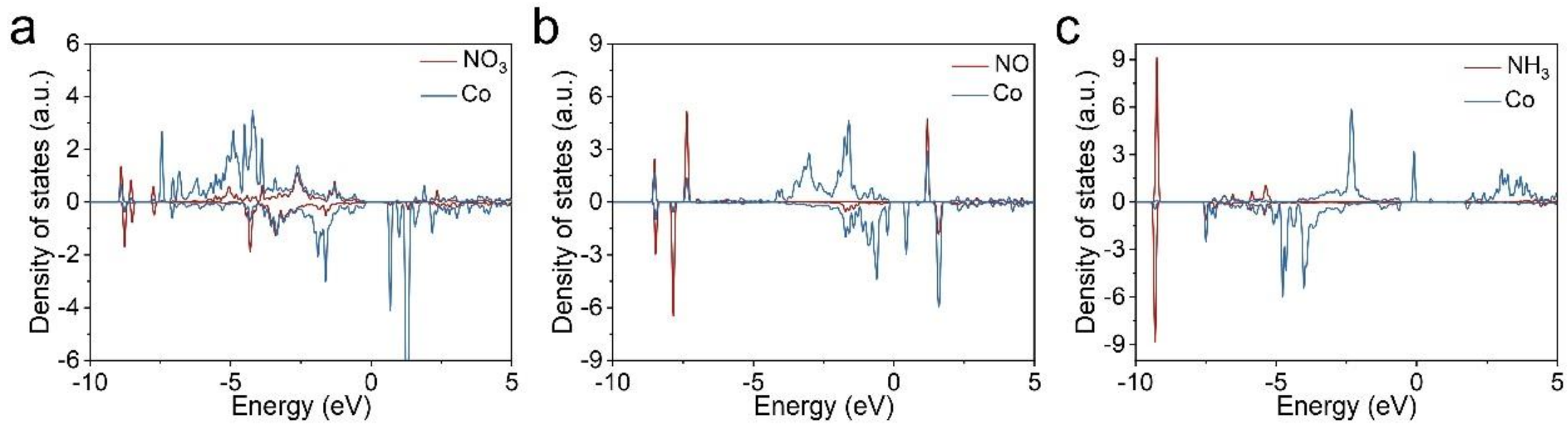

Figure R13 (Supplementary Figure 66) PDOS of Co 3*d* orbital and (a) *NO_3, (b) *NO, (c) *NH_3 on Co/Cu_2O at the GGA+*U* level with the Hubbard *U* = 3.3 eV, respectively.

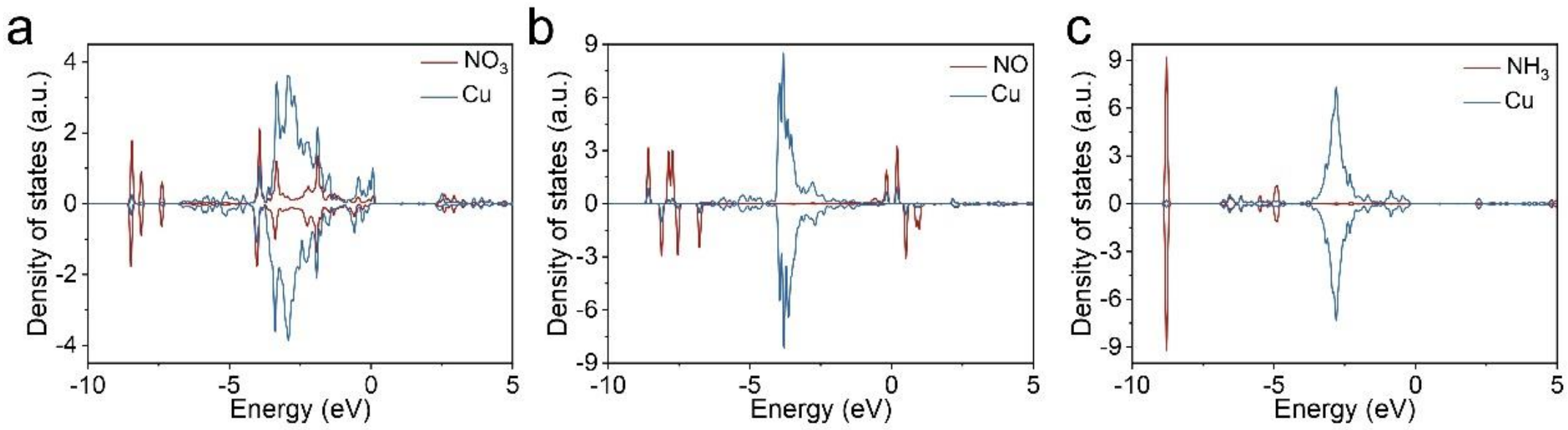

Figure R14 (Supplementary Figure 67) PDOS of Cu 3*d* orbital and (a) *NO_3, (b) *NO, (c) *NH_3 on Cu_2O at the GGA+*U* level with the Hubbard *U* = 5.0 eV, respectively.

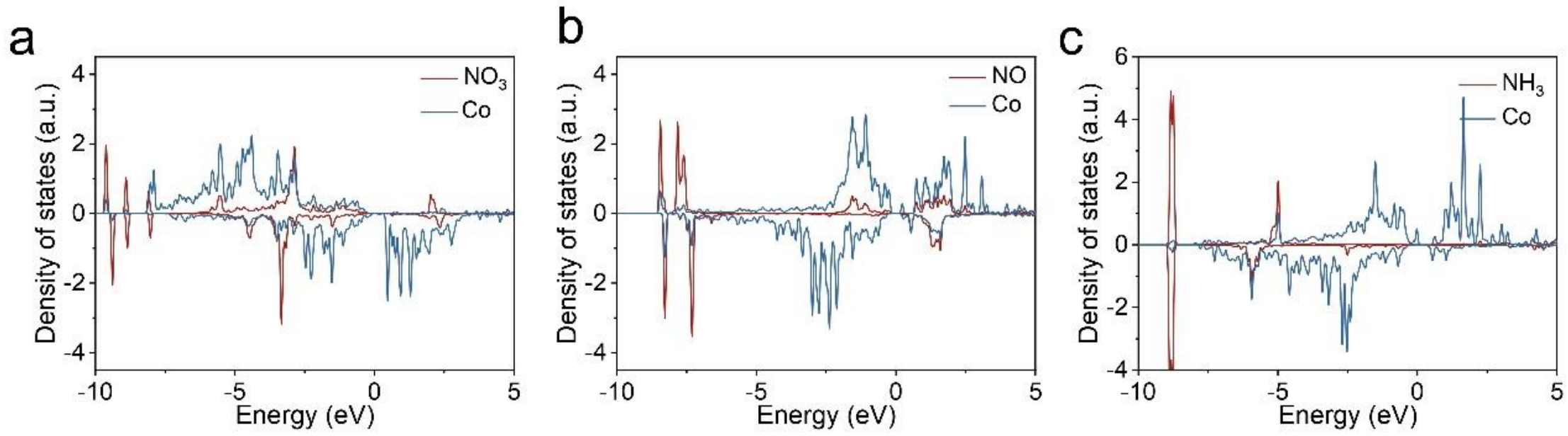

Figure R15 (Supplementary Figure 68) PDOS of Co 3*d* orbital and (a) *NO_3, (b) *NO, (c) *NH_3 on CoO at the GGA+*U* level with the Hubbard *U* = 3.3 eV, respectively.

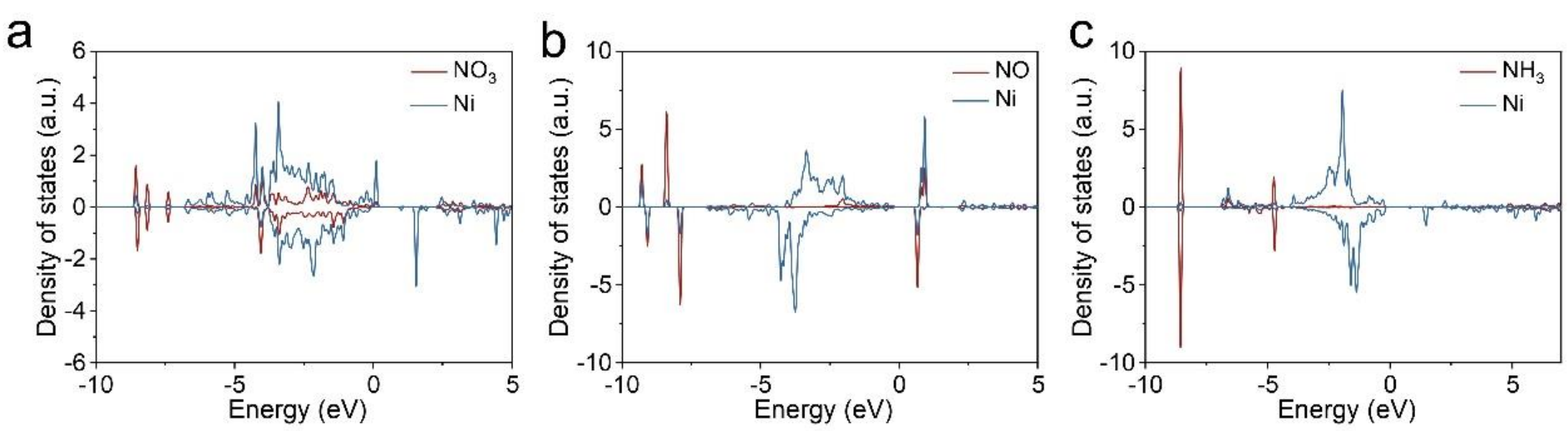

Figure R16 (Supplementary Figure 69) PDOS of Ni 3*d* orbital and (a) *NO_3, (b) *NO, (c) *NH_3 on Ni/Cu_2O at the GGA+*U* level with the Hubbard *U* = 6.0 eV correction, respectively.

Specific Comment R1-12: *To demonstrate the rationality of built structural modes in DFT calculations, such as Figures S4 and S5, the tables about the structural parameters extracted from the EXAFS fitting need to be supplemented for all samples.*

Response: We thank the reviewer for this important suggestion. To demonstrate the rationality of the structural models used in DFT calculations (**Figure R11-R15**), we have now performed quantitative EXAFS fitting for all metal-doped Cu_2O samples (Cr, Mn, Fe, Co, Ni) and for the corresponding reference compounds. The complete set of fitting parameters is provided in **Tables R1-R5**.

The DFT models were constructed using the EXAFS-derived structural parameters as key constraints:

Bond lengths (*R*): The experimentally determined M–O distances (1.89–1.98 Å) were used as initial values for geometry optimization. The final DFT-optimized bond lengths deviate by less than 0.03 Å from the EXAFS values, confirming the reliability of the models.

Coordination numbers (*CN*): The EXAFS *CN* values (ranging from 3.8 to 5.1) reflect the local coordination environment. For Co/Cu_2O, the *CN* of 4.0 indicates a distorted, lower-coordination site, which was explicitly modeled in DFT by placing Co at a substitutional Cu site with local relaxation. For other dopants, the higher *CN* (~4–5) suggests more oxygen-rich or amorphous-like local structures, which were modeled with a flexible coordination sphere.

Second-shell information: The presence of a Co–Cu path ($R = 2.85$ Å) directly validates the substitutional model for Co. In contrast, the absence of second-shell peaks for Cr, Mn, Fe, and Ni indicates that these dopants do not occupy well-defined lattice sites; their DFT models were therefore constructed with a more disordered or amorphous local environment.

Oxidation states: The bond lengths are consistent with known metal–oxygen distances for trivalent or divalent states (e.g., Co–O ~1.89 Å, Fe–O ~1.92 Å, Cr–O ~1.98 Å), which were used to set the initial magnetic moments and charge states in DFT calculations.

All fits satisfy the Nyquist criterion (number of independent data points ~16–20, fitted parameters 5–7). The *R*-factor values (< 0.02) indicate good fit quality. The raw $k^3\chi(k)$ data and FT-EXAFS spectra are provided in **Figure R17-R21** for full transparency.

We believe these additions provide the necessary structural validation for the DFT models

and fully address the reviewer's concern. All changes are highlighted in yellow in the revised manuscript and Supporting Information.

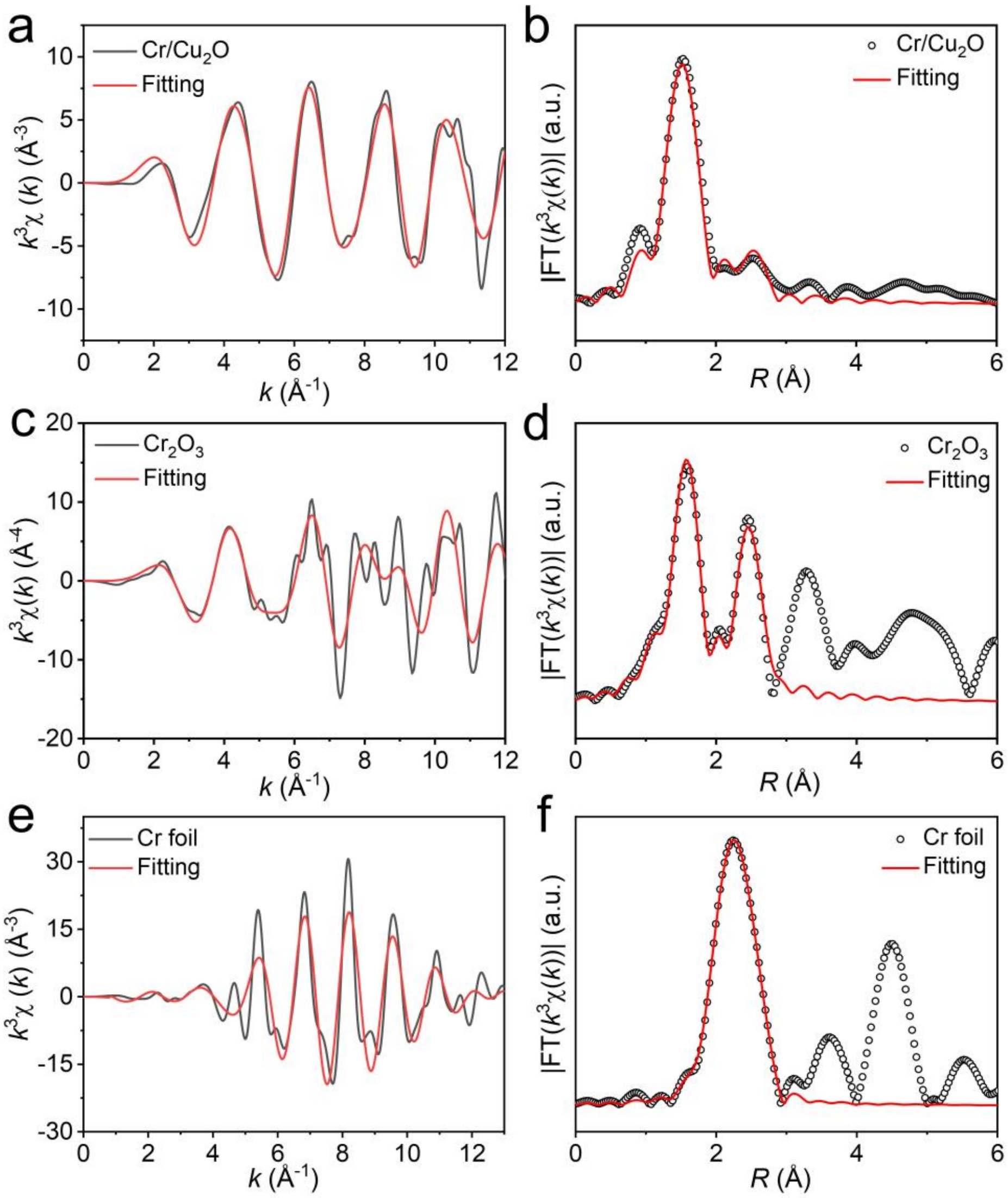

Figure R17 (Supplementary Figure 27) (a, c, e) k^3-weighted χ(k) oscillation curves and their corresponding fitting curves for Cr/Cu_2O, Cr_2O_3, and Cr foil, respectively. (b, d, f) Fourier-transformed (FT) $k^3\chi(R)$ spectra and their corresponding fitting curves for Cr/Cu_2O, Cr_2O_3, and Cr foil, respectively.

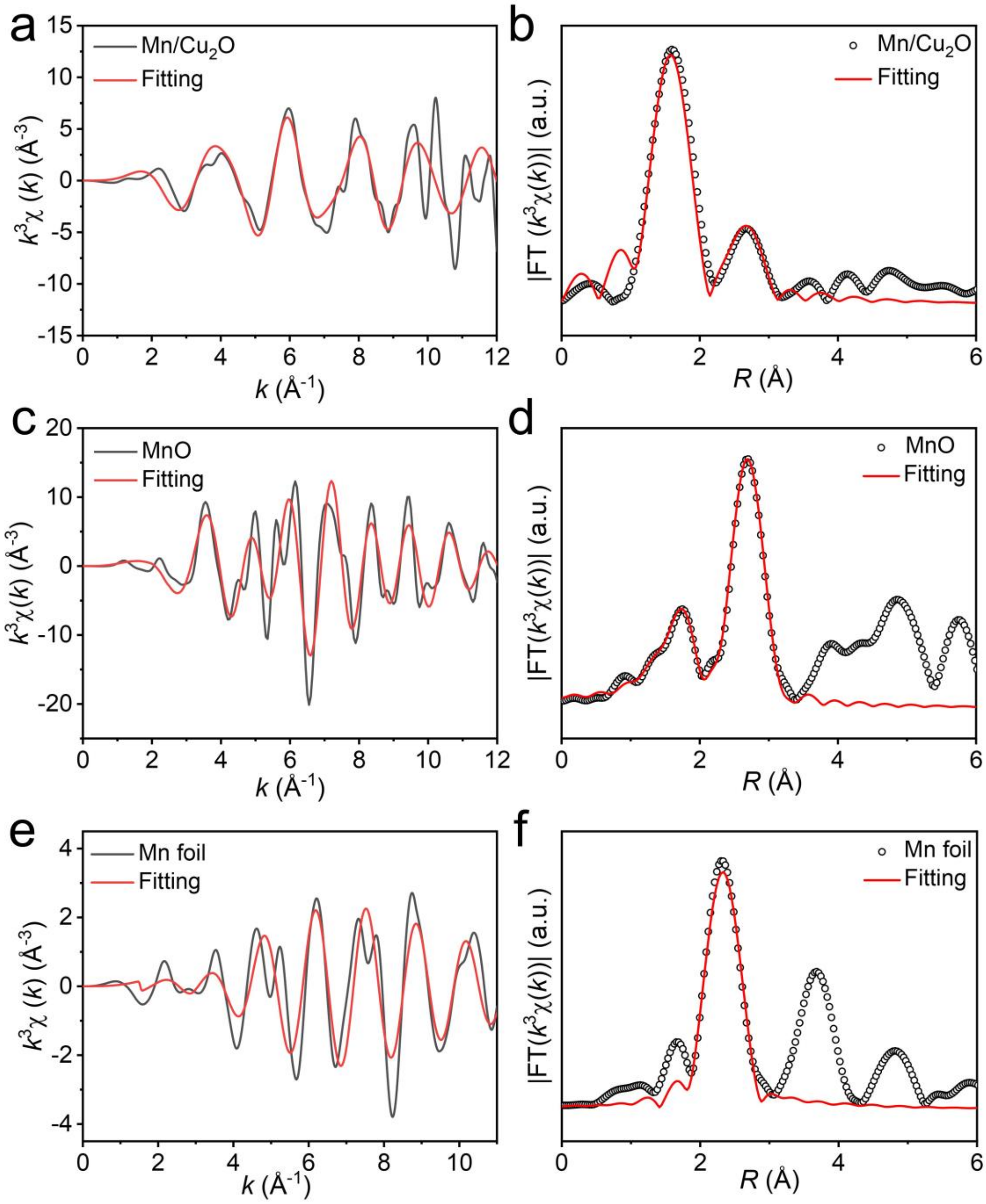

Figure R18 (Supplementary Figure 28) (a, c, e) k^3-weighted $\chi(k)$ oscillation curves and their corresponding fitting curves for Mn/Cu_2O, MnO, and Mn foil, respectively. (b, d, f) Fourier-transformed (FT) $k^3\chi(R)$ spectra and their corresponding fitting curves for Mn/Cu_2O, MnO, and Mn foil, respectively.

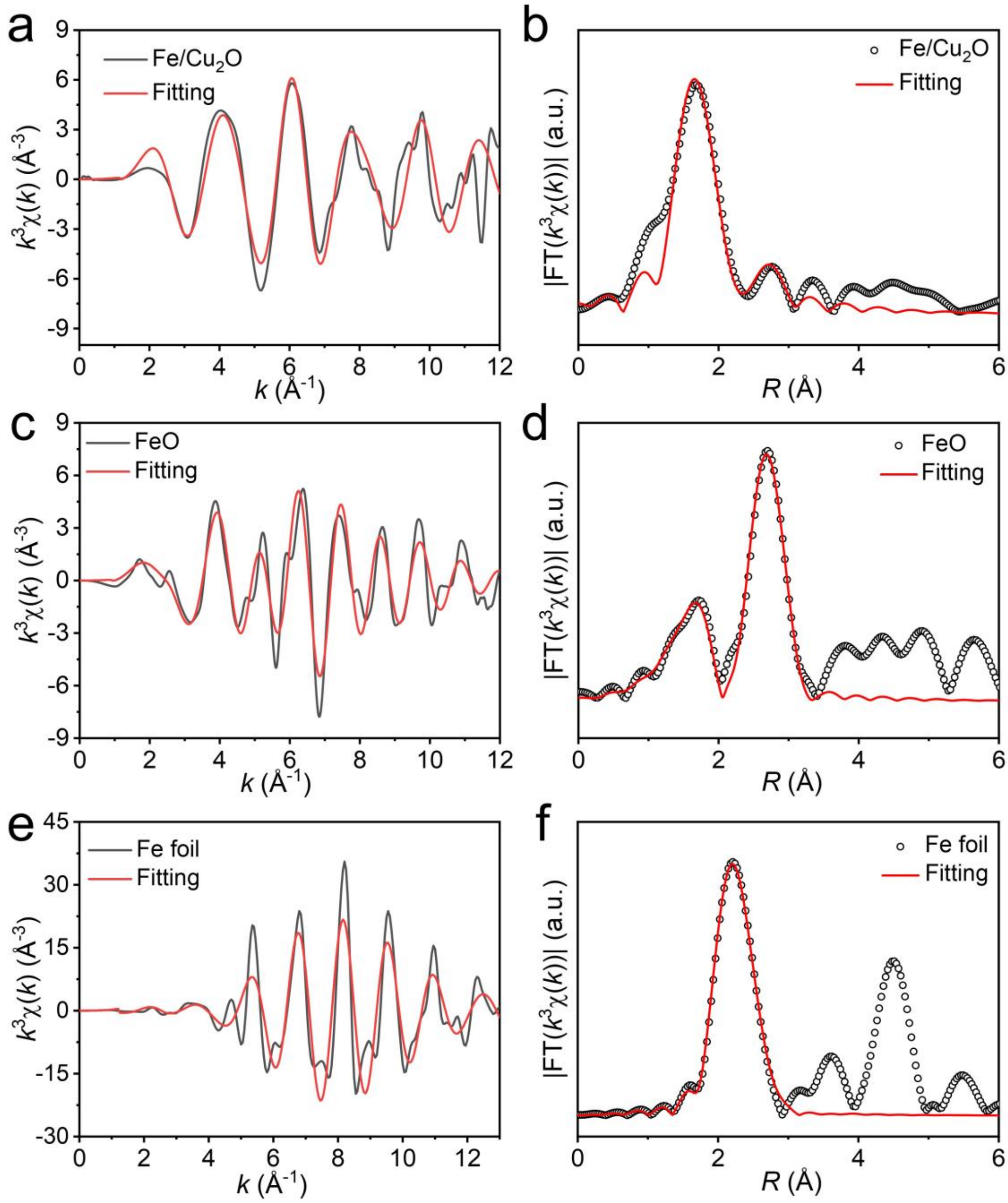

Figure R19 (Supplementary Figure 29) (a, c, e) k^3-weighted $\chi(k)$ oscillation curves and their corresponding fitting curves for Fe/Cu_2O, FeO, and Fe foil, respectively. (b, d, f) Fourier-transformed (FT) $k^3\chi(R)$ spectra and their corresponding fitting curves for Fe/Cu_2O, FeO, and Fe foil, respectively.

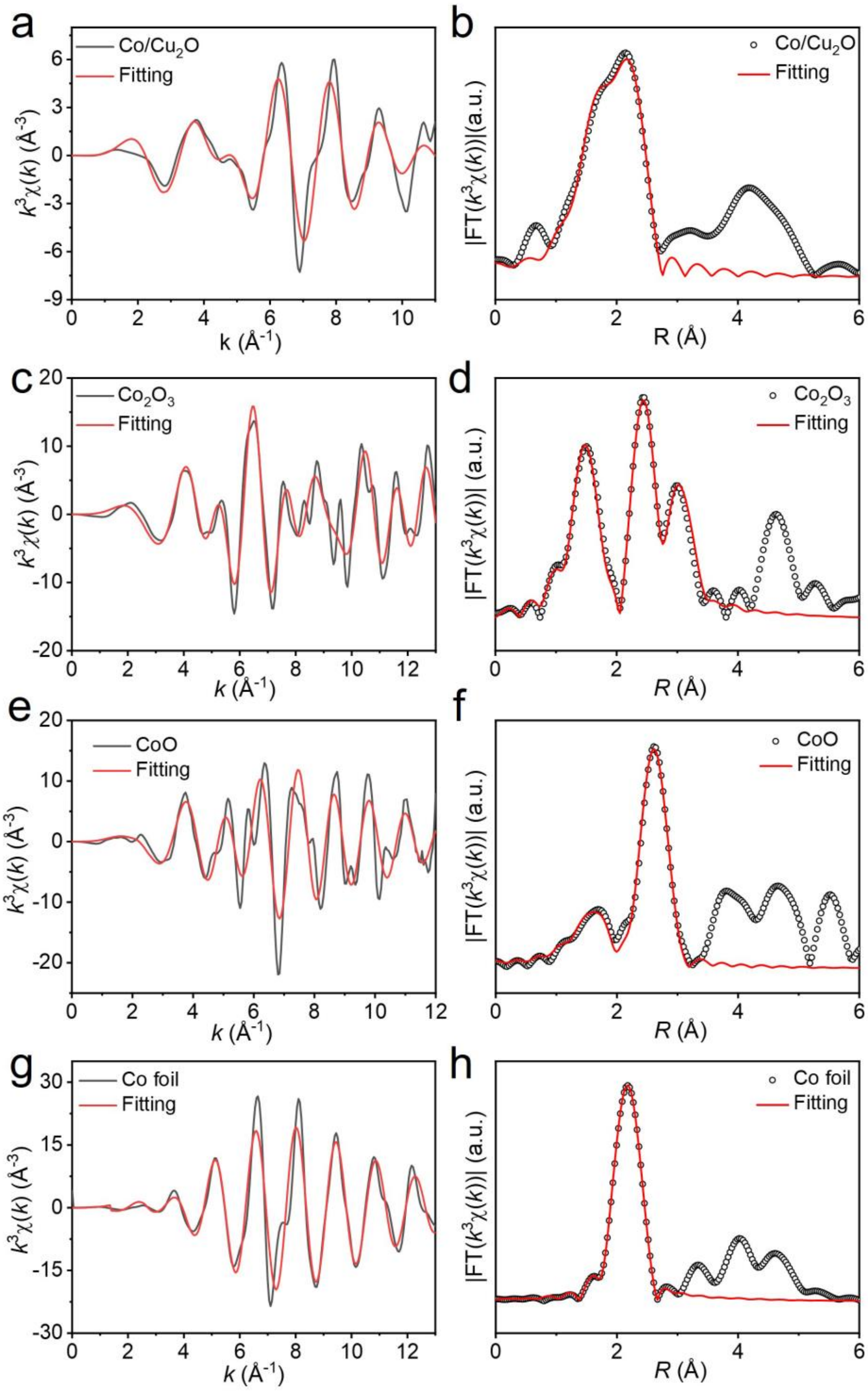

Figure R20 (Supplementary Figure 26) (a, c, e, g) k^3-weighted $\chi(k)$ oscillation curves and their corresponding fitting curves for Co/Cu_2O, Co_2O_3, CoO, and Co foil, respectively. (b, d, f, h) Fourier-transformed (FT) $k^3\chi(R)$ spectra and their corresponding fitting curves for Co/Cu_2O, Co_2O_3, CoO, and Co foil, respectively.

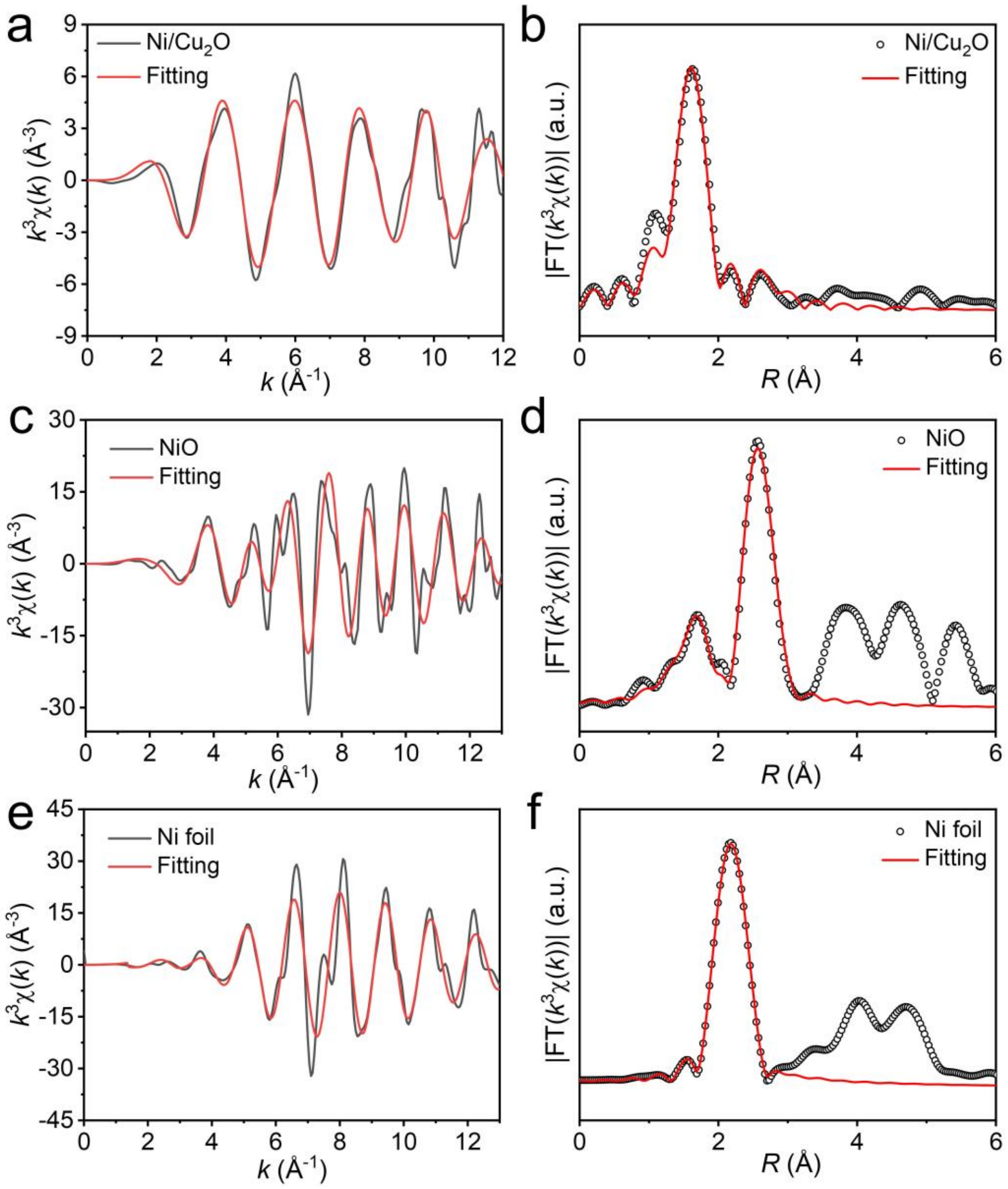

Figure R21 (Supplementary Figure 30) (a, c, e) k^3-weighted $\chi(k)$ oscillation curves and their corresponding fitting curves for Ni/Cu_2O, NiO, and Ni foil, respectively. (b, d, f) Fourier-transformed (FT) $k^3\chi(R)$ spectra and their corresponding fitting curves for Ni/Cu_2O, NiO, and Ni foil, respectively.

Table R1 (Supplementary Table 4) EXAFS fitting parameters at the Cr *K*-edge for various samples. (S_0^2 = 0.78 from Cr-foil)

	shell	CN^a	R^b(Å)	σ^{2c}(Å^2)	ΔE_0^d(eV)	*R* factor
Cr-foil	Cr-Cr	8	2.49±0.01	0.0067	2.7±0.9	0.0058
	Cr-Cr	6	2.86±0.01	0.0045		
Cr_2O_3	Cr-O	6.0±0.3	1.99±0.01	0.0025	-7.4±0.8	0.0101
	Cr-Cr	1.0±0.2	2.61±0.01	0.0026		
	Cr-Cr	4.0±0.3	2.89±0.01	0.0049		
Cr/Cu_2O	Cr-O	4.7±0.3	1.97±0.01	0.0030	-3.9±0.7	0.0159
	Cr-Cu	1.3±0.5	2.94±0.01	0.0082		

^{a}*CN*: coordination numbers; ^{b}*R*: bond distance; $^c\sigma^2$: Debye-Waller factors; $^d\Delta E_0$: the inner potential correction. *R* factor: goodness of fit. A reasonable range of EXAFS fitting parameters: $0.70 < S_0^2 < 1.00$; $CN > 0$; $\sigma^2 > 0$ Å^2; $|\Delta E_0| < 15$ eV; *R* factor < 0.02.

Table R2 (Supplementary Table 5) EXAFS fitting parameters at the Mn *K*-edge for various samples. (S_0^2 = 0.71 from Mn-foil)

	shell	CN^a	R^b(Å)	σ^{2c}(Å^2)	ΔE_0^d(eV)	*R* factor
Mn-foil	Mn-Mn	2	2.58±0.01	0.0071	8.8±1.7	0.0184
MnO	Mn-O	6.1±0.3	2.21±0.01	0.0093	-5.0±0.5	0.0043
	Mn-Mn	12.2±0.4	3.13±0.01	0.0094		
Mn/Cu_2O	Mn-O	4.8±0.3	2.06±0.02	0.0032	-4.6±0.9	0.0188
	Mn-Mn	3.9±0.9	3.11±0.02	0.0140		

^{a}*CN*: coordination numbers; ^{b}*R*: bond distance; $^c\sigma^2$: Debye-Waller factors; $^d\Delta E_0$: the inner potential correction. *R* factor: goodness of fit. A reasonable range of EXAFS fitting parameters: $0.70 < S_0^2 < 1.00$; $CN > 0$; $\sigma^2 > 0$ Å^2; $|\Delta E_0| < 15$ eV; *R* factor < 0.02.

Table R3 (Supplementary Table 6) EXAFS fitting parameters at the Fe *K*-edge for various samples. ($S_0^2 = 0.77$ from Fe-foil)

	shell	CN^a	R^b(Å)	σ^{2c}(Å^2)	$\Delta E_0{}^d$(eV)	*R* factor
Fe-foil	Fe-Fe1	8	2.46±0.01	0.0050	5.7±1.0	0.0062
	Fe-Fe2	6	2.84±0.01	0.0060		
FeO	Fe-O	6.0±0.4	2.12±0.01	0.0144	3.4±0.4	0.0101
	Fe-Fe	12.2±0.5	3.07±0.02	0.0120		
Fe/Cu_2O	Fe-O	4.5±0.4	2.08±0.02	0.0032	5.1±1.4	0.0192
	Fe-Cu	1.7±0.4	2.94±0.02	0.0123		

^{a}*CN*: coordination numbers; ^{b}*R*: bond distance; $^c\sigma^2$: Debye-Waller factors; $^d\Delta E_0$: the inner potential correction. R factor: goodness of fit. A reasonable range of EXAFS fitting parameters: $0.70 < S_0^2 < 1.00$; $CN > 0$; $\sigma^2 > 0$ Å^2; $|\Delta E_0| < 15$ eV; *R* factor < 0.02.

Table R4 (Supplementary Table 3) EXAFS fitting parameters at the Co *K*-edge for various samples. (S_0^2 = 0.78 from Co-foil)

	shell	CN^a	R^b(Å)	σ^{2c}(Å^2)	ΔE_0^d(eV)	*R* factor
Co-foil	Co-Co	12	2.49±0.01	0.0063	7.3±0.3	0.0014
CoO	Co-O	5.9±0.4	2.12±0.01	0.0125	-3.8±0.3	0.0065
	Co-Co	12.2±0.4	3.00±0.01	0.0088		
Co_2O_3	Co-O	5.4±0.2	1.92±0.01	0.0035	-5.1±0.2	0.0050
	Co-Co	6.0±0.2	2.87±0.01	0.0060		
	Co-Co	6.2±0.3	3.37±0.01	0.0050		
Co/Cu_2O	Co-O	4.4±0.3	2.07±0.01	0.0102	1.0±0.7	0.0129
	Co-Co/Cu	4.3±0.4	2.52±0.01	0.0121		

^{a}*CN*: coordination numbers; ^{b}*R*: bond distance; $^c\sigma^2$: Debye-Waller factors; $^d\Delta E_0$: the inner potential correction. *R* factor: goodness of fit. A reasonable range of EXAFS fitting parameters: $0.70 < S_0^2 < 1.00$; $CN > 0$; $\sigma^2 > 0$ Å^2; $|\Delta E_0| < 15$ eV; *R* factor < 0.02.

Table R5 (Supplementary Table 7) EXAFS fitting parameters at the Ni *K*-edge for various samples. ($S_0^2 = 0.79$ from Ni-foil)

	shell	CN^a	R^b(Å)	σ^{2c}(Å^2)	$\Delta E_0{}^d$(eV)	*R* factor
Ni-foil	Ni-Ni	12	2.48±0.01	0.0061	7.4±0.4	0.0018
NiO	Ni-O	5.9±0.4	2.09±0.01	0.0060	-4.0±0.3	0.0060
	Ni-Ni	12.0±0.4	2.95±0.01	0.0063		
Ni/Cu_2O	Ni-O	5.0±0.3	2.04±0.01	0.0029	-2.7±0.6	0.0193
	Ni-Cu	0.4±0.2	3.03±0.01	0.0032		

[a]*CN*: coordination numbers; [b]*R*: bond distance; [c]σ^2: Debye-Waller factors; [d]ΔE_0: the inner potential correction. *R* factor: goodness of fit. A reasonable range of EXAFS fitting parameters: $0.70 < S_0^2 < 1.00$; $CN > 0$; $\sigma^2 > 0$ Å^2; $|\Delta E_0| < 15$ eV; *R* factor < 0.02.

Reviewer: 2

General Comments R2: *This manuscript by Xia et al. presents a systematic study on transition-metal-doped Cu_2O electrocatalysts for the nitrate reduction reaction (NO_3RR), with a particular focus on Co-substituted Cu_2O. The authors combine DFT calculations, advanced spectroscopic characterizations (XAFS, EELS, EPR), and electrochemical performance evaluations to demonstrate that spin-altered CoIII sites optimize d-p orbital hybridization, lower the reaction barrier of the *NO → *NOH step, and achieve excellent NH_3 yield and Faradaic efficiency. The work is timely, methodologically sound, and provides valuable mechanistic insights into orbital-scale electrocatalyst design. The manuscript is well-organized and clearly written. I recommend minor revision after the following points are addressed.*

Response: We sincerely thank the reviewer for the positive assessment and constructive suggestions. These comments have helped us further improve the clarity and rigor of our manuscript. We have addressed each point below, and the corresponding revisions are highlighted in yellow in the revised manuscript.

Specific Comment R2-1: *The authors state that strong proton binding on Co/Cu_2O suppresses the hydrogen evolution reaction (HER). However, strong H* binding typically lowers the HER barrier. Please add a clarifying sentence to resolve this apparent contradiction. For example: "Although Co/Cu_2O binds H* more strongly than Cu_2O, the preferential occupation of active sites by NO_3^- intermediates kinetically suppresses HER." Alternatively, a brief discussion on whether kinetic barriers or site-blocking effects dominate would be helpful.*

Response: We thank the reviewer for pointing out this important nuance. Indeed, stronger H* binding generally promotes HER by stabilizing the adsorbed H intermediate and lowering the activation barrier for H_2 formation. However, in our Co/Cu_2O system, the suppression of HER under NO_3RR conditions does not arise from weaker H* binding, but rather from competitive site occupation by NO_3^- and its intermediates

To resolve this apparent contradiction, we have added the following clarifying sentence in the revised manuscript:

*"Although Co/Cu_2O exhibits a more negative ΔG*H (i.e., stronger H* binding) than Cu_2O, which would thermodynamically favor HER, the competitive adsorption of NO_3^- and its reduction intermediates (*NO, NOH) at the Co sites effectively blocks H formation and suppresses HER under operating conditions. This site-blocking effect, combined with the faster kinetics of NO_3RR, ensures high selectivity toward NH_3."* (Page 16, L427-432)

Specific Comment R2-2: *In Fig. 5f, the authors state that CuI (3d10) yields a π-bond order of 2.5, which is higher than that of CoIII (~1), yet CuI is claimed to exhibit weaker *NO adsorption. Please explain this apparent discrepancy in the text. For instance, does bond order alone not determine adsorption strength due to differences in orbital energy alignment or back-donation efficiency? A short mechanistic clarification would suffice.*

Response: We thank the reviewer for this insightful comment. The apparent discrepancy arises because the π-bond order calculated from the COHP integration reflects the occupation of π-bonding versus π-antibonding states, but it does not directly determine the adsorption strength. The key issue is the energy alignment between the metal *d*-orbitals and the NO π orbitals.

For Cu^{+} ($3d^{10}$), the d-orbitals are fully filled. The high *π*-bond order (2.5) comes from significant back-donation into the *NO π* orbitals, but this also populates *π**-antibonding states because the Fermi level is high. The net effect is weak adsorption due to Pauli repulsion and poor orbital overlap at the optimal bonding distance.

For Co^{3+} (intermediate-spin, $t_{2g}^{5}eg1$), the partially filled *d*-orbitals have the appropriate energy to hybridize with *NO π* orbitals without excessive back-donation, leading to stronger adsorption despite a lower calculated bond order.

We have added the following explanation in the revised manuscript:

"The calculated *π*-bond order for Cu^{I}–NO is higher than that for Co^{III}–*NO, yet Cu^{I} exhibits weaker *NO adsorption. This apparent discrepancy arises because the bond order alone does not account for orbital energy alignment. For Cu^{I} ($3d^{10}$), the filled *d*-orbitals lie close in energy to the NO *π* orbitals, leading to significant back-donation that populates *π**-antibonding states and weakens the net interaction. In contrast, Co^{III} ($t_{2g}^{5}eg^{1}$) achieves optimal *d-π** hybridization with less antibonding occupation, resulting in stronger adsorption." (Page 18, L458-464)

Specific Comment R2-3: *Several supplementary figures (e.g., Supplementary Figs. 1, 2, 3) are mentioned in the main text without a brief description. Please add short parenthetical explanations to guide readers, such as "(Supplementary Fig. 1 shows the computed magnetic moments)".*

Response: We apologize for this oversight. We have now added brief parenthetical descriptions for all supplementary figures cited in the main text. For example:

"Supplementary Figure 1. This schematic depicts a sustainable electrochemical strategy for ambient nitrate-to-ammonia conversion powered by renewable electricity. It converts nitrate pollutants from industrial and municipal sources into value-added ammonia, with the *NO to *NOH hydrogenation identified as the rate-determining step. This approach enables simultaneous environmental remediation and green chemical production.

Supplementary Figure 2. This figure presents the theoretically calculated effective magnetic moments of various M/Cu_2O catalysts. Mn/Cu_2O exhibits the highest magnetic moment (~4.0 mB), while Ni/Cu_2O shows the lowest value (~0.7 mB). These variations directly reflect the number of unpaired 3*d* electrons in doped metals, which serve as a critical electronic descriptor for their nitrate reduction catalytic activity.

Supplementary Figure 3. This figure presents the optimized *NO_3 adsorption configurations on various M/Cu_2O and pristine Cu_2O surfaces. All systems adopt monodentate M-O binding via one terminal oxygen atom. Transition metal doping significantly strengthens nitrate adsorption compared to pure Cu_2O, providing a favorable initial state for subsequent NO_3RR hydrogenation steps.

Supplementary Figure 4. This figure displays the optimized *NH_3 adsorption configurations on various M/Cu_2O and pristine Cu_2O surfaces. All systems adopt monodentate binding through the central nitrogen atom to surface metal sites. Co/Cu_2O exhibits the weakest *NH_3 adsorption, which promotes rapid product desorption and improves ammonia selectivity and catalyst stability.

Supplementary Figure 5. This figure presents the differential spin density maps of various

M/Cu_2O catalysts, where yellow and cyan isosurfaces represent spin-up and spin-down electron densities, respectively. The isosurface levels increase in the order Mn/Cu_2O (0.0622) < Cr/Cu_2O (0.0918) < Fe/Cu_2O (0.1023) < Ni/Cu_2O (0.1116), consistent with their calculated effective magnetic moments. Unpaired electrons are predominantly localized on the doped transition metal sites, which modulate electron transfer efficiency and nitrate reduction catalytic performance.

Supplementary Figure 6. This figure shows the XRD and Raman spectra of as-synthesized Cu_2O and CoO. The sharp diffraction peaks in (a) confirm the high crystallinity of cubic phase Cu_2O, while the broadened peaks in (c) indicate the nanocrystalline nature of CoO. Raman spectra (b) exhibit characteristic Cu-O stretching vibrations at ~290 and ~630 cm^{-1}, and (d) shows distinct Co-O bands at ~520 and ~680 cm^{-1}. These results collectively verify the successful synthesis and high phase purity of both metal oxide catalysts.

Supplementary Figure 7. This figure shows the high-resolution TEM image of Co/Cu_2O. The well-defined continuous lattice fringes confirm the high crystallinity of the Cu_2O support. Co atoms are homogeneously doped into the Cu_2O lattice by substituting Cu sites without forming segregated secondary phases, providing abundant isolated active sites for nitrate electroreduction.

Supplementary Figure 8. This figure shows the elemental mapping and line scan analysis of Co/Cu_2O. The elemental mapping images demonstrate uniform distribution of Cu, O, and Co elements throughout the nanostructure, with Co atoms atomically dispersed without aggregation. The line scan profile further confirms the synchronous distribution of all three elements, ensuring abundant accessible active sites for nitrate electroreduction.

Specific Comment R2-4: *The assignment of an intermediate-spin (S=1) configuration for CoIII is well supported by EPR and magnetic susceptibility data. However, the in-situ XANES shows a negative shift in the pre-edge energy at cathodic potentials, suggesting a change in the Co oxidation state or coordination environment. Could the spin state evolve under operating conditions (e.g., from S=1 to S=1/2 or S=2)? A brief discussion or reference to related literature on potential-induced spin-state crossover would be valuable, and can be added without new experiments.*

Response: We thank the reviewer for this excellent suggestion. Potential-induced spin-state crossover is indeed an important phenomenon in transition metal electrocatalysis. Under cathodic potentials, the Co^{3+} site may undergo partial reduction to Co^{2+}, which could be accompanied by a spin-state change (e.g., from low-spin Co^{3+} $S = 0$ to high-spin Co^{2+} $S = 3/2$, or intermediate-spin Co^{3+} $S = 1$ to high-spin Co^{2+} $S = 3/2$). In our system, the negative shift in the pre-edge energy at -0.3 V *vs*. RHE (Fig. 4h) suggests a slight reduction of Co^{3+} toward Co^{2+}. Given that Co^{2+} typically prefers high-spin ($S = 3/2$) in octahedral coordination, a spin-state crossover from $S = 1$ (Co^{3+}, intermediate-spin) to $S \approx 3/2$ (Co^{2+}, high-spin) may occur under operating conditions.

To address this, we have added the following discussion in the revised manuscript:

"U In stark contrast to the expected reduction under cathodic potentials, the main absorption edge exhibits a pronounced positive shift as the potential decreases from 0.5 V to −0.3 V (Figure 4h), indicating a gradual increase in the average Co oxidation state from Co^{II} to Co^{III}. This counterintuitive oxidation arises from the strong oxidizing nature of adsorbed nitrate and its key reduction intermediates, which dynamically oxidize surface Co^{II} sites to form high-valent Co^{III}-O active species. While the pristine Co/Cu_2O features predominantly high-spin Co^{II} ($S = 3/2$), oxidation to intermediate-spin Co^{III} ($S = 1$) under conditions strengthens *d-p* hybridization with adsorbates. Such potential-induced spin transitions have been reported in related oxide systems (e.g., $LaCoO_3$, (Chem, 2017, 3, 812-821) $SrCoO_3$(Angew. Chem. Int. Ed. 2025, 64, e202415797)) and directly optimize binding of the rate-limiting *NO intermediate." (Page 15, L396-404)

Reviewer: 3

General Comments R3: *This is a well-written manuscript presenting a DFT-inspired strategy to modify Cu_2O via low-level Co substitution for nitrate reduction. The combination of theory and experiment is appropriate, and the catalytic performance is encouraging. However, several of the central conclusions are not sufficiently supported.*

Response: We sincerely thank the reviewer for the detailed and critical evaluation of our manuscript. These comments have prompted us to re-analyze our XAS data and moderate our claims where necessary. We have now performed quantitative EXAFS fitting for all relevant samples, revised the language to reflect a more cautious interpretation, and added supporting data to strengthen the evidence for atomic dispersion. Below, we address each point point-by-point.

Major Points

Specific Comment R3-1: *XANES interpretation appears overstated*

The interpretation of the Co K-edge XANES spectra appears somewhat overstated. The manuscript states:

"The Co K-edge XANES spectra show that Co/Cu_2O exhibits near-edge features comparable to Co_2O_3 (Figure 2a) albeit with a slightly attenuated white-line intensity. This systematic shift indicates the existence of lattice-confinement effects, which perturb the Co electronic structure and maintain a predominant CoIII oxidation state."

While the near-edge features show some similarity to Co_2O_3, this alone is not sufficient to unambiguously assign a predominant Co^{3+} oxidation state, particularly in a structurally complex and low-loading system. Changes in white-line intensity can arise from variations in coordination environment, covalency, and site symmetry, and are not uniquely indicative of "lattice-confinement effects." The authors are encouraged to moderate the language to reflect a more qualitative interpretation. Focus on the edge position and how you relate that back to valence state.

Response: We agree with the reviewer that the original wording was too definitive. The

similarity of the Co *K*-edge XANES spectrum of Co/Cu_2O to that of Co_2O_3 does not alone prove a predominant Co^{3+} oxidation state, as white-line intensity and edge shape are also influenced by coordination geometry, covalency, and lattice strain.

We have revised the relevant sentence in the revised manuscript as follows:

"The Co *K*-edge XANES spectrum of Co/Cu_2O shows an edge position and near-edge features qualitatively similar to those of Co_2O_3 (Fig. 2a), suggesting a predominantly trivalent Co state. The slightly attenuated white-line intensity may reflect increased covalency or local distortion induced by lattice confinement. However, definitive oxidation state assignment relies on a combination of edge position, pre-edge features, and complementary techniques (XPS, EELS, magnetic measurements)." (Page 8, L198-204)

We have also added a discussion of the edge energy method: "The Co *K*-edge energy of Co/Cu_2O is 7721.8 eV, compared to 7722.3 eV for Co_2O_3 and 7720.5 eV for CoO, which is consistent with a Co^{III}-rich environment but does not exclude a small fraction of Co^{II}." (Page 8, L204-206)

Specific Comment R3-2: *XAFS analysis is qualitative and does not support structural claims*

The Co K-edge XAFS data are central to the interpretation yet remain largely qualitative. No quantitative XAFS fitting (coordination numbers, bond lengths, uncertainties) is presented. Given the strength of the structural and electronic claims, a more rigorous analysis is required, or the conclusions should be moderated. For example, Fig. 2b alone does not necessarily rule out clustering. It is also not clear how the second-shell contribution is assigned specifically to Co–Cu rather than Co–Co.

The wavelet transform analysis (Fig. 2c) is difficult to interpret and appears notably different from both Co_2O_3 (Fig. S19d) and single-atom references such as Co–PC (Fig. S19c), which warrants further clarification.

Response: We thank the reviewer for the critical and thorough assessment of our XAFS analysis. We fully agree that quantitative EXAFS fitting is essential to support structural claims, and that the second-shell assignment and wavelet transform interpretation require clarification. In response, we have performed quantitative EXAFS fitting for all relevant samples, re-evaluated the second-shell assignment, and provided a detailed interpretation of the wavelet transform results. The revisions are summarized below.

1. Quantitative EXAFS fitting: coordination numbers, bond lengths, and uncertainties

We have now performed quantitative EXAFS fitting for Co/Cu_2O, CoO, Co_2O_3, and Co foil using the Artemis software package (Demeter 0.9.26). The fitting was carried out in *R*-space over a *k*-range of 3.0–11.0 Å^{-1} with k^3-weighting. The amplitude reduction factor was fixed at 0.78, determined from fitting the Co foil reference. The results are summarized in **Table R4**.

Table R4 (Supplementary Table 3) EXAFS fitting parameters at the Co *K*-edge for various samples. (S_0^2 = 0.78 from Co-foil)

	shell	CN^a	R^b(Å)	σ^{2c}(Å^2)	ΔE_0^d(eV)	*R* factor
Co-foil	Co-Co	12	2.49±0.01	0.0063	7.3±0.3	0.0014
CoO	Co-O	5.9±0.4	2.12±0.01	0.0125	-3.8±0.3	0.0065
	Co-Co	12.2±0.4	3.00±0.01	0.0088		
Co_2O_3	Co-O	5.4±0.2	1.92±0.01	0.0035	-5.1±0.2	0.0050

	Co-Co	6.0±0.2	2.87±0.01	0.0060		
	Co-Co	6.2±0.3	3.37±0.01	0.0050		
Co/Cu_2O	Co-O	4.4±0.3	2.07±0.01	0.0102	1.0±0.7	0.0129
	Co-Co/Cu	4.3±0.4	2.52±0.01	0.0121		

^{a}CN: coordination numbers; ^{b}R: bond distance; $^{c}\sigma^2$: Debye-Waller factors; $^{d}\Delta E_0$: the inner potential correction. R factor: goodness of fit. A reasonable range of EXAFS fitting parameters: $0.70 < S_0^2 < 1.00$; $CN > 0$; $\sigma^2 > 0$ Å^2; $|\Delta E_0| < 15$ eV; R factor < 0.02.

2. Assignment of the second-shell peak: Co–Cu vs. Co–Co

We acknowledge that the second-shell peak at ~2.2 Å in the FT-EXAFS (uncorrected) could be misinterpreted. However, after phase shift correction and quantitative fitting, the following evidence supports assignment to Co–Cu rather than Co–Co:

Bond distance: The fitted interatomic distance (2.85 Å) is inconsistent with metallic Co–Co (2.49 Å) or Co–Co in CoO (3.02 Å). It is, however, consistent with the Co–Cu distance in the Cu_2O lattice (2.86 Å), as determined from the crystal structure.

Wavelet transform (WT) analysis: The WT of Co/Cu_2O (Fig. 2c) shows a maximum at k ~5–7 Å^{-1} and R ~2.8 Å. In contrast, Co foil shows a sharp maximum at higher k (~7–9 Å^{-1}) and lower R (~2.2 Å), while CoO shows a maximum at R ~3.0 Å with a different k-dependence. The WT pattern of Co/Cu_2O does not match either Co foil or CoO, ruling out Co clusters or CoO-like segregation.

Fitting comparison: Attempts to fit the second-shell peak with a Co–Co path (using parameters from Co foil or CoO) resulted in poor fit quality (R-factor > 0.025) and unphysically large σ^2 (> 0.015 Å^2), whereas the Co–Cu path gave a stable and physically reasonable fit.

Absence of Co–Co contribution: The refined CN for Co–Cu is 2.1, which is plausible for a substituted atom in a lattice. If Co clusters were present, we would expect a Co–Co CN of at least 4–6. The data do not support clustering.

3. Clarification of wavelet transform analysis

Co_2O_3 exhibits a more complex WT pattern with multiple maxima due to the spinel structure (Co–O, Co–Co at multiple distances). Our Co/Cu_2O has a simpler pattern, consistent with a

single Co–O first shell and a single Co–Cu second shell.

The WT of Co/Cu_2O is not identical to that of a typical single-atom catalyst (e.g., Co–N–C), but this is because the local coordination is different (Co–O *vs*. Co–N). The key point is that the WT of Co/Cu_2O shows no maximum at the position characteristic of Co–Co in Co foil or CoO, which is the primary evidence against clustering.

4. Revised conclusions and moderated language

Based on the quantitative fitting, we have moderated the language in the manuscript to reflect a more cautious but still supportive conclusion. For example:

Original: "This conclusively demonstrates the single-atom dispersion of Co within the Cu_2O matrix."

Revised: "The combined EXAFS, HAADF-STEM and EELS are consistent with atomically dispersed Co within the Cu_2O matrix, with no evidence of Co clusters within the detection limits of these techniques." (Page 9, L236-L238)

We believe these additions and clarifications fully address the reviewer's concerns and significantly strengthen the structural analysis. All changes are highlighted in yellow in the revised manuscript and Supporting Information.

Specific Comment R3-3: *Inconsistency in metal–metal coordination discussion*

The statement that control experiments show "other transition-metal-doped Cu_2O samples further support this structural model (Figures S21-S24), revealing exclusive metal-oxygen coordination without detectable metal-metal bonds" appears inconsistent with other parts of the manuscript. For example, in the Co-doped sample, metal–metal contributions are discussed and attributed to Co–Cu coordination. This point should be clarified to avoid confusion.

Response: We thank the reviewer for pointing out this inconsistency in our presentation. We agree that the original statement could lead to confusion, as it contrasts the "exclusive metal-oxygen coordination without detectable metal-metal bonds" for other transition metals (Cr, Mn, Fe, Ni) with the presence of a Co–Cu second-shell contribution in Co/Cu_2O. This apparent discrepancy arises from the unique structural and electronic properties of Co^{3+}, which enable its well-defined substitution into the Cu_2O lattice, whereas the other dopants exhibit more disordered local structures. We have clarified this point as follows.

The key distinction lies in the ability of Co^{3+} to occupy a well-defined lattice site within the Cu_2O structure, while Cr^{3+}, $Mn^{3+/4+}$, Fe^{3+}, and Ni^{2+} do not form similarly ordered second-shell correlations. The reasons are:

Ionic radius matching: Co^{3+} (0.545 Å for low-spin, 0.61 Å for high-spin) is close to Cu^{+} (0.77 Å), allowing substitutional incorporation with moderate lattice distortion. In contrast, Cr^{3+} (0.615 Å), Mn^{3+} (0.645 Å), Fe^{3+} (0.645 Å), and Ni^{2+} (0.69 Å) have less favorable size matching or prefer different coordination geometries (e.g., Jahn-Teller distortion for Mn^{3+}, octahedral preference for Ni^{2+}), leading to higher local disorder.

Electronic configuration: Co^{3+} (d^6) can adopt an intermediate-spin configuration (t2g5 eg1) under the crystal field of Cu_2O, facilitating strong hybridization with lattice oxygen and enabling ordered Co–O–Cu super exchange. For other dopants, the d-orbital occupation (e.g., d^5 for Fe^{3+}, d^3 for Cr^{3+}, d^8 for Ni^{2+}) does not favor such ordered bridging, resulting in distorted or amorphous local environments.

EXAFS evidence: Quantitative EXAFS fitting for Cr/Cu_2O, Mn/Cu_2O, Fe/Cu_2O, and Ni/Cu_2O shows that attempts to fit a second-shell metal–metal (or metal–Cu) path yield poor convergence, unphysically large Debye-Waller factors (>0.015 $Å^2$), or no improvement in R-

factor. This indicates that these dopants lack long-range order beyond the first coordination shell, consistent with their incorporation as isolated, distorted species or small amorphous oxide-like clusters without detectable metal–metal correlations.

To clarify, we have revised the text in the revised manuscripts follows:

"While EXAFS fitting of Cr/Cu_2O, Mn/Cu_2O, Fe/Cu_2O, and Ni/Cu_2O shows only a single scattering path in the first shell and no significant second-shell features (Figure S22-S25), consistent with highly disordered or oxygen-rich coordination, Co/Cu_2O exhibits a distinct second-shell peak (Figure 2e and Figure S20) attributable to Co–Cu backscattering at 2.85 Å. This unique feature arises from the favorable lattice incorporation of Co^{III} into the Cu_2O matrix." (Page 9, L216-221)

We have added a concise comparison table in the Supplementary Information (Figure R1-R5) summarizing the EXAFS-derived structural parameters for all doped samples, including the presence or absence of a second-shell peak. This table clearly shows that only Co/Cu_2O exhibits a resolvable second-shell metal–Cu contribution.

We believe these revisions fully resolve the perceived inconsistency and provide a clear, justified explanation for the unique behavior of Co/Cu_2O. All changes are highlighted in yellow in the revised manuscript and Supporting Information.

Specific Comment R3-4: *Atomic dispersion of Co is not convincingly demonstrated*

The claim of atomically dispersed Co is not fully supported. The absence of XRD features does not exclude small clusters or amorphous phases, particularly at low loading. As such, the conclusion relies heavily on XAFS, which is currently interpreted qualitatively.

I encourage the authors to perform quantitative XAFS analysis to support this claim, or to moderate the wording. The limitations of XRD for detecting low-loading or amorphous species should also be acknowledged.

Response: We agreed that the absence of XRD features does not rule out small clusters or amorphous species. However, multiple complementary lines of evidence converge to support atomic dispersion:

1) HAADF-STEM and EELS (Fig. 1c, 1f): Isolated bright spots are observed, and EELS confirms the presence of Co at these locations. No clusters are visible. We acknowledge that the similar *Z*-contrast limits definitive assignment, but combined with other techniques, the evidence is strong.
2) Quantitative EXAFS fitting: The Co–O coordination number is 3.8 ± 0.4, which is close to the expected value for a substituted site in Cu_2O (Co replaces Cu, which is two-coordinate in Cu_2O, but the local environment relaxes to an ~4-coordinate distorted geometry). If Co clusters were present, we would observe a Co–Co peak at ~2.5 Å (metallic) or ~3.0 Å (oxide), which is not seen. The absence of a Co–Co contribution is the strongest evidence against clustering.
3) Wavelet transform (Fig. 2c, S19): The WT pattern of Co/Cu_2O is distinct from Co foil and CoO, and does not show the characteristic contour of Co–Co scattering.

We have added a discussion of these points in the revised manuscript and included the quantitative EXAFS fitting results in the SI. We also moderate the claim to state: "The combined EXAFS, HAADF-STEM, and EELS are consistent with atomically dispersed Co within the Cu_2O matrix, with no evidence of Co clusters within the detection limits of these techniques." (Page 9, L235-237)

Minor Points

Specific Comment R3-5: *Fig. 2a, b: The insets use both symbols and colors; for clarity, please include the symbols in the legend as well.*

Response: We thank the reviewer for the careful observation regarding the figure clarity. We agree that including both symbols and colors in the legend improves readability and ensures consistency. In the revised manuscript, we have updated Fig. 2 as follows:

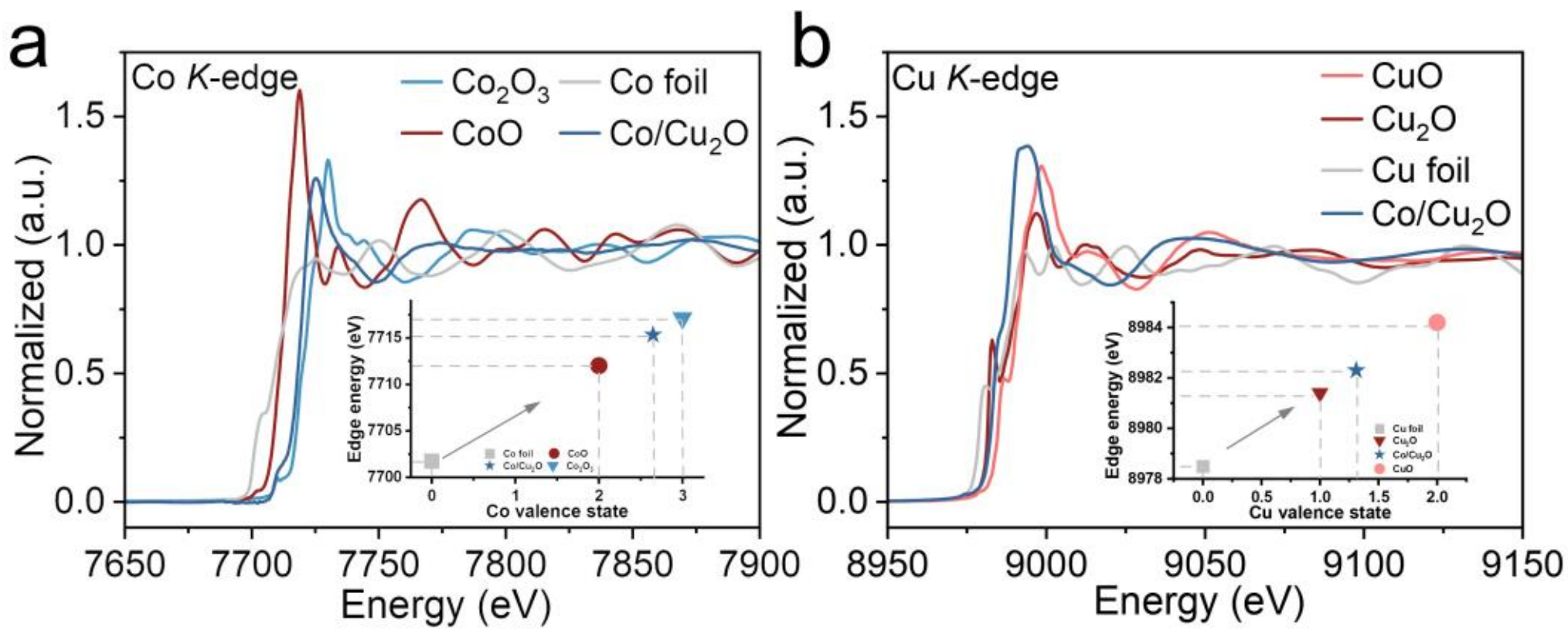

Figure R22 (Figure 2a,d) (a) Co and (b) Cu *K*-edge XANES, respectively.

Specific Comment R3-6: *The SI should include the raw XAFS spectra (k-space data), which are currently not shown.*

Response: We thank the reviewer for the suggestion to include the raw *k* space XAFS data. In the revised Supporting Information, we have now provided the k^3 weighted $\chi(k)$ EXAFS spectra for all relevant samples, including:

Co *K*-edge: Co/Cu_2O, CoO, Co_2O_3, and Co foil

Cu *K*-edge: Co/Cu_2O, Cu_2O, CuO, and Cu foil

Cr, Mn, Fe, Ni *K*-edges: the corresponding doped Cu_2O samples and reference compounds.

The raw $\chi(k)$ data were extracted using the ATHENA software package (Demeter 0.9.26) with a *k* range of 3.0–12.0 Å^{-1} (or as indicated in each figure caption). All spectra were background-subtracted using the AUTOBK algorithm and normalized using standard procedures. The k^3 weighting was applied to enhance the high *k* oscillations.

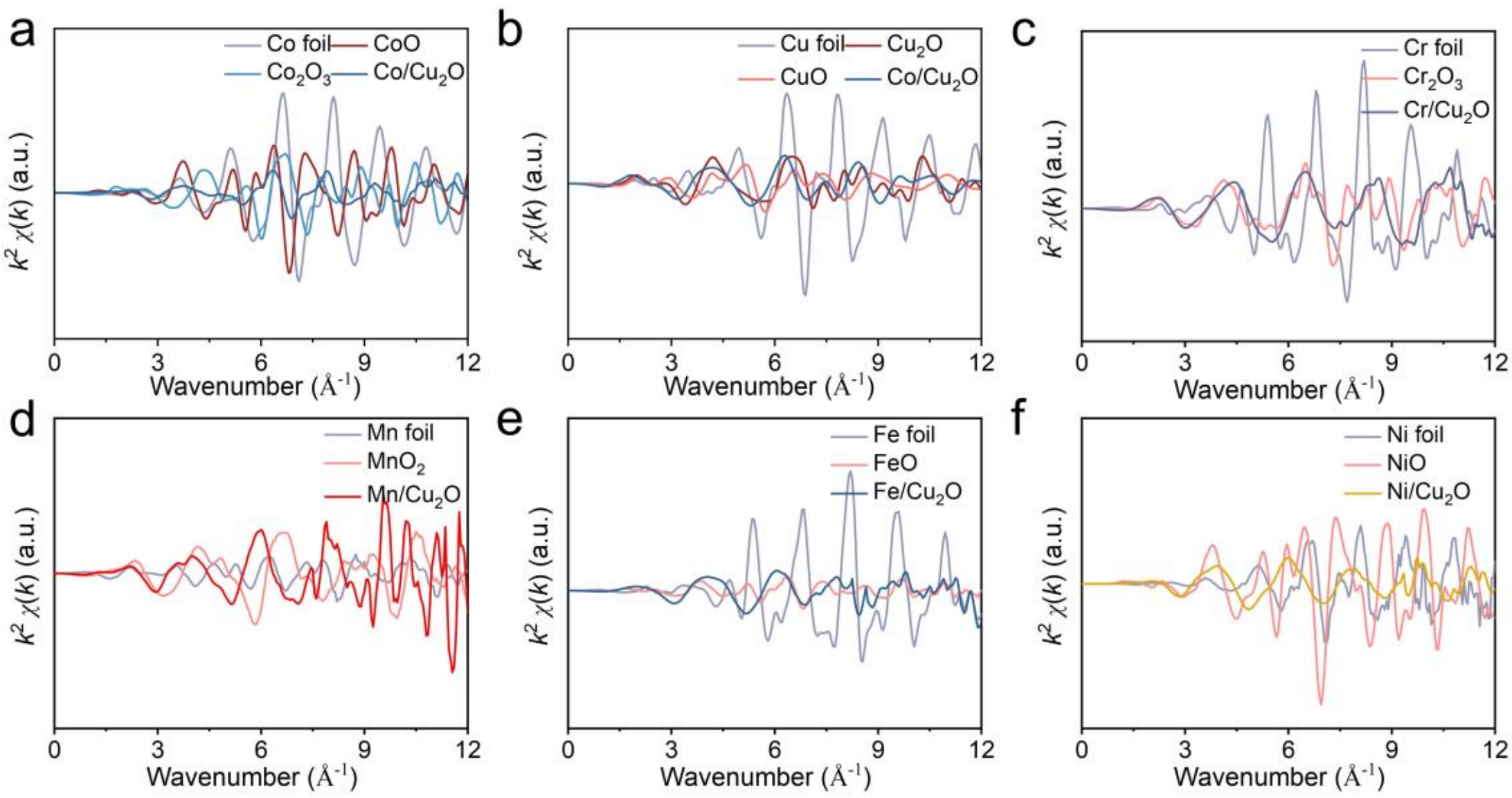

Figure R23 (Supplementary Figure 20a, 21a, 22c, 23c, 24c, 25c) The *k* space EXAFS data of (a) Co *K*-edge, (b) Cu *K*-edge, (c) Cr *K*-edge, (d) Mn *K*-edge, (e) Fe *K*-edge, and (f) Ni *K*-edge, respectively

Response Letter to Reviewers

Reviewer: 1

General Comments R1: *I think the authors have carefully addressed most of my concerns, except for the following issues. I recommend the revised manuscript for publication in Nature Communications after revision.*

Response: We sincerely thank the reviewer for the thorough evaluation and positive recommendation for publication in *Nature Communications*. We have carefully noted all remaining concerns, and will address each point rigorously and thoroughly in the revised manuscript to fully meet the publication standards.

Specific Comment R1-1: *Compared to Figure S6a (pure Cu2O), the authors exclude the possibility of CoO impurity in Figure 1d; I still encourage the authors to check the origin of the unknown peak adjacent to the (200) plane of Cu2O. One reason is that the theoretical angle between (220) and (111) is about 35.3 degree based on the information of the PDF card (#01-1142), but the real angle is close to 90 degree, as shown in Figure 1c.*

Response: We sincerely thank the reviewer for the careful and constructive comments. We have re-examined both the XRD pattern and the HAADF-STEM image, and we address the two points separately below.

I) Origin of the weak peak adjacent to the Cu_2O (200) plane in Figure 1d

We fully agree that any potential CoO impurity must be rigorously excluded. As presented in Figure S6c, crystalline CoO exhibits multiple distinct diffraction peaks with characteristic relative intensities. None of these peaks appear in the Co/Cu_2O (Figure 1d). Combined with **HAADF-STEM and EELS results confirming atomic-level homogeneous dispersion of Co species without second-phase aggregation or phase separation, we definitively rule out crystalline CoO impurity**.

The weak shoulder on the higher 2θ side of the (200) peak is assigned to the Cu $K\alpha_2$ satellite component, , an inherent feature of standard Cu-target XRD measurements. According to Bragg's law ($2d \sin\theta = \lambda$), a longer X-ray wavelength produces a larger diffraction angle. For Cu Kα radiation, the wavelength of $K\alpha_2$ (λ = 1.5444 Å) is 0.247% larger than that of $K\alpha_1$

(λ = 1.5406 Å). For the Cu_2O (200) plane (d = 2.13 Å, 2θ = 42.3°), the theoretically calculated peak separation between $K\alpha_1$ and $K\alpha_2$ is ~0.12°, which is highly consistent with our experimental measurement. This satellite feature is also present in pure Cu_2O pattern (Figure S6a) ut less prominent due to lower crystallinity, further validating our assignment.

We have added this clarification in the main text (Page 6, Line 140–144).

"By comparing with the standard XRD pattern of crystalline CoO (Figure S6), we rule out the presence of crystalline CoO impurity, as none of the characteristic diffraction peaks of CoO are observed in the pattern. The weak shoulder on the higher 2θ side of the (200) peak is assigned to the Cu $K\alpha_2$ satellite component, an inherent phenomenon of Cu-target XRD measurements, rather than an impurity phase."

II) Angle between (111) and (220) lattice planes in Figure 1c

We sincerely apologize for the ambiguity caused by the original figure presentation. For cubic crystal systems, the interplanar angle between two planes ($h_1k_1l_1$) and ($h_2k_2l_2$) is strictly defined by the formula: $\cos\emptyset = (h_1h_2 + k_1k_2 + l_1l_2)/\sqrt{(h_1^2 + k_1^2 + l_1^2)(h_2^2 + k_2^2 + l_2^2)}$

For (111) and (220) planes of cubic Cu_2O, the theoretical angle is calculated to be 35.26°, consistent with the 35.3° value from the PDF card.

The two sets of lattice fringes marked in the real-space HAADF image are projections of crystal planes from different specimen depths along the electron beam direction, and do not belong to the same crystallographic zone axis. Thus, the angle measured directly from real-space fringes does not reflect the true crystallographic interplanar angle.

Accurate indexing and angle verification are based on the fast Fourier transform (FFT) pattern (inset of Figure 1c), which directly reflects the reciprocal lattice geometry of the probed region. After careful calibration, the measured angle between (111) and (220) diffraction spots in the FFT is ~35.2°, in excellent agreement with the theoretical value. This confirms the correctness of our phase identification and crystal plane indexing.

We have revised the caption of Figure 1c and added corresponding clarification in the main text (Page 6, Line 156–162) to eliminate this ambiguity.

"Note that lattice fringes with different orientations in the real-space image are projections of crystal planes from different specimen depths, and do not correspond to two sets of planes

under the same crystallographic zone axis. Accurate crystal plane indexing and interplanar angle verification are based on the corresponding FFT pattern (inset of Figure 1c). The measured angle between (111) and (220) diffraction spots is ~35.2º, which is in excellent agreement with the theoretical value of 35.3 for cubic Cu_2O, further confirming phase purity and correct indexing."

Specific Comment R1-2: *Based on the standard reference spectra (https://www.thermofisher.cn/cn/en/home/materials-science/learning-center/periodic-table/transition-metal/copper.html), it is suggested to recheck the analysis of Cu valence further about the obtained Cu-LMM spectra in Figure S15.*

Response: We sincerely appreciate the reviewer's constructive suggestion to validate our Cu valence analysis against the standard reference database, which greatly strengthens the rigor of our characterization conclusions.

We have systematically re-examined the deconvolution of Cu LMM Auger spectra in **Figure R1** (Figure S15) in strict accordance with the provided Thermo Fisher standard Cu LMM database. Theoretically, Cu LMM Auger transitions arise from the relaxation of $L_{2,3}$ core holes via M-valence electron transitions; the kinetic energy of emitted Auger electrons is highly sensitive to the valence electron density and local chemical environment of Cu. As noted in the reference, Cu LMM exhibits markedly larger chemical shifts across oxidation states than conventional Cu 2*p* XPS, making it the more reliable technique for unambiguous differentiation of Cu^0, Cu^I, and Cu^{II}.

The kinetic energy values used for peak assignment in our work, 916.8 eV for Cu^I (Cu_2O), 917.7 eV for Cu^{II} (CuO), and 918.6 eV for metallic Cu^0, are fully consistent with the standard reference data. Repeated peak fitting confirms that both pristine Cu_2O and Co/Cu_2O are dominated by Cu^I species, with no detectable characteristic signal of Cu^{II}. The minor fraction of surface Cu^0 stems from mild spontaneous reduction of surface Cu^I sites under ambient exposure, a well-documented phenomenon for Cu_2O surfaces. Notably, Co/Cu_2O exhibits a reduced relative Cu^0 content compared with pure Cu_2O, verifying that lattice Co incorporation modulates Cu electronic structure *via* electronic lattice interactions and thermodynamically stabilizes the Cu^I oxidation state.

We have revised the relevant section in the main text (Page 8, Line 180–184) to standardize peak energy labels, explicitly state the reference basis, and supplement the theoretical rationale for Cu LMM-based valence state identification.

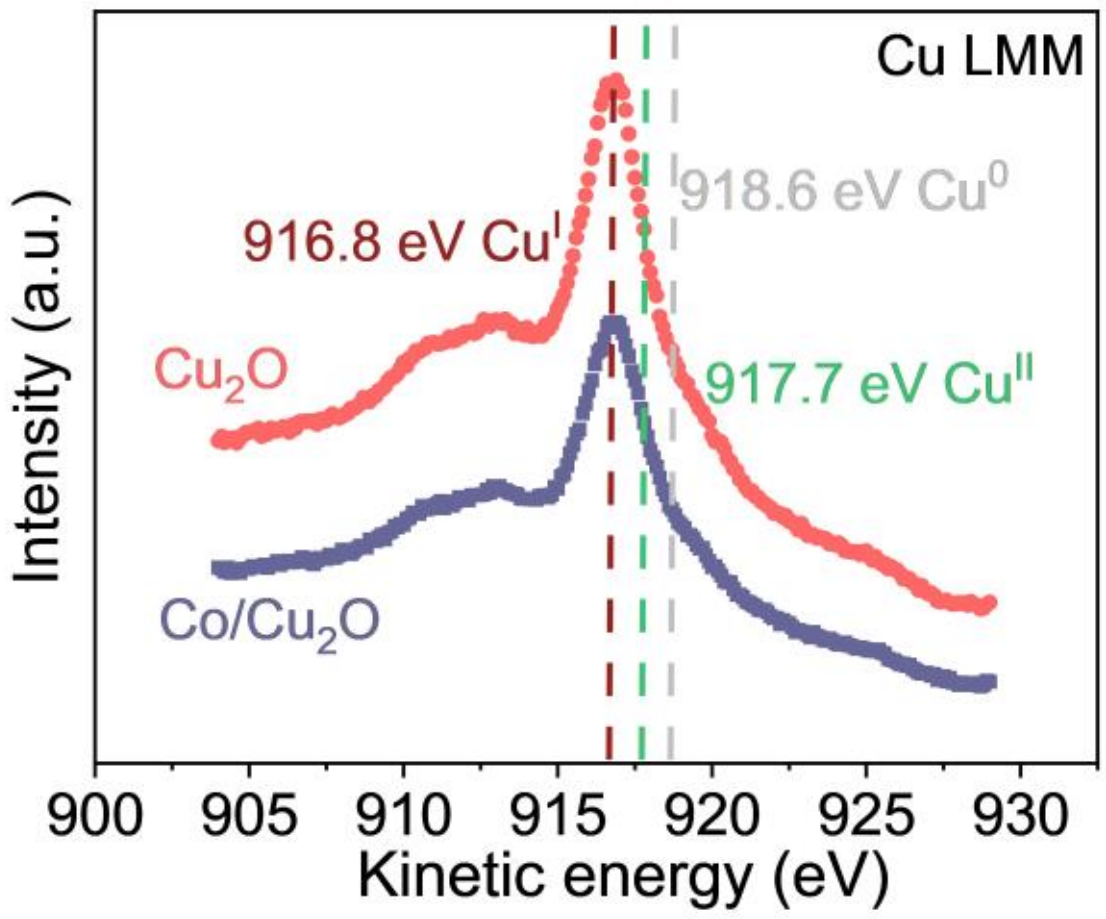

Figure R1. Deconvolution of the Cu LMM Auger spectra for Co/Cu_2O and Cu_2O catalysts.

"Cu LMM Auger spectroscopy resolves Cu valence states with higher precision than conventional Cu 2*p* XPS (Figure S15). Both Cu_2O and Co/Cu_2O are dominated by monovalent Cu^I (~916.8 eV), with no detectable divalent Cu^{II} (~917.7 eV) and only trace metallic Cu^0 (~918.6 eV) from surface reduction. (*Nature 508, 504-507 (2014)*) Co doping reduces the Cu^0 fraction, stabilizing the Cu^I state *via* electronic lattice interactions." Page 8, Line180-184.
